# Challenging the Macrocycle Paradigm: Four-Arm, High-Denticity Acyclic Chelators for Radiopharmaceuticals Incorporating Actinium and Lanthanides

**DOI:** 10.1021/acs.inorgchem.6c00216

**Published:** 2026-03-17

**Authors:** Daniel Fernández-Pavón, Andrés de Blas, María Martínez-Cabanas, José L. Barriada, Brian O. Patrick, Chris Orvig, François Bénard, Hua Yang, Luke Wharton, María de Guadalupe Jaraquemada-Peláez, Teresa Rodríguez-Blas

**Affiliations:** † Grupo METMED, Departamento de Química, 16737 Universidade da Coruña, Campus da Zapateira s/n, 15071 A Coruña, Spain; ‡ Department of Chemistry, 8166 University of British Columbia, Vancouver, BC V6T 1Z1, Canada; § Medicinal Inorganic Chemistry Group, Department of Chemistry, 8166 University of British Columbia, Vancouver, BC V6T 1Z1, Canada; ∥ Department of Molecular Oncology, BC Cancer Research Institute, Vancouver, BC V5Z 1L3, Canada; ⊥ Department of Radiology, University of British Columbia, Vancouver, BC V5Z 1M9, Canada; # Life Sciences Division, 10084 TRIUMF, 4004 Wesbrook Mall, Vancouver, BC V6T 2A3, Canada

## Abstract

As an alternative to macrocyclic chelators, a family of high-denticity, acyclic chelating agents based on picolinate arms has been devised with the aim of finding versatile chelating agents for ^225^Ac, ^161^Tb, and ^177^Lu. This family comprises six symmetrical four-arm decadentate chelators that differ in the nature of the backbone spacer, ethylene (H_4_ **tpaen**), *o*-phenylene (H_4_ **tpaopd**), 2,3-naphtylene (H_4_ **tpaond**), *m-*xylylene (H_4_ **tpamxd**), *p*-xylylene (H_4_ **tpapxd**), and 2-hydroxypropylene (H_4_ **tpadapo**), and two dissymmetrical octadentate ligands, H_3_ **tripaen** and H_4_ **asyoctapa**, structurally derived from H_4_ **tpaen** by the loss of one arm (the first) and the replacement of two vicinal picolinate pendent arms by acetate groups (the second). The coordination chemistry of each chelating ligand with nonradioactive Lu^3+^, Tb^3+^, and La^3+^ (the latter as a surrogate for [^225^Ac]­Ac^3+^) has been explored to rationalize the findings in radiolabeling and human serum stability studies. With an RCY of 96% at 10^–7^ M, which is directly comparable to the gold standard macrocyclic chelators H_2_ **macropa** and H_4_ **crown**, H_4_ **tpaond** is an excellent candidate for the development of radiopharmaceuticals based on ^225^Ac. In addition, both H_4_ **tpaen** and H_4_ **tpaopd** are also an opportunity for the ^225^Ac/^155^Tb theranostic pair. Meanwhile, H_4_ **asyoctapa** appears to be a great opportunity, not only for developing terbium radiopharmaceuticals, but also for the promising ^177^Lu/^155^Tb pair.

## Graphical abstract

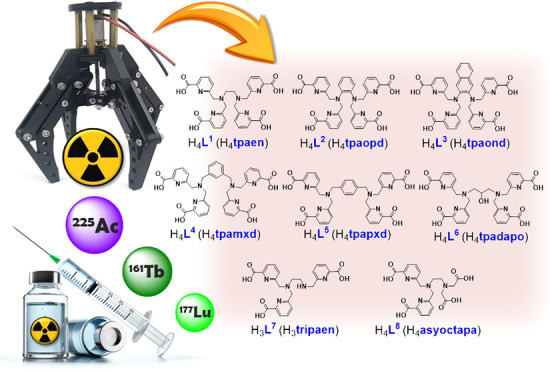

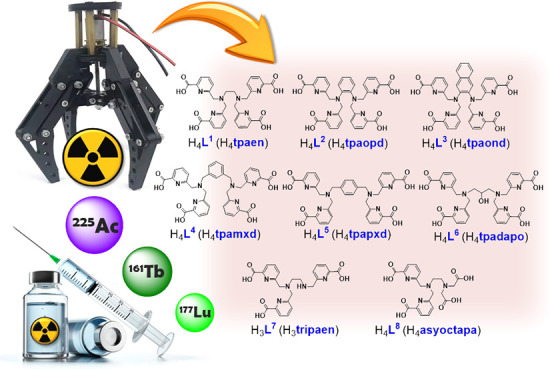

## Introduction

Undoubtedly, coordination chemistry is the cornerstone of metal-based radiopharmaceutical development because it provides the fundamental principles for creating stable complexes that bind radioactive metal ions to target molecules and direct them to specific sites in the body for diagnostic or therapeutic purposes. These principles not only allow the design of molecules that can accurately transport the metal to the desired location, but also the formation of stable coordination complexes, essential for the success of radiopharmaceuticals to prevent the metal from being released in off-target locations. As different metal ions have different coordination properties, coordination chemistry also guides the selection of suitable chelators that can effectively bind the specific radiometal ion.

In vivo stability of the resulting radiometal-chelate complex, and an efficient radiolabeling are essential requirements for chelators when designed for metal-based radiopharmaceuticals. As the labeling process uses a radionuclide at very low concentrations, a high radiolabeling yield is required, preferably achieved rapidly under mild conditions (room temperature). In the meantime, the complex must not undergo hydrolysis under physiological conditions and the chelator must bind more strongly to the radiometal than to physiological metal ions (e.g., Na^+^, Mg^2+^, K^+^, Ca^2+^, Fe^2+^, Fe^3+^, Co^2+^ or Zn^2+^), thus preventing transmetalation and the release of the radionuclide. Similarly, the radiometal ion should have a higher affinity for the chelator than for competing proteins such as transferrin and human serum albumin (HSA). Additionally, a practical and useful chelator should be easily synthesized and functionally versatile, since the chemical properties of the spacer (e.g., charges, hydrophobicity, and hydrophilicity) influence the overall pharmacokinetics.^1^ The challenge of finding a highly versatile chelating agent that can bind to different biological vectors and effectively sequester different radioactive metals of interest in nuclear medicine, forming stable complexes under biological conditions and fast complexation kinetics at room temperature, continues to attract the interest of many researchers.

In principle, radiopharmaceuticals can be designed for imaging (visualization of a radionuclide distribution in the organism) or for therapy. The final function of the drug defines the nature of the radionuclide it incorporates: γ or β^+^ radiation emitters for imaging (SPECT, PET), or α, β^–^, Auger electron emitters, which interact strongly with matter leading to low penetration, for destruction of cells. These two fields have recently merged to give rise to theranostics (therapy + diagnostics), a treatment that uses diagnostic imaging to identify whether target receptors are present, followed by precision radiation therapy that targets these receptors. Theranostics involving the use of radiolabeled agents is radiotheranostics.

Radionuclides used in nuclear medicine are produced artificially, and their availability for medical use depends heavily on factors such as production capacity and accessibility; this limits opportunities for research with them. Within this field, radiolanthanides and actinium are of great interest. In the lanthanides, radioisotopes of lanthanum, promethium, samarium, terbium, holmium, thulium and lutetium are in the spotlight. ^177^Lu (*T*_1/2_ = 6.65 days, *E*_β–,av_ = 134 keV; *E*_γ_ = 113 keV (6.17%), *E*_γ_ = 208 keV (10.36%)) was the first radiolanthanide isotope studied for radiotherapy and has been approved for clinical use in both the EU and the US, where [^177^Lu]­Lu-dota-tate (**dota-tate** = dota-(Tyr^3^)-octreotate; **dota** = 1,4,7,10-tetraazacyclododecane-1,4,7,10-tetraacetate) is indicated for the treatment of somatostatin receptor-positive gastroenteropancreatic neuroendocrine tumors (GEP-NETs). It has also been extensively studied in radiotheranostics, as the ^68^Ga/^177^Lu-theranostic pair.^2^ Among radiolanthanides, another element with remarkable potential is terbium, which presents four radionuclides of medical interest: ^149^Tb (*T*_1/2_= 4.12 h, β^+^= 7.1%, *E*_β+_ = 730 keV; α = 16.7%, *E*_α_ = 3967 keV), ^152^Tb (*T*_1/2_ = 17.5 h, β^+^ = 20.3%, *E*_β+_ = 1140 keV), ^155^Tb (*T*_1/2_= 5.23 days, *E*_γ_ = 86.6 keV (32%), *E*_γ_ = 105.3 keV (25.1%)) and ^161^Tb (*T*_1/2_ = 6.96 days, β^–^ = 100%, *E*_β–_ = 154 keV; *E*_γ and X‑ray_ ≈ 48 keV (17%), *E*_γ_ = 74.6 keV (10.3%)).^3^ Therefore, ^152^Tb is suitable for PET imaging, ^155^Tb for SPECT imaging, ^149^Tb for alpha-therapy and PET imaging, and ^161^Tb for beta-minus therapy and SPECT imaging. Moreover, in therapeutic applications ^161^Tb is considered a logical evolution from ^177^Lu, increasing the local dose deposition with respect to the latter through the coemission of short-range conversion and Auger electrons. The therapeutic benefit of ^161^Tb over ^177^Lu has been demonstrated preclinically with different compounds^4^ and a clinical SPECT/CT protocol has been proposed for imaging with ^161^Tb.^5^ These four isotopes share identical chemical properties, allowing for the creation of diagnostic and therapeutic agents with the same pharmacokinetics, while their distinct radioactive properties enable their different roles in light of their nuclear properties. Terbium is therefore an ideal option for personalized cancer treatment.^6,7^ Meanwhile, actinium, which only has radioactive isotopes, also offers a significant opportunity in nuclear medicine, in particular for radiopharmaceuticals for targeted alpha-therapy (TAT), where the isotope ^225^Ac (*T*_1/2_ = 9.9 days, α = 100%, *E*_α_ = 5600–5830 keV) represents a unique opportunity.^8^ The generation of four high-energy alpha particles through its progeny makes it extremely tumoricidal when administered and, ideally, internalized in cancer cells, where the decay products are confined. Likewise, with the gamma emission of some of its daughter nuclides such as ^221^Fr or ^213^Bi, actinium-225 provides the possibility to trace it after injection through photon coemissions.^8^ Furthermore, combinations of actinium, terbium and lutetium are interesting options for developing radiotheranostics.

Chelators for radiolanthanides must be suitable for trivalent lanthanide ions, which interact mostly electrostatically due to their high charge. According to Pearson’s classification,^9^ they are therefore classified as on the softer side of hard Lewis acids, and hard oxygen and borderline nitrogen donors are an excellent choice for incorporation into the chelator framework.^10,11^ Actinium is also found in aqueous solution as a hard-acceptor trivalent ion and its chemistry resembles that of the lanthanides, although it is somewhat more covalent. Due to their hard character, the coordination numbers (CN) of these ions are mainly dictated by their sizes. They tend to achieve high CN, which is perfectly consistent with their large size: Ac^3+^ is slightly larger than Ln^3+^ [1.065 Å (current value after revision)^12^ versus 1.032 to 0.861 Å for Ln^3+^ (La^3+^ to Lu^3+^),^13^ 6-coordinated]. Ac^3+^ prefers CN of 9 to 12, while Ln^3+^ ions are satisfied with 8 to 9 (the heavier the lanthanide, the lower the CN).^14^ Beyond high denticity and the requirement of hard donor atoms, the optimal location of these donors in the framework to maximize metal–ligand interactions as well as the introduction of steric restrictions (topology) are issues that must be considered when designing a chelator capable of achieving the perfect match. Numerous acyclic and macrocyclic aminocarboxylates, mainly with denticity eight to ten, have been proposed and studied as potential chelators for these radiometals.^15^ The octadentate macrocyclic chelator **dota**, reported in 1976 for the first time,^16^ is widely used in medical probes (US FDA-approved) and drugs nowadays as well as the most frequently used chelator in nuclear medicine. Being octadentate, it forms complexes with high stability and kinetic inertness not only with heavier Ln^3+^ but also with large Ln^3+^and with Ac^3+^. Additionally, it can be functionalized and conjugated to diverse targeting vectors (peptides, antibodies).^8,17^ The reason for this high stability and inertness stems from the preorganized macrocyclic backbone. However, the inertness of these complexes comes at expense of slow complexation kinetics. Only low radiochemical yields are obtained at room temperature, and long reaction times and/or high temperatures are required for optimal radiolabeling.^15^ These conditions are incompatible with heat-sensitive bioconjugates, such as antibodies, which rely on relatively weak domain interactions to maintain structural integrity.^18,19^ An attempt has been made to resolve this by developing a two-step radiolabeling procedure, which, however, makes the process of preparing the radiopharmaceutical tedious and complicated, and a less than optimal solution. This obvious limitation of **dota** calls into question the preference for macrocyclic architectures over acyclic ones when devising chelating agents for metal-based radiopharmaceuticals.

Based on picolinate groups, in 2009 Rodríguez-Blas and co-workers designed the well-known and renowned decadentate macrocycle *N*,*N*′-bis­[(6-carboxy-2-pyridyl)­methyl]-4,13-diaza-18-crown-6 (first named H_2_ **bp18c6**, later renamed H_2_ **macropa**), which showed unprecedented preference toward larger lanthanides.^20^ Radiolabeled with [^225^Ac]­Ac^3+^ in submicromolar concentration over 5 min at RT, the complex remained intact over 7 to 8 days and did not accumulate in any organ after 5 h in healthy mice.^21^ Nowadays **macropa** is a preferred option for ^225^Ac chelation and is currently in a clinical trial based on macropa-pelgifatamab conjugate.^22^ In 2021, Orvig and co-workers devised the potentially undecadentate acyclic chelator H_4_ **py4pa** (see Chart 1),^23^ which also possesses excellent affinity for ^225^Ac: quantitative radiochemical yields (RCYs) at RT in 30 min at 10^–6^ M chelator concentration. It is stable in mouse serum for at least 9 days, and a conjugate incorporating Trastuzumab through a short phenyl-NCS linker displayed excellent in vivo stability and tumor specificity. These findings support that a successful topology and a compromise between rigidity and flexibility that allows the metal to easily access the cavity provided by the chelating agent and remain effectively trapped thereafter must be the focus, regardless of whether the scaffold is macrocyclic or not.

**Chart 1:**
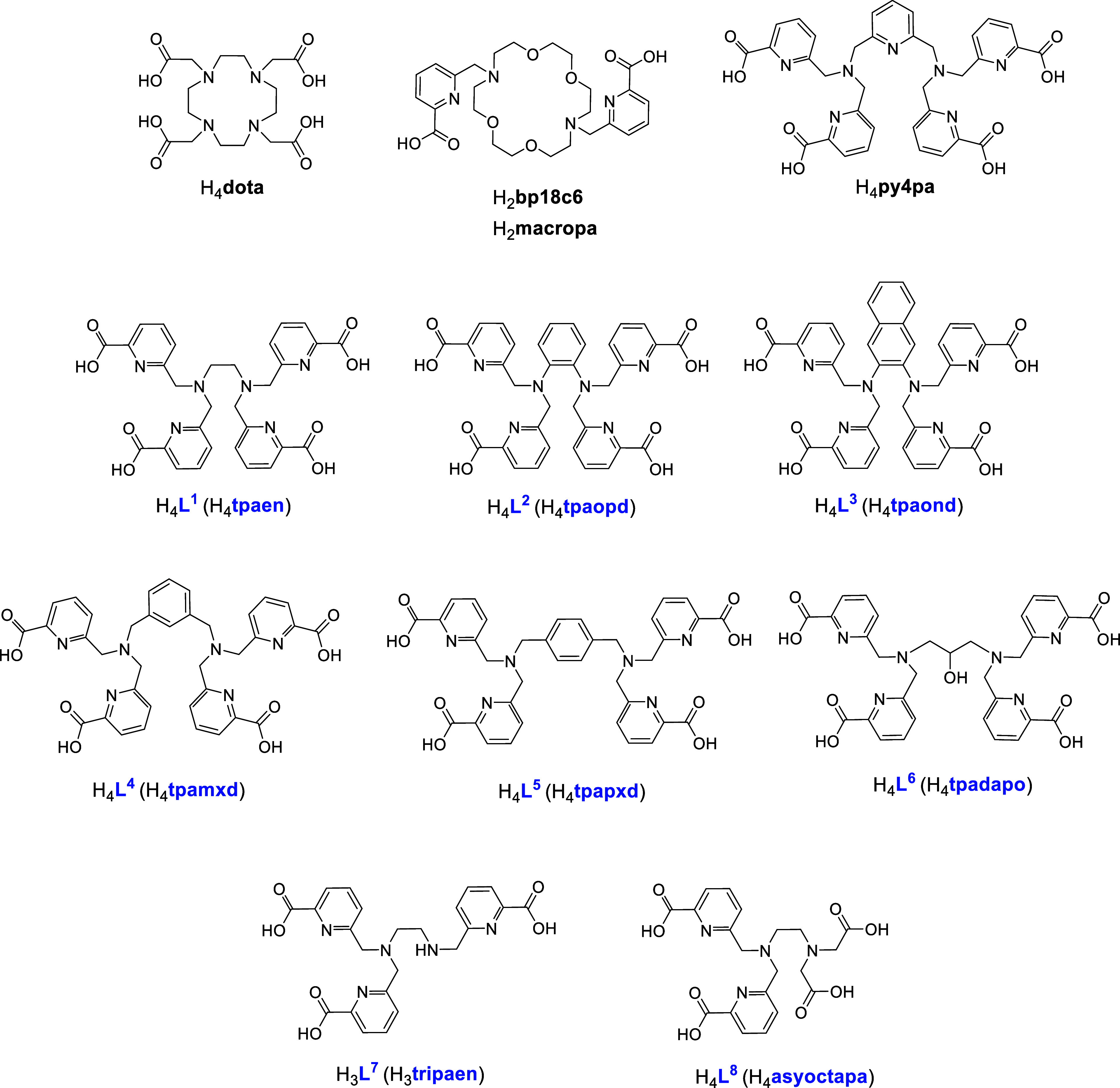
Chelators Discussed in this Paper

With the aim of finding an easy-to-prepare and versatile chelator that, like **dota**, forms stable complexes with both radiolanthanides and actinium, but unlike **dota**, demonstrates fast radiolabeling at room temperature, herein we have combined the expertise of the Coruña and Vancouver groups in the design of polydentate chelators. Finding a versatile chelating agent capable of satisfying both the coordination properties of Ac^3+^ and those of the Ln^3+^ radioactive isotopes commonly used in nuclear medicine not only reduces the radiopharmaceutical’s design cost for these radiometals, but also facilitates the development of theranostics, a fundamental technique in personalized medicine. Keeping H_4_ **py4pa** in mind, we have devised a family of decadentate tetrapicolinate chelators (H_4_ **L** ^**1**^ to H_4_ **L** ^**6**^, see Chart 1) that arises from modifying the spacer. The linker 2,5-dimethylene-pyridine has been conveniently replaced by ethylene, *o*-phenylene, 2,3-naphthylene, *m-*xylylene, *p*-xylylene or 2-hydroxypropylene groups to evaluate the impact of the spacer properties (primarily length and rigidity/flexibility) on the chelator cavity and its ability to adapt structurally to encapsulate the guest metal ion effectively. Structural and topological modifications to H_4_ **L** ^**1**^ involving the removal of one picolinate arm or the replacement of two of them with acetate groups result in the dissymmetrical octadentate chelators H_3_ **L** ^**7**^ and H_4_ **L** ^**8**^, respectively (see Chart 1). Radiolabeling studies with [^225^Ac]­Ac^3+^, [^161^Tb]­Tb^3+^, and [^177^Lu]­Lu^3+^ as well as studies of the inertness of the corresponding radioactive chelates have been performed and the results are rationalized based on a structural study of the formed complexes. The chelator H_4_ **L** ^**1**^ has already been studied in the field of nuclear fuel recycling as a possible complexing agent in solvent extraction processes for the separation of americium from fission products, including lanthanides and curium,^24–28^ and was also found to form stable complexes with Eu^3+^ and Tb^3+^, which can be used as efficiency luminescence probes.^29^ However, to date no studies have been conducted with this chelating agent for the development of radiopharmaceuticals with the radiometals of our interest, nor have any studies of any kind been carried out with any radiometal using the other chelators we have devised.

## Results and Discussion

### Synthesis and Characterization

The synthetic route followed to obtain the four-arm, decadentate ligands H_4_ **L** ^**1**^ to H_4_ **L** ^**6**^ is shown in Scheme 1. It consists of two steps starting from commercially available ethyl 6-(chloromethyl)­picolinate and the corresponding diamine; the reaction in anhydrous acetonitrile under inert atmosphere leads to an ethyl ester intermediate that, after hydrolysis with HCl (6 M), yields the desired chelator as a hydrochloride salt, as confirmed by elemental analysis. This straightforward synthetic strategy is an adaptation of that reported for H_4_ **L** ^**1**^ by Mazzanti,^29^ which we have also slightly modified. Here we have used the ethyl ester of the picolinate appended arm instead of the methyl analogue and have applied Finkelstein’s methodology,^30^ which increases the reactivity of the previously functionalized alkyl position by converting it from chloride (R-Cl) to iodide (R-I) in the presence of KI under anhydrous conditions. The decadentate chelators H_4_ **L** ^**2**^, H_4_ **L** ^**3,**^ H_4_ **L** ^**5**^ and H_4_ **L** ^**6**^, respectively denoted as H_4_ **tpaopd**, H_4_ **tpaond**, H_4_ **tpapxd**, H_4_ **tpadapo,** in line with the previous naming conversion given to H_4_ **L** ^**1**^ (H_4_ **tpaen**) that include the acronym of the corresponding diamine incorporated as spacer, are prepared and described here for the first time. H_4_ **L** ^**4**^, referred to here as H_4_ **tpamxd**, was first described by the group from Coruña ten years ago with the aim of preparing Mn^2+^-based MRI contrast agents;^31^ no studies of this chelator with *f*-block metals have thus far been reported in the literature.

**Scheme 1:**
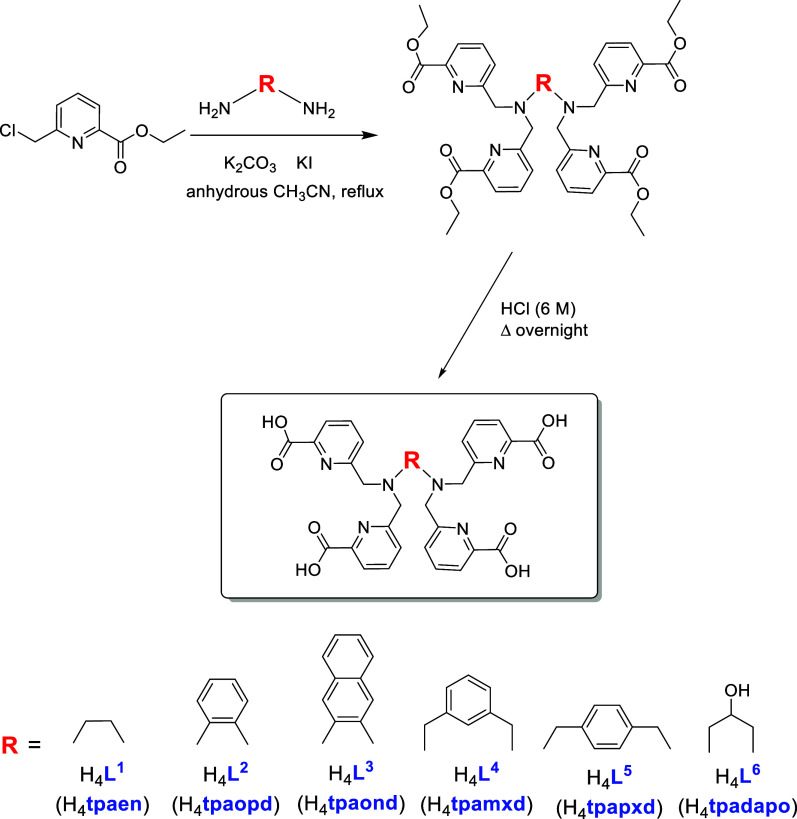
Synthetic Route for H_4_ **L** ^**1**^ to H_4_ **L** ^**6**^

The ^1^H and ^13^C­{^1^H} NMR spectra of these six chelators, recorded in D_2_O solution at 298 K, show clearly resolved signals and confirm the high symmetry of the ligands, with the four picolinate pendant arms being chemically and magnetically equivalent. All spectra were recorded at acidic pH, except for that of H_4_ **tpaond**, which was recorded at pD 12 due to its low solubility in acidic media. Assignments were made with the aid of COSY, HSQC, and HMBC two-dimensional experiments (see Figures S1–S30 in the Supporting Information and assignments in the Experimental Section). The ^13^C­{^1^H} NMR spectra display seven signals for the pendant arms and a variable number for the spacers, the latter in perfect agreement with their chemical structures (one for H_4_ **tpaen**, two for H_4_ **tpaopd**, five for H_4_ **tpaond** and H_4_ **tpamxd**, three for H_4_ **tpapxd** and two for H_4_ **tpadapo**), consistent with the presence of a *C*_2_ symmetry axis and a plane of reflection (σ) in all cases. This is also confirmed by the ^1^H NMR spectra, which show the number of expected signals. In all cases the protons of the methylene groups are magnetically equivalent except in H_4_ **tpadapo** where the CH_2_ protons of the 2-hydroxypropylene spacer (denoted Ha) experience a diastereotopic splitting [3.84 ppm (dd, *J* = 13.7, 2.8 Hz) and 3.69 ppm (dd, *J* = 13.7, 9.1 Hz)] being also coupled with the attached CH­(OH) proton. Rigidity found in this chelator seems to come from the presence of a hydrogen bond involving the hydroxyl group and both N atoms of the tertiary amines.

Single crystals suitable for X-ray diffraction of the ligand H_**4**_ **tpapxd** (H_4_ **L** ^**5**^) were grown from a solution of water/acetonitrile by slow evaporation. Crystals contain six lattice water molecules as well as the ligand in a zwitterion form (Figure 1), where both nitrogen atoms of the tertiary amines are protonated, while two carboxylate groups are anionic. This highlights the strongly basic nature of the nitrogen atoms in tertiary amines. Both ammonium groups are arranged as far away from each other as possible to keep electrostatic repulsion to a minimum and adopt an *anti* conformation with respect to the benzene ring. This arrangement is similar to that found for *p*-xylylenediaminium salts,^32–34^ although the distance between the two N atoms is slightly greater in our zwitterion than in the *p*-xylylenediaminium dication (7.607 Å vs 7.450–7.499 Å). As it can be seen in Figure 1, lattice water molecules participate in hydrogen bonding interactions among themselves, and one also acts as a bridge between the anionic picolinate group and the hydrogen atom transferred to the nitrogen of the tertiary amine (Table S3, Supporting Information). Crystal packing results from hydrogen bonding interactions as well as a π-stacking interaction between neutral picolinate groups of adjacent molecules.

**Figure 1:**
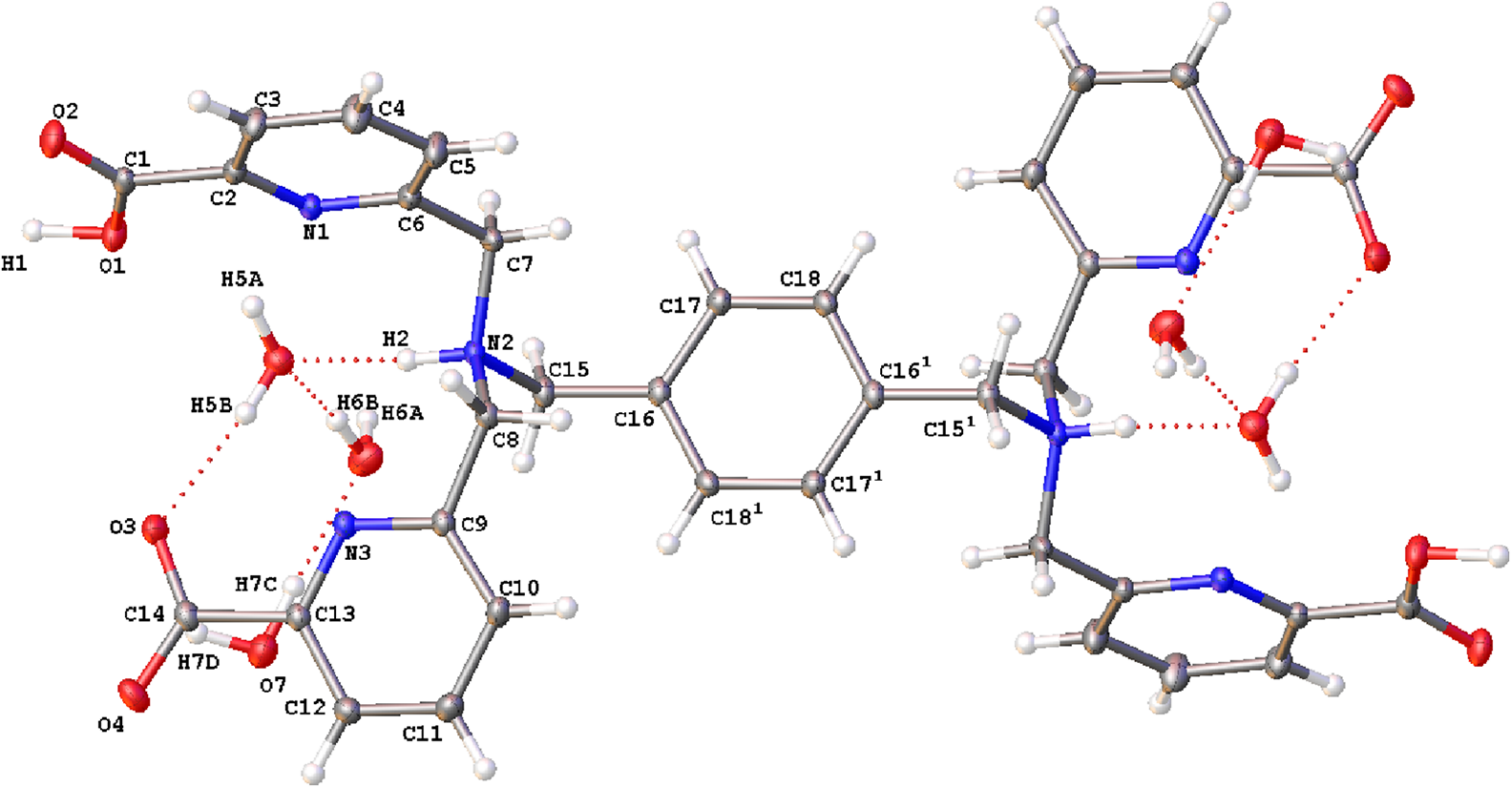
X-ray crystal structure of H_**4**_ **tpapxd**·6H_2_O with atom labeling ^1^(– *x*, 1 – *y* ,2 – *z*). Ellipsoids are shown at the 50% probability level. [Hydrogen bonding interaction lengths and angles are given in the Supporting Information (Table S3)].

The basicity of different ionizable and nonionizable protons governs the extent to which a metal ion competes with the protons for the binding sites of the chelators during metal complexation. Therefore, knowing the protonation constants is essential when evaluating any chelator for metal complexation. Bearing this in mind we determined the ligand protonation constants of the chelators H_4_ **L** ^**2**^ to H_4_ **L** ^**6**^ using potentiometric titrations (Figure S127, Supporting Information). These ligand protonation constants (*K*_*i*_) are defined in eq 1:

$$K_{i}=[H_{1}L]/[H^{+}][H_{i-1}L]$$ 1

The results (log *K*_*i*_) are shown in Table 1, which also includes the values for H_4_ **tpaen** previously reported.^29^ In order to enable meaningful comparisons of the values, the measurements were carried out using the same background electrolyte (0.10 M KCl) as was used for the H_4_ **tpaen** measurements.

**Table 1:** Protonation Constants of H_4_ **tpaopd**, H_4_ **tpaond**, H_4_ **tpamxd**, H_4_ **tpapxd**, and H_4_ **tpadapo** at 25 °C (*I* = 0.1 M KCl) Compared with Those of H_4_ **tpaen** ^a^

	H_4_ **tpaopd**	H_4_ **tpaond**	H_4_ **tpamxd**	H_4_ **tpapxd**	H_4_ **tpadapo**	H_4_ **tpaen**
log *K*_1_	10.2(2)	9.1(1)	7.4(2)	7.76(6)	7.4(1)	7.8(1)^b^
log *K*_2_	6.26(2)	6.57(3)	6.7(2)	7.04(1)	5.8(1)	5.1(1)^b^
log *K*_3_	4.87(1)	5.51(1)	4.1(2)	5.01(1)	4.1(1)	3.9(2)^b^
log *K*_4_	3.43(2)	4.49(2)	3.1(3)	3.74(1)	3.3(1)	3.2(1)^b^
log *K*_5_	3.13(3)	3.88(1)	2.7(6)	3.08(1)	3.0(2)	2.8(1)^b^

a Numbers in parentheses indicate the standard deviation of the last digit.

b Data from ref 29 (*I* = 0.1 M KCl).

These decadentate chelators possess ten protonation sites, with the exception of H_4_ **tpadapo** that has 11 because of the hydroxyl group. However, only five protonation constants were observed in the investigated pH range. The protonation constants obtained for the five chelators follow the trend of H_4_ **tpaen** as well as that observed for related four-arm ligands containing pendent picolinate groups. The first and second protonation constants correspond to the sequential protonation of the tertiary amines of the backbone, while the third, fourth, and fifth are assigned to the protonation of carboxylate groups of picolinate moieties. It was not possible to determine the p*K*_a_ for the most acidic protons as they fall below the electrode threshold (pH < 2); they include the carboxylic acid of the fourth pendent picolinate arm as well as the four protonated pyridinium nitrogen atoms in the picolinate groups, as reported in related systems.^23^

The five log *K*_a_ values of the ligand containing the spacer 1,3-xylylene (H_4_ **tpamxd**) and the previously reported H_4_ **tpaen** (with ethylene spacer) are very similar, except for log *K*_2_, which is higher in H_4_ **tpamxd** (Δlog *K*_2_ = 1.6). In both cases, the environment of both tertiary nitrogen atoms is quite similar, so it could be expected that the basicity of the two amines would be more similar in both chelators. In principle, the greater log *K*_2_ value of H_4_ **tpamxd** can be explained by the larger separation between the two nitrogen atoms afforded by the central linkage, as has been suggested for related four-arm chelators.^35^ This hypothesis is also supported by the corresponding value found for H_4_ **tpapxd**, which has a larger xylylene spacer and an even greater log *K*_2_ value (7.04(1)). On the other hand, comparing log *K*_1_ and log *K*_2_ values of H_4_ **tpamxd** with those found in the structurally related chelator H_4_ **py4pa** (log *K*_1_ = 6.96(1), log *K*_2_ = 6.07(1)),^23^ it follows that the tertiary nitrogen donors of H_4_ **tpamxd** are more basic. Although carboxylic acids also have lower p*K*_a_ values in H_4_ **py4pa**, it is nevertheless more basic (Σ log *K*_*i*_ = 28.24) than H_4_ **tpamxd** (Σ log *K*_*i*_ = 24.0) due to the pyridine nitrogen located in the spacer linker, which also provides an additional coordination site.

Taking into account the electron-withdrawing effect of the aromatic rings, log *K*_1_ would be expected to be lower in the chelators containing the *o*-phenylene and 2,3-naphthylene spacers (H_4_ **tpaopd** and H_4_ **tpaond**, respectively) than in that possessing an ethylene spacer (H_4_ **tpaen**). However, contrary to this simple expectation, the log *K*_1_ values found in the chelating agents containing rigid aromatic spacers are higher than those found for H_4_ **tpaen**. In fact, they are significantly higher. At this point, it should be remembered that the basicity of amines not only depends on electronic effects but is also affected by steric considerations, and it is the mutual electronic and steric effects which determines the final basicity. Likewise, it is conceivable that hydrogen bonds exist between the two amino groups, therefore reducing the electron density on one of the nitrogen atoms, thereby leading to an increase on the other. The latter also explains the significant difference in the protonation constant values between the two tertiary amines (log *K*_1_-log *K*_2_ = 3.89 for the ligand with the *o*-phenylene spacer and 2.57 for the ligand with the 2,3-naphthylene spacer). This difference is common and has been also found in the *o*-phenylenediamine (log *K*_1_ = 4.77, log *K*_2_ = 0.80).^36^ Notably, it has been reported that a greater difference in basicity between the tertiary amines in this type of chelator can actually be advantageous for metal coordination, whereby the less basic nitrogen favors complexation at lower pH, while the more basic nitrogen will act as a stronger donor group to metal ions.^37^

Scheme 2 illustrates the synthetic pathway followed to obtain the dissymmetric, three-armed H_3_ **L** ^**7**^ (H_3_ **tripaen**) and four-armed H_4_ **L** ^**8**^ (H_4_ **asyoctapa**) chelators, which have also been prepared for the first time here. The trialkylation of ethylenediamine required the preparation of the monoprotected intermediate with the 2-nitrobenzenesulfonamide (nosyl) group (**1**), which was synthesized following the literature.^38^ Alternatively, we have also tried monoprotection with other groups commonly used for this purpose, such as *tert*-butyloxycarbonyl (Boc) or toluenesulfonyl (Ts), but these routes proved unsuccessful. *N*-alkylation of this intermediate (**1**) with three equivalents of ethyl 6-(chloromethyl)­picolinate in the presence of K_2_CO_3_ and KI in anhydrous acetonitrile under inert atmosphere for 3 days led to (**2**), which was purified by MPLC and thereafter deprotected using thiophenol to give the ester (**3**). This ester was also purified by MPLC and its hydrolysis with HCl (6 M) yielded the expected H_3_ **tripaen** in the form of a hydrochloride salt. Mass spectrometry confirms the trialkylation (Figure S110), whereas NMR spectroscopy (Figures S33–S37, Supporting Information) indicates that the three pendant picolinate arms are magnetically nonequivalent. In the ^13^C­{^1^H} NMR spectrum (Figure S34), the methylene carbons of the pendants appear as separate resonances rather than a unique averaged signal, confirming that the three arms are chemically inequivalent. This is supported by the ^1^H NMR spectrum (Figure S33), in which these methylene protons appear as singlets at 4.90, 4.62, and 4.46 ppm. Likewise, the aromatic region displays several sets of closely spaced multiplets instead of three coincident signals for the picolinate protons In effect, this points out that nitrogen inversion or conformational exchange is slow on the NMR time scale, preserving the differentiation among the three picolinate moieties in solution.

**Scheme 2:**
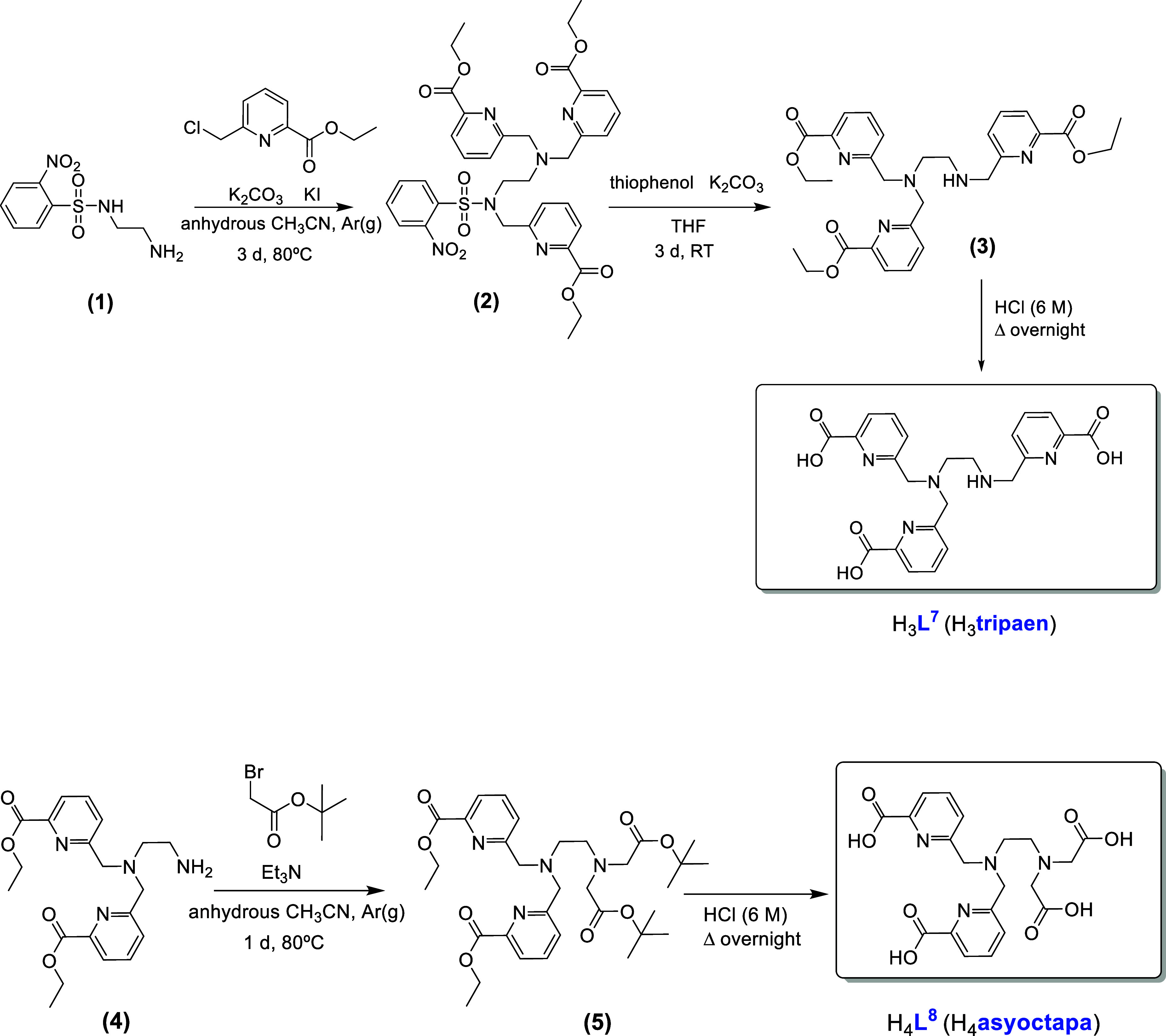
Synthetic Routes for H_4_ **L** ^**7**^ and H_4_ **L** ^**8**^

The yellow oily H_4_ **L** ^**8**^ (H_4_ **asyoctapa**) was also isolated as a hydrochloride salt. To achieve the two dissymmetrical dialkylations (2 + 2) on the ethylenediamine, the di-*N*-alkylated compound (**4**) was initially prepared following a reported strategy.^39^ Compound **4** was reacted with two equivalents of *tert*-butyl bromoacetate in the presence of triethylamine to obtain ester (**5**). Hydrolysis of (**5**) with HCl (6 M) resulted in the intended dissymmetric expected ligand as confirmed by NMR spectroscopy (Figures S39 and S40). Both the ^1^H and ^13^C spectra show two different signals for the ethylene spacer: the methylene protons of this linker appear as triplets at 3.58 and 3.62 ppm, and the corresponding carbons give signals at 51.71 and 51.29 ppm. The two pendant arms containing the picolinate groups are equivalent to each other, as are the two acetate arms.

### Study of the Coordinating Ability of Chelators with Nonradioactive Lanthanides

To ascertain whether the binding cavity of these chelators could adapt to the size and coordination preferences of the radiometals of our interest, we conducted complexation studies in both solution and solid states with the nonradioactive lanthanide ions La^3+^, Tb^3+^, and Lu^3+^. Because all actinium isotopes are radioactive, we have adopted the very common strategy of using the La^3+^ ion as a surrogate for the [^225^Ac]­Ac^3+^ ion for these studies.^21,23,40,41^ This strategy is based on the similarity in size of both ions.^12,13^ In any case, it must be pointed out that some authors who have applied it consider it debatable^41^ because the 5*f* orbitals are more diffuse than the 4*f* orbitals. The latter meaning that actinides are somewhat more covalent than lanthanides, giving as a result a slight difference in the absolute chemical hardness (Ac^3+^ = 14.4 eV, La^3+^ = 15.4 eV),^42^ which must be kept in mind when making comparisons. All the studies in solution were performed with complexes prepared in situ by mixing equimolar amounts of each ligand with the appropriate metal chloride salt in water (or water/acetonitrile) and study with HR ESI-MS. Those involving La^3+^ and Lu^3+^ were studied by NMR spectroscopy as well. The ^1^H and ^13^C­{^1^H} NMR spectra were acquired at 298 K and assigned with the aid of two-dimensional experiments (^1^H–^1^H COSY, ^1^H–^13^C HSQC and ^1^H–^13^C HMBC).

Mass spectrometry (Figures S110–S112 (La); Figures S116–S118 (Tb) and Figures S121–S123 (Lu)) clearly confirms the formation of the expected anionic [Ln­(L)]^−^ complexes for the three metal ions with the tetraanionic chelators **tpaen** (L^1^), **tpaopd** (L^2^) and **tpaond** (L^3^) at pH 6. Table 2 gives the *m*/*z* of the peaks found in ESI^–^. Experiments in ESI^+^ also show the expected peaks, but now corresponding to species [M + 2Na]^+^ (M = La-**tpaen**, La-**tpaopd**, La-**tpaond**, Tb-**tpaen**, Lu-**tpaen**), or [M + 2K]^+^ (M = Tb-**tpaopd**, Tb-**tpaond**, Lu-**tpaopd**, Lu-**tpaopd**), depending on the base used to adjust the pH (NaOH or KOH).

**Table 2:** High-Resolution Mass Spectrometry (ESI^–^) of La^3+^, Tb^3+^, and Lu^3+^ Complexes

	[**La**(L)]^−^		[**Tb**(L)]^−^		[**Lu**(L)]^−^	
	*m*/*z* experimental	*m*/*z* theoretical	*m*/*z* experimental	*m*/*z* theoretical	*m*/*z* experimental	*m*/*z* theoretical
**tpaen**	735.0724	735.0724	755.0923	755.0914	771.1075	771.1068
**tpaopd**	783.0730	783.0724	803.0922	803.0914	819.0974	819.1068
**tpaond**	833.0889	833.0881	853.1079	853.1071	869.1126	869.1225

NMR spectroscopy of the lanthanum complexes with these three chelators at pD = 6 (Figures S44–S61, Tables S4 and S5) confirms the presence of rigid mononuclear *C*_2_-symmetric [La­(L)]^−^ species with the four picolinate pendant arms coordinated to the metal ion in the NMR scale. A chiral helical structure similar to those found in solution for the Eu^3+^ and Ce^3+^ complexes with H_4_ **tpaen** ^29^ as well as for La-**py4pa** ^23^ was found. Both the ^1^H and ^13^C­{^1^H} NMR spectra of the La^3+^ complex with **tpaen** prepared by us at pD 6 are similar to those found at pD 7.4, which were published by others while this manuscript was in preparation.^43^ The three ^1^H NMR spectra are compared in Figure 2. The spectra of the metal complexes and those of their respective free ligands show that the two arms attached to the same tertiary amine nitrogen atom are no longer equivalent. Likewise, as expected, coordination causes diastereotopic splitting of all the methylene hydrogen atoms present in the ligands backbones (Hb/Hb’, Hi/Hi’, Ha/Ha’) which appear as pairs of mutually coupled doublets with distinctly different chemical shifts, as explicitly depicted in Figure 2 (see also Table S4). Although specific assignment of the axial and equatorial CH_2_ protons is impossible based on the 2D NMR spectra, it can be achieved using the stereochemically dependent proton shift effect, resulting from the polarization of the C–H bonds by the electric field effect of the cation charge. This results in a deshielding effect of the equatorial protons, which are pointing away from the metal ion.^44^ (Apostrophe denotes axial protons in Figure 2 as well as Figures S44, S50 and S56). Furthermore, the nature of the spacer has a significant effect on the chemical shifts of the methylene hydrogens of the pendant arms (Hb/Hb’; Hi/Hi’), which tend to show greater deshielding the more aromatic is the bridge (ethylene – phenylene – naphthalene). In addition, the large difference in the chemical shifts exhibited by Hi/Hi’ in the two complexes containing rigid aromatic spacer (Δδ_*i*/i’_ = 2.02 ppm in [La­(**tpaopd**)]^−^, Δδ_*i*/i’_ = 2.04 ppm in [La­(**tpaond**)]^−^) indicates that the picolinate arm containing such methylene protons is bound in a distinctly more anisotropic local environment in the complex.^45^ This pseudo *C*_2_-symmetry is also confirmed by ^13^C­{^1^H} NMR spectra (Figures S46, S52, and S58), which show 15, 17, and 19 signals for the 30, 34, and 38 carbon nuclei of **tpaen**, **tpaopd**, and **tpaond**, respectively. Each pair of diastereotopic methylene groups is associated with the chemically identical carbons [C­(b) at 62.51 ppm in La-**tpaopd**, 63.07 ppm in La-**tpaond**, and 63.38 ppm in La-**tpaen**; C­(i) at 60.85 ppm in La-**tpaopd**, 61.04 ppm in La-**tpaond**, and 62.42 ppm in La-**tpaen**; C­(a) at 59.33 ppm].

**Figure 2:**
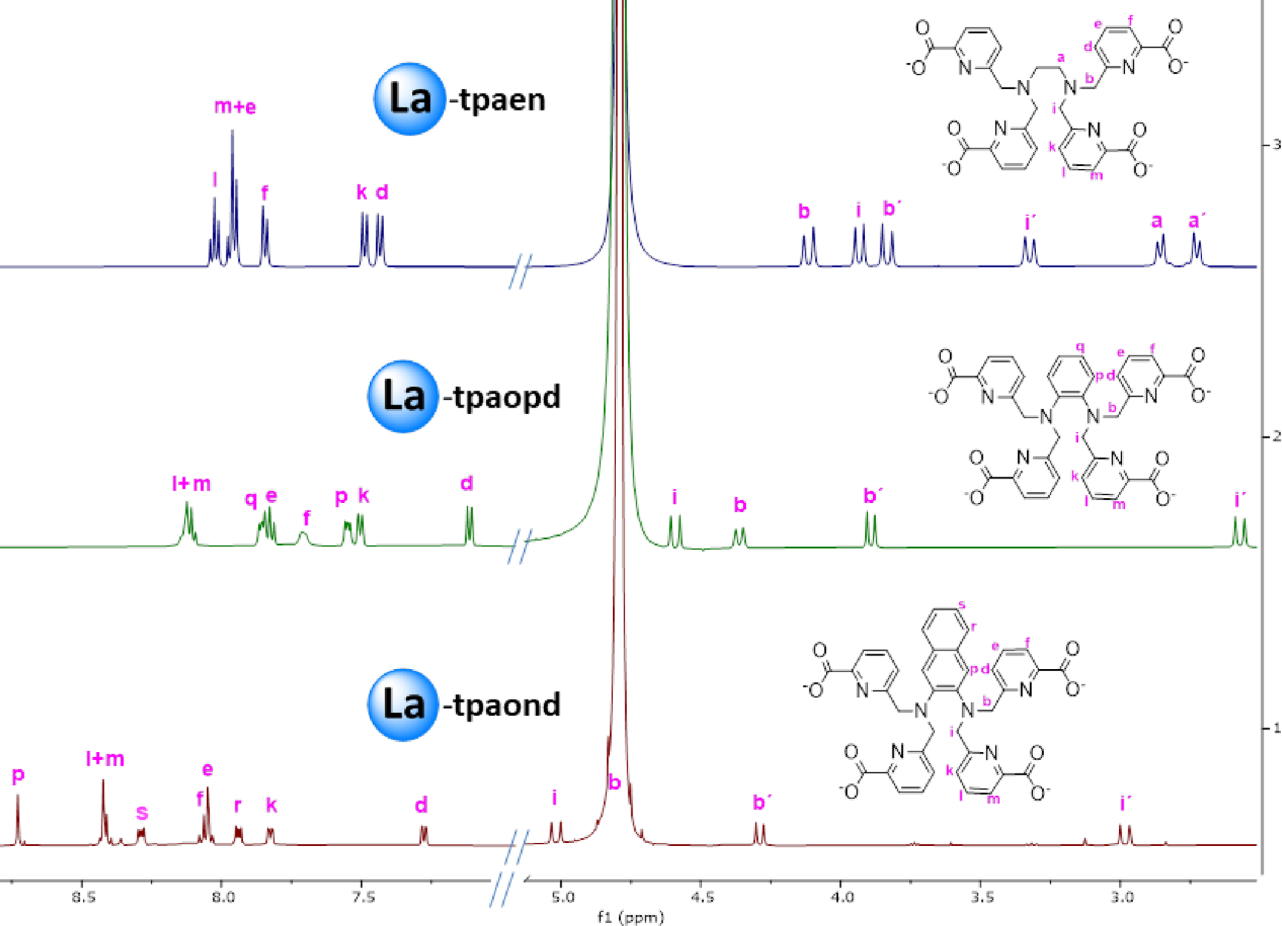
^1^H NMR spectra (500 MHz, 298 K) of the complexes [La­(**tpaen**)]^−^ (D_2_O pD = 6), [La­(**tpaopd**)]^−^ (D_2_O pD = 6), and [La­(**tpaond**)]^−^ (D_2_O:CD_3_CN 7:3 pD = 6).

From the solutions prepared for the NMR studies, single crystals of formula {[La­(**tpaen**)]­Na­(H_2_O)_4_}_2_·6H_2_O, {[La­(**tpaopd**)]­Na­(H_2_O)_4_}, and {[La­(**tpaond**)]­Na·13.75H_2_O} were grown. The X-ray crystallographic analyses confirmed the presence of the chiral helical entities [La­(L)]^−^ observed in solution by NMR, with the picolinate arms of the chelators wrapping around the La^3+^ ion in a pseudo-*C*_2_-symmetry. Figure 3 shows these helical structures, while bond lengths of the coordination spheres are compiled in Table 3. In all three cases, the chelating agents are completely deprotonated and the La^3+^ ion is ten-coordinate while bound to the deprotonated oxygen atom from each of the four carboxylate groups (O_COO_), to the four nitrogen atoms of the pyridines (N_pyr_), and to both nitrogen atoms of the tertiary amines from the spacer (N_am_). The metal ion is perfectly encapsulated within the cavity provided by the respective chelator and there are no water molecules within the coordination spheres. This helical arrangement found for the La^3+^ complexes in the solid state is similar to that reported for Eu-**tpaen** and Ce-**tpaen**,^29^ and two possible helical arrangements are available (Δ and Λ).^46^ The three La^3+^ complexes crystallize as racemic mixtures of both enantiomers in centrosymmetric space groups and in all, the sodium cation interacts not only with water molecules, but also with noncoordinated oxygen atoms of some carboxylate groups of the chelators, resulting in monomeric or dimeric structures similar to those found for Eu^3+^ and Ce^3+^ complexes with **tpaen.**

**Figure 3:**
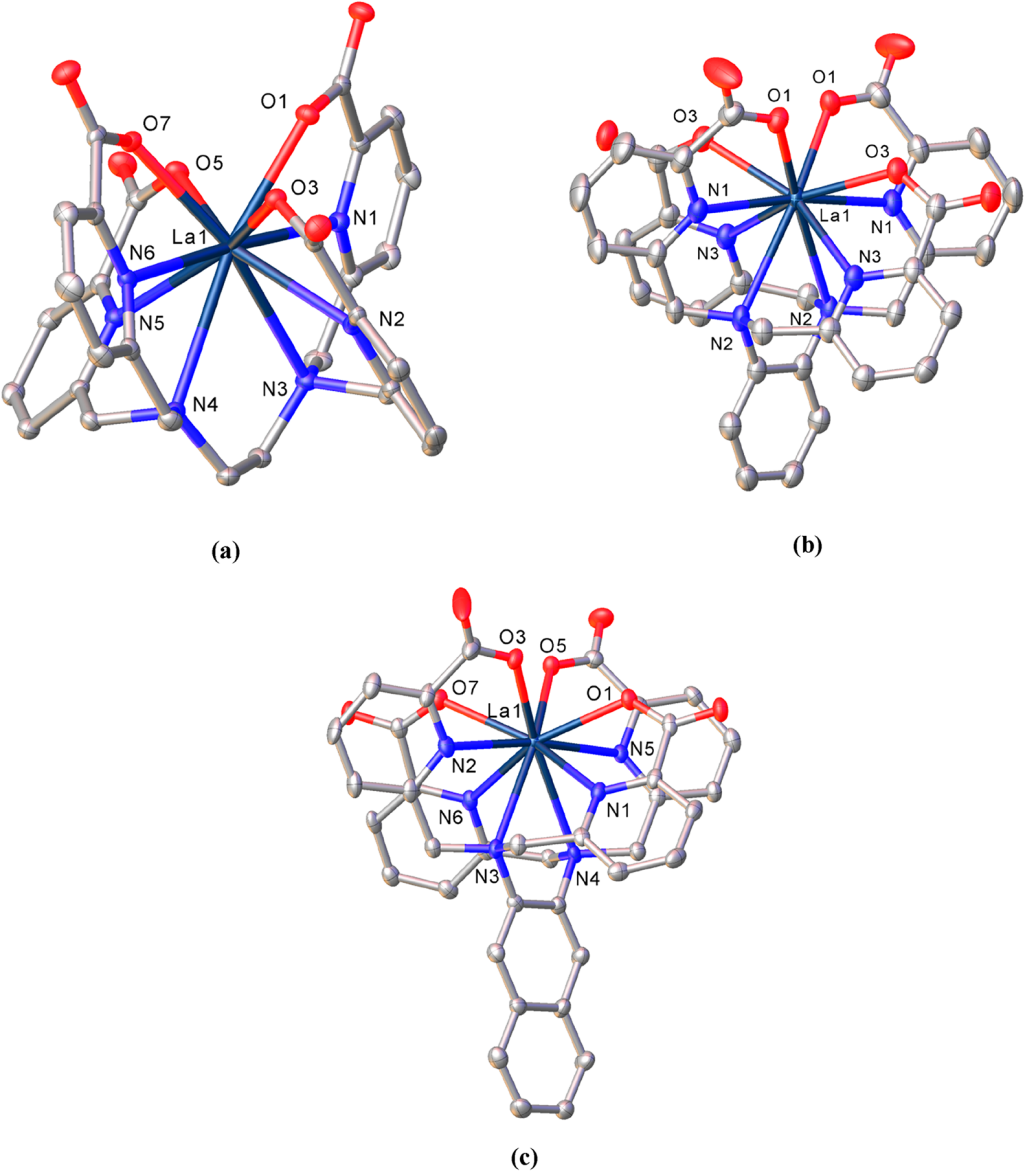
Solid-state X-ray structures of (a) [La­(**tpaen**)]^−^, (b) [La­(**tpaopd**)]^−^, and (c) [La­(**tpaond**)]^−^. Thermal ellipsoids are drawn at 50% probability. Only heteroatoms of the coordination sphere are labeled; hydrogen atoms are omitted for clarity.

**Table 3:** Selected Bond Lengths (Å) in [La­(**tpaen**)]^−^, [La­(**tpaopd**)]^−^, and [La­(**tpaond**)]^−^

	[La(**tpaen**)]^−^	[La(**tpaopd**)]^−^	[La(**tpaond**)]^−^
La–O_COO_	2.515(1)	2.456(2)	2.538(3)
La–O_COO_	2.572(1)	2.485(2)	2.464(3)
La–O_COO_	2.482(1)	2.456(2)	2.495(3)
La–O_COO_	2.514(1)	2.485(2)	2.576(3)
La–N_pyr_	2.733(2)	2.770(2)	2.731(4)
La–N_pyr_	2.713(2)	2.725(3)	2.716(4)
La–N_pyr_	2.722(2)	2.770(2)	2.737(4)
La–N_pyr_	2.739(2)	2.725(3)	2.702(4)
La–N_am_	2.892(2)	3.008(3)	2.980(4)
La–N_am_	2.940(2)	3.008(3)	2.969(4)

The La–O_COO_ and La–N_pyr_ distances are within the range of values reported in the literature for ten-coordinate La^3+^ complexes with ligands containing deprotonated pendent picolinate groups^43,47–52^ as well as those derived from the decadentate macrocyclic receptor **macropa** and others related,^21,53,54^ in which this metal ion achieves a coordination number of 11 through the additional coordination of a water molecule. When compared with theoretical values, the La–O_COO_ distances found in the three helical structures discussed here ([La­(**tpaen**)]^−^, [La­(**tpaopd**)]^−^, and [La­(**tpaond**)]^−^) are generally shorter than the theoretical value (2.542 Å) calculated as CRLa + rD [CRLa = 1.41 Å (CN = 10);^55^ rD taken from values reported for rare earth complexes^56^]. This is particularly noticeable in the complex with the *o*-phenylene spacer ([La­(**tpaopd**)]^−^) where La–O_COO_ distances are 2.456(2) and 2.485(2) Å. Although closer to the theoretical value (2.739 Å), the lengths found for La–N_pyr_ are also somewhat shorter in our structures, except for one of the distances found in [La­(**tpaopd**)]^−^, which exceeds it by 0.031 Å. The La–N_am_ distances are close to 3.0 Å for the complexes with rigid spacers ([La­(**tpaopd**)]^−^ and [La­(**tpaond**)]^−^) and somewhat smaller (close to 2.9 Å) for [La­(**tpaen**)]^−^. Values close to 3.0 Å have been reported for the La^3+^ complex with the related acyclic tetra-arm chelator **py4pa** (3.0519, 3.0926 Å - DFT calculations), which, as we have already mentioned, has proven to be a highly promising chelator for actinium.^23^

Such helical structures were also found in X-ray quality single-crystals of terbium grown from samples prepared in situ by mixing equimolar amounts of the corresponding chelator hydrochloride salt and TbCl_3_·6H_2_O in water/CH_3_CN 60:40 (v/v) at pH = 6 (adjusted by the addition of KOH). The complexes are also racemic in the solid state, with Δ and Λ isomers present in the unit cell. As in lanthanum crystals, the alkali cation present (in this case, K^+^) is bound to uncoordinated oxygen atoms of some carboxylate groups, as well as to water molecules. The structures of the chiral helical complexes ([Tb­(**tpaopd**)]^−^, [Tb­(**tpaond**)]^−^) are shown in Figure 4, while the bond distances of the coordination spheres are provided in Table 4.

**Figure 4:**
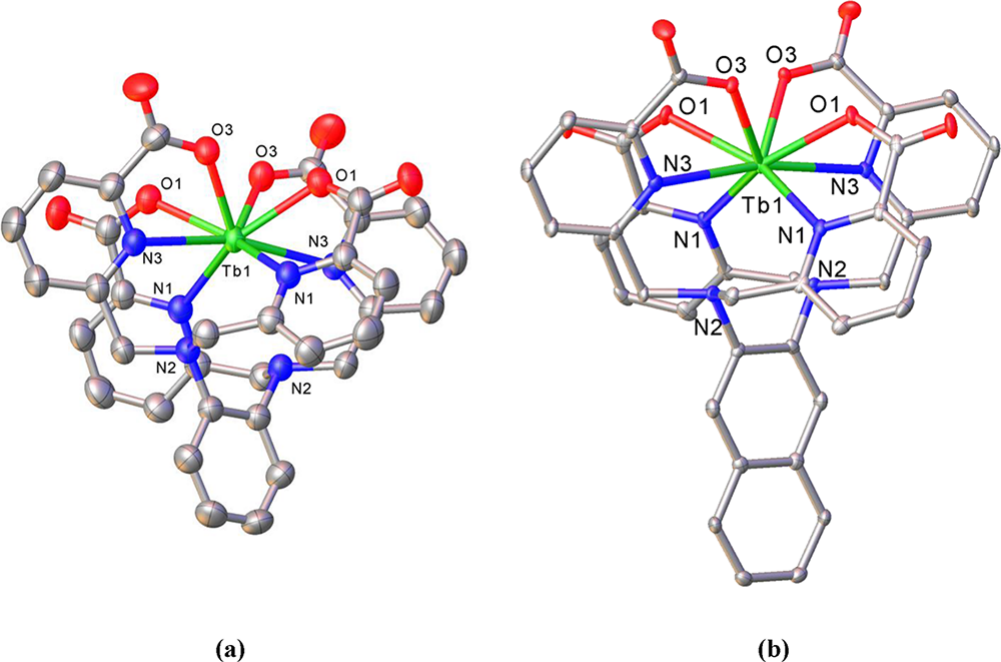
Solid-state X-ray structures of (a) [Tb­(**tpaopd**)]^−^ and (b) [Tb­(**tpaond**)]^−^. Thermal ellipsoids are drawn at 50% probability (crystal structure of [Tb­(**tpaopd**)]^−^ was measured at 293 K). Only heteroatoms of the coordination sphere as well as tertiary nitrogen atoms are labeled; hydrogen atoms are omitted for clarity.

**Table 4:** Selected Bond Lengths (Å) in [Tb­(**tpaopd**)]^−^ and [Tb­(**tpaond**)]^−^ ^a^

	[Tb(**tpaopd**)]^−^	[Tb(**tpaond**)]^−^
Ln–O1_COO_	2.418(2)	2.426(2)
Ln–O3_COO_	2.344(2)	2.356(2)
Ln–N1_pyr_	2.595(3)	2.605(3)
Ln–N3_pyr_	2.658(3)	2.666(3)

a Symmetry transformations used to generate equivalent atoms: #1 −*x* + 1,*y*,–*z* + 3/2.

As with the La^3+^ compounds described above, the four arms are also twisted in the Tb^3+^ complexes, effectively enveloping the metal ion and preventing water molecules from approach. However, unlike what is observed in La^3+^ complexes, which are ten-coordinate, the small Tb^3+^ ion satisfies its coordination requirement by binding to only eight of the ten donors offered by the chelating agents. Thus, in these terbium complexes, the metal ions are directly bound only to the four anionic oxygen atoms offered by the carboxylate groups and to the four nitrogen atoms from the pyridines; SHAPE analyses indicate that the coordination polyhedron about the Tb^3+^ most closely matches a triangular dodecahedron.^57^ Both N_am_ atoms of the spacer are too far from the metal center to ensure that a proper bond exists (Tb···N_am_ 3.101 Å in [Tb­(**tpaopd**)]^−^; Tb···N_am_ 3.019 Å in [Tb­(**tpaond**)]^−^). Although they are not considered to be in the coordination sphere, these amine nitrogen atoms are essential for enabling the picolinate arms to envelop effectively the metal cation, and, albeit indirectly, they also provide electron density to the metallic environment, which helps to stabilize the corresponding complex. The number of X-ray crystal structures reported Tb^3+^ complexes with ligands containing pendent picolinate groups is quite scarce, which makes it difficult to carry out comparative studies of the bond distances of coordination spheres. Moreover, most of the structures display nine-coordinated Tb^3+^ ions,^48,58–65^ and eight-coordination for this metal ion has only been found in the X-ray crystal structure of a polymeric entity based on the related four-arm decadentate chelator H_4_ **tpabn**, which contains an *n-*butylene spacer.^66^ Although when comparing the coordination-sphere bond distances, it is correct to do so for the same metal coordination environments, based on the analysis of the X-ray crystal structures reported, we have verified that the Tb–O_COO_ distances for nine- and eight-coordination are in the same range. In both helicates ([Tb­(**tpaopd**)]^−^ and [Tb­(**tpaond**)]^−^) two quite different Tb–O_COO_ distances are found. The shortest one (ca. 2.34–2.35 Å), which is within the range of those published, is associated with the arm that experiences the least torsion. In line with this, the longest one (ca. 2.42 Å, longer than those found in the literature) is found in the most twisted arm. With very few exceptions, the Tb–N_pyr_ distances of our helicates are in general longer than those found in the literature and, again, one is longer than the other (ca. 2.66 vs 2.60 Å). The longest Tb–N_pyr_ distance is linked to the shortest Tb–O_COO_ distance. Therefore, the more twisted the arm is, the closer N_pyr_ gets, forcing O_COO_ to move away, and vice versa. Consequently, it can be stated that the ultimate reason for these twists appears to be to achieve optimal (maximum) interactions between the metal and all the donor atoms.

Figure 5 displays the ^1^H NMR spectra of the Lu^3+^ complexes with chelators H_4_ **tpaen**, H_4_ **tpaopd**, and H_4_ **tpaond**. Spectra were recorded from samples prepared in situ by mixing equimolar amounts of each ligand with LuCl_3_ in D_2_O (Lu-**tpaen** and Lu-**tpaopd**) or D_2_O/CD_3_CN 70:30 (v/v) (Lu-**tpaond**) adjusted to pD = 6 by addition of KOH. The ^1^H NMR spectrum of the complex containing **tpaen** shows very broad signals indicative of fluxionality. Recording the spectrum at different temperatures did not yield satisfactory results, which prevented us from identifying the species present and reasons for this behavior. On the contrary, the ^1^H NMR spectra for the Lu^3+^ complexes with the chelators containing the rigid aromatic spacers (**tpaopd**, **tpaond**) exhibit sharp and well-defined signals with no observable fluxional isomerism in solution. Detailed analysis of the proton spectra reveals the presence of a single asymmetric [Lu­(L)]^−^ complex in solution, as indicated by 12 inequivalent one-proton signals in the aromatic region, which are composed of four sets of mutually coupled protons, corresponding to four chemically distinct picolinate units bound to the metal center. *C*_1_ symmetry of the species [Lu­(**tpaopd**)]^−^ and [Lu­(**tpaond**)]^−^ is also supported by the ^13^C­{^1^H} NMR spectra (Figures S73 and S79), which show 33 and 37 resonances, respectively; in both cases, there are two carbons that match in their chemical shifts. Assignments of proton and carbon spectra were achieved with the aid of 2D experiments (Figures S71–S82, and Tables S6 and S7).

**Figure 5:**
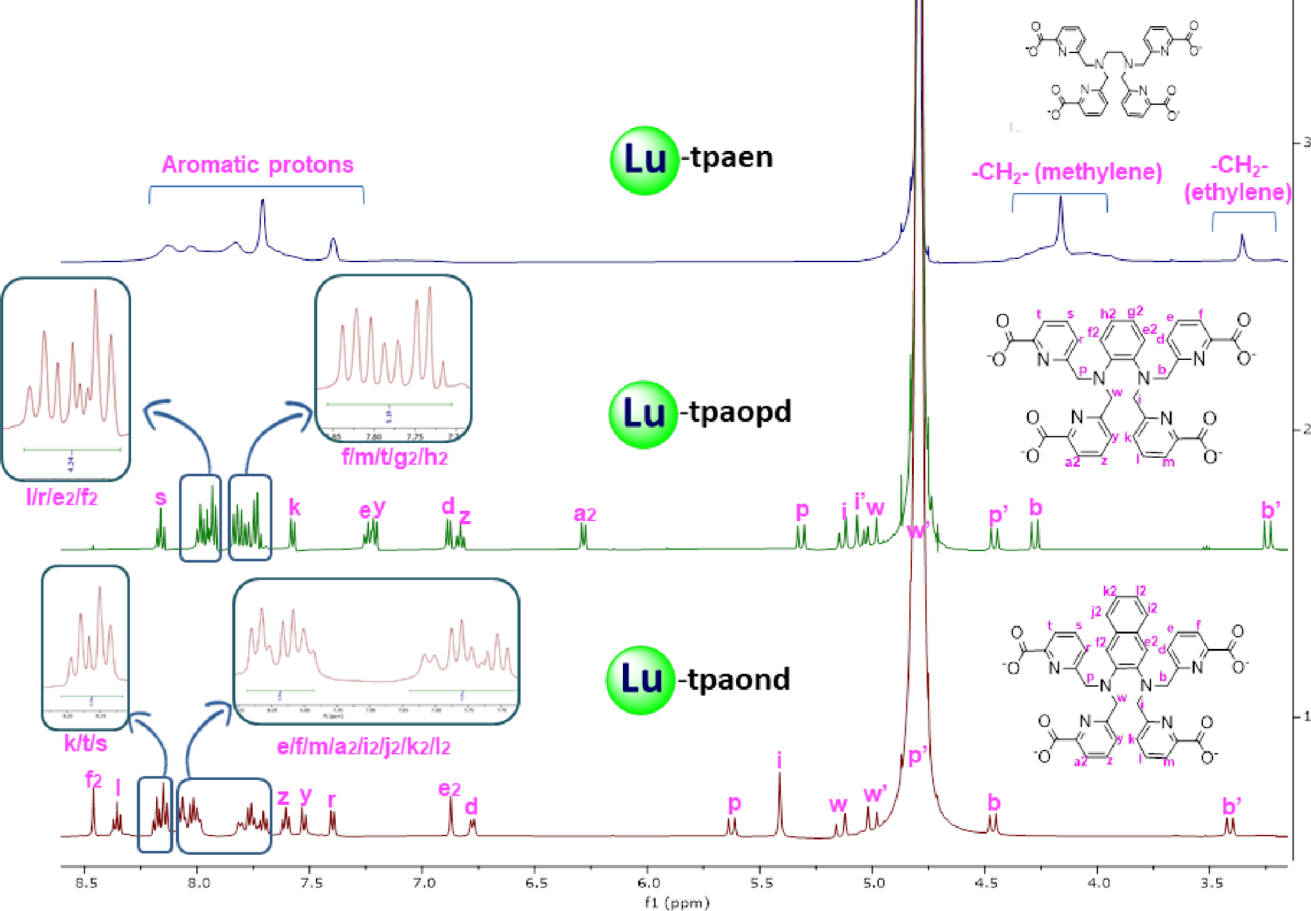
^1^H NMR spectra (500 MHz, 298 K) of the complexes Lu-**tpaen** (D_2_O pD = 6), [Lu­(**tpaopd**)]^−^ (D_2_O pD = 6), and [Lu­(**tpaond**)]^−^ (D_2_O:CD_3_CN 7:3, pD = 6). (For better resolution, see Figures S70, S71, and S77).

The ^1^H NMR spectrum of [Lu­(**tpaopd**)]^−^ clearly shows the eight nonequivalent one-hydrogen doublets due to the four pairs of diastereotopic methylene protons (Hp/Hp’, Hi/Hi’, Hw/Hw’, Hb/Hb’) resulting from the coordination of the four picolinate residues, with characteristic large coupling constants and different chemical shifts (see Table 6), indicating two different chemical environments. In the ^1^H NMR spectrum of [Lu­(**tpaond**)]^−^ only three pairs of diastereotopic methylene protons are found: Hp/Hp’, Hw/Hw’ and Hb/Hb’; the protons of one methylene group (Hi) are not split, appearing as a singlet signal at 5.41 ppm. The large difference in chemical shifts exhibited by Hp/Hp’ and Hb/Hb’ in both complexes [Δδ_p/p’_ ∼ 0.85 ppm, Δδ_b/b’_ ∼ 1.05] indicates that two of the four picolinate arms are bound in a more anisotropic local environment in the complex, as discussed above for the La^3+^ complexes.

Single crystals of the Lu^3+^ complex with **tpaopd** were obtained from the solution used for NMR studies after acetonitrile was added and the solution was allowed to evaporate slowly, and their X-ray diffraction analysis confirmed the presence of the expected lutetium helical complex. The refinement of this crystal structure was particularly arduous; although, in general, the refinement of all the crystal structures of the helical complexes analyzed in this article has proved very difficult, the refinement of this lutetium structure was particularly challenging. All the crystals exhibit particularly complicated disorder due to the presence of numerous disordered water molecules, some of them close to special positions, others coordinated with the potassium (or sodium) ion, and many of them also involved in an extensive and changing network of hydrogen bonds. In most of the cases, the disorder was modeled just with soft restraints and/or fixing occupation factors and, in some cases, the position of any hydrogen atom. However, in the case of the lutetium crystal structure, to reach a satisfactory convergence it was necessary to perform the squeeze procedure implemented in PLATON, as described in the Experimental Section, to mask not only the solvent electron density but also the potassium ion present in the crystal lattice. Figure 6 illustrates the structure of the helical [Lu­(**tpaopd**)]^−^ complex, which is similar to the structure found for the Tb^3+^ analogue, with the four arms wrapping around the sequestered metal ion. As in the terbium complex, the lutetium ion is also eight-coordinated, being directly bound only to the four anionic oxygen atoms of the carboxylate groups and to the four nitrogen atoms from the pyridines; the coordination polyhedron matches a triangular dodecahedron, too. Tertiary nitrogen atoms are quite far from the metal ion (Lu···N_am_ 3.157 Å). The metal-donor distances of the coordination sphere in [Lu­(**tpaopd**)]^−^ (see Figure 6) are slightly shorter than those found in [Tb­(**tpaopd**)]^−^ as expected given the smaller size of Lu^3+^. The number of X-ray crystal structures described for Lu^3+^ complexes with ligands containing pendant picolinate groups is also quite limited, again making it difficult to conduct comparative studies of the bond distances of the coordination spheres. In any case it can be seen that the Lu–O_COO_ distances in [Lu­(**tpaopd**)]^−^ are within the range of the values described in the literature for eight-coordinate Lu^3+^ complexes^48,50,51,67–70^ while the Lu–N_pyr_ distances are ca. 0.1 to 0.2 Å longer. As found in the Tb^3+^ helicates, two quite different Lu–O_COO_ and Lu–N_pyr_ distances exist, and similarly, the longest Lu–N_pyr_ distance is linked to the shortest Lu–O_COO_ one.

**Figure 6:**
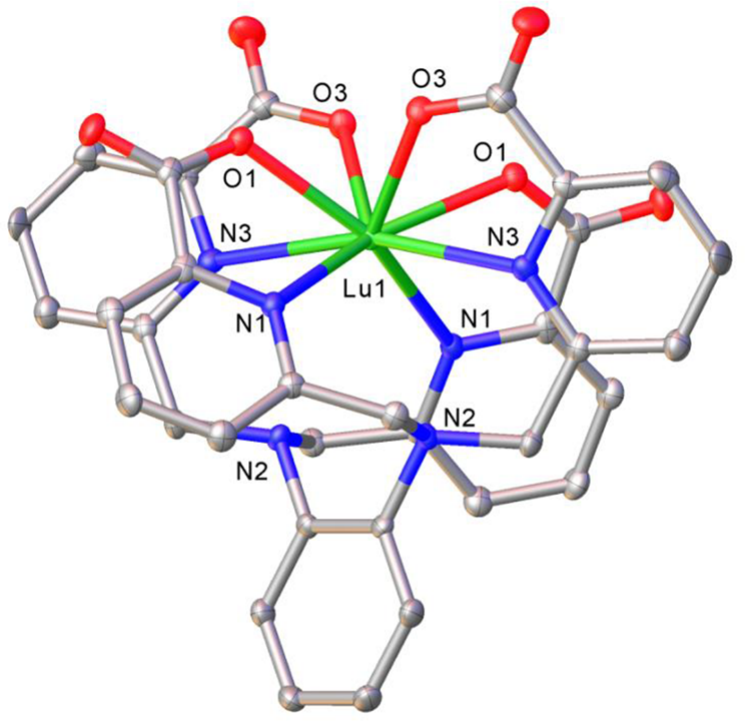
Solid-state X-ray structure of [Lu­(**tpaopd**)]^−^. Thermal ellipsoids are drawn at 50% probability. Only heteroatoms of the coordination sphere as well as tertiary nitrogen atoms are labeled; hydrogen atoms are omitted for clarity. Distances of the coordination sphere (Å): Lu–O1 2.274(3); Lu–O2 2.364(3); Lu–N1 2.633(3); and Lu–N3 2.560(3).

With an optimal molecular architecture, when fully deprotonated, these three acyclic four-arm chelating agents (**tpaen**, **tpaopd**, and **tpaond**) can create cavities suitable for accommodating lanthanide ions of different sizes and effectively encapsulating them. Their structural adaptability to meet the steric requirements of these lanthanide cations can be rationalized based on N_am_···N_am_ distance, N_py_···N_py_. distances of the nitrogen atoms of the pyridine rings in opposite positions in the coordination sphere, as well as the bite angle N_am_–Ln–N_am_ involving the central chelate of the helix together with the dihedral angles N_am_–C–C–N_pyr_ and N_am_–C–C–N_am_. The values of these parameters for the six complexes are shown in Table 5, together with those for the analogous [Ce­(**tpaen**)]^−^ and [Eu­(**tpaen**)]^−^ for comparison.^29^ The N_am_···N_am_ and N_py_···N_py_ distances confirm that the cavity expands or contracts depending on the size of the ion that chelator hosts, whereas the dihedral angles N_am_–C–C–N_pyr_ give an idea of the greater or lesser tension to which the pendant arms are subjected when they twist to allow the N_pyr_ atoms to approach the metal ion. From the comparison of the angle values for each pair of arms linked to the same N_am_, it follows that in structures containing aromatic spacers [*o*-phenylene (**tpaopd**), 2,3-naphthylene (**tpaond**)], one of the arms is highly twisted with respect to the other. Although this greater twist of one arm compared to the other is also observed in the structure of [La­(**tpaen**)]^−^, it is not as pronounced in this case. The greater or lesser torsion of the pendant arms seems to be also related with the dihedral angle N_am_–C–C–N_am_. The relatively flexible ethylene spacer of **tpaen** twists in order to allow the pyridine nitrogen atoms to access the metal coordination environment more easily. This leads to increased torsion of the ethylene bridge and reduced torsion of the pendant arms. However, the rigid *o*-phenylene and 2,3-naphthylene groups can hardly twist (N_am_–C–C–N_am_ dihedral angles are ca. 5 to 10° in complexes of **tpaopd**, and ca. 15.5° for **tpaond** derivatives), resulting in one of the two mutual pendant arms being forced to twist extensively (ca. 60°). The forced twisting of the pendant arms aims to achieve the ideal frontal overlap between the metal ion and the N_pyr_, which donates its electron pair through the *sp* ^2^ orbital. Depending on the size of the metal ion and the greater or lesser flexibility of the spacer, the arms reach a torsional compromise to achieve this overlap in the most effective way for the four N_pyr_. Table 6 relates the torsion angles of the arms with the corresponding angle Ln-N_pyr_-C_pyr(*para*)_. It can be seen that the greater the torsion angle, the greater the angle Ln-N_pyr_-C_pyr(*para*)_ (this value should be 180° for the ideal frontal overlap). In these picolinate-based helical structures, negative dihedral angles N_am_–C–C–N_am_ (and positive N_am_–C–C–N_pyr_) indicate a Δ configuration, while positive angles N_am_–C–C–N_am_ (and negative N_am_–C–C–N_pyr_) are present in the Λ configuration.^71^

**Table 5:** Selection of Distances (Å) and Angles (°) Found in the X-ray Crystal Structures of the Ln^3+^ Complexes with **tpaen**, **tpaopd**, and **tpaond**

	N_am_···N_am_	N_pyr_···N_pyr_	N_am_-Ln-N_am_	dihedral angles (N_am_–C–C–N_pyr_)	dihedral angle (N_am_–C–C–N_am_)
[La(**tpaen**)]^−^	3.002	5.469; 4.831	61.95	–20.13; −23.70; −42.10; −47.78	58.87
[Ce(**tpaen**)]^−^ ^a^	3.011	5.455; 4.813	62.29	–20.59; −24.11; −41.05; −45.61	58.30
[Eu(**tpaen**)]^−^ ^a^	2.978	5.375; 4.718	61.55	24.05; 24.43; 27.94; 33.66	–66.37
[La(**tpaopd**)]^−^	2.958	5.438; 4.850	58.90	19.57; 57.22	–10.50
[Tb(**tpaopd**)]^−^	2.873	5.273; 4.585	55.19	14.88; 60.57	–4.84
[Lu(**tpaopd**)]^−^	2.857	5.208; 4.468	53.82	–15.34; −61.86	5.06
[La(**tpaond**)]^−^	2.932	5.439; 4.789	59.06	14.00; 16.00; 55.77; 58.20	–13.55
[Tb(**tpaopd**)]^−^	2.889	5.310; 4.555	57.17	13.85; 57.74	–13.84

a Data from ref 29.

**Table 6:** Relationship between the Dihedral Angle N_am_–C–C–N_pyr_ and the Angle Ln-N_pyr_–C_pyr(*para*)_ (°) for Each Pendant Arm in the Ln^3+^ Complexes with **tpaen**, **tpaopd**, and **tpaond**

	dihedral angle (N_am_–C–C–N_pyr_)	angle (Ln-N_pyr_–C_pyr(*para*)_)
[La(**tpaen**)]^−^	–20.13	158.95
	–23.70	162.51
	–42.10	172.71
	–47.78	161.32
[La(**tpaopd**)]^−^	19.57	156.64
	57.22	164.38
[La(**tpaond**)]^−^	14.00	153.33
	16.00	154.72
	55.77	165.31
	58.20	162.58
[Tb(**tpaopd**)]^−^	14.88	154.91
	60.57	160.83
[Tb(**tpaond**)]^−^	13.85	154.09
	57.74	163.37
[Lu(**tpaopd**)]^−^	–15.34	154.23
	–61.86	160.93

In order to assess the decisive effect of the presence of the nitrogen pyridine in the long linker present in the promising chelator for actinium-225 H_4_ **py4pa**, we decided to include the chelator H_4_ **tpamxd** to our study. This chelator is similar to H_4_ **py4pa** in terms of its molecular architecture, but the pyridine ring has been replaced by a benzene ring in the spacer. We have found that, unlike H_4_ **py4pa**, which very effectively encapsulates the La^3+^ ion in a mononuclear helical structure similar to those found in [La­(**tpaen**)]^−^, [La­(**tpaopd**)]^−^, and [La­(**tpaond**)]^−^ described above, the ligand H_4_ **tpamxd** forms a coordination polymer with the lanthanide ions.

The 1:1 reaction of lanthanide­(III) chlorides (La^3+^, Tb^3+^, Lu^3+^) with H_4_ **tpamxd** in water leads to the formation of a white solid which tends to remain in suspension and settles over time. ^1^H NMR spectroscopy of the supernatant solution confirmed the polymeric nature of the species formed. Both the La^3+^ spectrum (Figure S62) and the Lu^3+^ spectrum (Figure S83) confirm the coordination of the four picolinate groups, which are now equivalent, rather than being in pairs as in mononuclear helical structures. The coordination of the picolinate groups causes the diastereotopic splitting of the methylene protons adjacent to the picolinate [δ (ppm): 3.77 (d, *J* = 15.0 Hz) and 4.14 (d, *J* = 15.0 Hz) in the La^3+^ compound; 3.89 (d, *J* = 16.0 Hz) and 4.39 (d, *J* = 16.0 Hz) in the Lu^3+^ compound], but the CH_2_ protons of the *m*-xylylene spacer continues appearing as a singlet (4.14 and 4.17 ppm, respectively). The formation of a polymer instead of the helical mononuclear complex also occurs with the regioisomeric chelator H_4_ **tpapxd**, with a similar ^1^H NMR spectroscopy pattern being observed (Figures S86–S91). Moreover, the existence of polymers with these ligands containing xylylene spacers has also been confirmed by X-ray diffraction. Single crystals were obtained by slow evaporation of a solution of equimolar amounts of the hydrochloride salt of H_4_ **tpapxd** and TbCl_3_·6H_2_O in water at pH 4.5 (adjusted with NaOAc buffer). The X-ray diffraction study revealed the presence of a terbium coordination polymer with the formula {[Tb­(H_2_ **tpapxd**)­(H_2_O)]­Cl·4H_2_O}_∞_, whose structure is shown in Figure 7. It can be observed that an amine nitrogen atom and a carboxylate group located in a pendant arm attached to such nitrogen are protonated. The terbium ion is nine-coordinate; bond lengths of the coordination sphere are indicated in the legend of Figure 7. Heteroatoms of three neighboring H_2_ **tpapxd** chelators form part of the coordination sphere of each terbium ion. One of the ligands contributes with one amine nitrogen as well as the heteroatoms of the corresponding picolinate groups of the pendant arms hooked on it (two N_pyr_ and two O_COO_). The second ligand participates with the two oxygen atoms of the deprotonated carboxylate group anchored to the protonated N_am_ acting in a terminal bidentate mode. Finally, the third chelator provides an oxygen atom from a bidentate carboxylate group that acts as a bridge between two adjacent metal centers. The oxygen atom of a water molecule completes the coordination sphere. It should be noted that, unlike the free chelator, in which both amine groups are in an *anti* conformation with respect to the benzene ring (see Figure 1), the conformation adopted is *syn* in this structure. Although this conformation is required to promote the encapsulation of the metal ion and form mononuclear complexes, the structural characteristics of the spacer (high rigidity and length) seem to be responsible for this not being achieved. The distance N_am_···N_am_ (7.131 Å) continues to be very large and ultimately, the system is forced to polymerize to satisfy the coordination preferences of terbium, which include high CN (8 or 9). Hydrogen bonding interactions involving lattice water molecules, chloride anions, the protonated amine groups and some picolinate groups help to stabilize the crystal.

**Figure 7:**
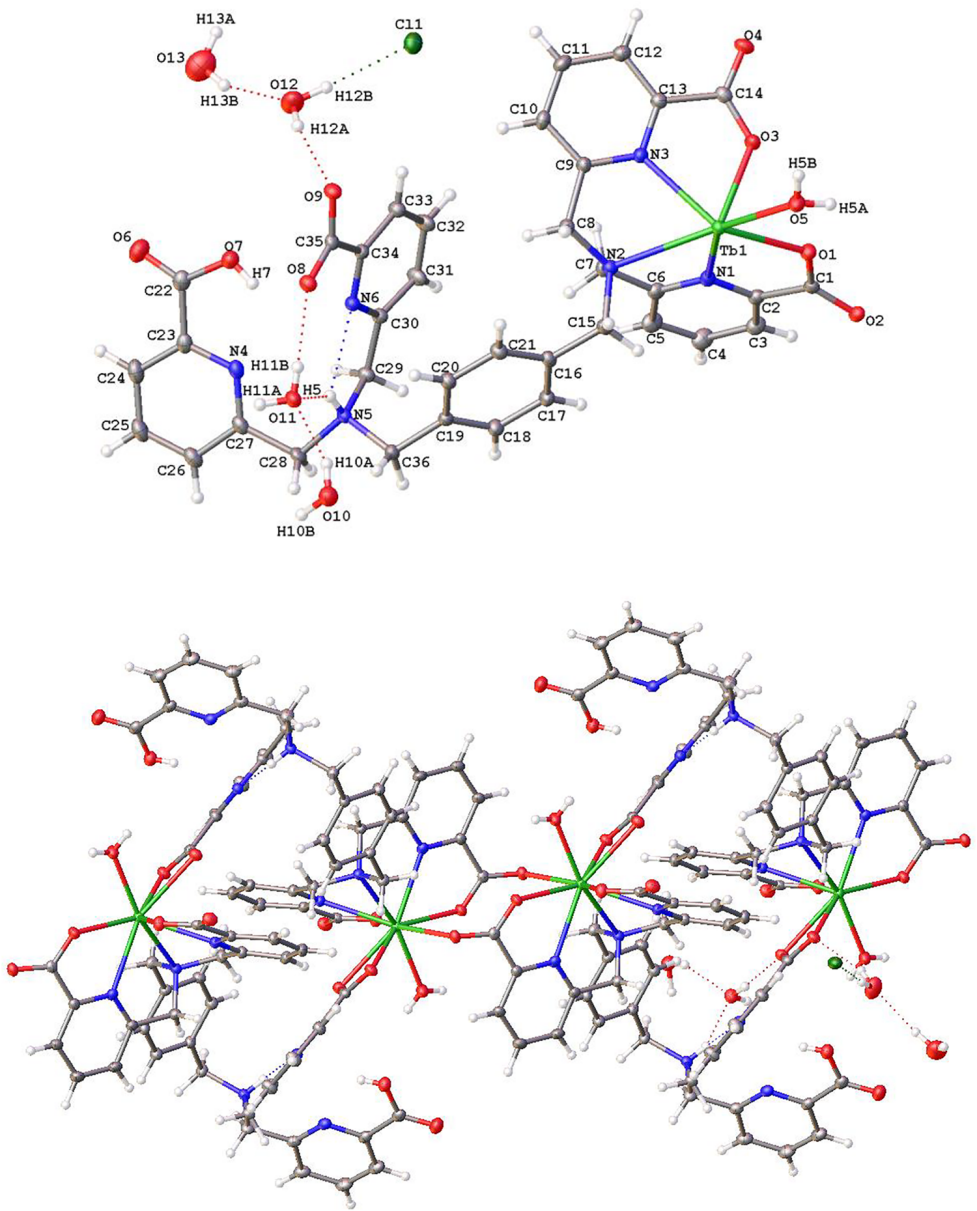
Solid-state X-ray structure of {[Tb­(H_2_ **tpapxd**)­(H_2_O)]­Cl·4H_2_O}_∞_. Ellipsoids are drawn at 50% probability. Distances of the coordination sphere (Å): Tb–O1 2.4201(18); Tb–O2^1^ 2.3922(18); Tb–O3 2.3247(19); Tb–O5 2.367(2); Tb–O8^2^ 2.6223(19); Tb–O9^2^ 2.4232(19); Tb–N1 2.555(2); Tb–N2 2.739(2); and Tb–N3 2.525(2).

The absence of a central heteroatom, which (as in **py4pa**) has a directing effect on the coordination of the metal ion, appears to be the key factor determining the formation of polymeric species with these ligands derived from the long xylene-bridges. Similar coordination polymers of lanthanide ions have also been found with the related chelator H_4_ **tpabn** containing the long, flexible spacer *n*-butylene.^65,66^

With a shorter spacer (propylene), the chelator H_4_ **tpadapo** has a heteroatom (in this case O) in the connecting bridge, now incorporated as hydroxyl group linked to the central carbon. When this chelator is mixed with lanthanum chloride in water (1:1 molar ratio, pH 6 adjusted with NaOH), a very fine white precipitate also appears to form, although nowhere near the amount that occurs with the H_4_ **tpamxd** and H_4_ **tpapxd** chelators. The result is a cloudy solution that greatly hinders characterization by NMR. The ^1^H NMR spectrum is very messy and no clear conclusions can be drawn. Although no turbidity was observed in solution for Lu^3+^ compound, its ^1^H NMR spectrum is also not well resolved and does not shed light on the species that may have formed. Confirmation of metal complexation was possible through HR-MS; peaks corresponding to the species [Ln­(**tpadapo**)] at *m*/*z* 765.0831 (La) and 801.1179 (Lu) are observed in the HR-MS ESI^–^ spectra. In ESI^+^, peaks at *m*/*z* 811.0622 [M + 2Na]^+^, 789.0801 [M + H + Na]^+^ and 767.0981 [M + 2H]^+^ appear in the spectrum of the lanthanum derivative; with lutetium, the peak corresponding to [M + Na + H]^+^ appears at *m*/*z* 825.1149 (M = [Ln­(**tpadapo**)] (Figures S113 and S124).

The ability of the two asymmetrical potentially octadentate chelating agents (H_3_ **tripaen** and H_4_ **asyoctapa**) to form complexes with lanthanide ions was also evaluated. As mentioned, these chelators structurally derive from H_4_ **tpaen** through the loss of one arm (the first) and the replacement of two vicinal picolinate pendent arms by acetate groups (the second). The study of H_3_ **tripaen** with La^3+^ and Lu^3+^ was carried out by NMR spectroscopy on samples prepared in situ in D_2_O by mixing equimolar amounts of lanthanide­(III) chloride and the chelator and the pD was adjusted to 6 with NaOD. Figure 8 shows the ^1^H NMR spectra of the uncoordinated octadentate chelator and its metal complexes (see also Figures S63 and S92). Although in both spectra the signals of the methylene protons appear broadened and poorly defined, they are shown with better definition in the La^3+^ complex; in both complexes most of them are upfield shifted indicating metal complexation. Aromatic protons also experience a shielding effect and appear grouped into two sets of signals centered at 7.4 and 7.9 ppm. It has not been possible to make a complete assignment of the signals in the spectra of the complexes; however, no free ligand signals are observed in these spectra. HR-MS ESI, both in positive and negative mode, confirm the formation of mononuclear complexes. Peaks corresponding to species [M + Na]^+^ and/or [M + H]^+^ appear in the ESI^+^-MS while those corresponding to [M – H]^−^ are observed in ESI^–^ -MS (M = [Ln­(**tripaen**)], see Figures S114, S119 and S125).

**Figure 8:**
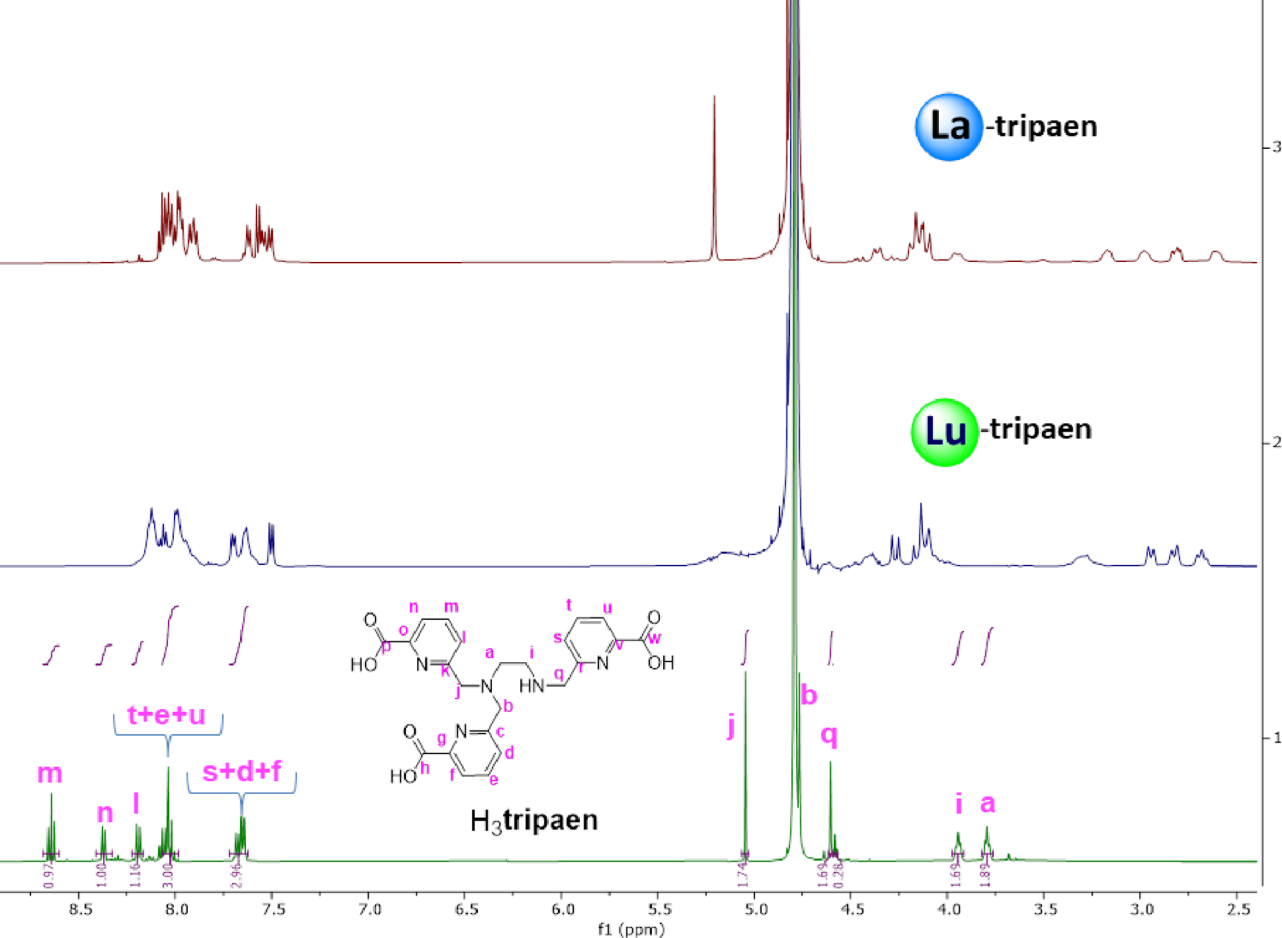
^1^H NMR spectra (500 MHz, 298 K, D_2_O) of La-**tripaen** (pD = 6), Lu-**tripaen** (pD = 6), and the ligand H_3_ **tripaen**.

H_4_ **asyoctapa** forms mononuclear complexes in solution with the three metal ions (La^3+^, Tb^3+^ and Lu^3+^) at pH = 6. ESI-MS in negative mode (Figures S115, S120 and S126) shows the expected peaks at *m*/*z* 581.0193, 601.0386, and 617.0554 corresponding to the [Ln­(**asyoctapa**)]^−^ species (La, Tb, Lu, respectively). Figure 9 displays the ^1^H NMR spectra of both lanthanum and lutetium complexes compared with that of the free chelator. The ^1^H NMR spectrum of each complex exhibits sharply resolved peaks, supporting the formation of a single, conformationally stable species in solution. As in the free ligand, the complexes’ two pendant arms containing the picolinate groups are equivalent to each other, as are the two acetate donors. In agreement with this observation, the ^13^C­{^1^H} NMR spectra of both complexes show 10 signals for the 20 carbons of the skeleton (Figures S66 and S95). Complexation causes the shielding of all hydrogens (both aliphatic and aromatic) and the diastereotopic splitting of the methylene-H atoms of the pendants (Hb and Hj). The CH_2_ protons of the ethylene spacer, which appear as closely spaced triplets in the free ligand (3.58 and 3.62 ppm), do not split in either complex. In the spectrum of the complexes, they continue to appear as triplets, although they are much further apart from each other [3.13 and 3.02 ppm; *J* = 4.9 Hz ([La­(**asyoctapa**)]^−^); 3.15 and 3.06 ppm, *J* = 3.15 Hz ([Lu­(**asyoctapa**)**]** ^**‑**^)]. In addition to the upfield shift experienced by the aromatic protons, complexation of the picolinate rings also causes changes in their patterns, now appearing as a triplet and two well-separated doublets.

**Figure 9:**
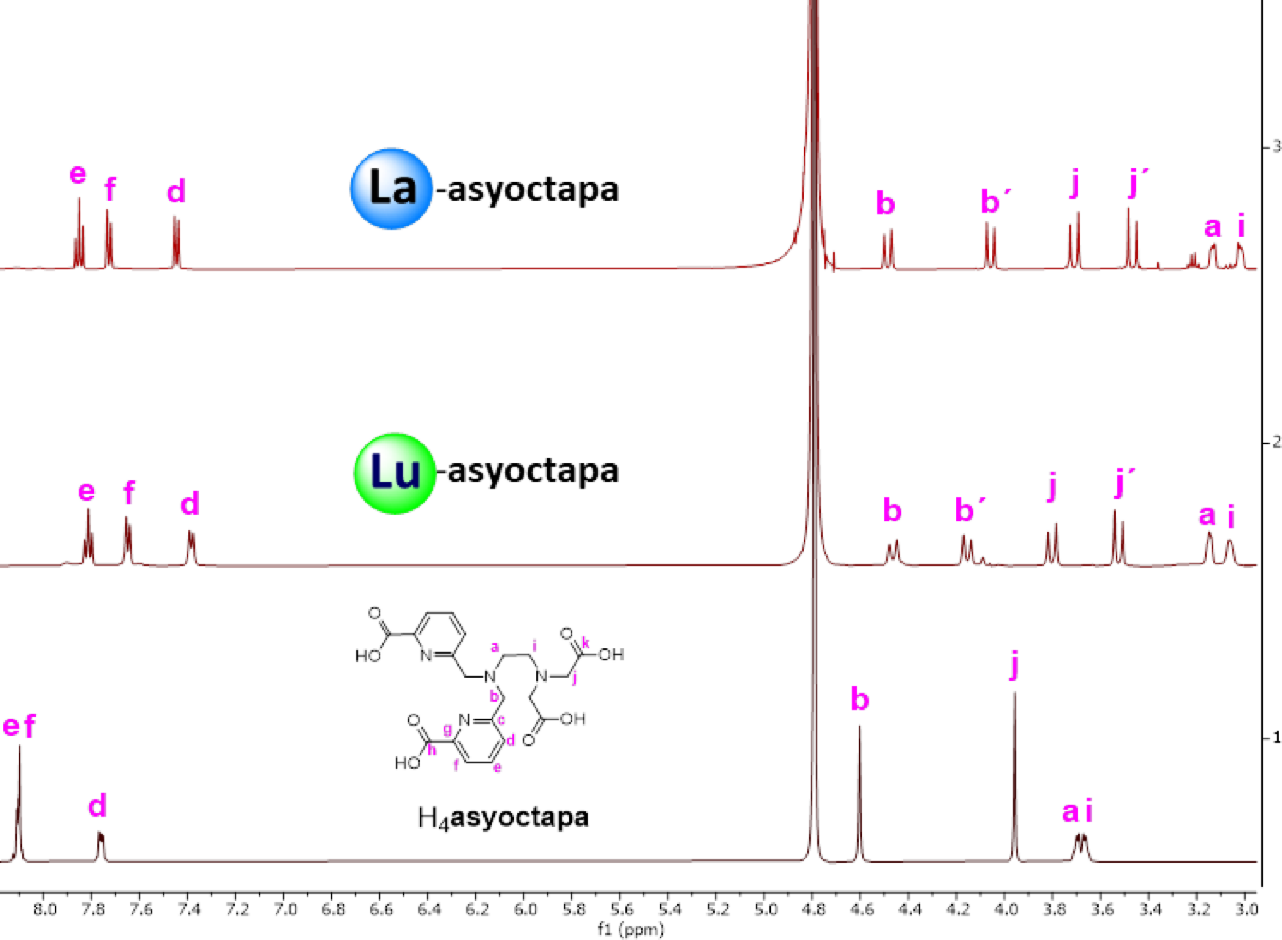
^1^H NMR spectra (500 MHz, 298 K, D_2_O) of [La­(**asyoctapa**)]^−^ (pD = 6), [Lu­(**asyoctapa**)]^−^, (pD = 6) and the ligand H_4_ **asyoctapa**.

### Radiolabeling Studies and Stability Challenges in Human Serum

Labeling experiments were performed under the conditions indicated in the Experimental Section. All radiolabeling experiments with the acyclic chelators described herein were carried out at RT and monitored over 30 min to mimic the mild conditions required for labeling of biological radiotracers.

Concentration-dependent radiolabeling studies were undertaken for ^225^Ac with each of the eight chelating ligands, and compared with positive controls [H_4_ **dota**, H_2_ **macropa**, and H_4_ **crown** (2,2’,2”,2”’-1,10-dioxa-4,7,13,16-tetraazacyclooctadecane-4,7,13,16-tetrayl-tetraacetic acid)]; see Figure 10 and Table 7. The ligands with the short ethylene-bridged (H_4_ **tpaen**) or vicinal-substituted phenyl backbones (H_4_ **tpaopd** and H_4_ **tpaond)** that we have verified to be capable of very efficiently encapsulating the nonradioactive La^3+^ ion, also demonstrated highly efficient radiolabeling with [^225^Ac]­Ac^3+^, achieving quantitative radiochemical yields (RCYs) at chelate concentrations as low as 10^–6^ M within 30 min at RT. Furthermore, H_4_ **tpaond** (derived from 2,3-naphthalene) was found to be the most effective new chelating agent for [^225^Ac]­Ac^3+^, achieving RCYs of 96 ± 4% at 10^–7^ M, which is directly comparable to the gold standard macrocyclic chelators H_2_ **macropa** and H_4_ **crown** under the same conditions.

**Figure 10:**
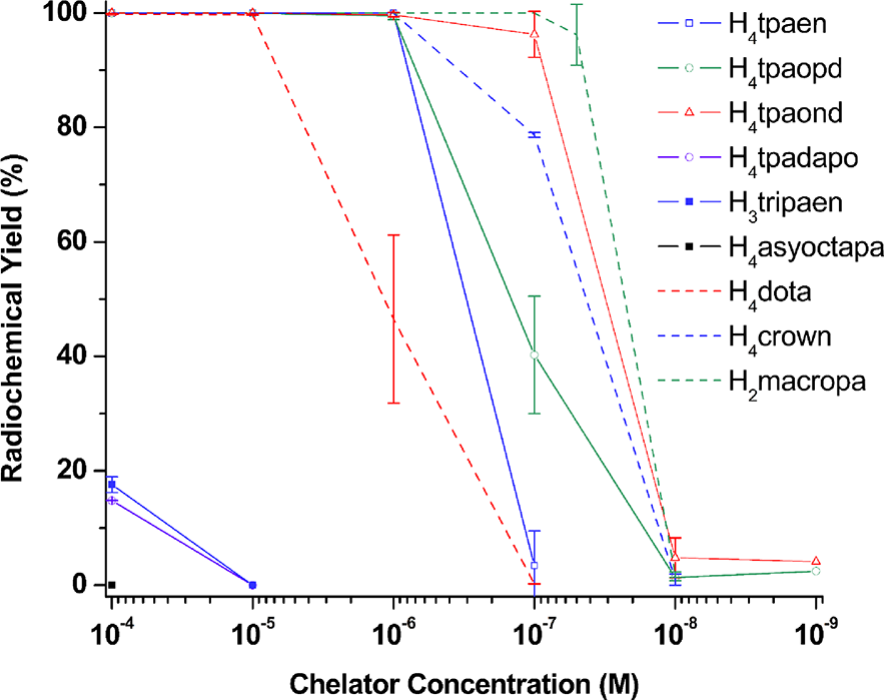
Concentration-dependent radiolabeling with [^225^Ac]­Ac^3+^ (20–25 kBq per reaction) in NH_4_OAc buffer (0.2 M, pH 6.5–7.0); *V_t_* = 50 μL. All reactions were performed at RT and RCYs determined by radio-TLC after 30 min. . Note: H_4_ **tpamxd**, H_4_ **tpapxd**, and H_4_ **asyoctapa** did not show any radiolabeling with [^225^Ac]­Ac^3+^. H_4_ **dota**, H_4_ **crown**, and H_2_ **macropa** were included as positive controls. Reactions with H_4_ **dota** were carried out at 85 °C for 30 min.

**Table 7:** Radiochemical Yields for Different Chelators Investigated in Concentration Dependent Radiolabeling with [^225^Ac]­Ac^3+^ ^a^

	**chelator concentration (M)**					
**chelator**	**1 × 10** ^**–3**^	**1 × 10** ^**–4**^	**1 × 10** ^**–5**^	**1 × 10** ^**–6**^	**1 × 10** ^**–7**^	**1 × 10** ^**–8**^
H_4_ **tpaen** ^b^	100	100	100	100	3(6)	
H_4_ **tpaopd** ^b^	100	100	100	>99	40(10)	1(1)
H_4_ **tpaond** ^b^	100	100	100	>99	96(4)	5(4)
H_4_ **tpadapo** ^b^,^c^		15	0	0	0	0
H_4_ **tpamxd** ^b^,^c^	0					
H_4_ **tpapxd** ^b^,^c^	0					
H_3_ **tripaen** ^b^,^c^		18(1)	0	0	0	0
H_4_ **asyoctapa** ^b^,^c^		0	0	0	0	0
H_4_ **dota** ^b^,^d^		100	100	47(15)	0	
H_4_ **crown** ^e^	100	100	100	100	79	1(1)
H_2_ **macropa** ^b^			100	100	96(5)	2(1)

a All reactions were performed at RT and RCYs determined by radio-TLC (footnotes b, c, and e) after 30 min. Performed with 2–4 replicates per concentration. Note: H_4_ **tpamxd**, H_4_ **tpapxd**, and H_4_ **asyoctapa** did not show any radiolabeling with [^225^Ac]­Ac^3+^. H_4_ **dota**, H_4_ **crown**, and H_2_ **macropa** were included as positive controls.

b TLC: Si–Al with the sodium citrate (0.4 M, pH 4) eluent.

c TLC: SA-paper with the EDTA (50 mM, pH 5.5) eluent.

d Reactions with H_4_ **dota** were carried out 85 °C for 30 min. Numbers in brackets indicate the standard deviation.

e TLC: Si–Al with EDTA (50 mM, pH 5.5).

By comparison, the remaining chelators showed poor compatibility with [^225^Ac]­Ac^3+^, either achieving low RCYs at high ligand concentration or showing no evidence of complex formation. Unlike H_4_ **py4pa** (also an excellent chelator for [^225^Ac]­Ac^3+^),^23^ the structural analogue H_4_ **tpamxd** and its regioisomeric H_4_ **tpapxd** did not radiolabel at all; this appears to be a consequence of their tendency to form insoluble polymers. On the other hand, the poor results obtained in radiolabeling with the chelator containing a 2-hydroxypropylene spacer (H_4_ **tpadapo**) seems to be due to the hydrogen bonding between the hydroxyl (OH) group and the tertiary amine nitrogen atoms, which limits the cavity size and prevents those nitrogen donors from forming part of the coordination sphere, too. Finally, the poor radiolabeling results obtained for the octadentate chelators H_3_ **tripaen** and H_4_ **asyoctapa** are a reflection of the mismatch between a low denticity ligand within a nonoptimal acyclic topology, and the coordination requirements of the large [^225^Ac]­Ac^3+^ ion (typically with coordination numbers 9 or 10).

Endogenous proteins challenge the stability of chelators in radiopharmaceuticals in vivo. Among others, apotransferrin, albumin, ceruloplasmin, superoxide dismutase, and metallothioneins are present in human serum and are known to be able to displace (transchelate) metal complexes in vivo. Because human serum contains such endogenous ligands, in vitro serum stability challenge assays can be predictive indicators of in vivo inertness of the radiolabeled chelates. Given the high affinity of [^225^Ac]­Ac^3+^ toward H_4_ **tpaen**, H_4_ **tpaopd**, and H_4_ **tpaond**, further studies of the kinetic inertness were undertaken in the presence of pooled human serum. High molar activity (100 kBq/nmol) samples of each ^225^Ac-labeled chelate were prepared and incubated in human serum at 37 °C for 8 days. [^225^Ac]­Ac-**tpaen** showed a marked drop in complex integrity, remaining 70% intact after 1 day in human serum, and showed a sustained gradual release over the course of the study, with 36% remaining labeled at 8 days. [^225^Ac]­Ac-**tpaopd** and [^225^Ac]­Ac-**tpaond** exhibited more favorable stability in serum, with [^225^Ac]­Ac-**tpaopd** maintaining >90% radiochemical integrity at 4 days (see Figure 11). At this point it should be noted that a small decrease in radiochemical purity is somewhat typical of ^225^Ac-labeled compounds, which can be attributed to the radiolytic potency of this radionuclide, causing degradation over time, a factor that can be mitigated by the inclusion of radiolysis quenchers as sodium ascorbate or gentisic acid.

**Figure 11:**
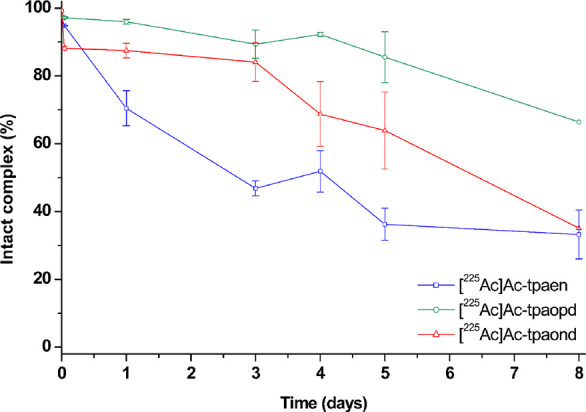
Human serum stability challenge assay of [^225^Ac]­Ac-**tpaen**, [^225^Ac]­Ac-**tpaopd**, and [^225^Ac]­Ac-**tpaond** (100 kBq/nmol) in human serum incubated at 37 °C for 8 days; percentage of the intact complex was determined by radio-TLC. All measurements were performed in triplicate.

The radiolabeling characteristics of the eight chelators were also examined with [^161^Tb]­Tb^3+^ (Figure 12, Table 8). Similarly to the [^225^Ac]­Ac^3+^ experiments, H_4_ **tpamxd** and H_4_ **tpapxd**, radiolabeling with [^161^Tb]­Tb^3+^ was unsuccessful. However, the other six ligands (not only the decadentate H_4_ **tpaen**, H_4_ **tpaopd**, H_4_ **tpaond**, but also H_4_ **tpadapo** and the octadentate H_4_ **asyoctapa** and H_3_ **tripaen**) showed efficient coordination of [^161^Tb]­Tb^3+^ with nearly quantitative RCYs achieved at 10^–5^ M. Furthermore, RCYs ca. 96% (H_4_ **tpadapo**, H_3_ **tripaen**), 94% (H_4_ **tpaopd**) and 93% (H_4_ **asyoctapa**) were achieved at 10^–6^ M. The structural study carried out with nonradioactive metal ions revealed that, with these acyclic ligands, the Tb^3+^ coordination requirements are satisfied with only eight donors. Therefore, it is not surprising, but rather expected, that, unlike what occurs with the large Ac^3+^ ion, both H_4_ **tpadapo** and the two octadentate ligands are effectively radiolabeled with the smaller [^161^Tb]­Tb^3+^.

**Figure 12:**
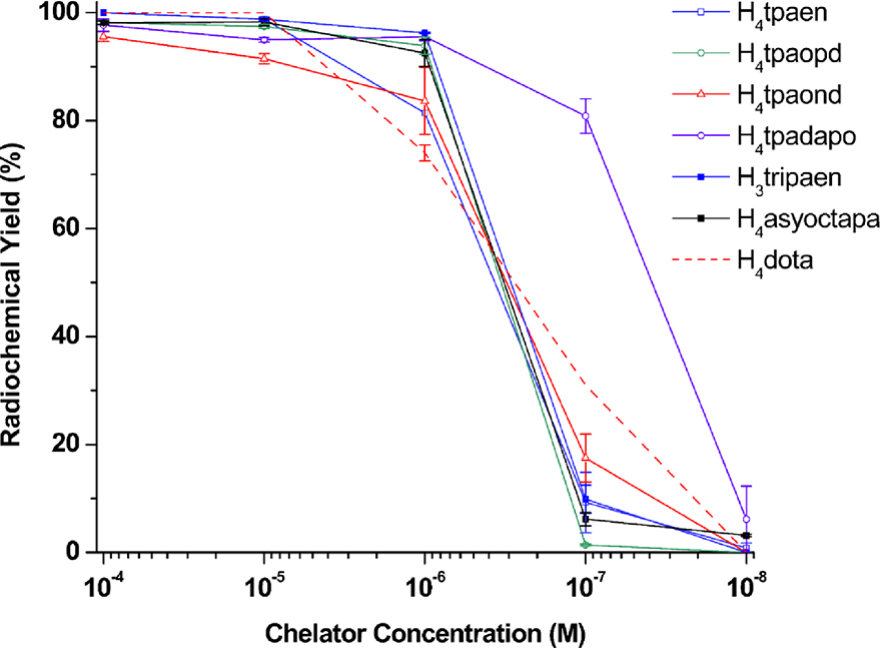
Concentration-dependent radiolabeling with [^161^Tb]­Tb^3+^ (100 kBq per reaction) in NH_4_OAc buffer (0.2 M, pH 6.0–6.5); *V_t_* = 50 μL. All reactions were performed at RT and RCYs determined by radio-TLC after 30 min. Note: H_4_ **tpamxd** and H_4_ **tpapxd** were tested but showed no radiolabeling with [^161^Tb]­Tb^3+^. H_4_ **dota** was included as a positive control. Reactions with H_4_ **dota** were carried out at 85 °C for 30 min.

**Table 8:** Radiochemical Yields for Different Chelators Investigated in Concentration Dependent Radiolabeling with [^161^Tb]­Tb^3+^ ^a^

	**chelator concentration (M)**					
**chelator**	**1 × 10** ^**–3**^	**1 × 10** ^**–4**^	**1 × 10** ^**–5**^	**1 × 10** ^**–6**^	**1 × 10** ^**–7**^	**1 × 10** ^**–8**^
H_4_ **tpaen** ^b^	100	100	99	81(2)	9(6)	1(1)
H_4_ **tpaopd** ^b^	100	98	97	94(2)	1(1)	0
H_4_ **tpaond** ^b^	100	96(1)	92(1)	84(6)	18(5)	0
H_4_ **tpadapo** ^b^		98(1)	95	96(1)	81(3)	6(1)
H_4_ **tpamxd** ^c^,^b^	0					
H_4_ **tpapxd** ^c^,^b^	0					
H_3_ **tripaen** ^b^		100	99	96	10(3)	0
H_4_ **asyoctapa** ^b^		98	98(1)	93(3)	6(1)	3
H_4_ **dota** ^c^,^d^	100	100	100	74(2)	31(27)	0

a All reactions were performed at RT and RCYs determined by radio-TLC (footnotes b and c) after 30 min. Performed with 2–4 replicates per concentration. Note: H_4_ **tpamxd** and H_4_ **tpapxd** were tested but showed no radiolabeling with ^161^Tb. H_4_ **dota** was included as a positive control.

b TLC: SA-paper with the EDTA (50 mM, pH 5.5) eluent.

c TLC: Si–Al with the sodium citrate (0.4 M, pH 4) eluent.

d Reactions with H_4_ **dota** were carried out at 85 °C for 30 min. Numbers in brackets indicate the standard deviation.

Additional human serum stability assays were carried out for the most favorable ^161^Tb-labeled complexes; each compound was prepared with a high molar activity (1 MBq/nmol) and diluted in pooled human serum (see Figure 13). [^161^Tb]­Tb-**tpaen** and [^161^Tb]­Tb-**asyoctapa** showed a high degree of kinetic inertness toward serum proteins, with <5% release of [^161^Tb]­Tb^3+^ over 5 days. [^161^Tb]­Tb-**tpaopd** and [^161^Tb]­Tb-**tpaond** show a fast initial release of activity after 1 h, remaining ∼70 and 60% intact, respectively, for the remainder of the study. In contrast, [^161^Tb]­Tb-**tripaen** and [^161^Tb]­Tb-**tpadapo** showed fast transchelation of [^161^Tb]­Tb^3+^ to serum proteins during the first 24 h, with <5% of each complex remaining intact by 4 days.

**Figure 13:**
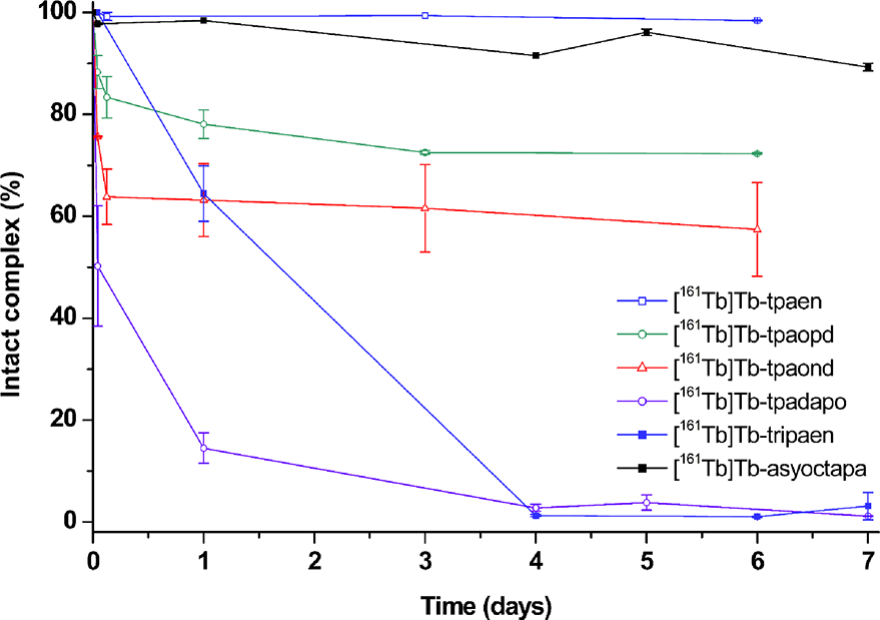
Human serum stability challenge assay with ^161^Tb-labeled chelators (1 MBq/nmol) in human serum incubated at 37 °C for 7 days; percentage of the intact complex was determined by radio-TLC. All measurements were performed in triplicate.

To date, we have only conducted a very preliminary study with [^177^Lu]­Lu^3+^, which has proven very promising. Given the favorable radiolabeling characteristics exhibited by H_4_ **tpaen** and H_4_ **asyoctapa** with [^161^Tb]­Tb^3+^, an initial study with [^177^Lu]­Lu^3+^ was undertaken to examine whether these excellent properties could be extended to the smallest radiolanthanide ion. Both chelators were found to coordinate [^177^Lu]­Lu^3+^ at RT within 30 min as shown in Figure 14 and Table 9.

**Figure 14:**
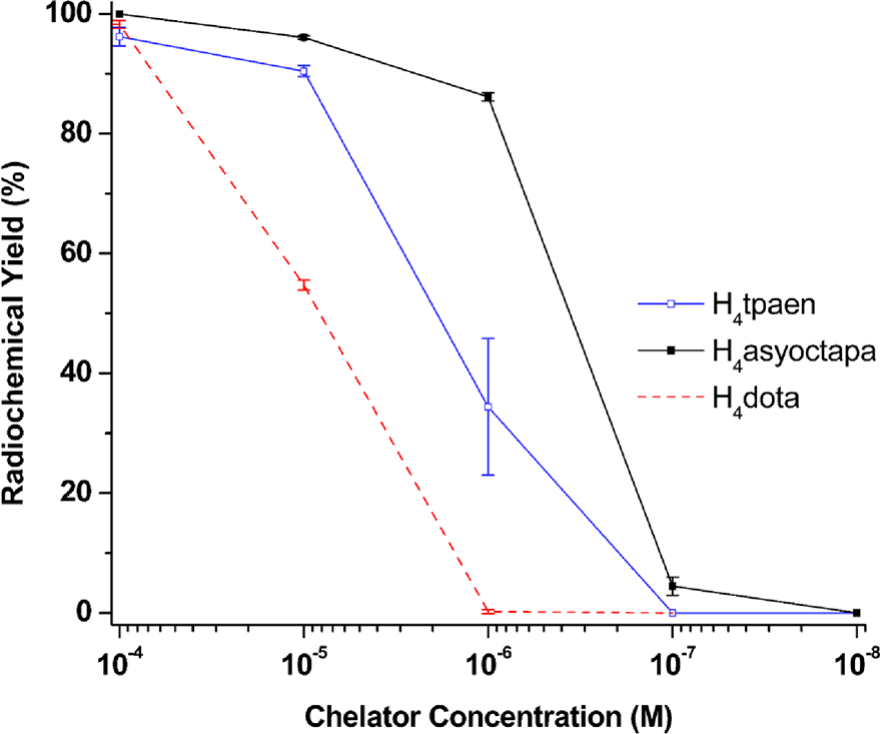
Concentration-dependent radiolabeling with [^177^Lu]­Lu^3+^ (75 kBq per reaction) in NH_4_OAc buffer (0.2 M, pH 6–6.5); *V_t_* = 50 μL. All reactions were performed at RT and RCYs determined by radio-TLC after 30 min. H_4_ **dota** was included as a positive control. Reactions with H_4_ **dota** were carried out 85 °C for 30 min.

**Table 9:** Radiochemical Yields for Different Chelators Investigated in Concentration Dependent Radiolabeling with [^177^Lu]­Lu^3+^ ^a^

	**chelator concentration (M)**					
**chelator**	**1 × 10** ^**–3**^	**1 × 10** ^**–4**^	**1 × 10** ^**–5**^	**1 × 10** ^**–6**^	**1 × 10** ^**–7**^	**1 × 10** ^**–8**^
H_4_ **tpaen** ^b^		96(2)	90(1)	34(11)	0	0
H_4_ **asyoctapa** ^b^		100	96	86(1)	5(2)	0
H_4_ **dota** ^c^,^d^	98	98	55(1)	0	0	

a All reactions were performed at RT and RCYs determined by radio-TLC (footnotes b and c) after 30 min. Performed with 2–4 replicates per concentration. H_4_ **dota** was included as a positive control.

b TLC: SA-paper with the EDTA (50 mM, pH 5.5) eluent.

c TLC: Si–Al with the sodium citrate (0.4 M, pH 4) eluent.

d Reactions with H_4_ **dota** were carried out 85 °C for 30 min. Numbers in brackets indicate the standard deviation.

## Conclusions

With the aim of finding a versatile chelating agent for actinium, terbium, and lutetium radiometal ions, a family of high-denticity acyclic chelating agents based on picolinate arms has been explored. An extensive study of complexation has been carried out in solution and solid state with nonradioactive Lu^3+^, Tb^3+^, and La^3+^, the latter as a surrogate for Ac^3+^, which supports the effectiveness of such chelators when radiolabeling. The decadentate four-arm ligands with the short spacer (ethylene (H_4_ **tpaen**), *o*-phenylene (H_4_ **tpaopd**) and 2,3-naphthylene (H_4_ **tpaond**)), which are very easy to synthesize, form helical mononuclear complexes with the three metal ions. The smaller ions (Tb^3+^ and Lu^3+^) satisfy their coordination preferences with the eight donors provided by the four picolinates, while La^3+^ completes its coordination sphere with the additional binding to the tertiary amine nitrogens. Depending on the size of the metal ion and the greater or lesser flexibility of the spacer, the arms reach a torsional compromise to achieve optimal (maximum) interactions between the metal ion and all donor atoms. The denticity, topology, and molecular architecture of these three chelators is optimal for [^225^Ac]­Ac^3+^ as demonstrated by radiolabeling assays. The three chelators achieve quantitative RCYs at chelate concentrations as low as 10^–6^ M within 30 min at RT and the rigid H_4_ **tpaond** has RCY of 96% at 10^–7^ M, which is directly comparable to the gold standard macrocyclic chelators H_2_ **macropa** and H_4_ **crown**. [^225^Ac]­Ac-**tpaopd** and [^225^Ac]­Ac-**tpaond** exhibited very favorable stability in serum with [^225^Ac]­Ac-**tpaopd** maintaining >90% radiochemical integrity in 4 days. These three chelators achieve nearly quantitative RCYs with [^161^Tb]­Tb^3+^ at 10^–5^ M, with H_4_ **tpaopd** reaching 94% at 10^–6^ M. [^161^Tb]­Tb-**tpaen** showed high kinetic inertness toward transmetalation by serum proteins, with <5% release of [^161^Tb]­Tb^3+^ over 5 days, whereas [^161^Tb]­Tb-**tpaopd** showed a fast initial release of activity after 1 h, thereafter remaining ∼70% intact. Considering these results, we conclude that H_4_ **tpaond** is an excellent candidate for the development of ^225^Ac-based radiopharmaceuticals, whereas both H_4_ **tpaen** and H_4_ **tpaopd** present an opportunity for the ^225^Ac/^155^Tb theranostic pair. The functionalization of these three chelating agents is currently underway to create BFCs, and both in vitro and in vivo studies will be conducted with these bioconjugates. Our results also demonstrate that with this type of decadentate four-arm chelator it is not necessary to incorporate a long spacer to create the appropriate cavity size to suitably match the large Ac^3+^ ion. In fact, the reason for the excellent behavior of H_4_ **py4pa** stems from the presence of the pyridine nitrogen atom in the long linker, and not from its length.

Considering RCY values and human serum stability studies, the octadentate dissymmetric four-arm chelator H_4_ **asyoctapa** appears to be a great opportunity, not only for developing terbium radiopharmaceuticals, but also for the promising ^177^Lu/^155^Tb pair. Our results show that this ligand was not labeled with [^225^Ac]­Ac^3+^ due to an incompatibility between a low denticity ligand, with a nonoptimal acyclic topology, and the coordination requirements of the large ion Ac^3+^. Even though the NMR study in solution carried out with La^3+^ demonstrates that this ligand coordinates with this large lanthanide ion and showed that the structure of the lanthanum complex is similar to that found for lutetium, the different behavior found for Ac^3+^ versus La^3+^ reminds us that although the La^3+^ ion is often used as a surrogate for Ac^3+^, we must be very cautious when making this extrapolation.

## Experimental Section

### Materials and Methods

All reagents and solvents were purchased from commercial suppliers (Sigma-Aldrich, Merck, Alfa-Aesar, Thermofisher Scientific, Agilent Technologies) and were used as received except for the acetonitrile used in synthesis, which was dried.^72^ Reactions were monitored by TLC (Fluka kiesegel, aluminum sheet). Flash chromatography was performed using FlashPure EcoFlex Silica (40 g), Redisep Rf Al_2_O_3_ (48g, pH = 7) or FlashPure EcoFlex Al_2_O_3_ (8g, pH = 7) and a Combiflash Rf column machine. Water was ultrapure (18.2 MΩ cm^–1^ at 25 °C, Milli-Q). ^1^H and ^13^C NMR spectroscopies were performed at room temperature on a Bruker AVANCE 500 MHz spectrometer at the “Servicios de Apoio á Investigación - SAI” of the University of A Coruña (Spain). Chemical shifts (δ) are quoted in ppm relative to residual solvent peaks as appropriate. Coupling constants (*J*) are provided in Hertz (Hz). ^1^H NMR signals were designated as follows: s (singlet), d (doublet), t (triplet), q (quartet), quin (quintet), m (multiplet), or a combination of these, with br representing a broad signal. High-resolution ESI-MS was performed on a Thermo LTQ-Orbitrap Discovery (SAI, University of A Coruña); results are labeled with *m*/*z* values [M + X]^±^. Microanalyses for C, H, and N were performed on a FlashEA112 (ThermoFinnigan) (SAI, University of A Coruña).

Radiolabeling studies were analyzed using instant thin-layer chromatography (iTLC) with aluminum-backed Silica TLC plates (Silica gel 60 F_254_; Supelco, Sigma-Aldrich) or Silicic acid impregnated paper TLC strips (Agilent technologies). TLCs were developed using either sodium citrate (0.4 M, pH 4) or edta (50 mM, pH 5), and scanned using an AR2000 TLC imaging scanner (Eckert & Zeigler) with P10 gas using WinScan V3_14 software. Radioactivity was quantified using a high-purity germanium (HPGe) detector (Mirion Technologies (Canberra) Inc.) with Genie 2000 software. Actinium-225 was produced at TRIUMF using the 520 MeV Isotope Production Facility (IPF) via irradiation of ^232^Th foils at ∼480 MeV at up to 12,500 μAh; radionuclide purification was carried out as previously reported.^73,74^ Terbium-161 was purchased from TerThera B.V. (The Netherlands); lutetium-177 was purchased from McMaster University (Hamilton, Ontario, Canada). All work involving radionuclides at TRIUMF is carried out in shielded fumehoods by authorized personnel with nuclear energy worker (NEW) status, after completion of an internal Advanced Radiation Protection Training course.

### Synthesis

Compounds (**1**)^38^ and (**4**)^75^ (see Scheme 2) were prepared according to the literature. Ethyl 6-(chloromethyl)­picolinate was purchased from CheMatech.

#### 6,6′,6″,6‴-((Ethane-1,2-diylbis­(azanetriyl))­tetrakis­(methylene))­tetrapicolinic acid (H_4_ **tpaen**, H_4_ **L** ^**1**^)

Ethylenediamine (65.0 μL, 0.982 mmol), anhydrous potassium carbonate (0.680 g, 4.92 mmol), KI (0.707 g, 4.24 mmol) and ethyl 6-(chloromethyl)­picolinate (0.850 g, 5.50 mmol) were refluxed in anhydrous acetonitrile (35 mL) under argon atmosphere for 48 h. Once the reaction was complete, the mixture was allowed to cool to room temperature and filtered. The filtrate was evaporated under reduced pressure to give an orange oily residue which was dissolved in 35 mL of CH_2_Cl_2_, washed with water (2 × 25 mL) and then dried over anhydrous Na_2_SO_4_. After filtering, the solvent was removed under reduced pressure to give an orange oil corresponding to the ester derivative, which was refluxed overnight in a 6 M HCl aqueous solution (12 mL) to give the expected acid isolated as a hydrochloric salt (0.254 g; yield 45%). ^**1**^ **H NMR** (500 MHz, D_2_O) δ (ppm): 8.00–7.93 (m, 8H, He Hf), 7.60 (d, *J* = 7.3 Hz, 4H, Hd), 4.49 (s, 8H, Hb), 3.77 (s, 4H, Ha). ^**13**^ **C NMR** (126 MHz, D_2_O) δ (ppm): 166.17 (Ch), 151.44 (*Cc*), 146.45 (Cg), 141.22 (Ce), 128.11 (Cd), 125.30 (Cf), 58.09 (Cb), 50.89 (Ca). (Figures S1–S5). **HR-MS ESI** ^**+**^: *m*/*z* calcd. for [C_30_H_28_N_6_O_8_+H]^+^: 601.2042; found: 601.2045 (ppm: 0.50). **HR-MS ESI** ^**‑**^: *m*/*z* calcd. for [C_30_H_28_N_6_O_8_–H]^−^: 599.1895; found 599.1889 (ppm: −1.0). **Elemental Analysis (EA)**: Experimental (Calculated for **C**_**30**_ **H**_**28**_ **N**_**6**_ **O**_**8**_ **·2.5HCl**): **C%** 52.08 (52.04); **H%** 4.32 (4.41); **N%** 11.97 (12.14).

#### 6,6′,6″,6‴-((1,2-Phenylenebis­(azanetriyl))-tetrakis­(methylene))­tetrapicolinic acid (H_4_ **tpaopd**, H_4_ **L** ^**2**^)

It was also synthesized as a hydrochloric salt in a similar way to H_4_ **L** ^**1**^ using 1,2-phenylenediamine (99.4 mg, 0.920 mmol), anhydrous potassium carbonate (0.637 g, 4.61 mmol), KI (0.662 g, 3.99 mmol) and ethyl 6-(chloromethyl)­picolinate (0.796 g, 3.99 mmol). Brown oily ester was deprotected obtaining 0.389 g of the brownish solid chelator. Yield 57%. ^**1**^ **H NMR** (500 MHz, D_2_O) δ (ppm): 8.14 (t, *J* = 7.8 Hz, 4H, He), 8.00 (d, *J* = 7.8 Hz, 4H, Hd), 7.64 (d, 4H, Hf), 7.24 (dd, *J* = 6.0, 3.6 Hz, 2H, Hj), 7.06 (dd, *J* = 6.1, 3.5 Hz, 2H, Hi), 4.81 (s, 8H, Hb), ^**13**^ **C NMR** (126 MHz, D_2_O) δ (ppm): 165.15 (Ch), 153.99 (*Cc*), 146.64 (Cg), 143.41 (Ce), 143.28 (Ca), 128.58 (Cd), 125.57 (Cf), 124.72 (Cj), 123.30 (Ci), 56.03 (Cb). (Figures S6–S10). **HR-MS ESI** ^**+**^: *m*/*z* calcd. for [C_34_H_28_N_6_O_8_+Na]^+^: 671.1861; found: 671.1866 (ppm: 0.74). **HR-MS ESI** ^**+**^: *m*/*z* calcd. for [C_34_H_28_N_6_O_8_–H]^−^: 647.1895; found 647.1893 (ppm: −0.31). **Elemental Analysis (EA)**: Experimental (Calculated for **C**_**34**_ **H**_**28**_ **N**_**6**_ **O**_**8**_ **·2.5HCl**): **C%** 55.30 (55.20); **H%** 3.65 (4.15); **N%** 10,78 (11.36).

#### 6,6′,6″,6‴-((Naphthalene-2,3-diylbis­(azanetriyl))­tetrakis­(methylene))­tetrapicolinic acid (H_4_ **tpaond**, H_4_ **L** ^**3**^)

It was also synthesized as a hydrochloric salt in a similar way to H_4_ **L** ^**1**^ using naphthalene-2,3-diamine (0.117 mg, 0.734 mmol), anhydrous potassium carbonate (0.513 g, 3.71 mmol), KI (0.532 g, 3.21 mmol) and ethyl 6-(chloromethyl)­picolinate (0.640 g, 3.18 mmol). The mixture was allowed to react for 72 h and washed three times with distilled water instead of two. Greenish brown oily ester was deprotected obtaining 0.198 g of the greenish solid chelator. Yield 31%. ^**1**^ **H NMR** (500 MHz, D_2_O, pD = 12) δ (ppm): 7.57 (d, *J* = 6.7 Hz, 4H, Hf), 7.52 (dd, *J* = 6.2, 3.4 Hz, 2H, Hk), 7.41 (t, *J* = 7.7 Hz, 4H, He), 7.30 (s, 2H, Hi), 7.21 (dd, *J* = 6.2, 3.3 Hz, 2H Hl), 7.04 (d, *J* = 7.8 Hz, 4H, Hd), 4.78 (s, 8H, Hb). ^**13**^ **C NMR** (126 MHz, D_2_O, pD = 12) δ (ppm): 173.18 (Ch), 157.53 (*Cc*), 153.02 (Cg), 142.06 (Ca), 137.80 (Ce), 129.50 (Cj), 126.14 (Ck), 125.10 (Cl), 124.74 (Cd), 121.99 (Cf), 119.84 (Ci), 57.29 (Cb). (Figures S11–S15). **HR-MS ESI** ^**+**^: *m*/*z* calcd. for [C_38_H_30_N_6_O_8_+Na]^+^: 721.2018; found: 721.2025 (ppm: 0.97). **HR-MS ESI** ^**‑**^: *m*/*z* calcd. for [C_38_H_30_N_6_O_8_–H]^−^: 697.2052; found 697.2053 (ppm: 0.14). **Elemental Analysis (EA)**: Experimental (Calculated for **C**_**38**_ **H**_**28**_ **N**_**6**_ **O**_**8**_ **·HCl 7H**_**2**_ **O**): **C%** 53.20 (52.99); **H%** 5.42 (5.27); **N%** 9.02 (9.76).

#### 6,6′,6″,6‴-(((1,3-Phenylenebis­(methylene))­bis­(azanetriyl))­tetrakis­(methylene)) tetrapicolinic acid (H_4_ **tpamxd**, H_4_ **L** ^**4**^)

It was also synthesized as a hydrogen chloride salt (0.412 g, white solid) in a similar way to H_4_ **L** ^**1**^ using 1,3-phenylenedimethanamine (0.121 g, 0.887 mmol), anhydrous potassium carbonate (0.615 g, 4.45 mmol), KI (0.639 g, 3.85 mmol) and ethyl 6-(chloromethyl)­picolinate (0.768 g, 3.85 mmol). Yield 57%. ^**1**^ **H NMR** (500 MHz, D_2_O) δ (ppm): 7.83 – 7.77 (m, 9H, He Hf Hl), 7.49 (dd, *J* = 7.8, 1.7 Hz, 2H, Hj), 7.45 – 7.40 (m, 4H, Hd), 7.36 (t, *J* = 7.7 Hz, 2H, Hk), 4.61 (s, 8H, Hb), 4.56 (s, 4H, Ha). ^**13**^ **C NMR** (126 MHz, D_2_O) δ (ppm): 167.33 (Ch), 150.05 (*Cc*), 147.32 (Cg), 140.04 (Ce), 133.05 (Cl), 132.73 (Cj), 130.73 (Ck), 129.91 (Ci), 127.56 (Cd), 124.79 (Cf), 59.78 (Ca), 57.96 (Cb). (Figures S16–S20). **HR-MS ESI** ^**+**^: *m*/*z* calcd. for [C_36_H_32_N_6_O_8_+H]^+^: 677.2355 ; found: 677.2362 (ppm: 1.03). **HR-MS ESI** ^**‑**^: *m*/*z* calcd. for [C_36_H_32_N_6_O_8_–H]^−^: 675.2208; found 675.2198 (ppm: −1.48). **Elemental Analysis (EA)**: Experimental (Calculated for **C**_**36**_ **H**_**32**_ **N**_**6**_ **O**_**8**_ **·4HCl**): **C%** 52.08 (52.57); **H%** 4.28 (4.41); **N%** 10.99 (10.22).

#### 6,6′,6″,6‴-(((1,4-Phenylenebis­(methylene))­bis­(azanetriyl))­tetrakis­(methylene)) tetrapicolinic acid (H_4_ **tpapxd**, H_4_ **L** ^**5**^)

It was synthesized also as a hydrochloric salt (0.538 g, white solid) in a similar way to H_4_ **L** ^**1**^ using 1,4-phenylenedimethanamine (0.173 g, 1.27 mmol), anhydrous potassium carbonate (0.875 g, 6.33 mmol), KI (0.920 g, 5.54 mmol) and ethyl 6-(chloromethyl)­picolinate (1.097 g, 5.50 mmol). Yield 53%. ^**1**^ **H NMR** (500 MHz, D_2_O;) δ (ppm): 7.76 (t, *J* = 7.8 Hz, 4H, He), 7.70 (d, *J* = 7.7 Hz, 4H, Hf), 7.52 (s, 4H, Hj), 7.45 (d, *J* = 7.8 Hz, 4H, Hd), 4.61 (s, 12H, Ha, Hb). ^**13**^ **C NMR** (126 MHz, D_2_O) δ (ppm): 167.68 (Ch), 150.64 (*Cc*), 147.34 (Cg), 140.51 (Ce), 132.62 (Cj), 131.78 (Ci), 128.49 (Cd), 125.63 (Cf), 60.52 (Ca), 58.71 (Cb). (Figures S21–S25). **HR-MS ESI** ^**+**^: *m*/*z* calcd. for [C_36_H_32_N_6_O_8_+H]^+^: 677.2355; found: 677.2357 (ppm: 0.30). **HR-MS ESI** ^**‑**^: *m*/*z* calcd. for [C_36_H_32_N_6_O_8_–H]^−^: 675.2208; found 675.2212 (ppm: 0.59). **Elemental Analysis (EA)**: Experimental (Calculated for **C**_**36**_ **H**_**32**_ **N**_**6**_ **O**_**8**_ **·3.25HCl**): **C%** 54.65 (54.38); **H%** 4.34 (4.47); **N%** 10.20 (10.57). Colorless plate-shaped single crystals of formula H_4_ **L** ^**5**^·6H_2_O suitable for X-ray diffraction were grown by slow evaporation of a diluted solution of the corresponding hydrochloride salt in water/acetonitrile.

#### 6,6′,6″,6‴-(((2-Hydroxypropane-1,3-diyl)­bis­(azanetriyl))­tetrakis­(methylene)) tetrapicolinic acid (H_4_ **tpadapo**, H_4_ **L** ^**6**^)

1,3-diaminopropan-2-ol (60.1 mg, 0.667 mmol), anhydrous potassium carbonate (1.84 g, 13.3 mmol), KI (0.442 g, 2.66 mmol) and ethyl 6-(chloromethyl)­picolinate (0.532 g, 2.66 mmol) were refluxed in anhydrous acetonitrile (15 mL) under argon atmosphere for 72 h. Once the reaction was complete, the mixture was allowed to cool to room temperature and the solvent of the mixture was removed under reduced pressure to give an orange oily residue which was dissolved in 20 mL of CHCl_3_, washed with water (2 × 10 mL) and then dried over anhydrous Na_2_SO_4_. After filtering, the solvent was removed under reduced pressure to give a yellow crude which was purified by column chromatography (Redisep Rf Al_2_O_3_, 48 g, pH = 7, CHCl_3_/CH_3_OH: 1% CH_3_OH for 2.8 min *t*_R_ = 1.5 min). obtaining the corresponding ester derivate, which was refluxed overnight in a 6 M HCl aqueous solution (6 mL) to give the expected acid isolated as a white hydrochloric salt (0.152 g; yield 30%). ^**1**^ **H NMR** (500 MHz, D_2_O;) δ (ppm): 8.05–8.01 (m, 8H, Hd Hf), 7.68–7.61 (m, 4H, He), 5.04–4.96 (m, 1H, Hi), 4.85 (s, 8H, Hb), 3.84 (dd, *J* = 13.7, 2.8 Hz, 2H, Ha), 3.69 (dd, *J* = 13.7, 9.1 Hz, 2H, Ha). ^**13**^ **C NMR** (126 MHz, D_2_O) δ (ppm): 166.64 (Ch), 150.07 (*Cc*), 146.32 (Cg), 140.39 (Ce), 127.80 (Cd), 124.45 (Cf), 62.76 (Ci), 59.03 (Cb), 58.44 (Ca). (Figures S26–S30). **HR-MS ESI** ^**+**^: *m*/*z* calcd. for [C_31_H_30_N_6_O_9_+H]^+^: 631.2148; found: 631.2152 (ppm: 0.63). **HR-MS ESI** ^**–**^: *m*/*z* calcd. for [C_31_H_30_N_6_O_9_–H]^−^: 629.2001 ; found 629.2009 (ppm: 1.27). **Elemental Analysis (EA)**: Experimental (Calculated for **C**_**31**_ **H**_**30**_ **N**_**6**_ **O**_**9**_ **·4HCl**): **C%** 48.11 (47.95); **H%** 4.28 (4.41); **N%** 9.79 (10.32).

#### Diethyl 6,6′-(((2-((N-((6-(Ethoxycarbonyl)­pyridin-2-yl)­methyl)-2-nitrophenyl) sulfonamido) ethyl)­azanediyl)­bis­(methylene))­dipicolinate (**2**)

Compound (**1**) (0.205 g, 0.836 mmol), anhydrous potassium carbonate (0.580 g, 4.20 mmol), KI (0.453 g, 2.73 mmol) and ethyl 6-(chloromethyl)­picolinate (0.544 g, 2.73 mmol) were refluxed in anhydrous acetonitrile (35 mL) under argon atmosphere for 72 h. Once the reaction was complete, the mixture was allowed to cool to room temperature and the solvent of the mixture was removed under reduced pressure to give an orange oily residue which was dissolved in 35 mL of CH_2_Cl_2_, washed with 35 mL of water and then dried over anhydrous Na_2_SO_4_. After filtering, the solvent was removed under reduced pressure to give a brown crude which was purified by column chromatography (FlashPure EcoFlex silica 40 g, CH_2_Cl_2_/CH_3_OH: 0% CH_3_OH for 1 min and 0% CH_3_OH to 32% CH_3_OH for 1 to 4.9 min *t*_R_ = 3.75 min). obtaining 0.316 g of the corresponding the ester derivate. Yield 54%. **HR-MS ESI** ^**+**^: *m*/*z* calcd. for [C_35_H_38_N_6_O_10_S+H]^+^: 735.2443; found: 735.2433 (ppm: −1.36).

#### Diethyl 6,6′-(((2-(((6-(Ethoxycarbonyl)­pyridin-2-yl)­methyl)­amino)­ethyl)­azanediyl)­bis (methylene))­dipicolinate (**3**)

To a solution of **2** (0.316 g, 0.430 mmol) in tetrahydrofuran (4 mL), thiophenol (52.5 μL, 0.516 mmol) and anhydrous potassium carbonate (excess, ∼0.5 g) were added. The reaction mixture was stirred at RT for 72 h. The potassium carbonate was filtered several times until it was completely removed, and then the solvent was removed under reduced pressure to be purified by column chromatography. (Redisep Rf Al_2_O_3_, 48 g, pH = 7, CH_2_Cl_2_/CH_3_OH: 0% CH_3_OH to 32% CH_3_OH for 4.1 min *t*_R_ = 0.9 min), obtaining 0.192 g of the corresponding ester. Yield 81%. **HR-MS ESI** ^**+**^: *m*/*z* calcd. for [C_29_H_35_N_5_O_6_+H]^+^: 601.2042; found: 601.2045 (ppm: 0.73).

#### 6,6′-(((2-(((6-Carboxypyridin-2-yl)­methyl)­amino)­ethyl)­azanediyl)­bis­(methylene)) dipicolinic acid (H_3_ **tripaen**, H_3_ **L** ^**7**^)

It was synthesized also as a hydrochloric salt in quantitative yield (0.0402 g, orange oil) via deprotection of 50.3 mg of the compound **(3)** with 3 mL of HCl (6 M) at reflux overnight. ^**1**^ **H NMR** (500 MHz, D_2_O) δ (ppm): 8.50 (t, *J* = 8.0 Hz, 1H, Hm), 8.23 (d, *J* = 6.6 Hz, 1H, Hn), 8.05 (d, *J* = 9.3 Hz, 1H, Hl), 7.94–7.87 (m, 3H, Ht He Hu), 7.56–7.48 (m, 3H, Hs Hd Hf), 4.90 (s, 2H, Hj), 4.62 (s, 2H, Hb), 4.46 (s, 2H, Hq), 4.44 (s, 1H, H–N), 3.80 (t, *J* = 5.8 Hz, 2H, Hi), 3.65 (t, *J* = 5.9 Hz, 2H, Ha). ^**13**^ **C NMR** (126 MHz, D_2_O) δ (ppm): 167.26 (Cp+Ch+Cw), 166.44 (Cv+Cg), 162.07 (Co), 156.88 (Ck), 150.55 (Cr), 147.78 (*Cm*), 146.43 (*Cc*), 140.72 (Ct), 139.79 (Ce), 128.16 (Cd), 127.30­(Cl), 125.84 (Cn), 125.67 (Cu), 125.47 (Cs), 59.48­(Cj), 58.51 (Cb), 52.23 (Cq), 50.72 (Ci), 42.98 (Ca). (Figures S33–S37). **HR-MS ESI** ^**+**^: *m*/*z* calcd. for [C_23_H_23_N_5_O_8_+H]^+^: 466.1722; found: 466.1726 (ppm: 0.86). **HR-MS ESI** ^**–**^: *m*/*z* calcd. for [C_23_H_23_N_5_O_8_–H]^−^: 464.1575; found: 464.1572 (ppm: −0.65).

#### Diethyl 6,6′-(((2-(Bis­(2-(tert-butoxy)-2-oxoethyl)­amino)­ethyl)­azanediyl)­bis­(methylene)) dipicolinate (**5**)

0.130 g, (0.336 mmol) of (**4**) was dissolved in 12 mL of anhydrous acetonitrile. Et_3_N (0.164 mL, 1.18 mmol) was added, and the mixture was stirred 15 min at RT, under Ar. *tert*-butyl 2-bromoacetate (0.131g, 1.14 mmol) was added and the mixture was stirred at 80 °C 24 h, under Ar. When the reaction was finished, the solvent was removed under reduced pressure and the residue was dissolved in 20 mL of CH_2_Cl_2_. The organic layer was washed with water (2 × 15 mL), dried with anhydrous sodium sulfate, and filtered. The solvent was eliminated under reduced pressure to give an orange crude product which was purified by column chromatography (FlashPure EcoFlex Al_2_O_3_ 8 g, pH = 7, CH_2_Cl_2_/CH_3_OH 0% B for two min *t*_R_ = 0.5 min). 0.131 g of **(5)** were in the form of an orange-colored oil obtained after removing the solvent at reduced pressure. Yield 62%. ^**1**^ **H NMR** (500 MHz, MeOD; Figure S38) δ (ppm): 7.89 (dd, *J* = 7.3, 1.5 Hz, 2H, He Hf), 7.86–7.76 (m, 4H, Hd), 4.33 (q, *J* = 7.1 Hz, 4H, Hl), 3.84 (s, 4H, Hb), 3.26 (s, 4H, Hj), 2.79 (t, *J* = 6.8 Hz, 2H, Hi), 2.61 (t, *J* = 6.8 Hz, 2H, Ha), 1.37 (d, *J* = 2.6 Hz, 6H, Hm), 1.30 (s, 18H, Ho). **HR-MS ESI** ^**+**^: *m*/*z* calcd. for [C_33_H_48_N_4_O_7_+H]^+^: 615.3389; found: 615.3394 (ppm: 0.81).

#### 6,6′-(((2-(Bis­(carboxymethyl)­amino)­ethyl)­azanediyl)­bis­(methylene))­dipicolinic acid (H_4_ **asyoctapa**, H_4_ **L** ^**8**^)

It was isolated as a hydrochloric salt in a quantitative yield (0.0821 g, yellow oil) via deprotection of the compound **(5)** with 4 mL of HCl (6M) at reflux overnight. ^**1**^ **H NMR** (500 MHz, D_2_O) δ (ppm): 8.01 (m, 4H, He Hf), 7.67 (dd, *J* = 6.0, 3.0 Hz, 2H, Hd), 4.52 (s, 4H, Hb), 3.87 (s, 4H, Hj), 3.66 (t, *J* = 5.3 Hz, 2H, Hi), 3.58 (t, *J* = 5.3 Hz, 2H, Ha). ^**13**^ **C NMR** (126 MHz, D_2_O) δ (ppm): 171.20 (Ck), 166.06 (Ch), 151.62 (*Cc*), 146.19 (Cg), 141.56 (Cf), 128.39 (Cd), 125.22 (Ce), 57.91 (Cb), 55.85 (Cj), 51.84, (Ca), 51.06 (Ci). (Figures S39–S43). **HR-MS ESI** ^**+**^: *m*/*z* calcd. for [C_20_H_22_N_4_O_8_+H]^+^: 447.1510; found: 447.1514 (ppm: 0.89). **HR-MS ESI** ^**+**^: *m*/*z* calcd. for [C_20_H_22_N_4_O_8_–H]^−^: 445.1364; found 445.1368 (ppm: 0.90).

##### {[La­(**tpaen**)]­Na­(H_2_O)_4_}_2_·6H_2_O

Colorless prismatic single crystals suitable for X-ray diffraction were grown by slow evaporation of a solution containing equimolar amounts of H_4_ **tpaen** and LaCl·7H_2_O in D_2_O. The solution was adjusted to pD 6 by addition of NaOD.

##### {[La­(**tpaopd**)]­Na­(H_2_O)_4_}

Colorless needle-shaped single crystals suitable for X-ray diffraction were grown by slow evaporation of a solution containing equimolar amounts of H_4_ **tpaopd** and LaCl_3_·7H_2_O in D_2_O at pD 6 by addition of NaOD, and acetonitrile (20%).

##### {[La­(**tpaond**)]­Na·13.75H_2_O}

Yellow prism-shaped single crystals suitable for X-ray diffraction were grown by slow evaporation of a solution containing equimolar amounts of H_4_ **tpaond** and LaCl_3_·7H_2_O in D_2_O at pD 6 by addition of NaOD, and acetonitrile (20%).

##### {[Tb­(**tpaopd**)]­K·3H_2_O}

Colorless plate-shaped single crystals suitable for X-ray diffraction were grown by slow evaporation of a solution containing equimolar amounts of H_4_ **tpaopd** and TbCl_3_·6H_2_O in H_2_O/acetonitrile (60:40). The solution was adjusted to pH 6 by addition of KOH.

##### {[Tb­(**tpaond**)]­K·9H_2_O}_∞_

Colorless plate-shaped single crystals suitable for X-ray diffraction were grown by slow evaporation of a solution containing equimolar amounts of H_4_ **tpaond** and TbCl_3_·6H_2_O in H_2_O/acetonitrile (60:40). The solution was adjusted to pH 6 by addition of KOH.

##### {[Lu­(**tpaopd)**]­K­(H_2_O)_*n*_}

Colorless needle-shaped single crystals suitable for X-ray diffraction were grown by slow evaporation of a solution containing equimolar amounts of H_4_ **tpaopd** and LuCl_3_·7H_2_O in D_2_O:CD3CN (8:2) at pD 6 by addition of KOH.

##### {[Tb­(H_2_ **tpapxd**)­(H_2_O)]­Cl·4H_2_O}_∞_

Colorless plate-shaped single crystals suitable for X-ray diffraction were grown by slow evaporation of a solution of equimolar amounts of H_4_ **tpapxd** and TbCl_3_·6H_2_O in H_2_O (10^–3^ M). The solution was adjusted to pH 4.5 with NaOAc buffer.

### X-ray Crystallography

The X-ray intensity data of colorless plated-shaped crystals of H_4_ **tpapxd**·6H_2_O and {[Tb­(H_2_ **tpapxd**)­(H_2_O)]­Cl·4H_2_O}_∞_ were measured on a Bruker APEX II area detector diffractometer using MoK_α_ radiation (TRIUMPH monochromator, sealed X-ray tube) for the former and on a Bruker D8 VENTURE diffractometer for the latter. Both crystals held at *T* = 100(2) K during data collection. The total number of runs and images were based on the strategy calculation from the program *APEX5*. For both crystals, the unit cell was refined using *SAINT V8.40B*.^76^ *TWINABS-2012/1* was used for absorption correction for H_4_ **tpapxd**·6H_2_O, whereas *SADABS-2016/2* ^77^ was employed for {[Tb­(H_2_ **tpapxd**)­(H_2_O)]­Cl·4H_2_O}_∞_. Both structures were solved with the *SHELXT 2018/2* solution program^78^ using intrinsic Phasing methods and by using *OLEX2* ^79^ as the graphical interface. Both models were refined with *SHELXL 2019/3* ^80^ using full matrix least-squares minimization on *F* ^2^. All non-hydrogen atoms were refined anisotropically. All C–H hydrogen atoms were calculated geometrically and refined using the riding mode, while all O–H hydrogen atoms and any other hydrogen atom involved in hydrogen bonding were located in difference maps and refined freely.

The X-ray intensity data of crystals {[La­(**tpaopd**)]­Na­(H_2_O)_4_}, {[La­(**tpaond**)]­Na·13.75H_2_O} {[Tb­(**tpaond**)]­K·9H_2_O}_∞_ and {[Lu­(**tpaopd)**]­K­(H_2_O)_*n*_} were measured on a Bruker APEX-II diffractometer, whereas {[La­(**tpaen**)]­Na­(H_2_O)_4_}_2_·6H_2_O was measured on a Bruker D8 VENTURE PHOTON-III C14 diffractometer system equipped with a Incoatec IμS 3.0 microfocus sealed tube (Mo Kα, λ = 0.71073 Å) and a multilayer mirror monochromator. {[Tb­(**tpaopd**)]­K·3H_2_O} was measured on a Rigaku XtaLAB Synergy, Dualflex, HyPix-Arc 150 diffractometer. Data were corrected for Lorentz and polarization effects and for absorption using the Multi-Scan method (*SADABS*)^77^ except for {[Tb­(**tpaopd**)]­K·3H_2_O} for which a numerical absorption correction based on Gaussian integration over a multifaceted crystal model. This empirical absorption correction was performed using spherical harmonics, implemented in *SCALE3 ABSPACK* ^81^ scaling algorithm. Complex scattering factors were taken from the program *SHELX 2019* running under the *WinGX* program system.^82^ The measurement was performed at 100 K for all crystals but for {[Tb­(**tpaopd**)]­K·3H_2_O}, which showed a modulated structure at this temperature, so we repeated the measurement at 293 K. At this temperature the modulation disappeared, so the final refinement was performed with this data set. The six structures were refined by full-matrix least-squares on *F* ^2^ with *SHELXL 2019*.^80^ The hydrogen atoms were included in calculated positions and refined in riding mode except those of the water molecules that were located in the difference Fourier map and the usual restraints for water were applied (DFIX 0.96 for O–H distance and DANG 1.52 for H–H distance). Heavily disordered water molecules were found in all crystals, some of them coordinated to a sodium or a potassium ion and some others not coordinated, but all of them forming a very large and complicated hydrogen bond net. Moreover, some of them were located close to special positions. All of this made a good model difficult for them. The disorder was solved in most cases just fixing occupation factors and/or the position of some hydrogen atoms; all non-hydrogen atoms were refined anisotropically. However, for {[La­(**tpaond**)]­Na·13.75H_2_O} and {[Lu­(**tpaopd)**]­K­(H_2_O)_*n*_} it was not possible to find a reasonable model for the disorder, so that the squeeze^83^ procedure *PLATON* was performed. This procedure takes care of the contribution of a heavily disordered solvent to the calculated structure factors by back-Fourier transformation of the continuous density found in a masked region of the difference map. The masked region is defined as the solvent accessible region left by the ordered part of the structure. In the case of {[Lu­(**tpaopd)**]­K­(H_2_O)_*n*_, it was also necessary to mask the potassium ion to reach satisfactory results. Finally, the refinement converged in both crystals with anisotropic displacement parameters for all non-hydrogen atoms.

Crystal data and details on data collection and refinement of the eight crystal structures are summarized in Tables S1 and S2 (SI).

### Solution Thermodynamics

Protonation equilibria of the chelators were studied by titrations of 20 mL solutions containing [H_4_ **L** ^**2**^] = 2.49 × 10^–4^ M; [H_4_ **L** ^**3**^] = 2.47 × 10^–4^ M; [H_4_ **L** ^**4**^] = 2.62 × 10^–4^ M; [H_4_ **L** ^**5**^] = 2.52 × 10^–4^ M and [H_4_ **L** ^**6**^] = 2.27 × 10^–4^ M. Titrations were carried out in a thermostated cell at 25 °C under a nitrogen (N_2_) stream using potassium hydroxide as titrant. The ionic strength of both titrant and titration solution was adjusted to *I* = 0.1 M with KCl. Titrations were conducted covering pH 3 to 11 (100–150 experimental data points per titration); titrations were completed within 1.5–2 h.

All potentiometric titrations were carried out using an automated system composed of a buret (CRISON MicroBU 2031) with a 2.5 mL Hamilton syringe and a pH meter (CRISON GLP 22) equipped with a combination glass electrode (Radiometer Analytical). An in-lab homemade program was used to control the titrant additions and to record the readings of the automated titration system. The glass electrode was calibrated daily in hydrogen ion concentration by direct titration of a previously standardized HCl solution with freshly prepared KOH solution, as in the literature^84,85^ The protonation constants of the ligands were determined by analyzing potentiometric data with *HyperQuad2013* ^86^ (Table 1 and Figure S127). Six acid–base equilibria were entered into the *HyperQuad* program associated with the four picolinates in the molecule and two for the amines, but the first of these equilibria (very low pH/p*K*) could not be determined due to the experimental conditions, so the corresponding log *K* value was set to constant. In the case of H_4_ **L** ^**6**^, the presence of the alcohol required the inclusion of an additional equilibrium, which could not be determined and therefore, the log *K* value was also kept constant.

### Radiolabeling Studies

All radiolabeling studies involving [^225^Ac]­Ac^3+^, [^161^Tb]­Tb^3+^, and [^177^Lu]­Lu^3+^ were performed using a standardized procedure, wherein stock solutions of each radionuclide (1 μL) were added to serial dilutions of each chelator (5 μL, 10^–3^ to 10^–8^ M) in NH_4_OAc buffer (44 μL, 0.2 M), to give a final total volume of 50 μL. For [^225^Ac]­Ac^3+^ radiolabeling studies, 20–25 kBq of activity was used in each reaction, with a final pH of 7.0. For [^161^Tb]­Tb^3+^ radiolabeling studies, 100 kBq of activity was used per reaction, with a final pH of 6.0. For [^177^Lu]­Lu^3+^ radiolabeling studies, 75 kBq of activity was used per reaction, with a final pH of 5.5–6.0. The pH was checked by spotting 0.3 μL of solution on thin-cut pH test strips. Radiolabeling reactions were allowed to stand at RT for 30 min, before spotting 2 μL of solution onto Aluminum-backed Silica TLC plates or SA-paper TLC strips. All radiolabeling studies involving H_4_ **dota** were heated at 85 °C for 30 min, and then allowed to cool for 5 min, before spotting on TLC plates. TLC plates were developed using sodium citrate (0.4 M, pH 4) or EDTA (50 mM, pH 5.5) as eluent; under these conditions, radiolabeled complexes remain bound at the baseline (*R*_f_ = 0–0.1) while free radionuclide ions migrate with the solvent front (*R*_f_ = 0.9–1.0). In the case of ^225^Ac, radio-TLC plates were scanned a minimum of 6 h after development, to allow for decay of free daughter radionuclides (^221^Fr, ^213^Bi) and attainment of secular equilibrium between ^225^Ac with its daughter radionuclides. The activity used in these experiments was quantified by gamma spectroscopy after reaching secular equilibrium with daughter radionuclides, using the key gamma emission lines from ^221^Fr (100, 218, 410 keV) and ^213^Bi (293, 440, 807 keV) for radionuclide identification and quantification.

### Human Serum Stability Assays

Serum stability experiments were undertaken by preparing high molar activity samples of each radiolabeled complex.

For actinium-225 experiments, [^225^Ac]­Ac^3+^ (300 kBq, 10 μL) was added to solutions of each chelate (3 nmol, 3 μL, 10^–3^ M) in NH_4_OAc buffer (89 μL). The reactions were allowed to stand at RT for 30 min, after which quantitative RCY was confirmed using radio-TLC analysis. For actinium-225, radio-TLC analysis was carried out by cutting the developed TLC plates into two halves and quantifying the francium-221 content using HPGe gamma spectroscopy, TLCs were measured 30 min after development to allow for decay of free ^221^Fr. After confirmation of quantitative radiolabeling, 30 μL of each ^225^Ac-labeled chelate (*A*_M_ = 100 kBq/nmol) was diluted into 270 μL of pooled human serum, to give triplicates. The solutions were incubated at 37 °C and monitored by radio-TLC over 8 days.

For terbium-161, [^161^Tb]­Tb^3+^ (1 MBq, 3 μL) was added to solutions of each chelate (1 nmol, 1 μL, 10^–3^ M) in NH_4_OAc buffer (0.2 M, 96 μL). The reactions were allowed to stand at RT for 30 min. After confirmation of quantitative radiolabeling using radio-TLC, 30 μL aliquots of each ^161^Tb-labeled chelator (*A*_M_ = 1 MBq/nmol) were diluted into 270 μL of pooled human serum, to give triplicates. The solutions were incubated at 37 °C and monitored by radio-TLC over 6–7 days.

## Supplementary Material



## Notes

X-ray crystallographic data of H_4_ **tpapxd**·6H_2_O, {[La­(**tpaen**)]­Na­(H_2_O)_4_}_2_·6H_2_O {[La­(**tpaopd**)]­Na­(H_2_O)_4_}, {[La­(**tpaond**)]­Na·13.75H_2_O}, {[Tb­(**tpaopd**)]­K·3H_2_O}, {[Tb­(**tpaond**)]­K·9H_2_O}_∞_, {[Tb­(H_2_ **tpapxd**)­(H_2_O)]­Cl·4H_2_O}_∞_, and {[Lu­(**tpaopd)**]­K­(H_2_O)_*n*_}; NMR spectra (^1^H, ^13^C, DEPT, ^1^H–^1^H COSY, HSQC, HMBC) of chelators H_4_ **L** ^**1**^ to H_4_ **L** ^**8**^ and La­(III) and Lu­(III) complexes; HR-ESI-MS spectra of chelators H_4_ **L** ^**1**^ to H_4_ **L** ^**8**^ and La­(III), Tb­(III), and Lu­(III) complexes; and solution titration studies (PDF)

## Notes

This work is dedicated to Nacho Montesinos Rodriguez *in Memoriam*, who passed away while this project was underway, beloved son of Dr. Teresa Rodriguez-Blas. We researchers are unaware of how much time we steal from our children until we lose them. And then, that time can never be recovered; all we can do is mourn them and keep their memory alive as we continue with our daily work.
