# Supplementary material for: Challenging the Macrocycle Paradigm: Four-Arm, High-Denticity Acyclic Chelators for Radiopharmaceuticals Incorporating Actinium and Lanthanides
Source: Inorg Chem. 2026 Mar 17;65(12):6836–60. doi: 10.1021/acs.inorgchem.6c00216 (PMC13040536; doi:10.1021/acs.inorgchem.6c00216)
Supplement: Supplementary file 1 [file ic6c00216_si_001.pdf]

Supporting Information

Challenging the Macrocycle Paradigm: Four-Arm, High-Denticity Acyclic Chelators for Radiopharmaceuticals Incorporating Actinium and Lanthanides

Daniel Fernández-Pavón,[†] *Andrés de Blas,*[†] *María Martínez-Cabanas,*[†] *José L. Barriada,*[†]

Brian O. Patrick,[¬] *Chris Orvig,*[‡] *François Bénard,*[¥,⸙] *Hua Yang,*[§] *Luke Wharton,*[§,]*

María de Guadalupe Jaraquemada-Peláez[¥,]* *and Teresa Rodríguez-Blas*[†,]*

[†] Grupo METMED, Departamento de Química, Universidade da Coruña, Campus da Zapateira s/n 15071 A Coruña, Spain.

[¬] Department of Chemistry, University of British Columbia, Vancouver BC, V6T 1Z1, Canada.

[‡] Medicinal Inorganic Chemistry Group, Department of Chemistry, University of British Columbia, Vancouver BC, V6T 1Z1, Canada.

[¥] Department of Molecular Oncology, BC Cancer Research Institute, Vancouver, BC, V5Z 1L3, Canada.

[⸙] Department of Radiology, University of British Columbia, Vancouver, BC V5Z 1M9, Canada.

[§] Life Sciences Division, TRIUMF, 4004 Wesbrook Mall, Vancouver BC, V6T 2A3, Canada.

Corresponding Authors:

Teresa Rodríguez-Blas – E-mail: teresa.rodriguez.blas@udc.es

María de Guadalupe Jaraquemada-Peláez – E-mail: mpelaez@bccrc.ca

Luke Wharton – E-mail: lwharton@triumf.ca

Table of Contents

X-ray Crystallography (Tables S1 to S3) **S4**

NMR Spectra of chelators and precursors **S7**

NMR spectra of H_4**tpaen** (H_4**L**^{1}) (Figures S1 to S5) S7

NMR spectra of H_4**tpaopd** (H_4**L**^{2}) (Figures S5 to S10) S10

NMR spectra spectra of H_4**tpaond** (H_4**L**^{3}) at pD=12 (Figures S10 to S15) S13

NMR spectra of H_4**tpamxd** (H_4**L**^{4}) (Figures S16 to S20) S16

NMR spectra of H_4**tpapxd** (H_4**L**^{5}) (Figures S21 to S25) S19

NMR spectra of H_4**tpadapo** (H_4**L**^{6}) (Figures S26 to S30) S22

NMR spectra of compound **(2)** (Figure S31) S25

NMR spectra of compound **(3)** (Figure S32) S26

NMR spectra of H_3**tripaen** (H_3**L**^{7}) (Figures S33 to S37) S27

NMR spectra of compound **(5)** (Figure S38). S30

NMR spectra of H_4**asyoctapa** (H_4**L**^{8}) (Figures S39 to S43) S31

NMR Spectra of lanthanum(III) complexes **S34**

NMR spectra of [La(**tpaen**)]$^{-}$ (Figures S44 to S49) S34

NMR spectra of [La(**tpaopd**)]$^{-}$ (Figures S50 to S55) S37

NMR spectra of [La(**tpaond**)]$^{-}$ (Figures S56 to S61) S40

NMR spectra of the lanthanum(III) complex with H_4**tpamxd** (Figure S62). S43

NMR spectra of the lanthanum(III) complex with H_3**tripaen** (Figure S63). S44

NMR spectra of [La(**asyoctapa**)]$^{-}$ (Figures S64 to S69) S45

NMR Spectra of lutetium(III) complexes **S48**

NMR spectra of [Lu(**tpaen**)]$^{-}$ (Figures S70) S48

NMR spectra of [Lu(**tpaopd**)]$^{-}$ (Figures S71 to S76) S49

NMR spectra of [Lu(**tpaond**)]$^{-}$ (Figures S77 to S82) S52

NMR spectra of the lutetium(III) complex with H_4**tpamxd** (Figures S83 to S85). . S55

NMR spectra of the lutetium(III) complex with H_4**tpapxd** (Figures S86 to S91) ... S57

NMR spectra of the lutetium(III) complex with H_3**tripaen** (Figure S92) S60

NMR spectra of [Lu(**asyoctapa**)]$^{-}$ (Figures S93 to S98) S61

Tables with NMR chemical shift assignments
(Tables S4 to S9) **S64**

Mass Spectrometry of compounds (2), (3), (5) and chelators H_4L^{1} to H_4L^{8}
(Figures S99 to S109) **S70**

Mass Spectrometry of cold lanthanum(III) complexes
(Figures S110 to S115) **S80**

Mass Spectrometry of cold terbium(III) complexes
(Figures S116 to S120).. **S86**

Mass Spectrometry of cold lutetium (III) complexes
(Figures S121 to S126).. **S91**

Solution Thermodynamics
(Figure S127)... **S97**

X-ray Crystallography

Table S1. Selected crystallographic data for chelators and lanthanide(III) complexes.

Data	H_4**tpapxd**·$6H_2O$	{[La(**tpaen**)]Na(H_2O)$_4$}$_2$·$6H_2O$	{[La(**tpaopd**)]Na(H_2O)$_4$}	{[La(**tpaond**)]Na·13.75H_2O}
**Empirical formula**	$C_{36}H_{44}N_6O_4$	$C_{60}H_{92}La_2N_{12}Na_2O_{38}$	$C_{34}H_{32}LaN_6NaO_{12}$	$C_{152}H_{142}La_4N_{24}Na_4O_{55}$
**Formula weight**	784.77	1913.25	878.55	3832.49
**Colour**	colourless	colourless	colourless	colourless
**Temperature/K**	100(2)	100(2)	100(2)	100(2)
**Crystal system**	triclinic	monoclinic	monoclinic	monoclinic
**Space group**	P-1	P 21/c	C 2/c	P 21/c
**a/Å**	8.0230(6)	11.7340(3)	15.3894(7)	12.3408(5)
**b/Å**	8.7148(6)	14.5208(4)	18.2117(8)	22.4593(10)
**c/Å**	13.7912(10)	22.8949(6)	12.3988(5)	15.1505(7)
**α/°**	99.1520(10)	90	90	90
**β/°**	91.2250(10)	101.9170(10)	104.287(2)	102.605(2)
**γ/°**	102.9520(10)	90	90	90
**Volume/Å^3**	926.14(12)	3816.92(18)	3367.5(3)	4098.0(3)
**Z**	1	2	4	1
**ρ_{calc} g/cm^3**	1.407	1.665	1.733	1.553
**μ/mm^{-1}**	0.109	1.218	1.358	1.126
**F(000)**	414	1952	1768	1934
**Crystal size/mm^3**	055×0.35×0.12	0.133x0.094x0.082	0.070x0.040x0.020	0.040x0.030x0.020
**Θ range for data collection/°**	1.498 to 30.533	2.261 to 28.295	2.237 to 27.102	2.136 to 25.682
**Index ranges**	-11<=h<=11, -12<=k<=12, 0<=l<=19	-14<=h<=15, -19<=k<=19, -30<=l<=30	-19<=h<=19, -23<=k<=23, -15<=l<=15	-15<=h<=13, -27<=k<=27, -18<=l<=18
**Measured Reflections**	5647	98786	64644	107239
**Independent reflections**	5647	9470	3715	7772
**Data/restraints/parameters**	5647/0/285	9470/33/576	3715/6/257	7772/55/639
**Goodness-of-fit on F^2**	1.076	1.059	1.090	1.034
**Final R indexes [I>=2σ (I)]**	0.0378	0.0251	0.0326	0.0440
**Final R indexes [all data]**	0.0441	0.0288	0.0395	0.0627
***wR_2* (all data)**	0.1079	0.0618	0.0833	0.1255
wR_2	0.1044	0.0592	0.0789	0.1126
**Largest diff. peak/hole / e Å^{-3}**	0.473/-0.197	0.881/-0.621	1.740/-0.713	1.210/-1.562

Table S2. Selected crystallographic data for terbium(III) and lutetium(III) complexes.

Data	{[Tb(**tpaopd**)]K·3H_2O}	{[Tb(**tpaond**)]K·9H_2O}$_\infty$	{[Tb(H_2**tpapxd**)(H_2O)]Cl·4H_2O}$_\infty$	{[Lu(**tpaopd**)]K(H_2O)$_n$}
**Empirical formula**	$C_{34}H_{30}KN_6O_{11}Tb$	$C_{76}H_{82}K_2N_{12}O_{31}Tb_2$	$C_{72}H_{80}Cl_2N_{12}O_{26}Tb_2$	$C_{34}H_{24}K_0N_6O_8$
**Formula weight**	896.66	2055.57	1918.22	819.56
**Colour**	colourles	colouless	colourless	colourless
**Temperature/K**	293(2)	100(2)	100(2)	100(2)
**Crystal system**	monoclinic	monoclinic	triclinic	monoclinic
**Space group**	C 2/c	C 2/c	P-1	C 2/c
**a/Å**	14.82110(10)	14.9763(10)	11.6329(5)	14.5701(10)
**b/Å**	21.39590(10)	22.5245(15)	12.4689(6)	21.3012(15)
**c/Å**	12.03260(10)	12.1934(7)	14.9586(7)	11.9454(8)
**α/°**	90	90	65.652(2)	90
**β/°**	101.7780(10)	102.777(2)	69.475(2)	102.367(2)
**γ/°**	90	90	75.843(2)	90
**Volume/Å^3**	3735.33(5)	4011.4(4)	1838.51(15)	3621.4(4)
**Z**	4	2	1	4
**ρ_{calc} g/cm^3**	1.594	1.702	1.733	1.503
**μ/mm^{-1}**	10.875	1.043	1.103	1.500
**F(000)**	1792	2076	968	1620
**Crystal size/mm^3**	0.210×0.120×0.040	0.100×0.050×0.010	0.08×0.06×0.04	0.08×0.06×0.010
**Θ range for data collection/°**	4.132 to 70.051	1.965 to 20.518	2.175 to 23.699	2.043 to 21.956
**Index ranges**	-15<=h<=18, -26<=k<=25, -14<=l<=14	-18<=h<=18, -28<=k<=28, -15<=l<=15	-16<h<-16, -17<k<17, -21<l<21	-19<h<-19, -28<k<28, -15<l<15
**Measured Reflections**	75945	49671	143715	60355
**Independent reflections**	3511	4104	11277	4497
**Data/restraints/parameters**	3511/18/296	4104/38/359	11277/0/552	4497/0/222
**Goodness-of-fit on F^2**	1.094	1.069	1.105	1.077
**Final R indexes [I>=2σ (I)]**	0.0347	0.0343	0.0360	0.0383
**Final R indexes [all data]**	0.0353	0.0415	0.0420	0.0438
***wR_2* (all data)**	0.1102	0.0734	0.0948	0.0868
wR_2	0.1096	0.0772	0.0894	0.0842
**Largest diff. peak/hole / e Å^{-3}**	0.898/-0.467	1.165/-1.083	3.792/-0.613	1.126/-3.450

Table S3. Hydrogen bond information for H_4**tpapxd**·$6H_2O$.

**D**	**H**	**A**	**d(D-H)/Å**	**d(H-A)/Å**	**d(D-A)/Å**	**D-H-A/deg**
O1	H1	O3[1]	1.10(2)	1.37(2)	2.4685(9)	172.8(19)
N2	H2	O5	0.920(15)	1.952(15)	2.8531(10)	165.9(13)
O6	H6A	O4[4]	0.867(18)	1.883(18)	2.7499(11)	178.4(16)
O6	H6B	O5	0.883(19)	1.887(19)	2.7580(11)	168.6(17)
O7	H7C	O6	0.930(19)	1.927(19)	2.8530(11)	174.3(16)
O5	H5A	O1	0.856(17)	2.218(16)	2.9748(10)	147.3(14)
O5	H5A	N1	0.856(17)	2.301(16)	2.9360(11)	131.2(14)
O7	H7D	O2[1]	0.888(19)	2.06(2)	2.9021(10)	158.1(17)
O5	H5B	O3	0.879(18)	1.945(18)	2.8139(10)	169.2(16)

[1]1-x,2-y,1-z; [2]1-x,1-y,1-z; [3]1-x,2-y,2-z; [4]-1+x,+y,+z

NMR Spectra of chelators and precursors

NMR spectra of H_4**tpaen** (H_4**L**^{1}).

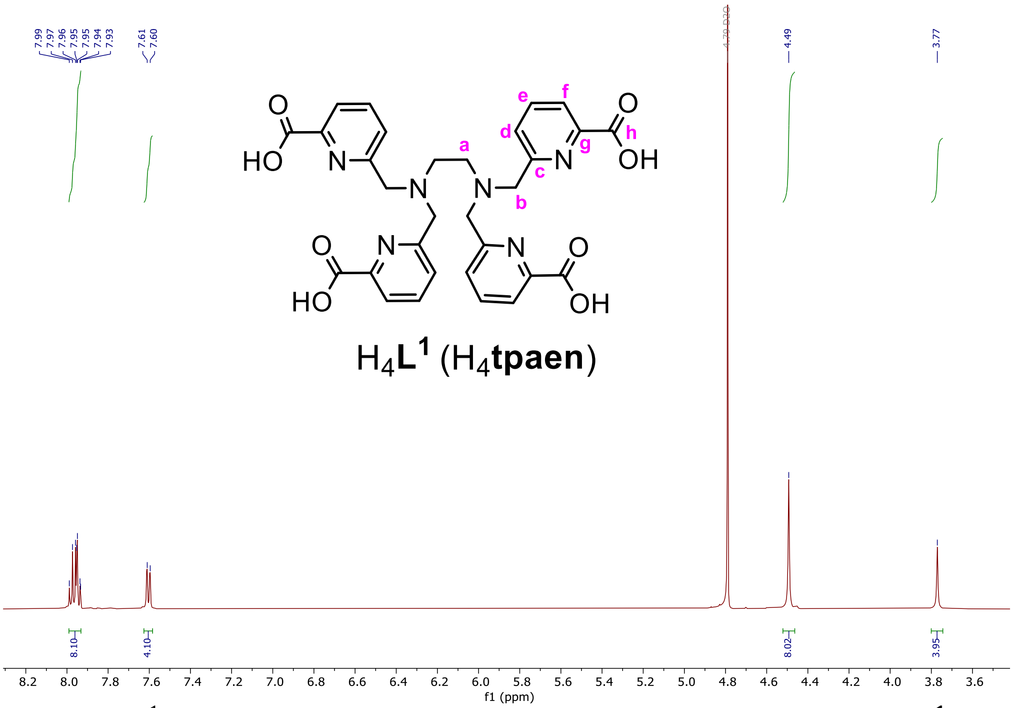

Figure S1. ^{1}H NMR spectrum (500 MHz, 298 K, D_2O) of H_4**tpaen** (H_4**L**^{1}).

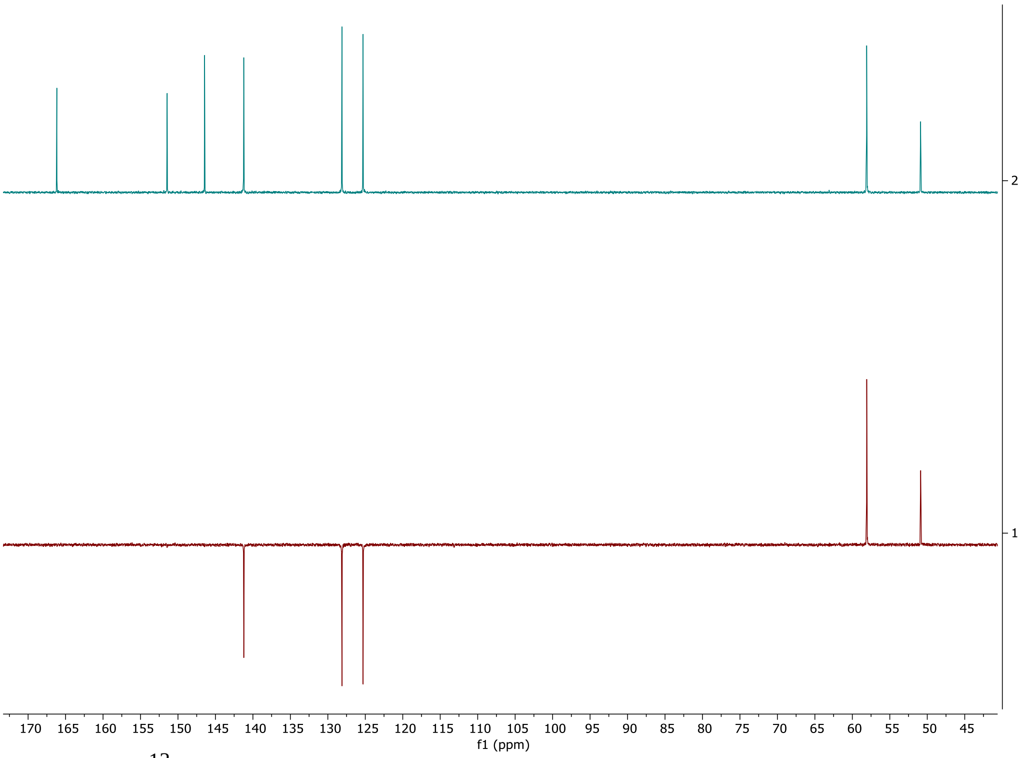

Figure S2. ^{13}C NMR and DEPT spectra (126 MHz, 298 K, D_2O) of H_4**tpaen** (H_4**L**^{1}).

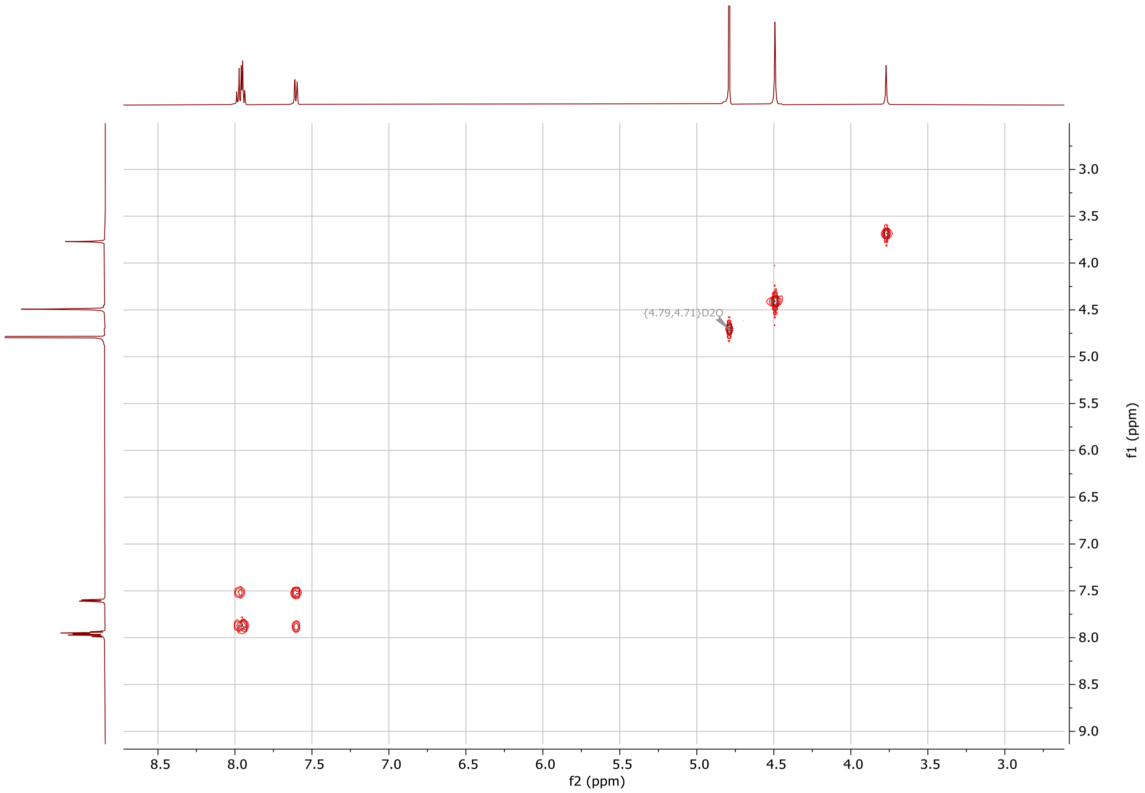

Figure S3. ^{1}H-^{1}H COSY NMR spectrum (500 MHz, 298 K, D_2O) of H_4**tpaen** (H_4**L**^{1}).

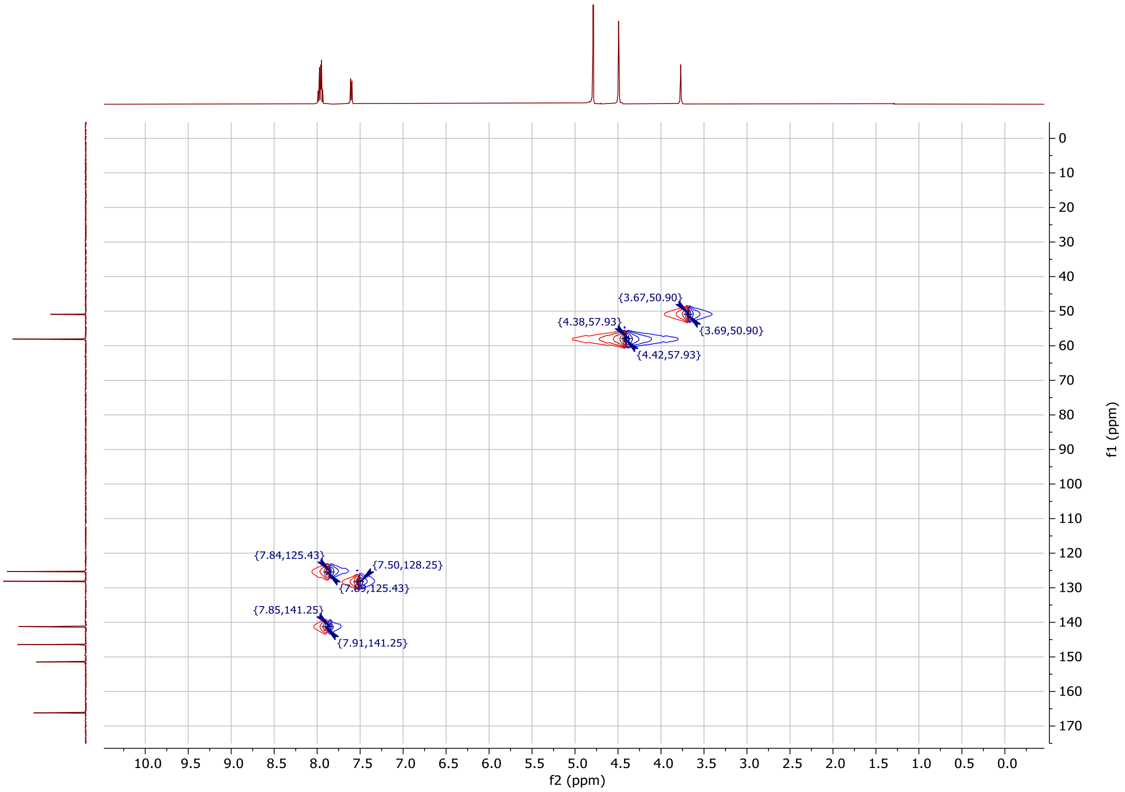

Figure S4. ^{1}H-^{13}C HSQC NMR spectrum (500 MHz, 298 K, D_2O) of H_4**tpaen** (H_4**L**^{1}).

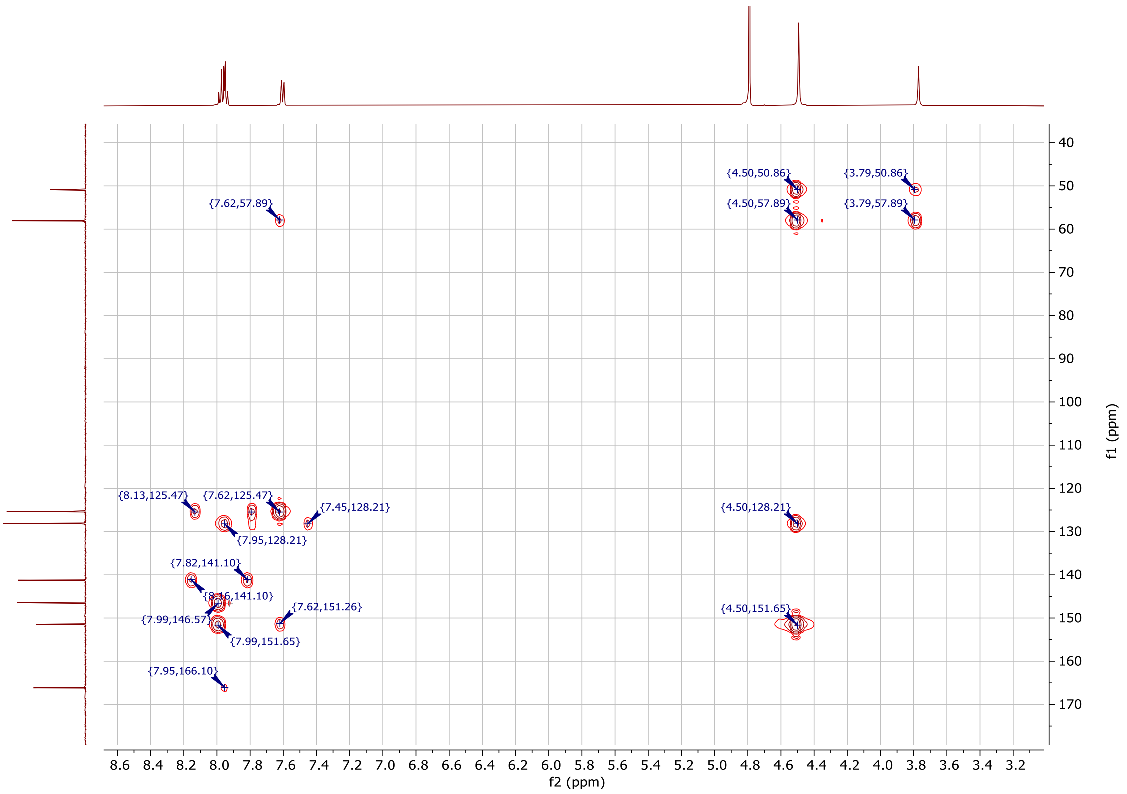

Figure S5. ^{1}H-^{13}C HMBC NMR spectrum (500 MHz, 298 K, D_2O) of H_4**tpaen** (H_4**L**$^{\mathbf{1}}$).

NMR spectra of H_4**tpaopd** (H_4**L**^{2}).

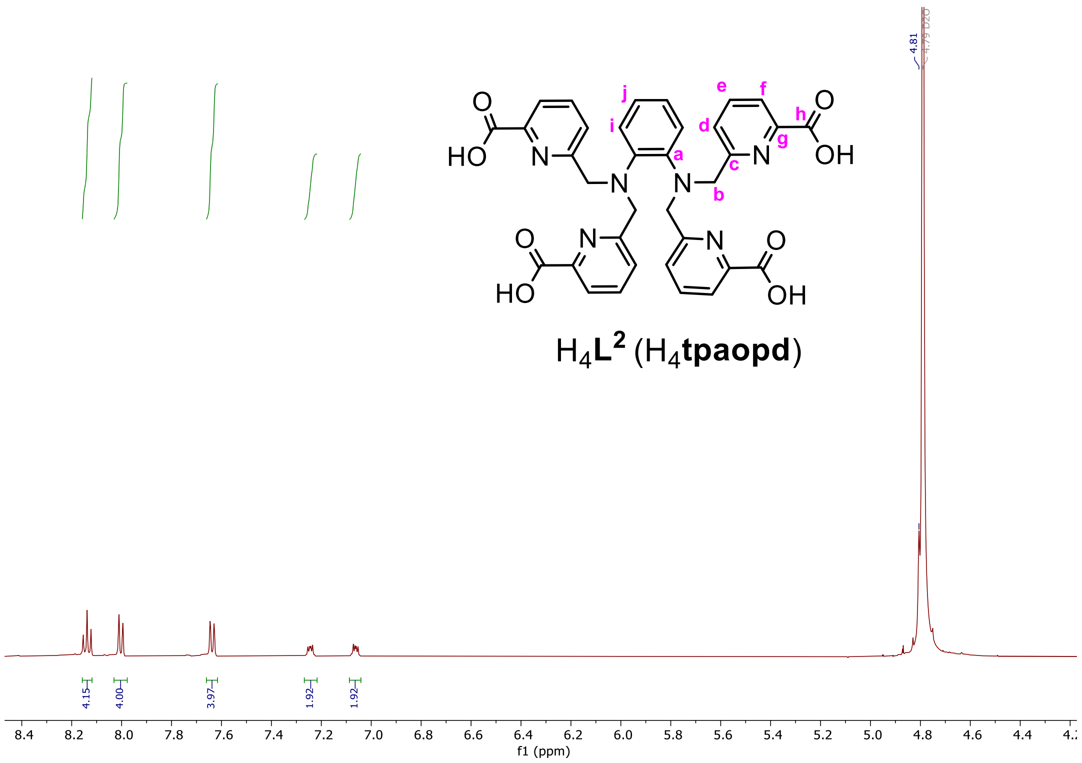

Figure S6. ^{1}H NMR spectrum (500 MHz, 298 K, D_2O) of H_4**tpaopd** (H_4**L**^{2}).

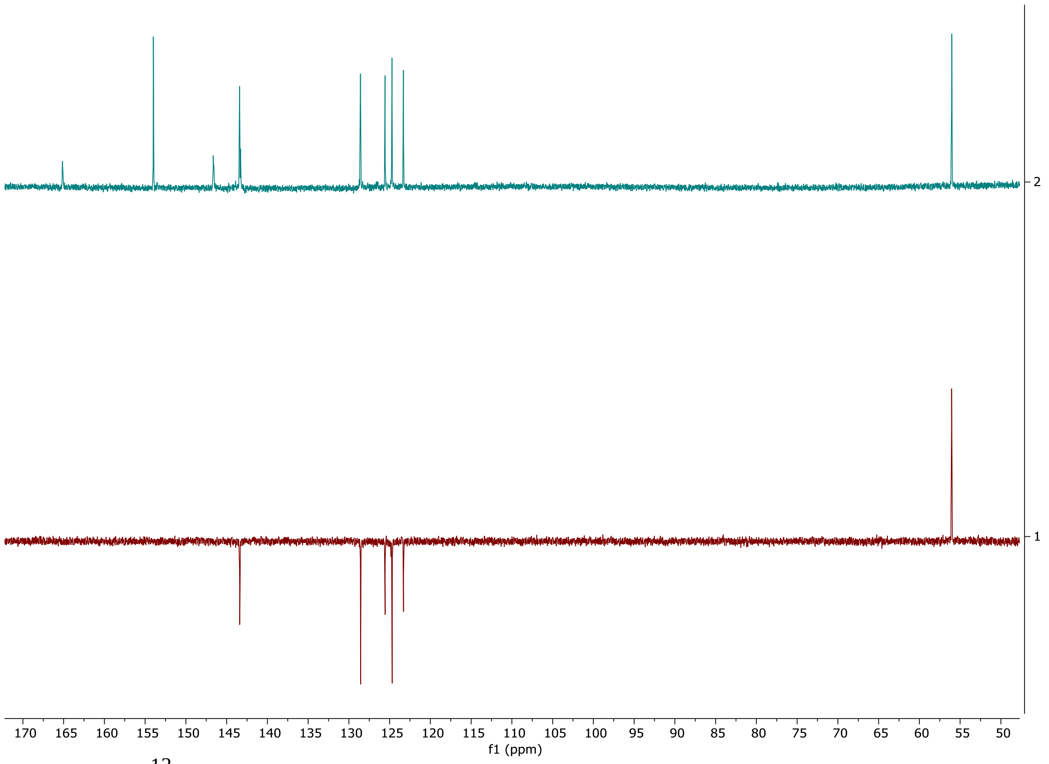

Figure S7. ^{13}C NMR and DEPT spectra (126 MHz, 298 K, D_2O) of H_4**tpaopd** (H_4**L**^{2}).

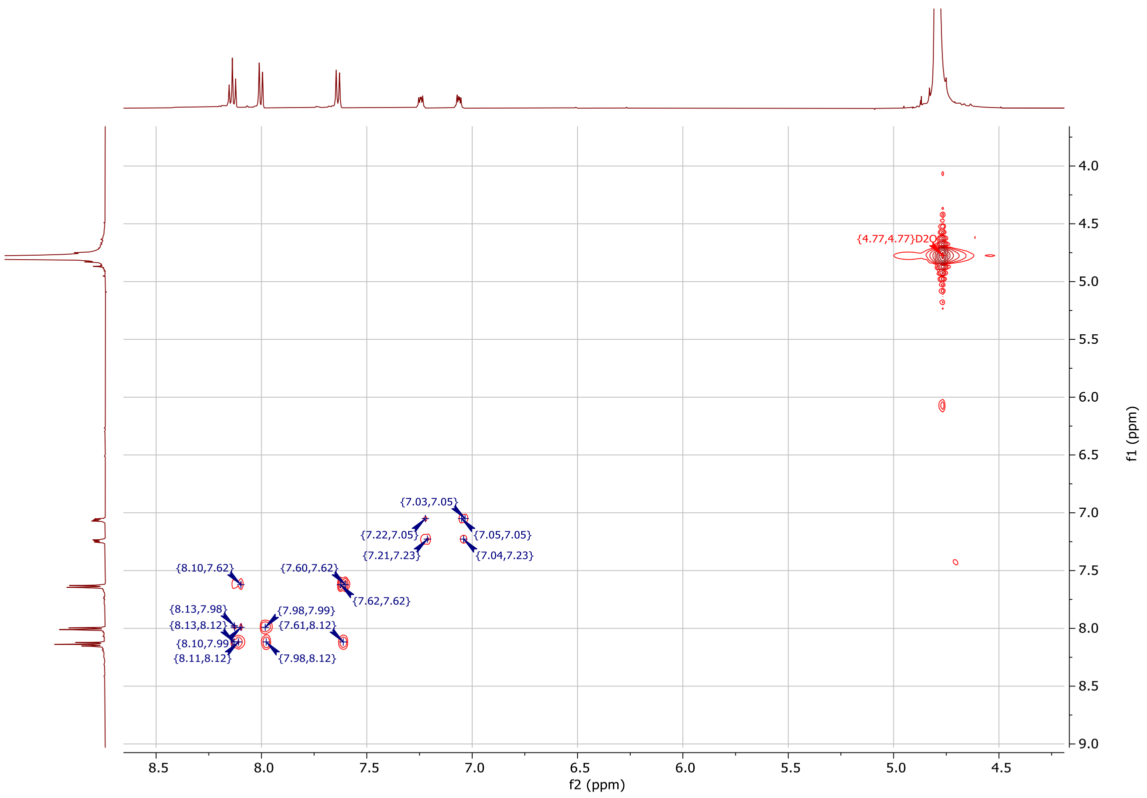

Figure S8. ^{1}H-^{1}H COSY NMR spectrum (500 MHz, 298 K, D_2O) of H_4**tpaopd** (H_4**L**^{2}).

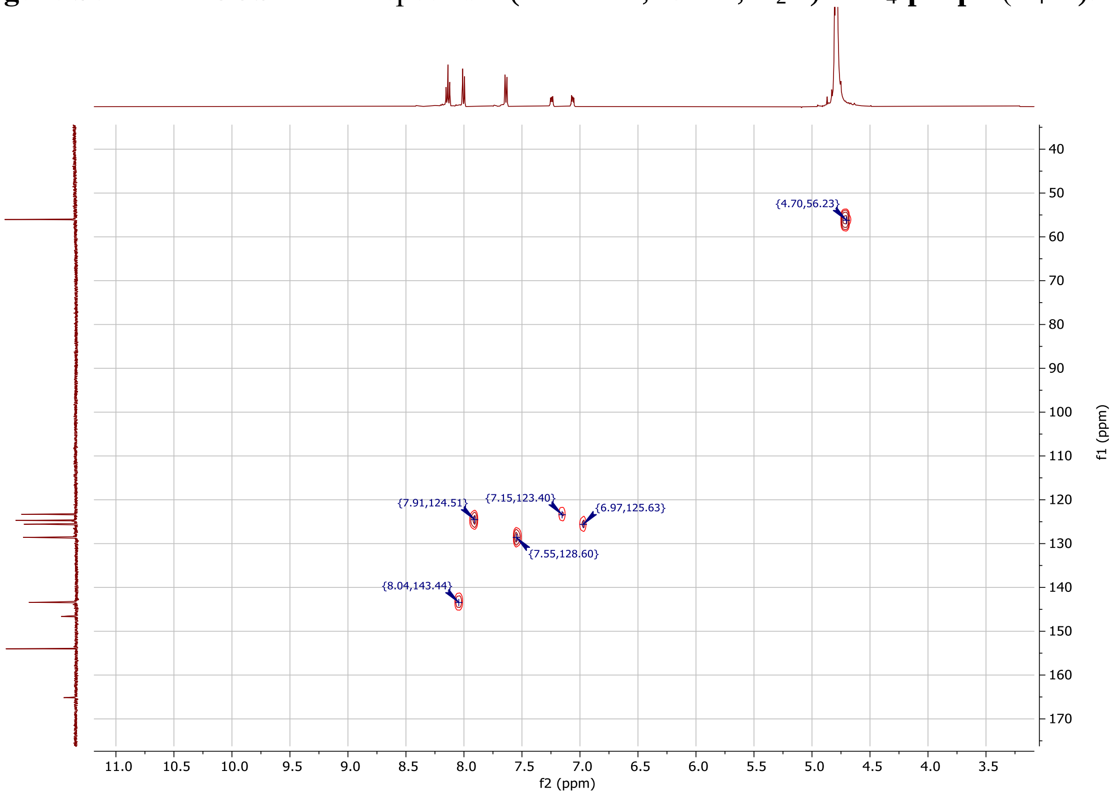

Figure S9. ^{1}H-^{13}C HSQC NMR spectrum (500 MHz, 298 K, D_2O) of H_4**tpaopd** (H_4**L**^{2}).

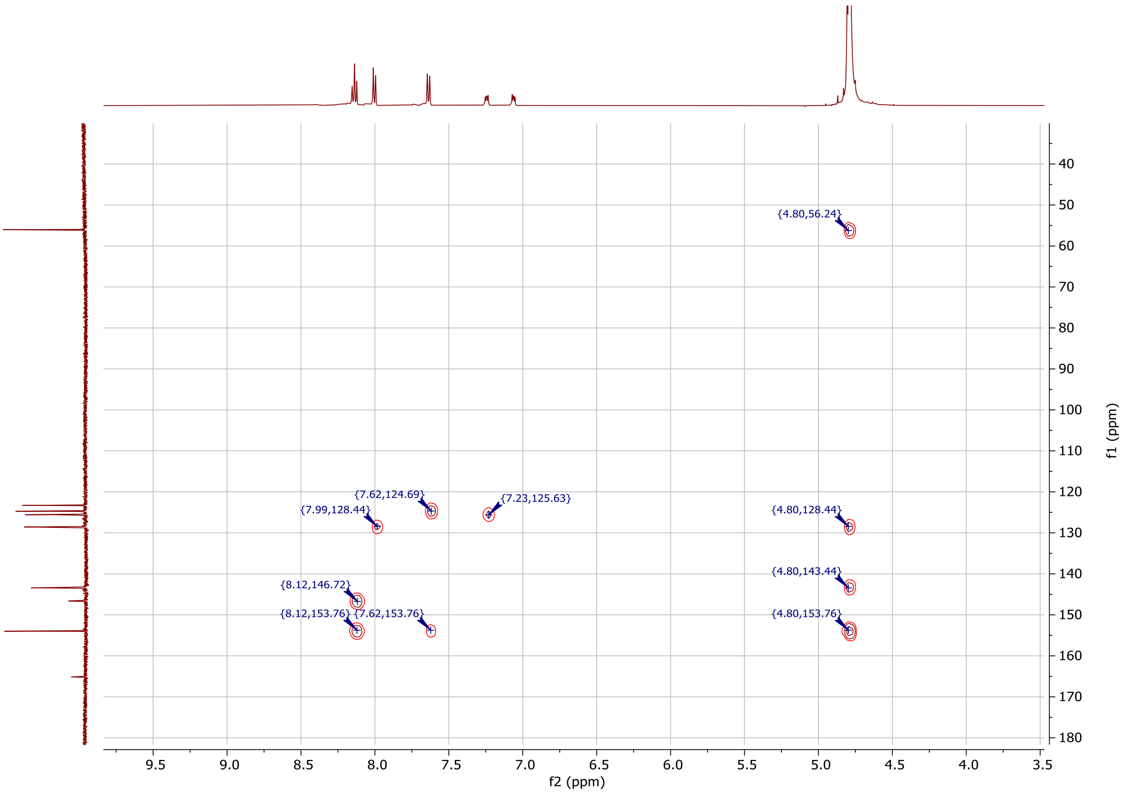

Figure S10. ^{1}H-^{13}C HMBC NMR spectrum (500 MHz, 298 K, D_2O) of H_4**tpaopd** (H_4**L**$^{\mathbf{2}}$).

NMR spectra spectra of H_4**tpaond** (H_4**L**^{3}) at pD=12.

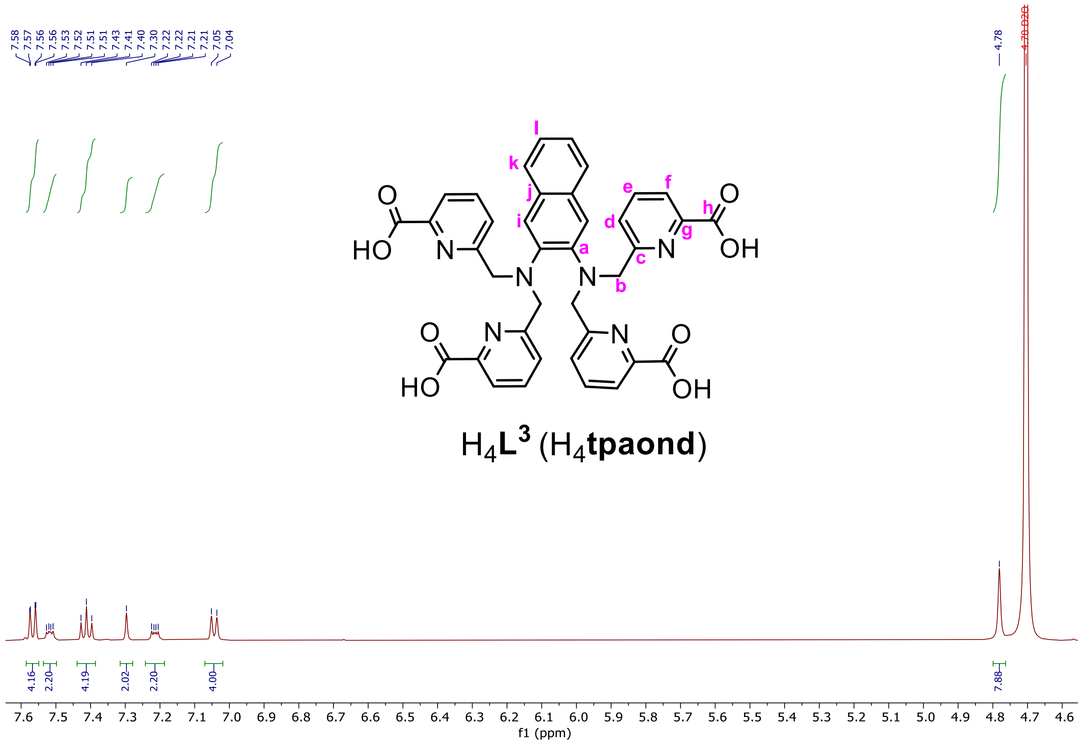

Figure S11. ^{1}H NMR spectrum (500 MHz, 298 K, D_2O) of H_4**tpaond** (H_4**L**^{3}) at pD = 12.

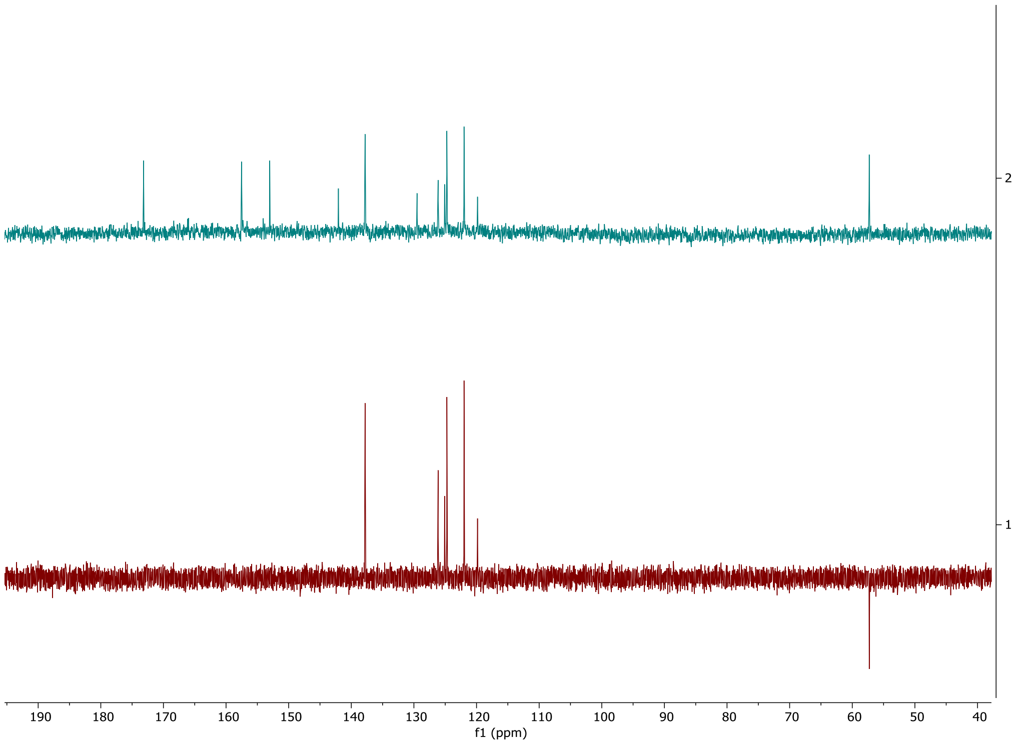

Figure S12. ^{13}C NMR and DEPT spectra (126 MHz, 298 K, D_2O) of H_4**tpaond** (H_4**L**^{3}) at pD = 12.

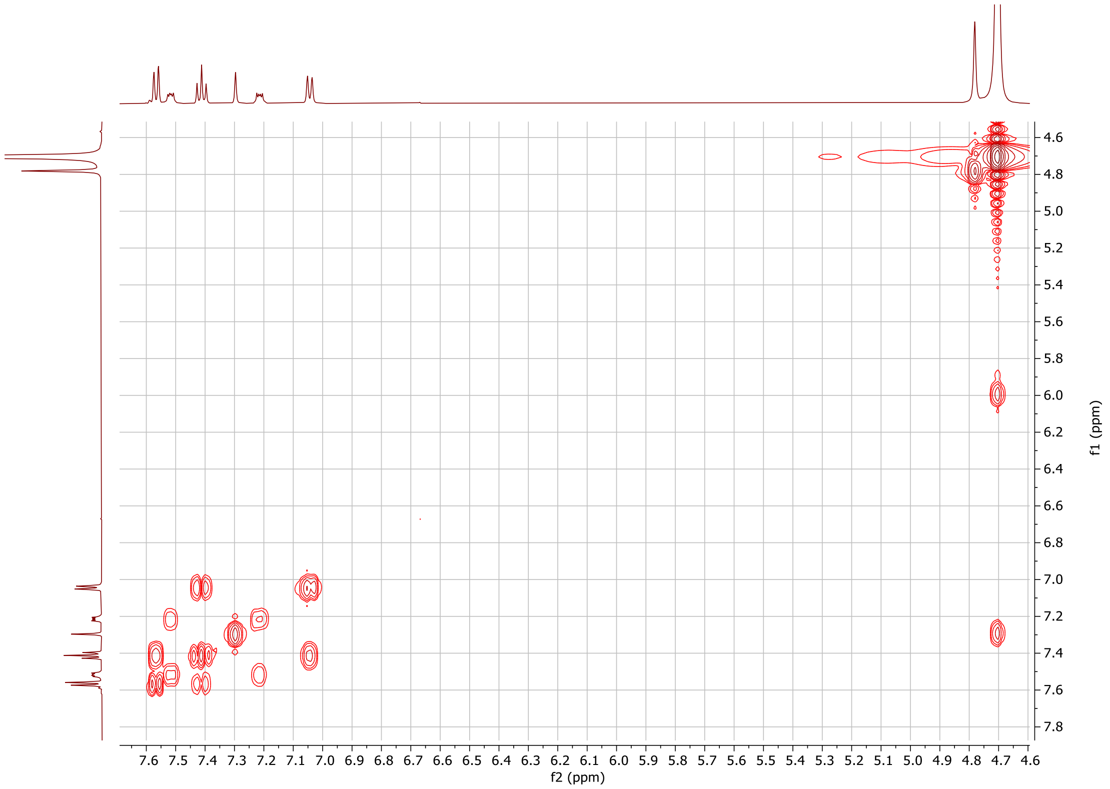

Figure S13. ^{1}H-^{1}H COSY NMR spectrum (500 MHz, 298 K, D_2O) of H_4**tpaond** (H_4**L**^{3}) at pD = 12.

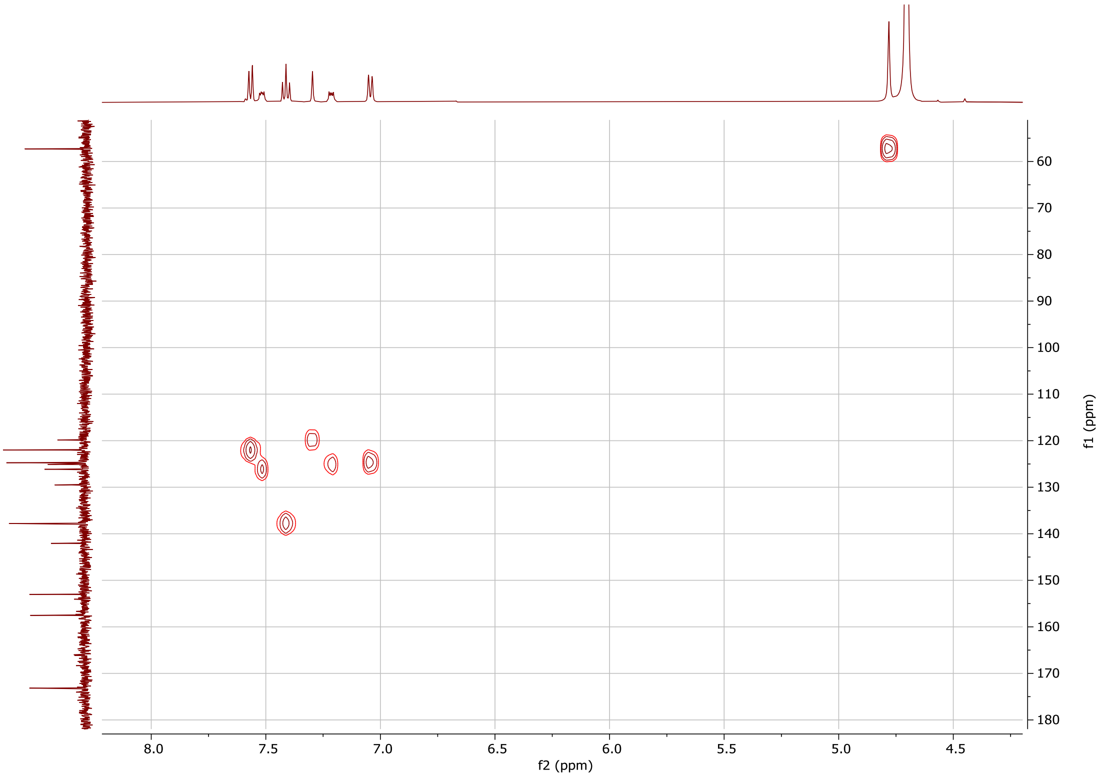

Figure S14. ^{1}H-^{13}C HSQC NMR spectrum (500 MHz, 298 K, D_2O) of H_4**tpaond** (H_4**L**^{3}) at pD = 12.

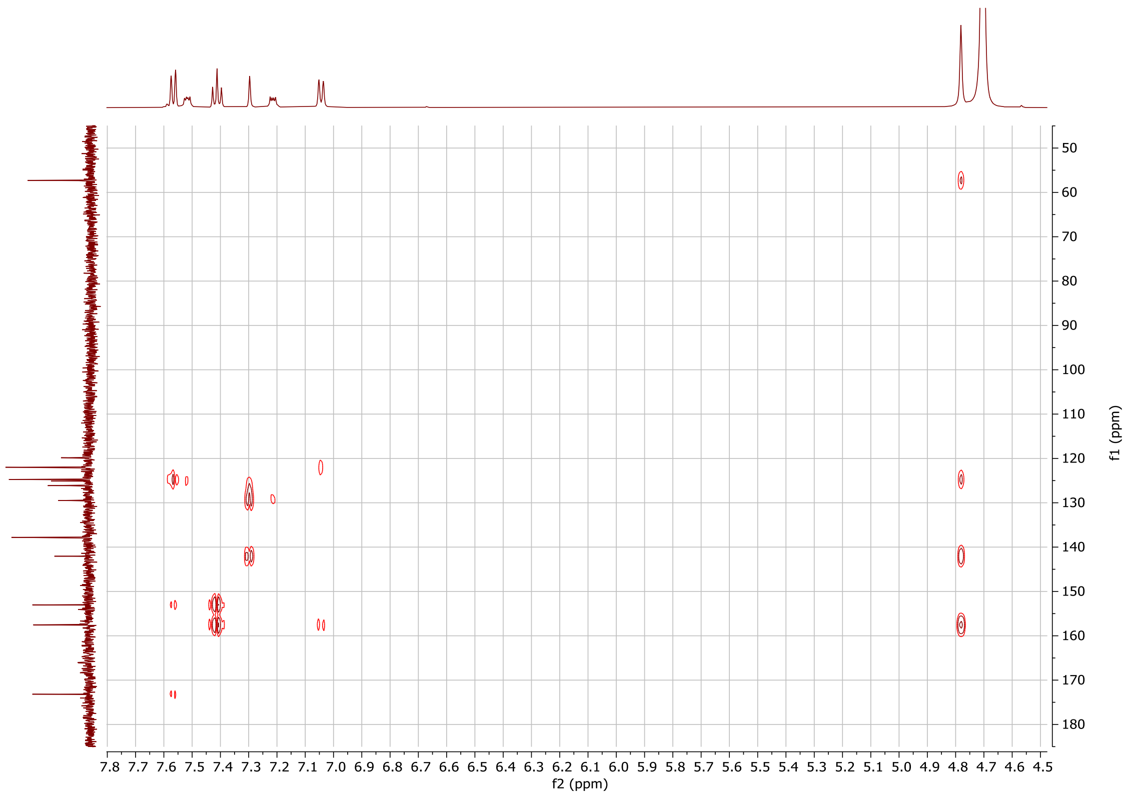

Figure S15. ^{1}H-^{13}C HMBC NMR spectrum (500 MHz, 298 K, D_2O) of H_4**tpaond** (H_4**L**$^{\mathbf{3}}$) at pD = 12.

NMR spectra of H$_4$**tpamxd** (H$_4$**L**^{4}).

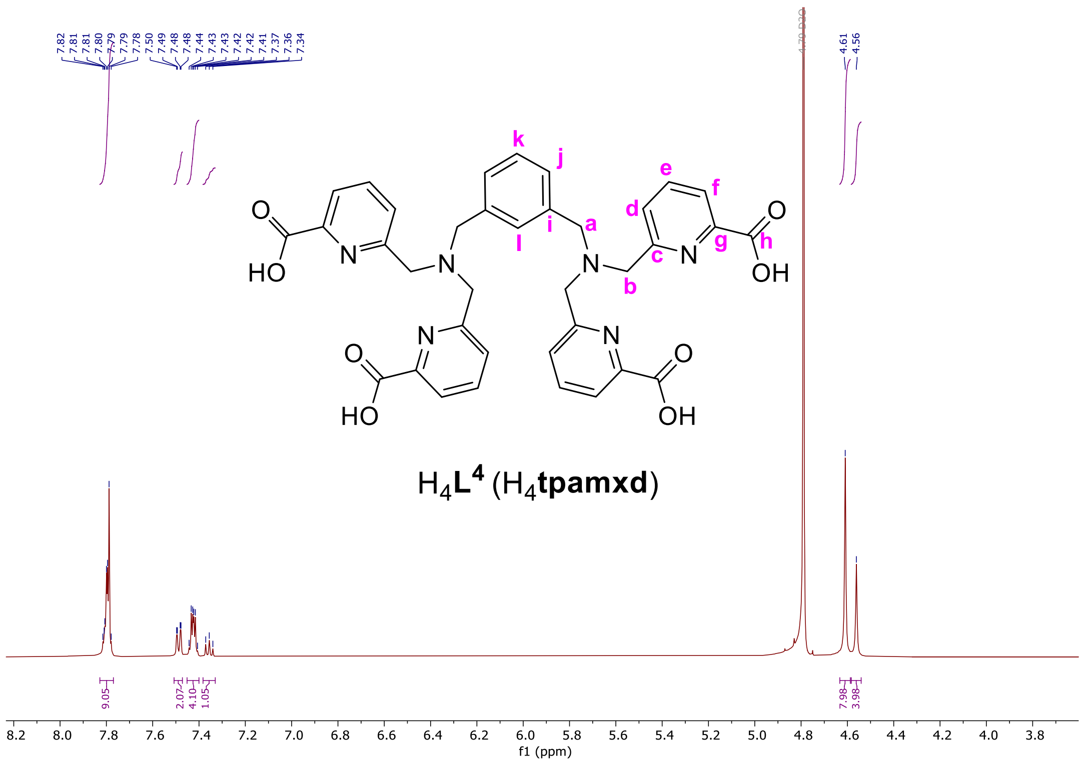

Figure S16. ^{1}H NMR spectrum (500 MHz, 298 K, D$_2$O) of H$_4$**tpamxd** (H$_4$**L**^{4}).

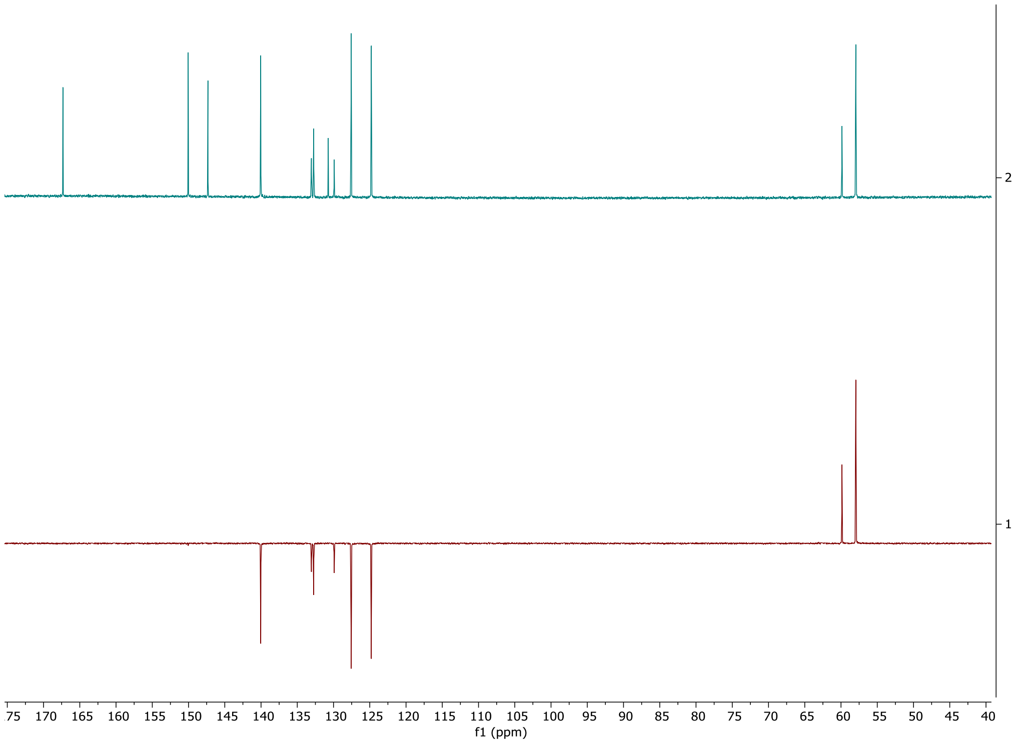

Figure S17. ^{13}C NMR and DEPT spectra (126 MHz, 298 K, D$_2$O) of H$_4$**tpamxd** (H$_4$**L**^{4}).

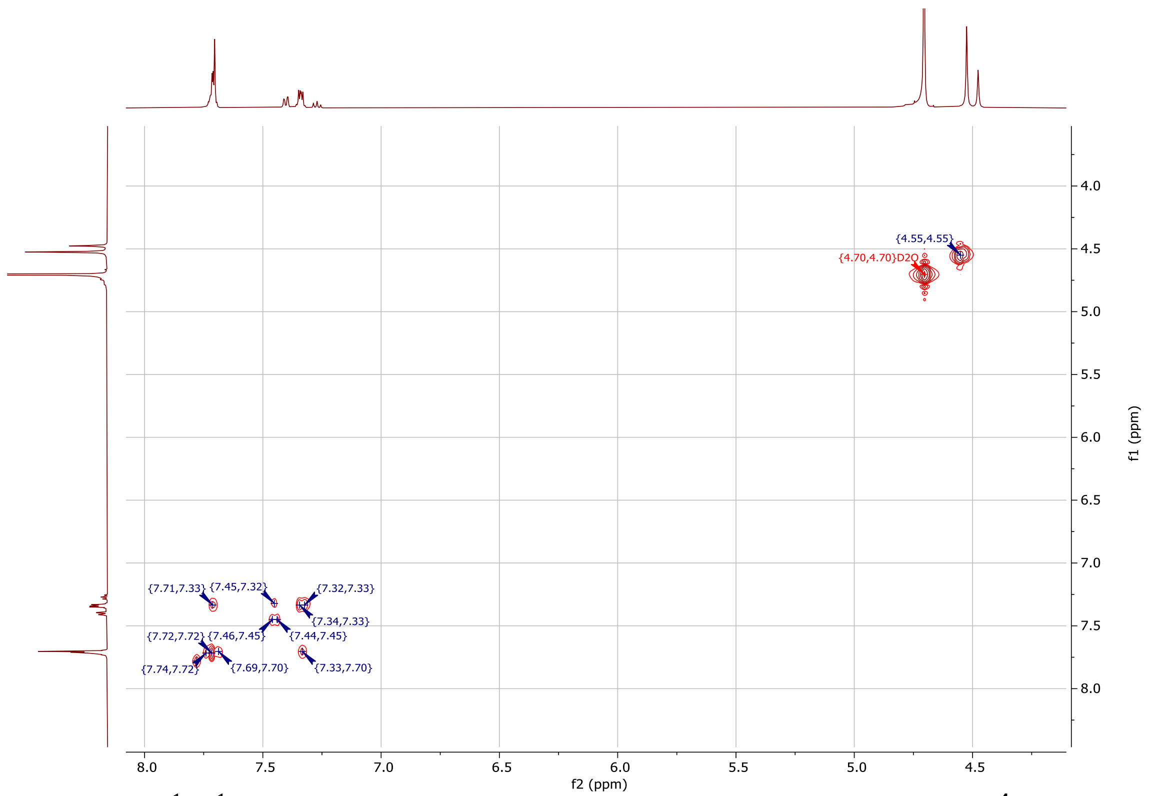

Figure S18. ^{1}H-^{1}H COSY NMR (500 MHz, 298 K, D_2O) of H_4**tpamxd** (H_4**L**^{4}).

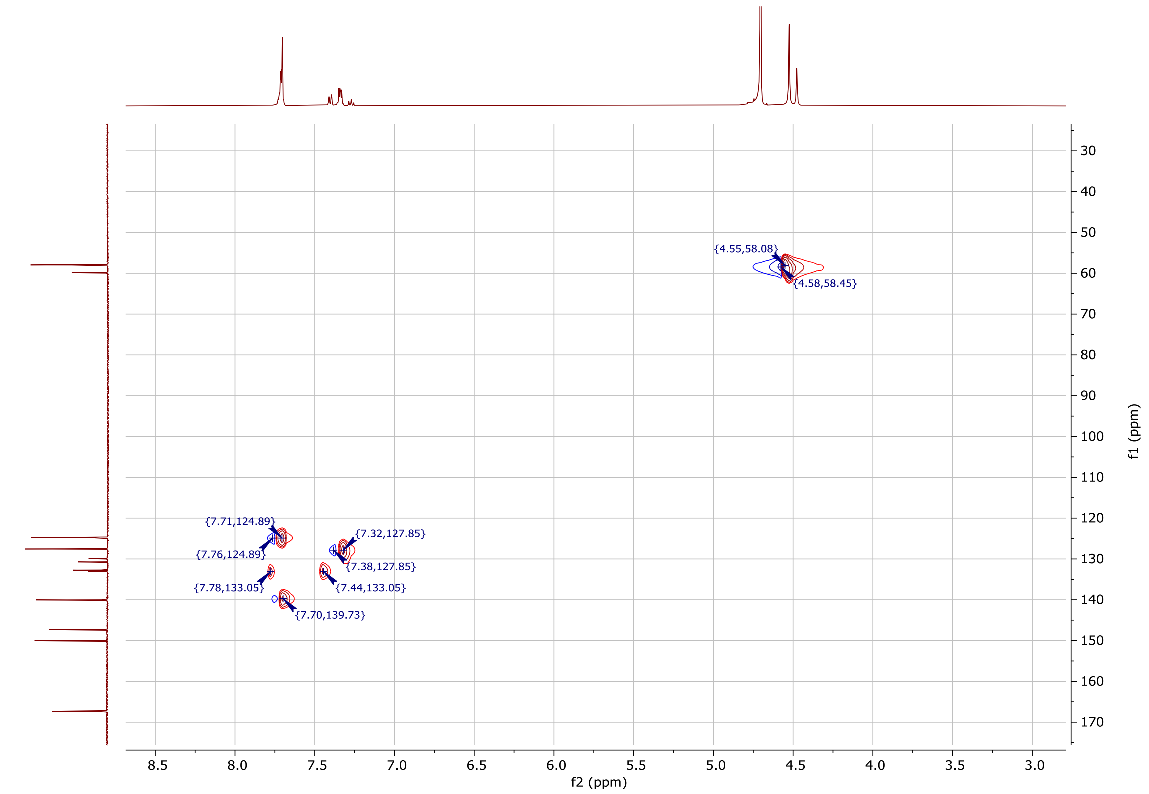

Figure S19. ^{1}H-^{13}C HSQC NMR spectrum (500 MHz, 298 K, D_2O) of H_4**tpamxd** (H_4**L**^{4}).

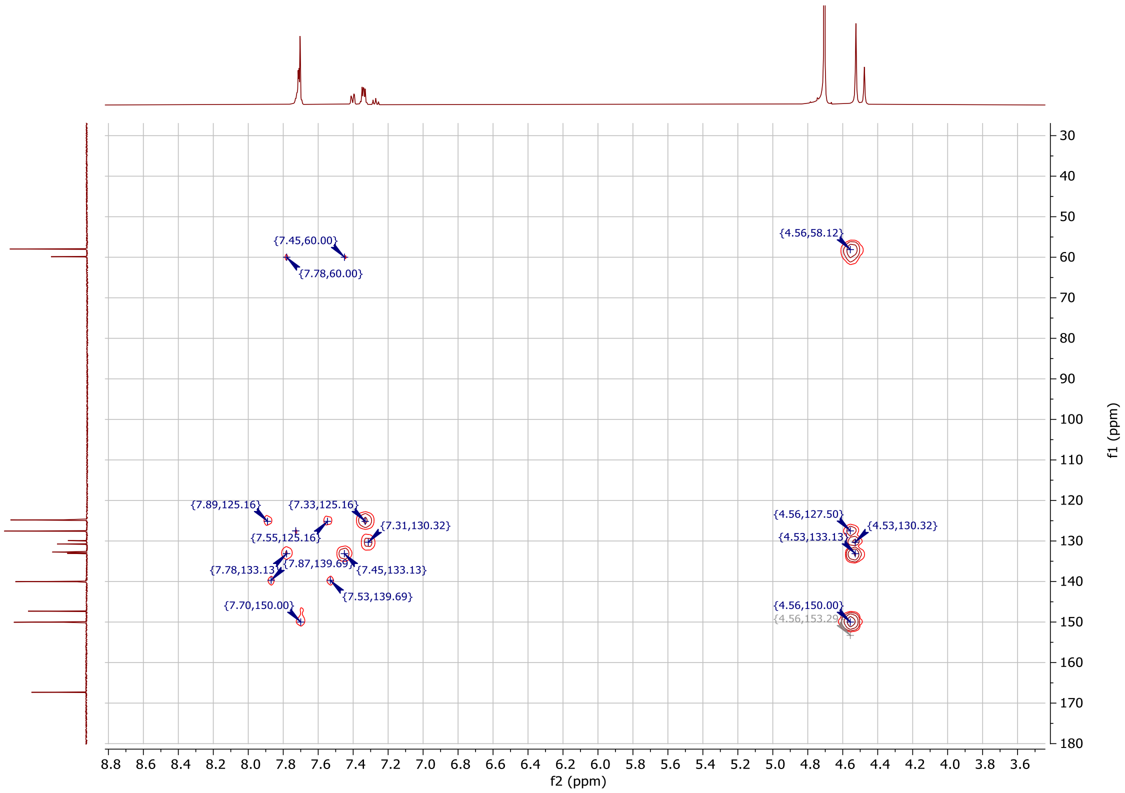

Figure S20. ^{1}H-^{13}C HMBC NMR spectrum (500 MHz, 298 K, D_2O) of H_4**tpamxd** (H_4**L^4**).

NMR spectra of H_4**tpapxd** (H_4**L**^{5}).

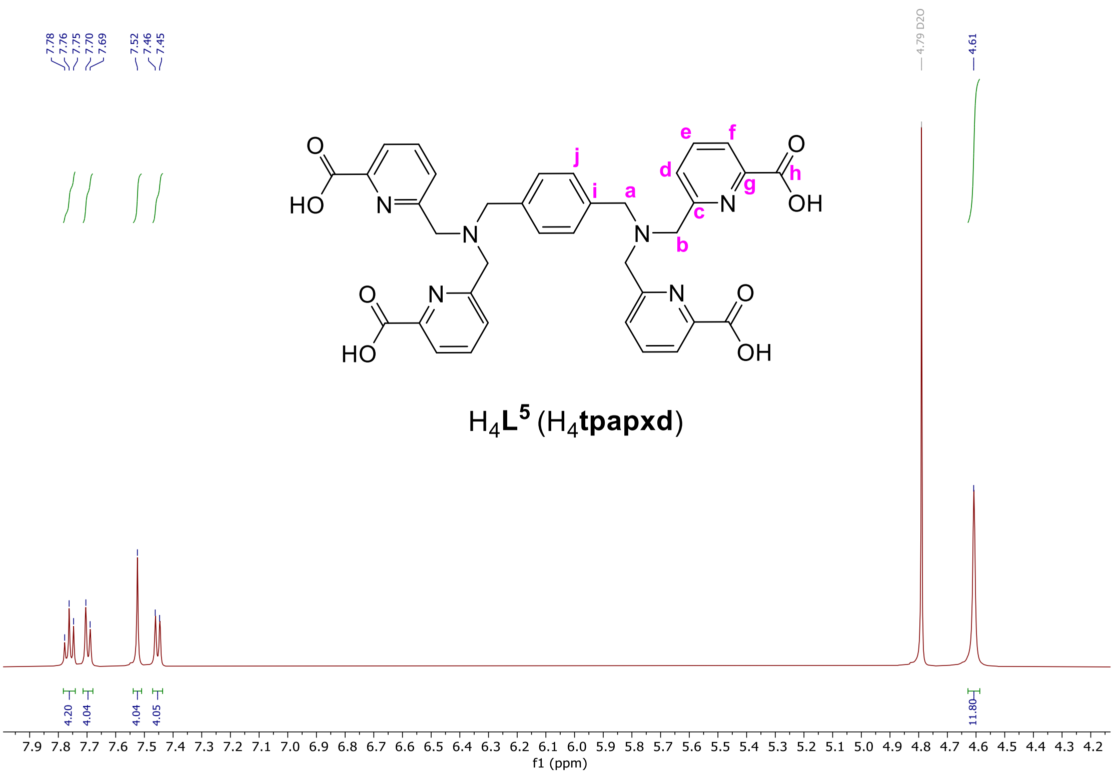

Figure S21. ^{1}H NMR spectrum (500 MHz, 298 K, D_2O) of H_4**tpapxd** (H_4**L**^{5}).

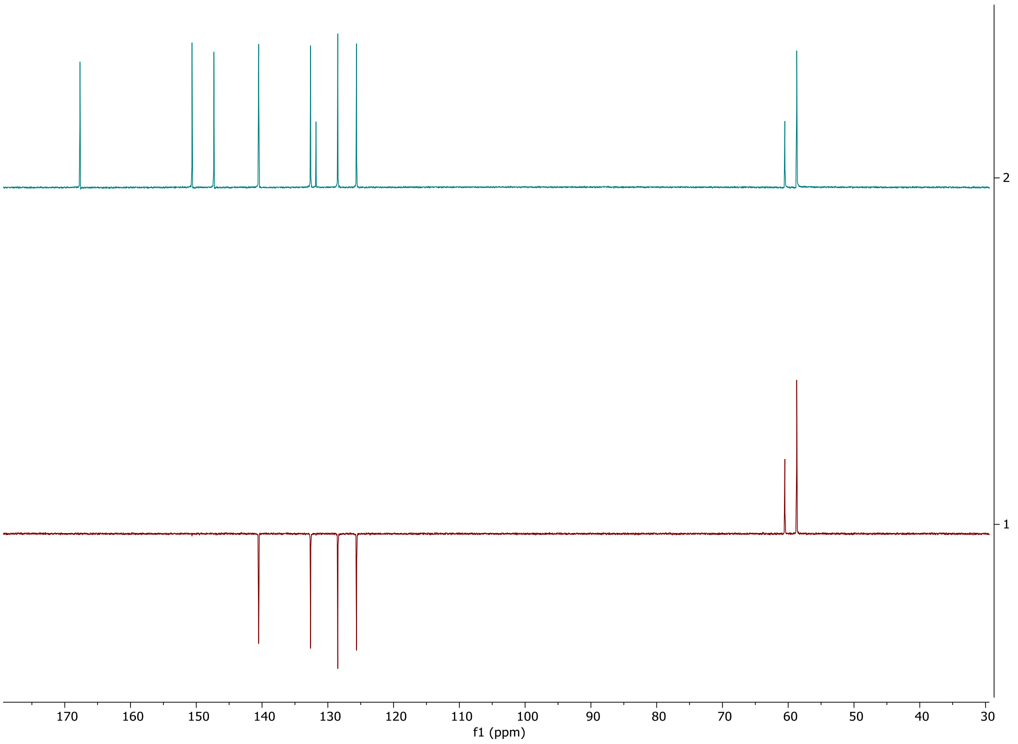

Figure S22. ^{13}C NMR and DEPT spectra (126 MHz, 298 K, D_2O) of H_4**tpapxd** (H_4**L**^{5}).

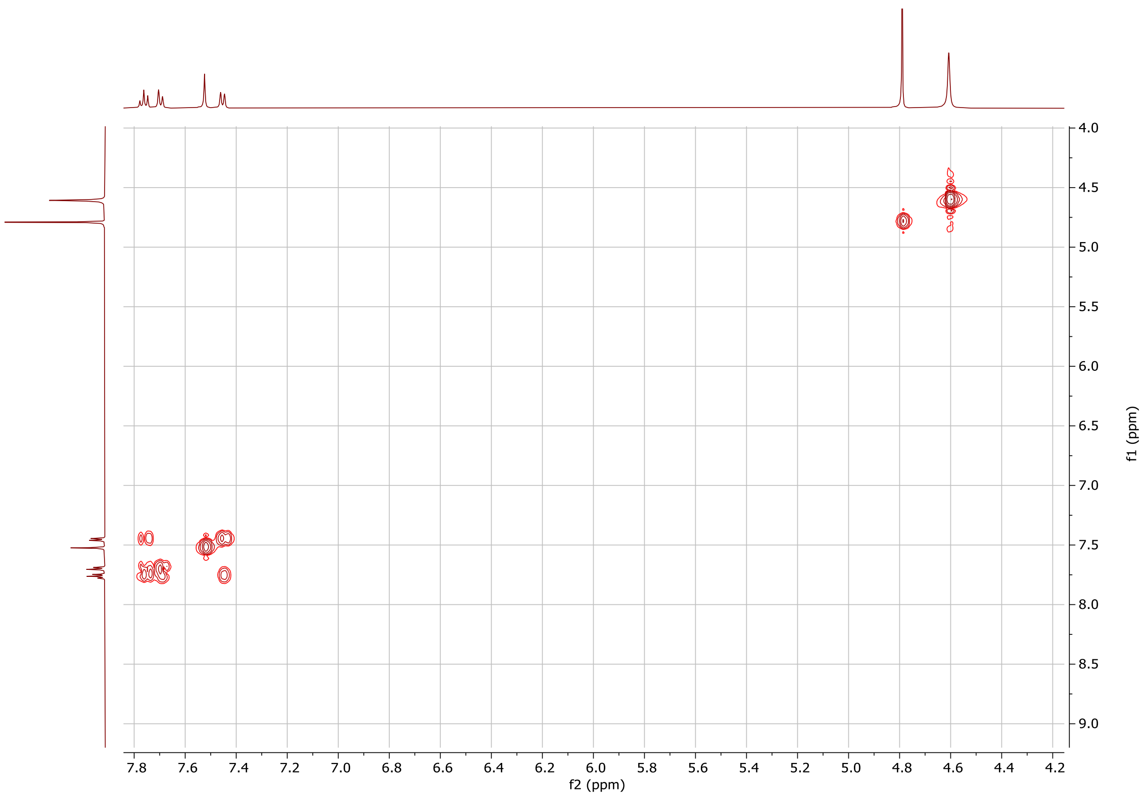

Figure S23. ^{1}H-^{1}H COSY NMR spectrum (500 MHz, 298 K, D_2O) of H_4**tpapxd** (H_4**L**^{5}).

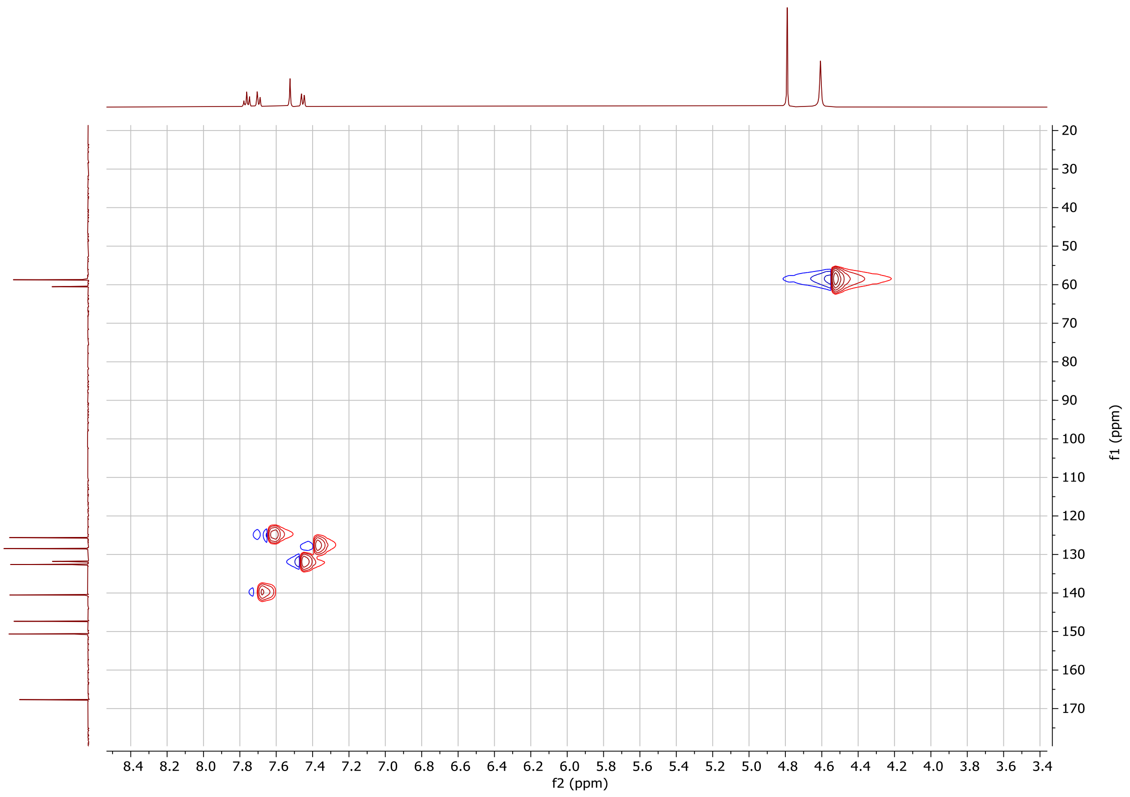

Figure S24. ^{1}H-^{13}C HSQC NMR spectrum of (500 MHz, 298 K, D_2O) of H_4**tpapxd** (H_4**L**^{5}).

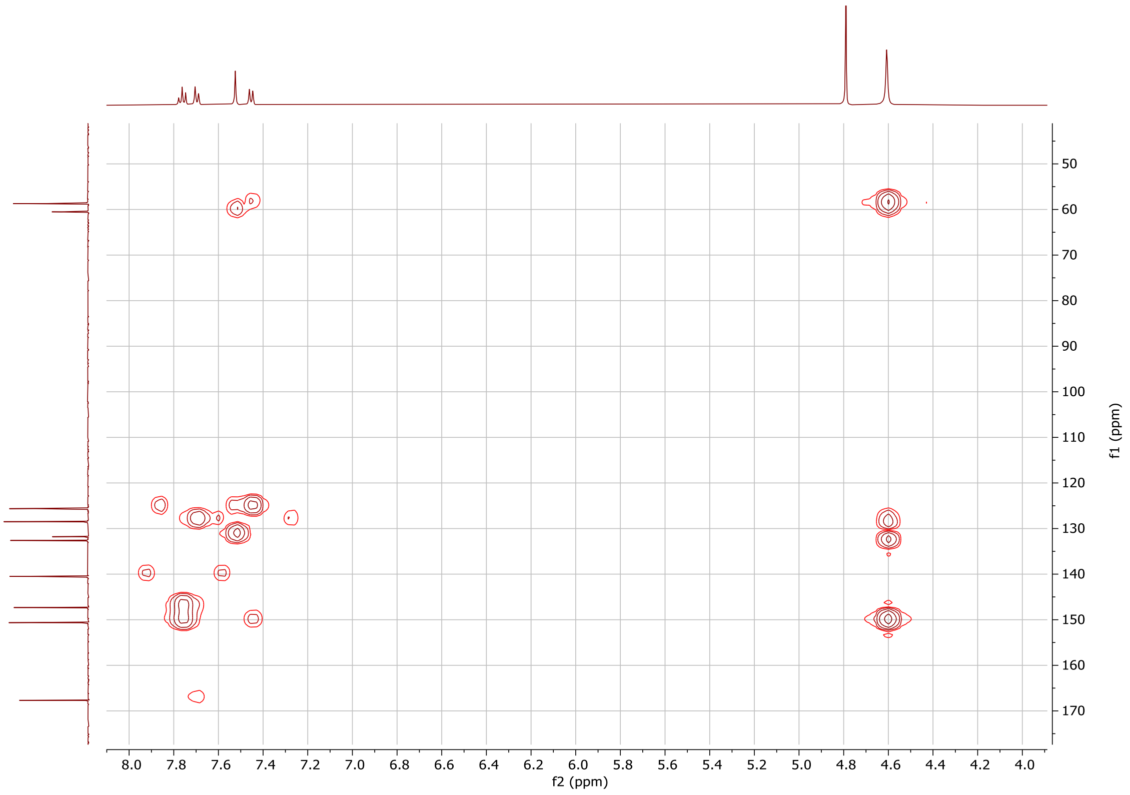

Figure S25. ^1H-^{13}C HMBC NMR spectrum (500 MHz, 298 K, D_2O) of H_4**tpapxd** (H_4**L^5**).

NMR spectra of H_4**tpadapo** (H_4**L**^{6}).

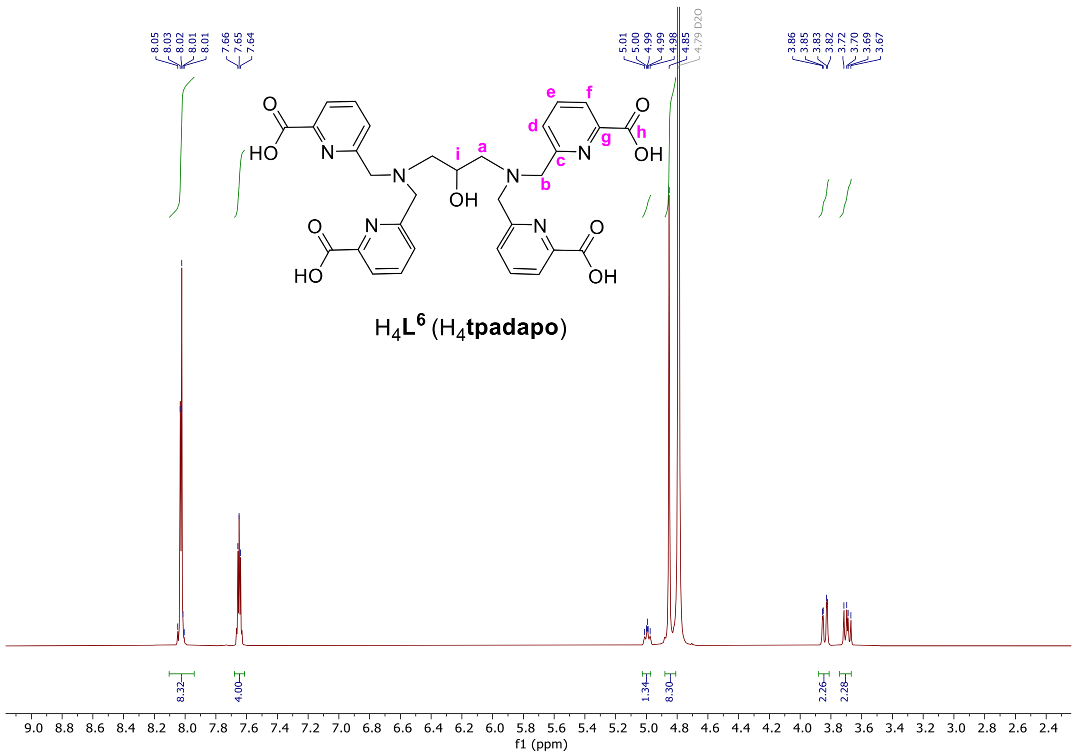

Figure S26. ^1H NMR spectrum (500 MHz, 298 K, D_2O) of H_4**tpadapo** (H_4**L**^{6}).

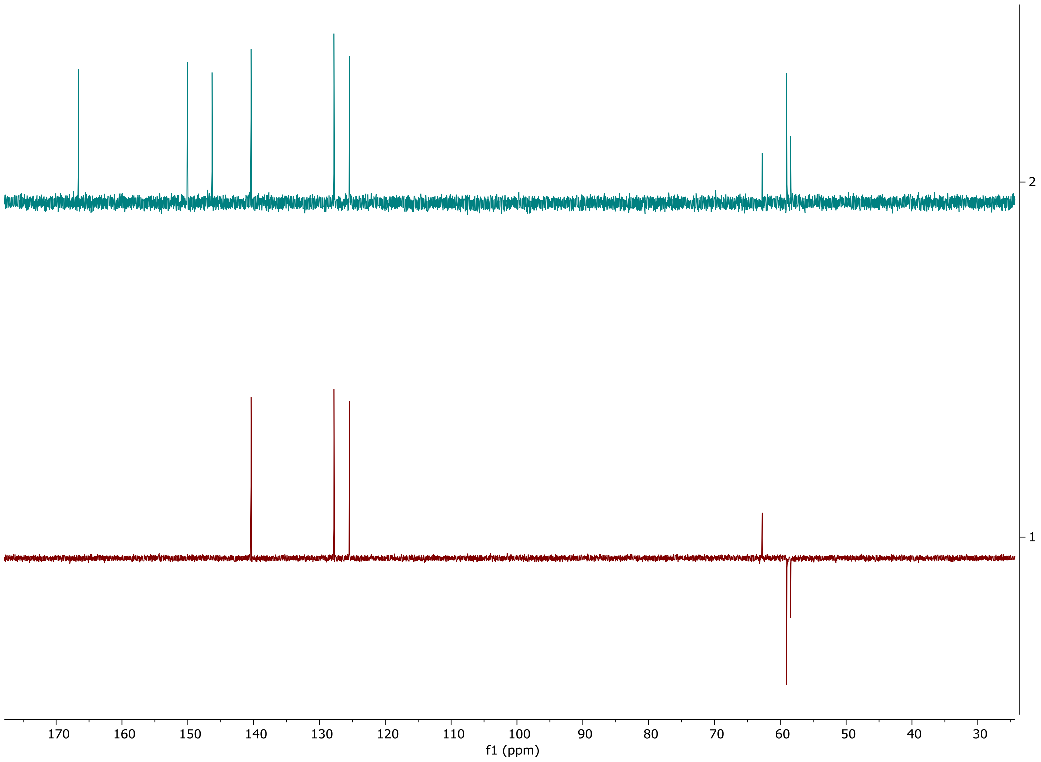

Figure S27. ^{13}C NMR and DEPT spectra (126 MHz, 298 K, D_2O) of H_4**tpadapo** (H_4**L**^{6}).

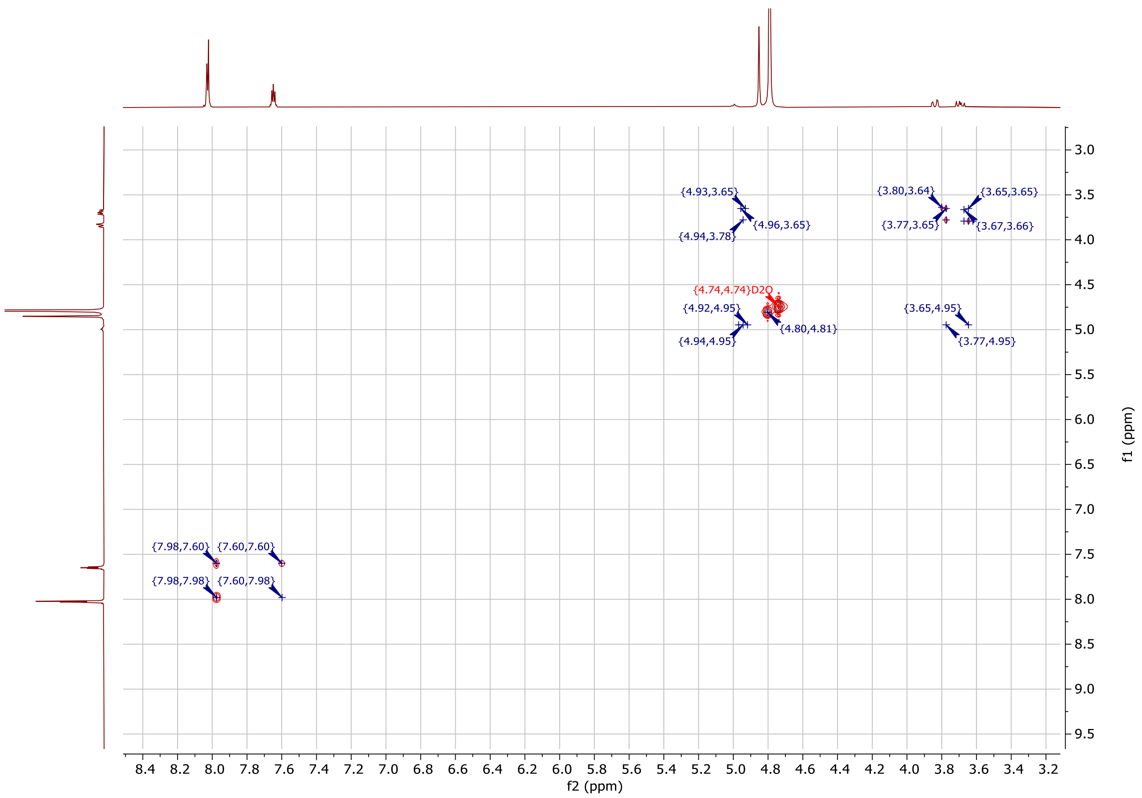

Figure S28. ^{1}H-^{1}H COSY NMR spectrum (500 MHz, 298 K, D_2O) of H_4**tpadapo** (H_4**L**^{6}).

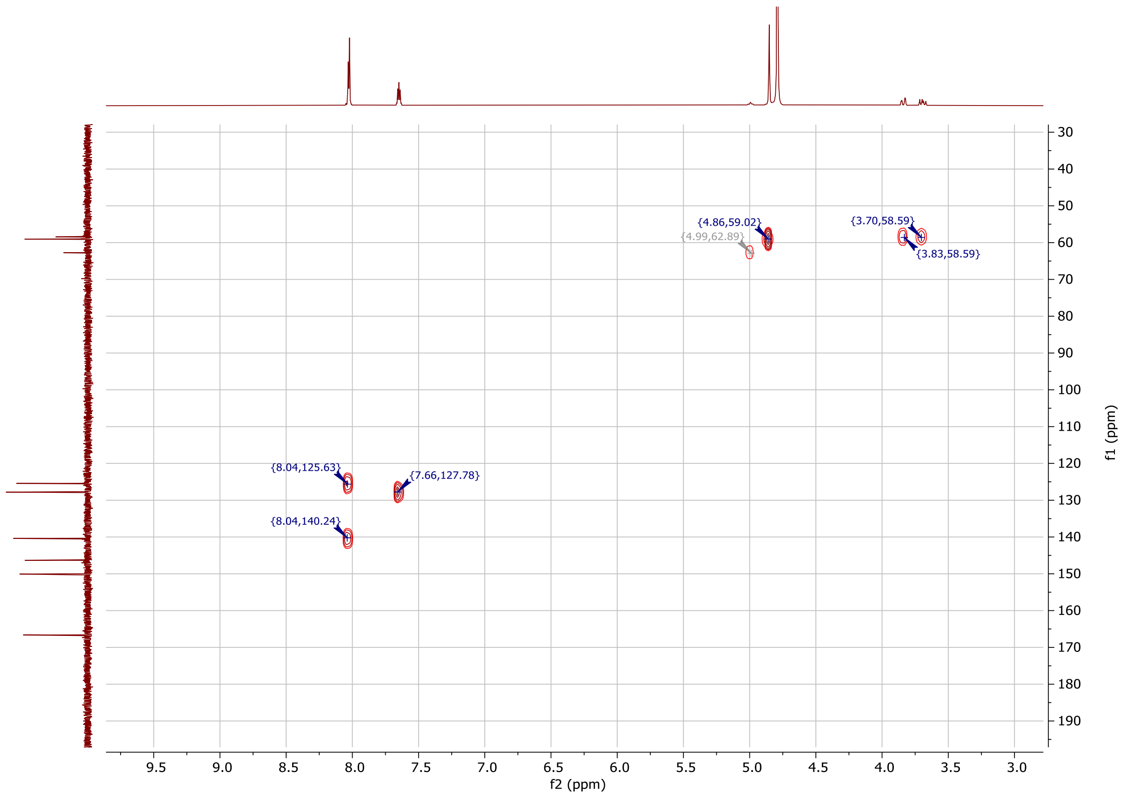

Figure S29. ^{1}H-^{13}C HSQC NMR spectrum (500 MHz, 298 K, D_2O) of H_4**tpadapo** (H_4**L**^{6}).

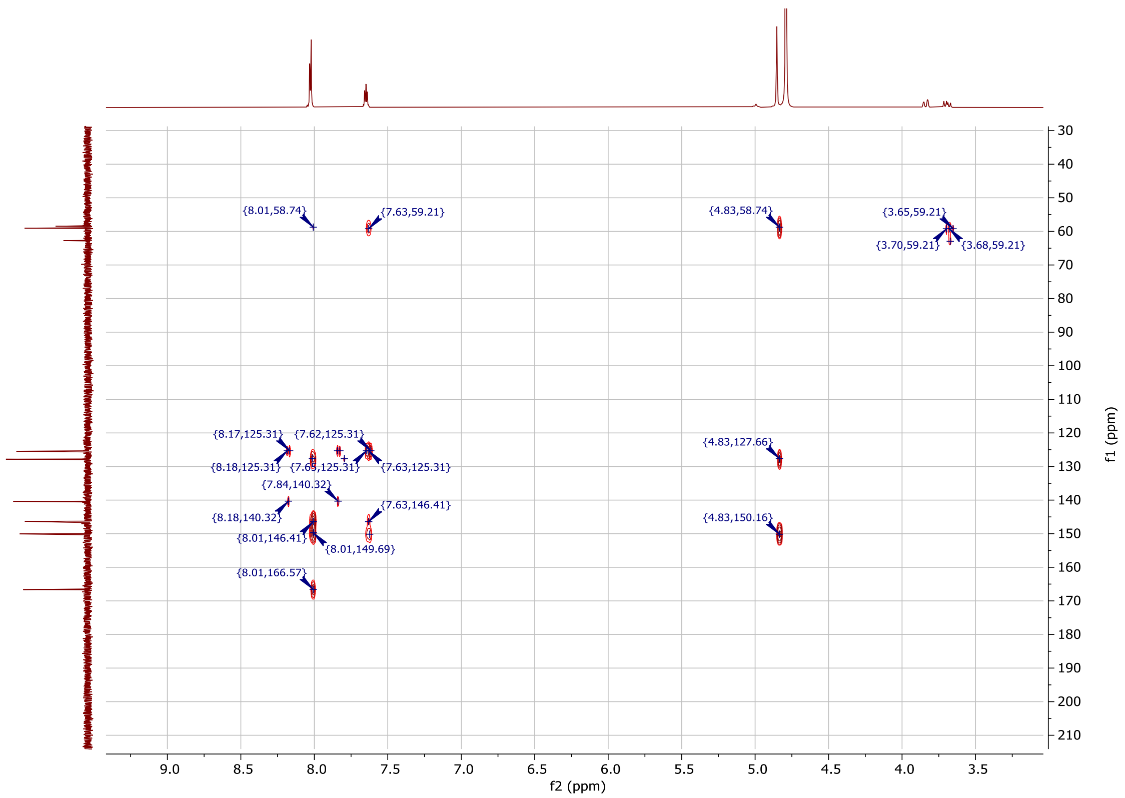

Figure S30. ^{1}H-^{13}C HMBC NMR spectrum (500 MHz, 298 K, D_2O) of H_4**tpadapo** (H_4**L**$^{\mathbf{6}}$).

NMR spectra of compound **(2)**.

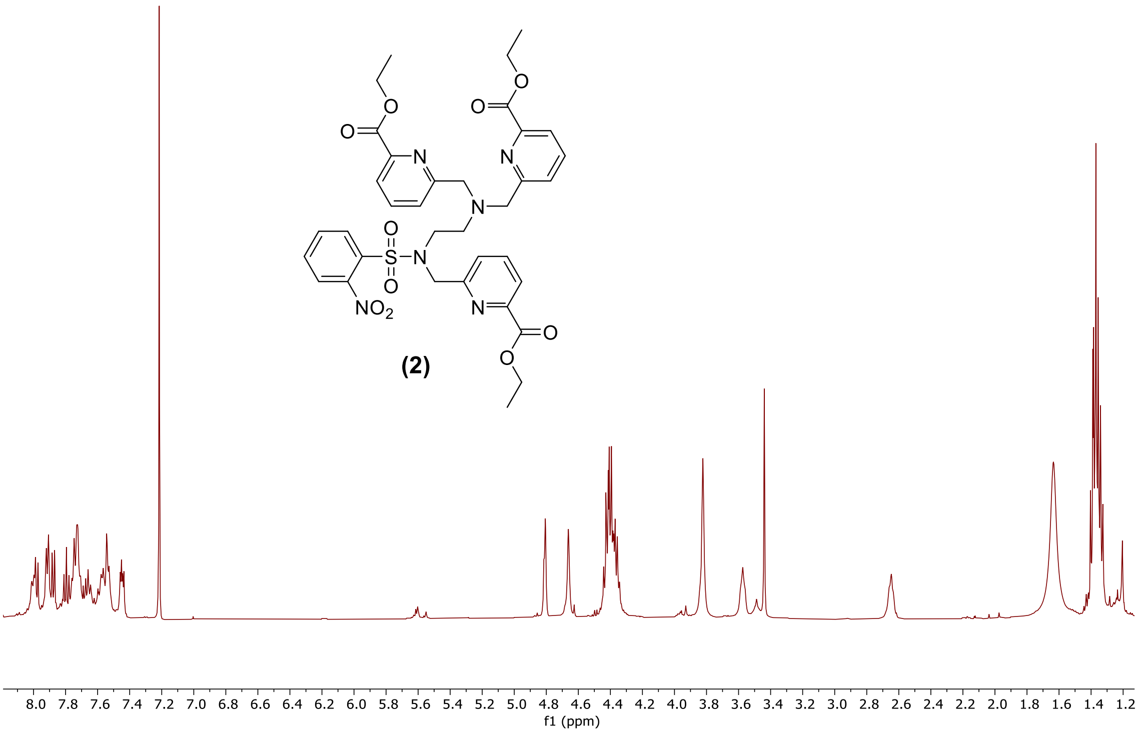

Figure S31. ^{1}H NMR spectrum (500 MHz, 298 K, $CDCl_3$) of compound (**2**).

NMR spectra of compound **(3)**.

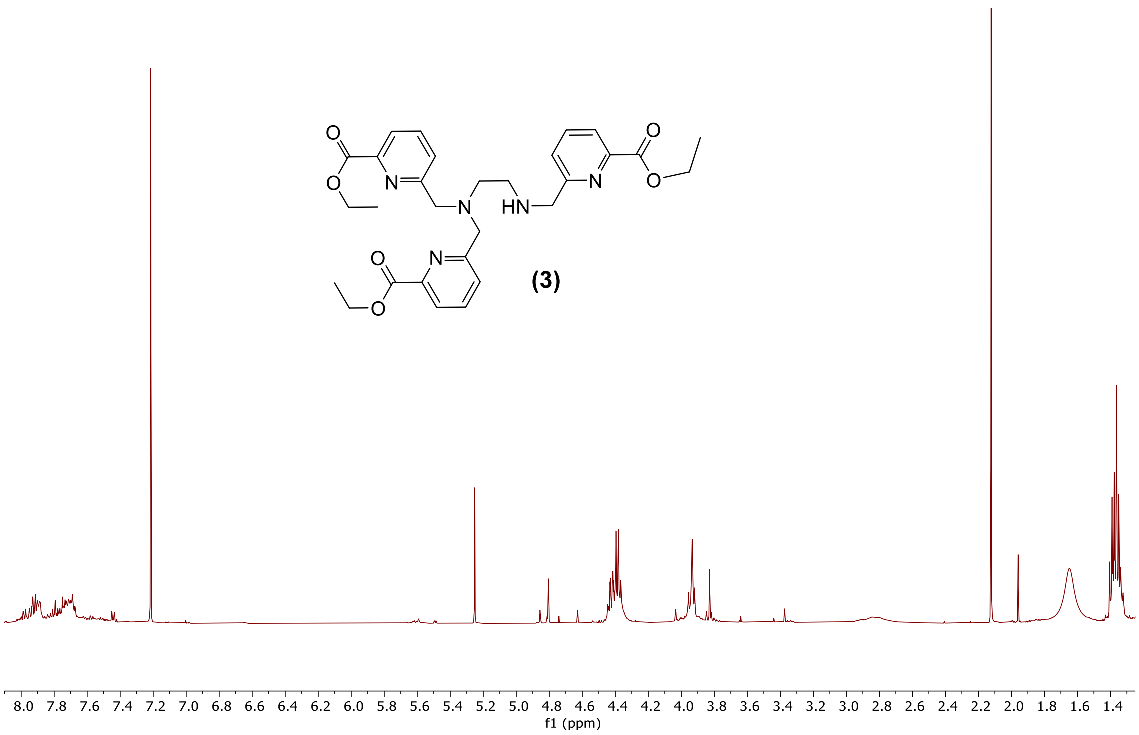

Figure S32. ^{1}H NMR spectrum (500 MHz, 298 K, $CDCl_3$) of compound (**3**).

NMR spectra of H_3**tripaen** (H_3**L**^{7}).

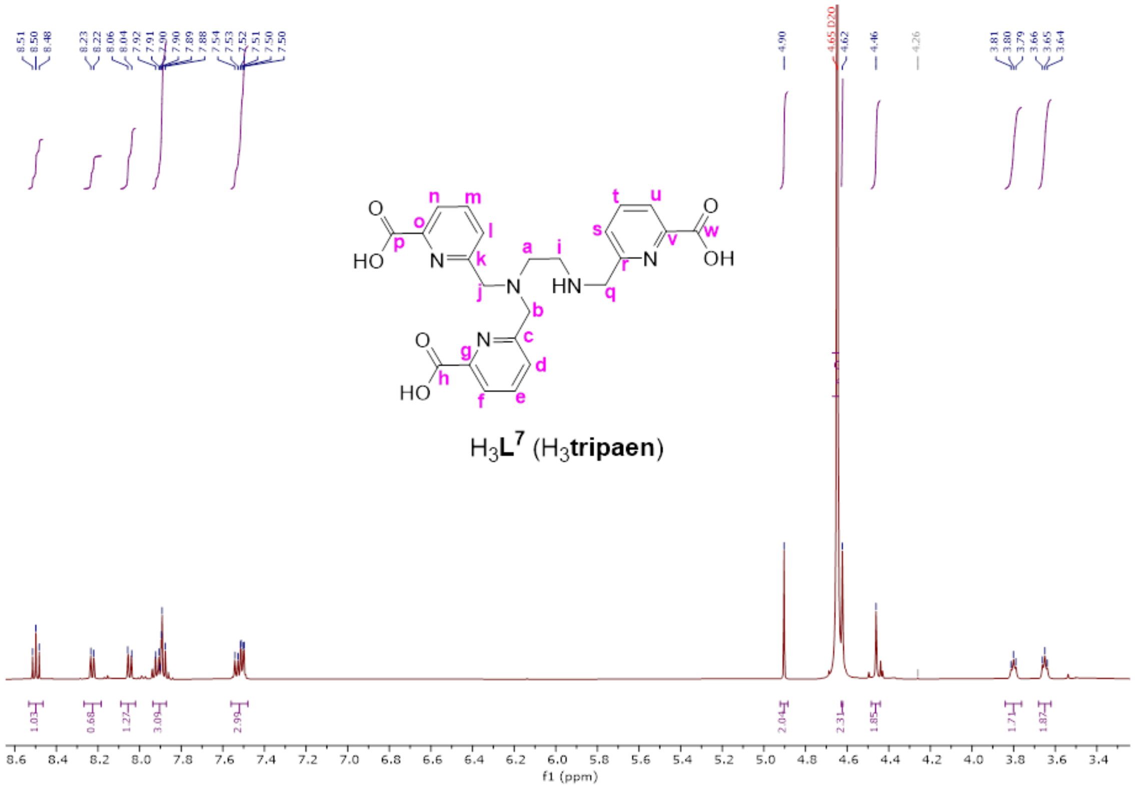

Figure S33. ^{1}H NMR spectrum (500 MHz, 298 K, D_2O) of H_3**tripaen** (H_3**L**^{7}).

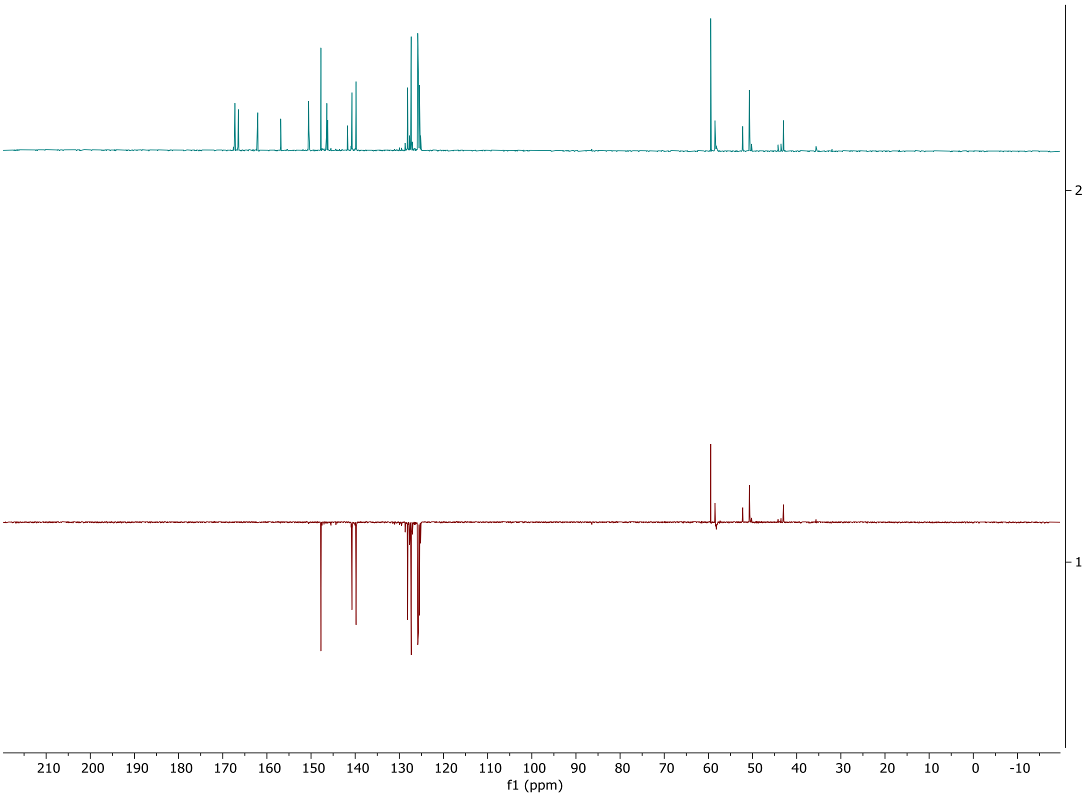

Figure S34. ^{13}C NMR and DEPT spectra (126 MHz, 298 K, D_2O) of H_3**tripaen** (H_3**L**^{7}).

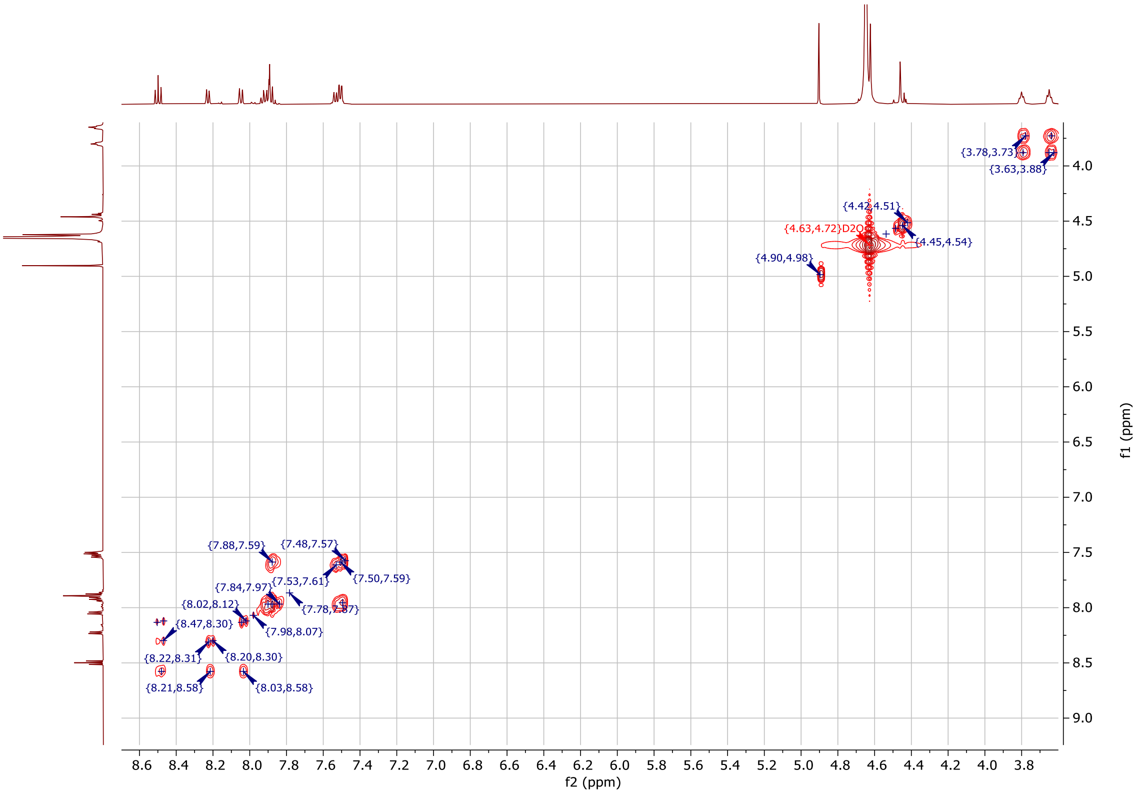

Figure S35. ¹H-¹H COSY NMR spectrum (500 MHz, 298 K, D_2O) of H_3**tripaen** (H_3**L**^{7}).

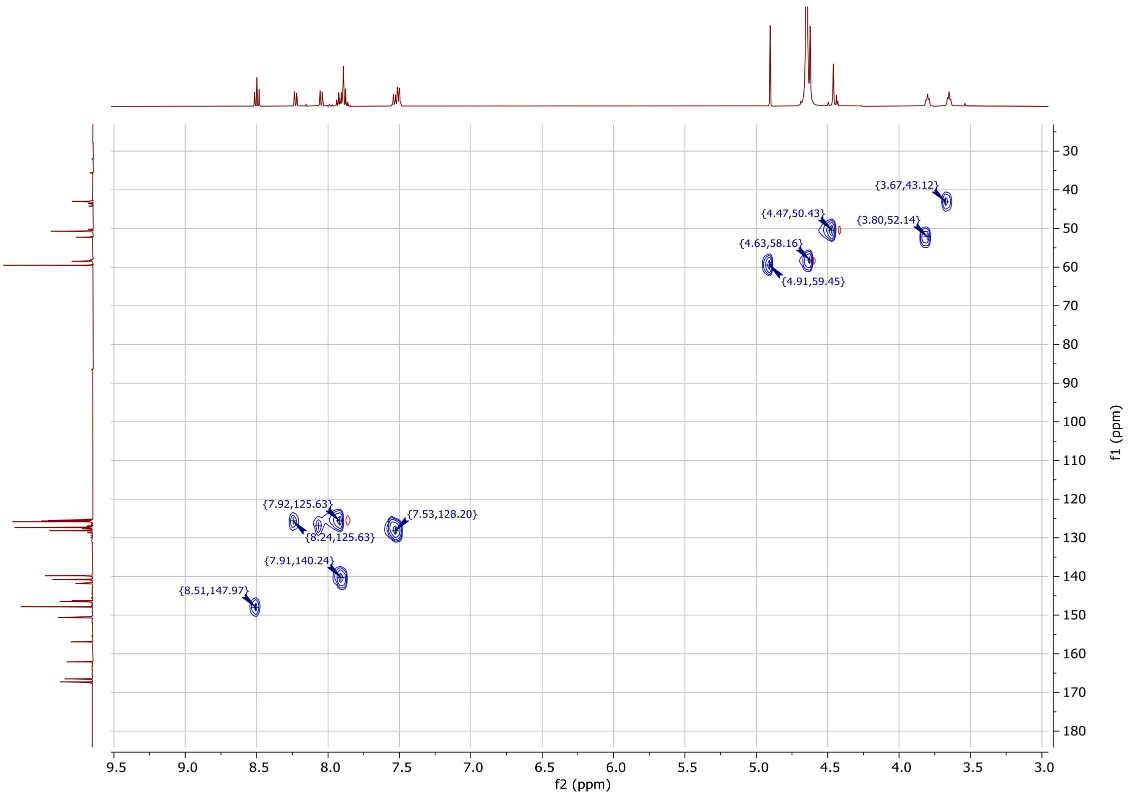

Figure S36. ¹H-¹³C HSQC NMR spectrum (500 MHz, 298 K, D_2O) of H_3**tripaen** (H_3**L**^{7}).

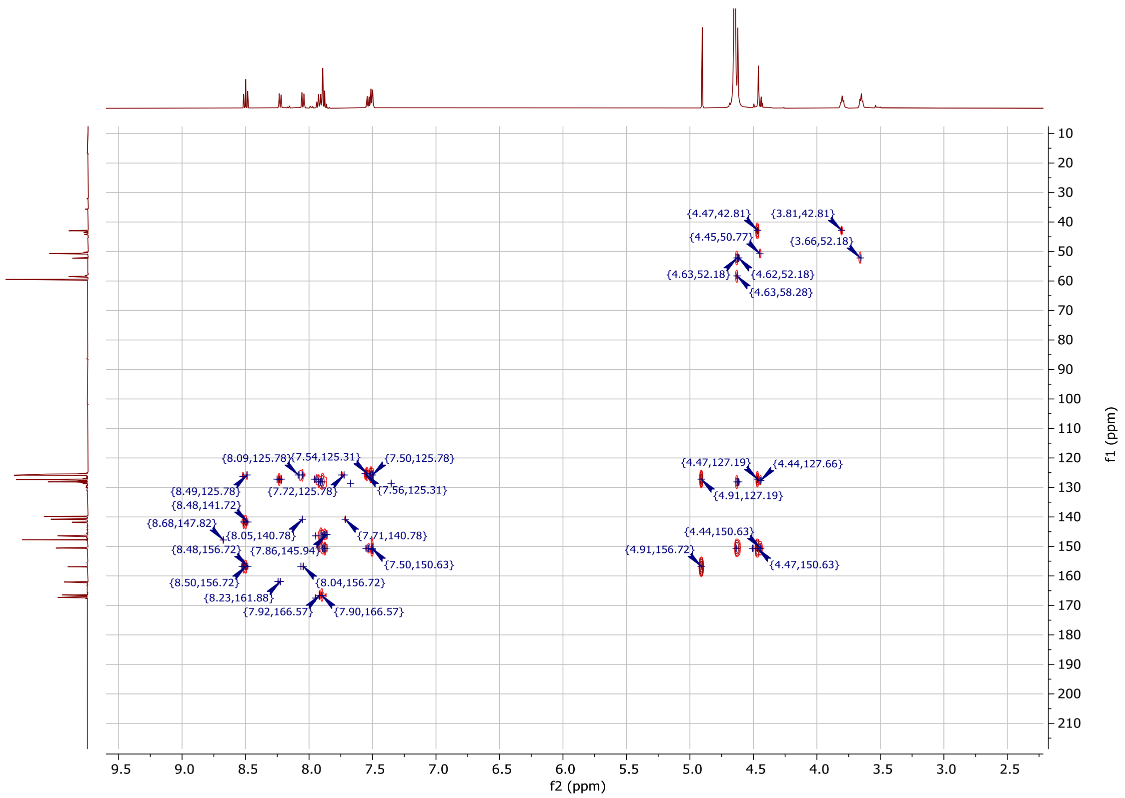

Figure S37. ^{1}H-^{13}C HMBC NMR spectrum (500 MHz, 298 K, D_2O) of H_3**tripaen** (H_3**L**^{7}).

NMR spectra of compound **(5)**.

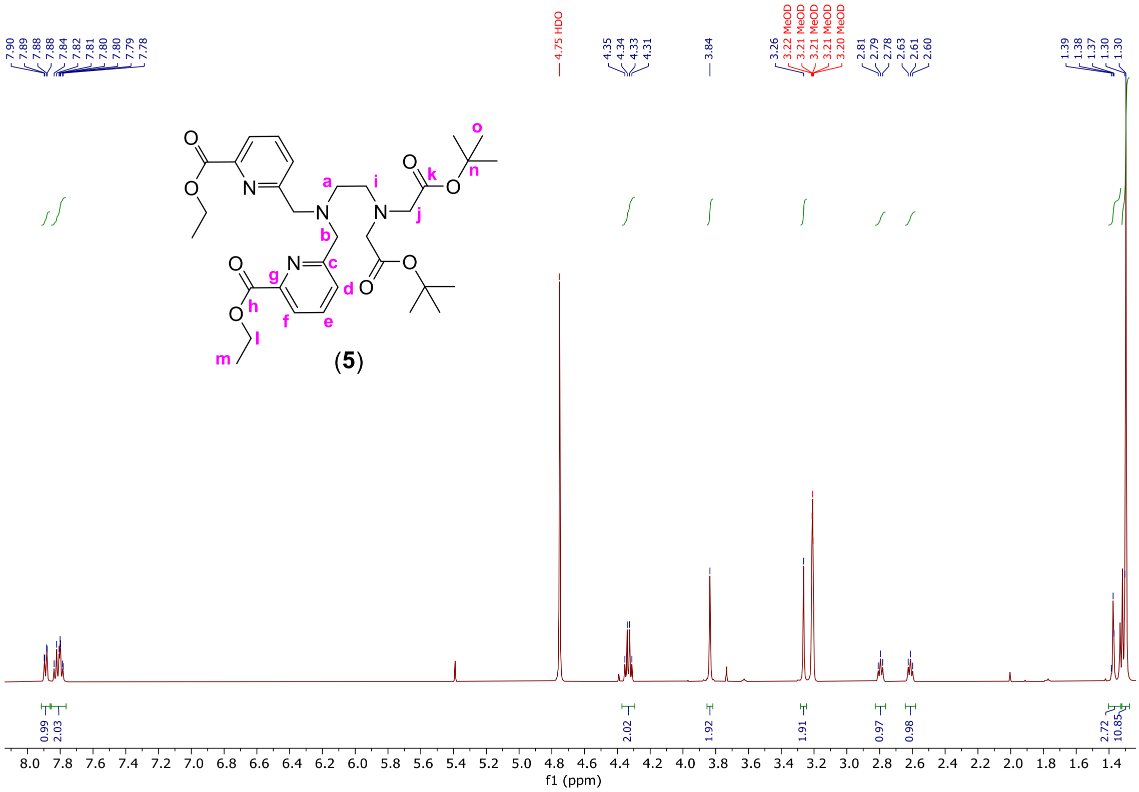

Figure S38. ^{1}H NMR spectrum (500 MHz, 298 K, MeOD) of compound (**5**).

NMR spectra of H_4**asyoctapa** (H_4**L**^{8}).

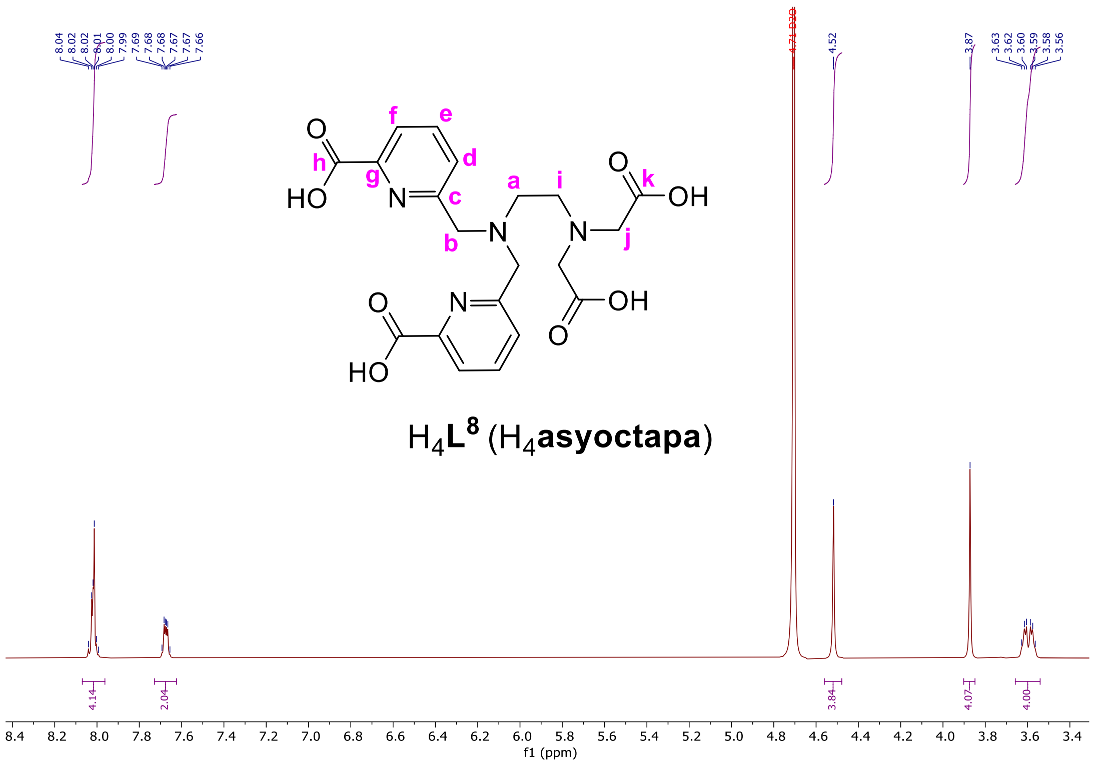

Figure S39. ^{1}H NMR spectrum (500 MHz, 298 K, D_2O) of H_4**asyoctapa** (H_4**L**^{8}).

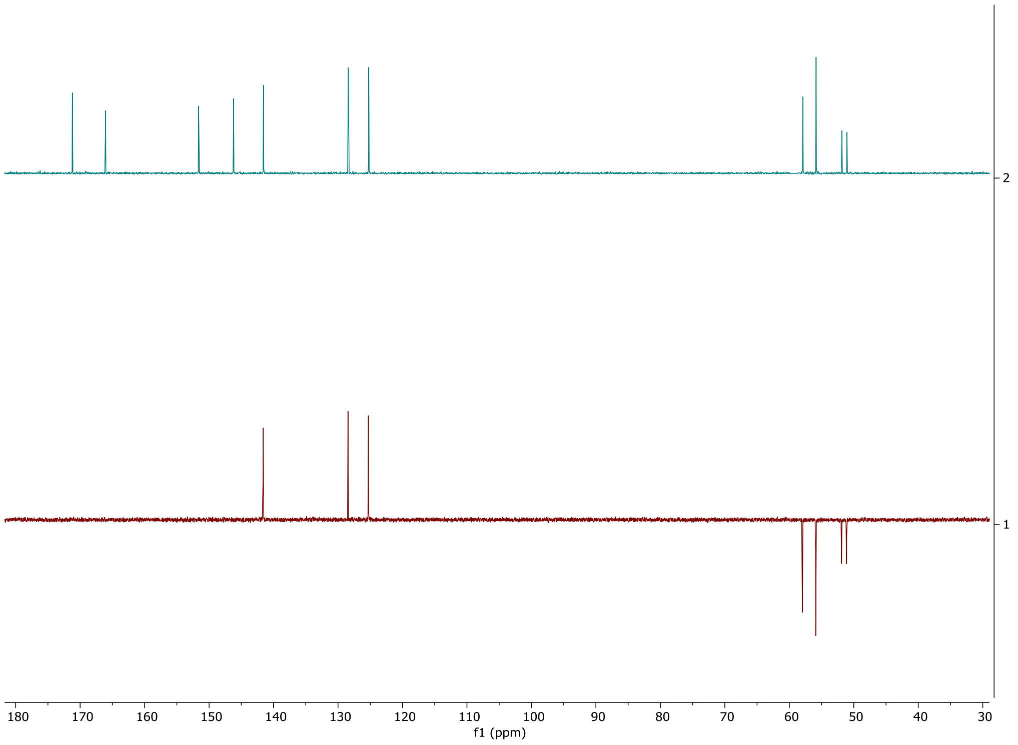

Figure S40. ^{13}C NMR and DEPT spectra (126 MHz, 298 K, D_2O) of H_4**asyoctapa** (H_4**L**^{8}).

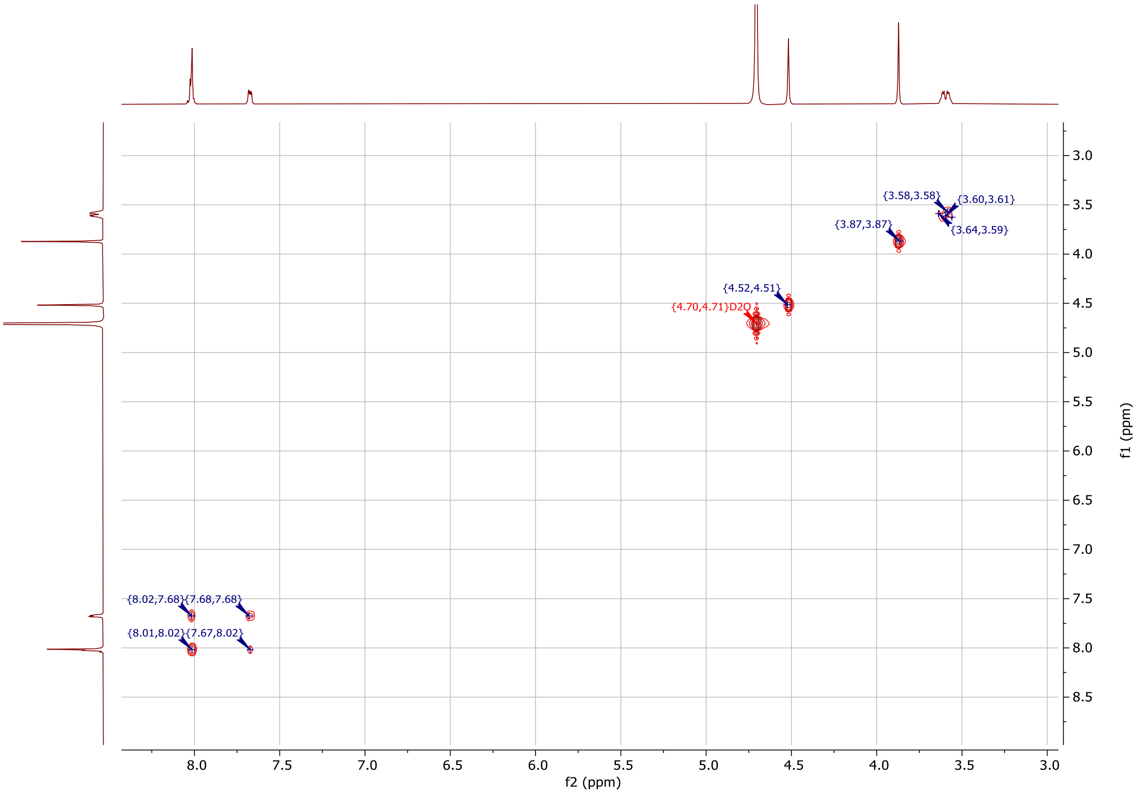

Figure S41. ^{1}H-^{1}H COSY NMR spectrum (500 MHz, 298 K, D_2O) of H_4**asyoctapa** ($H_4$$\mathbf{L^8}$).

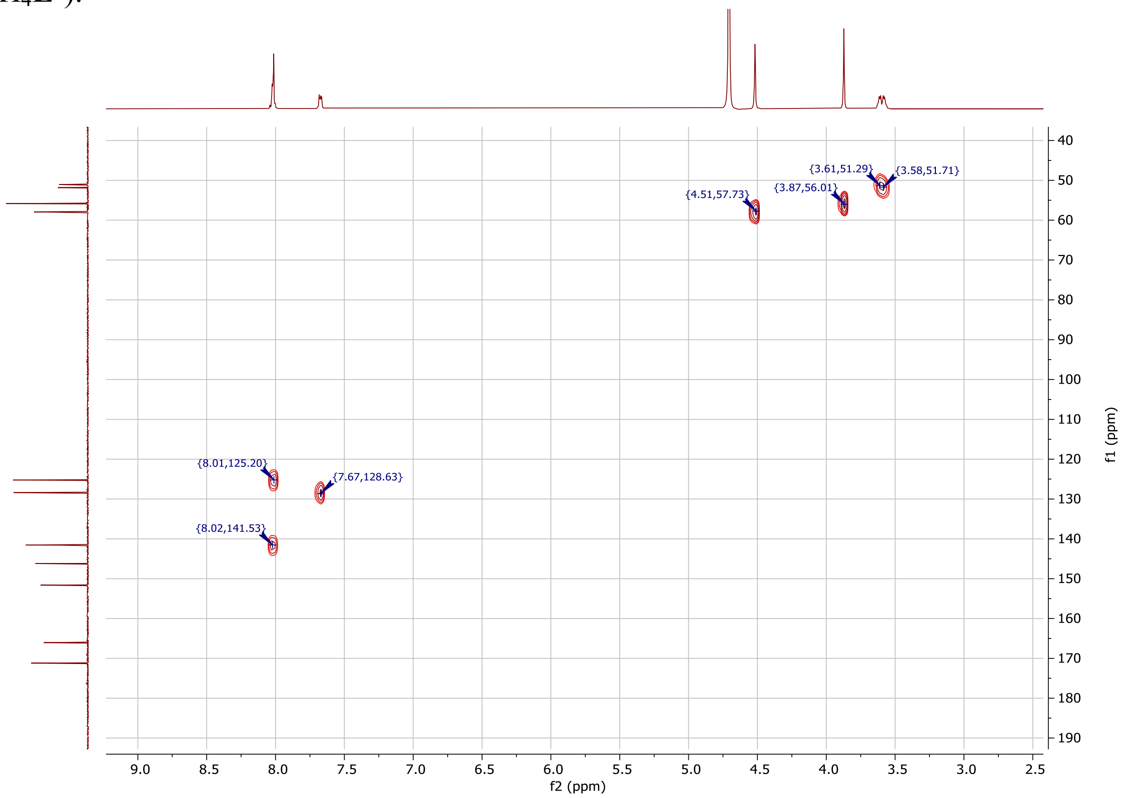

Figure S42. ^{1}H-^{13}C HSQC NMR spectrum (500 MHz, 298 K, D_2O) of H_4**asyoctapa** ($H_4$$\mathbf{L^8}$).

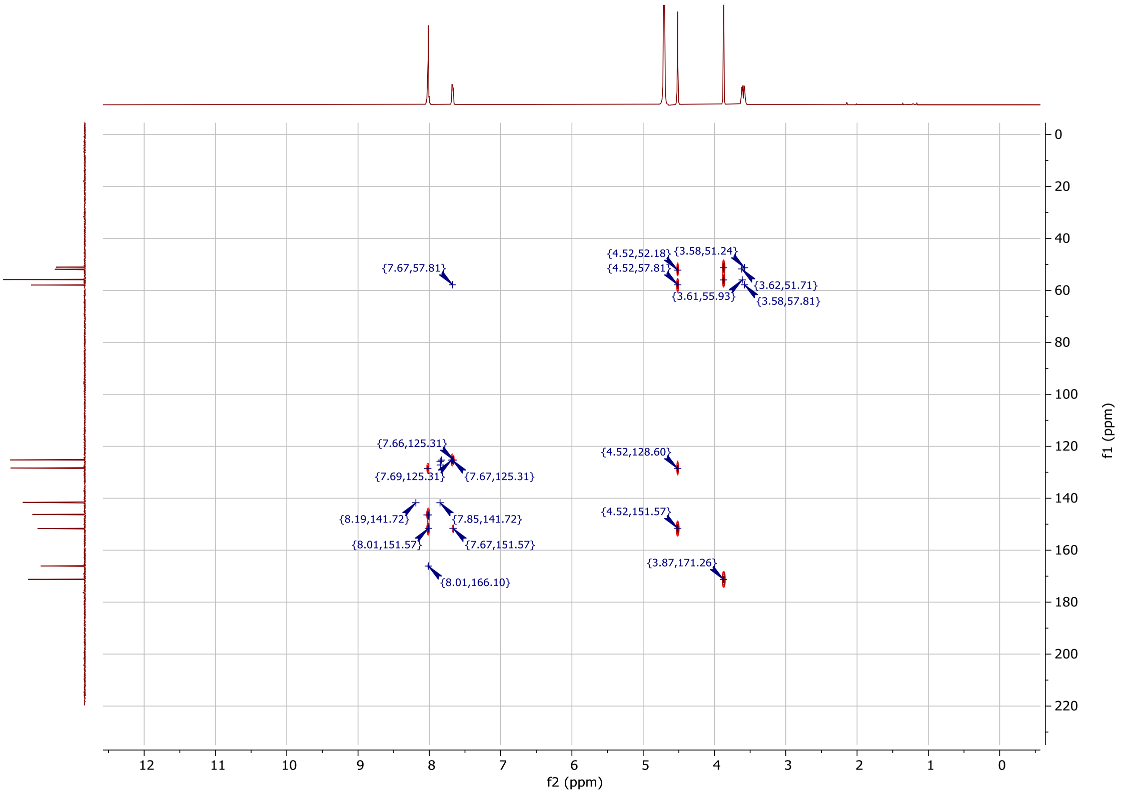

Figure S43. ^{1}H-^{13}C HMBC NMR spectrum (500 MHz, 298 K, D_2O) of H_4**asyoctapa** (H_4**L^8**).

NMR Spectra of lanthanum(III) complexes

NMR spectra of [La(**tpaen**)]⁻.

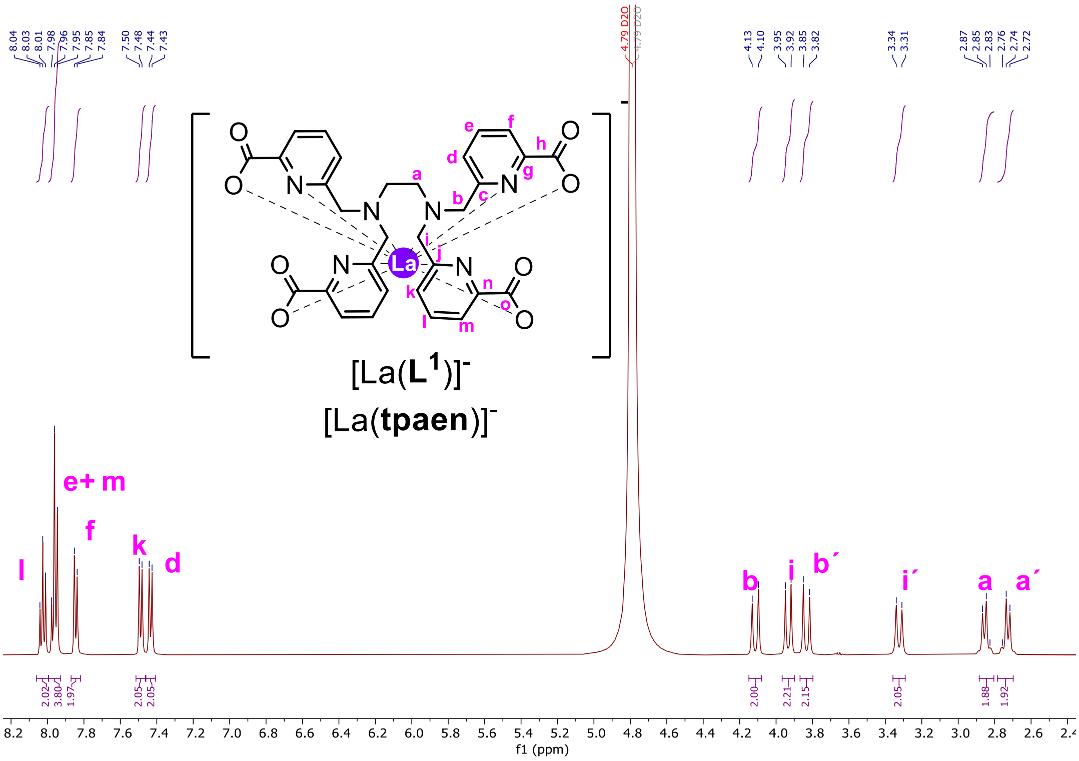

Figure S44. ^{1}H NMR spectrum (500 MHz, 298 K, D_2O pD=6) of [La(**tpaen**)]$^{-}$.

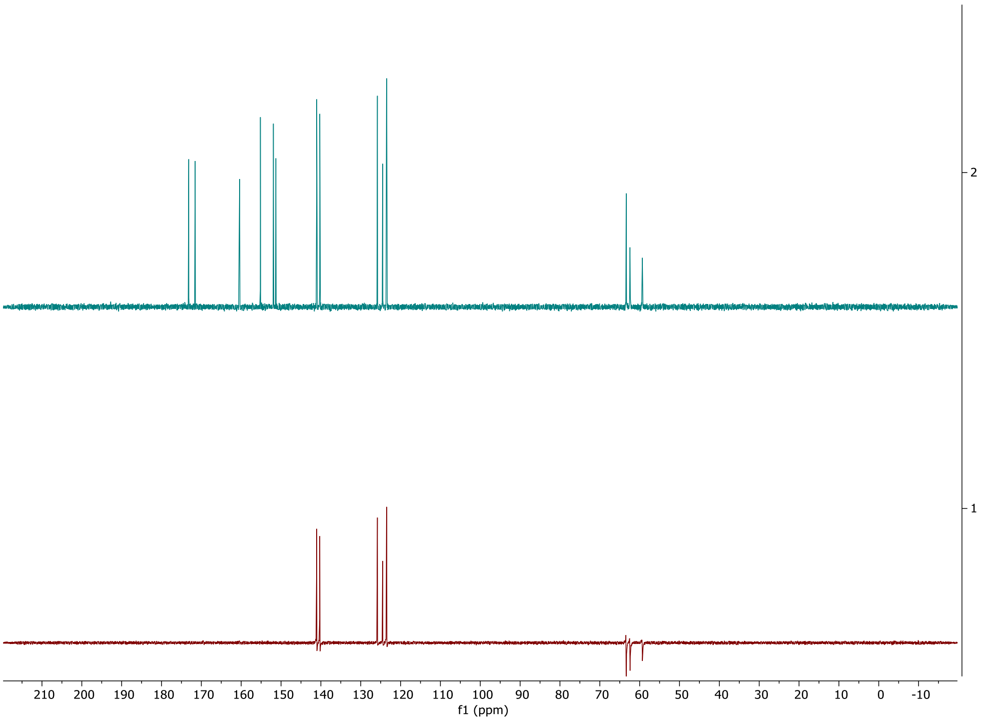

Figure S45. ^{13}C NMR and DEPT spectra (126 MHz, 298 K, D_2O pD=6) of [La(**tpaen**)]$^{-}$

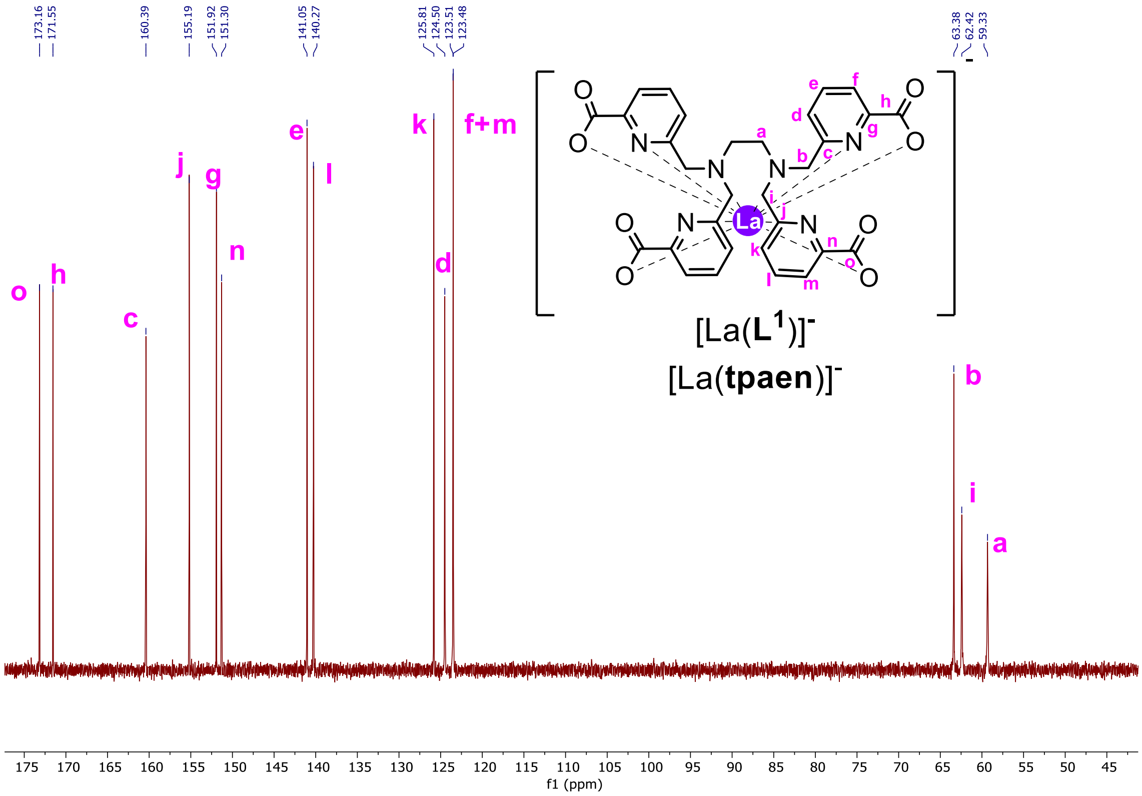

Figure S46. ^{13}C NMR spectrum (126 MHz, 298 K, D_2O pD=6) of [La(**tpaen**)]$^-$.

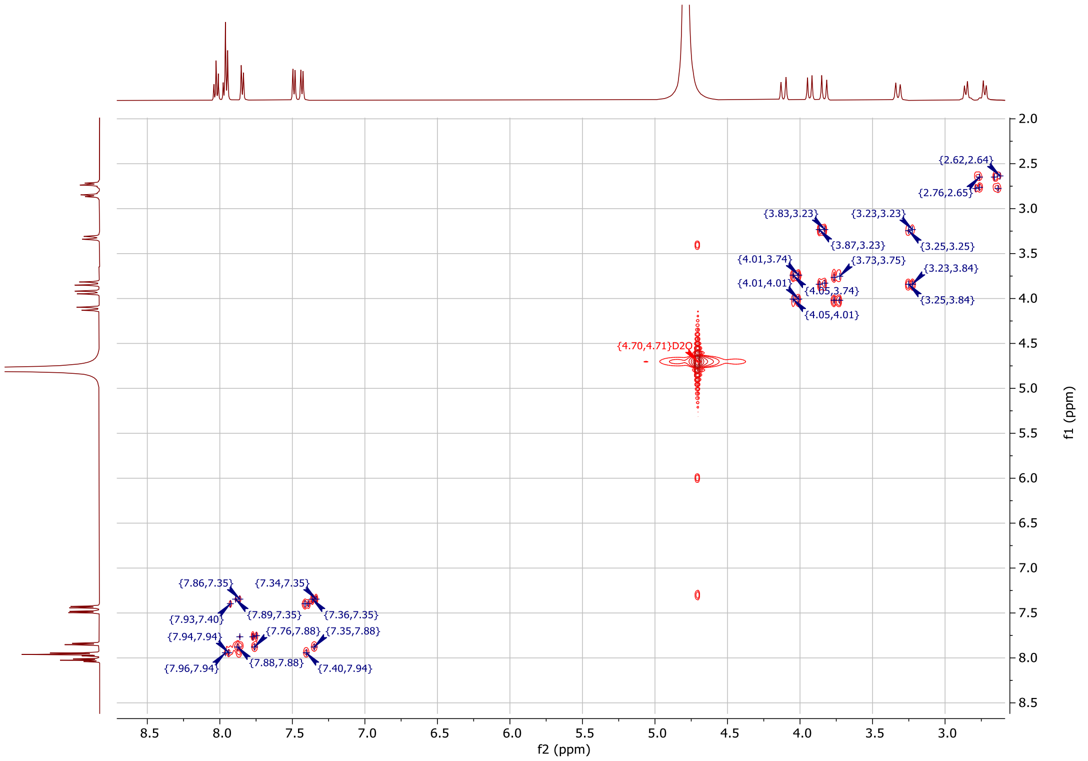

Figure S47. ^{1}H-^{1}H COSY NMR spectrum (500 MHz, 298 K, D_2O pD=6) of [La(**tpaen**)]$^-$.

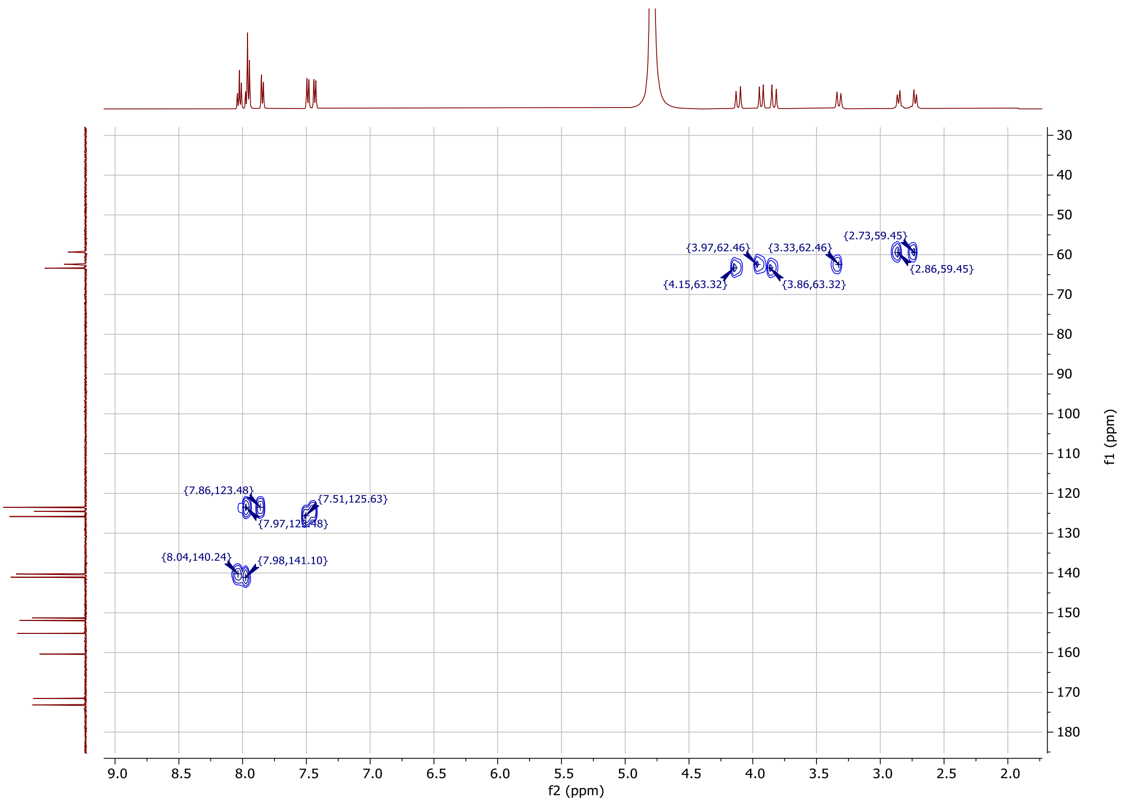

Figure S48. ^{1}H-^{13}C HSQC NMR spectrum (500 MHz, 298 K, D_2O pD=6) of [La(**tpaen**)]$^{-}$.

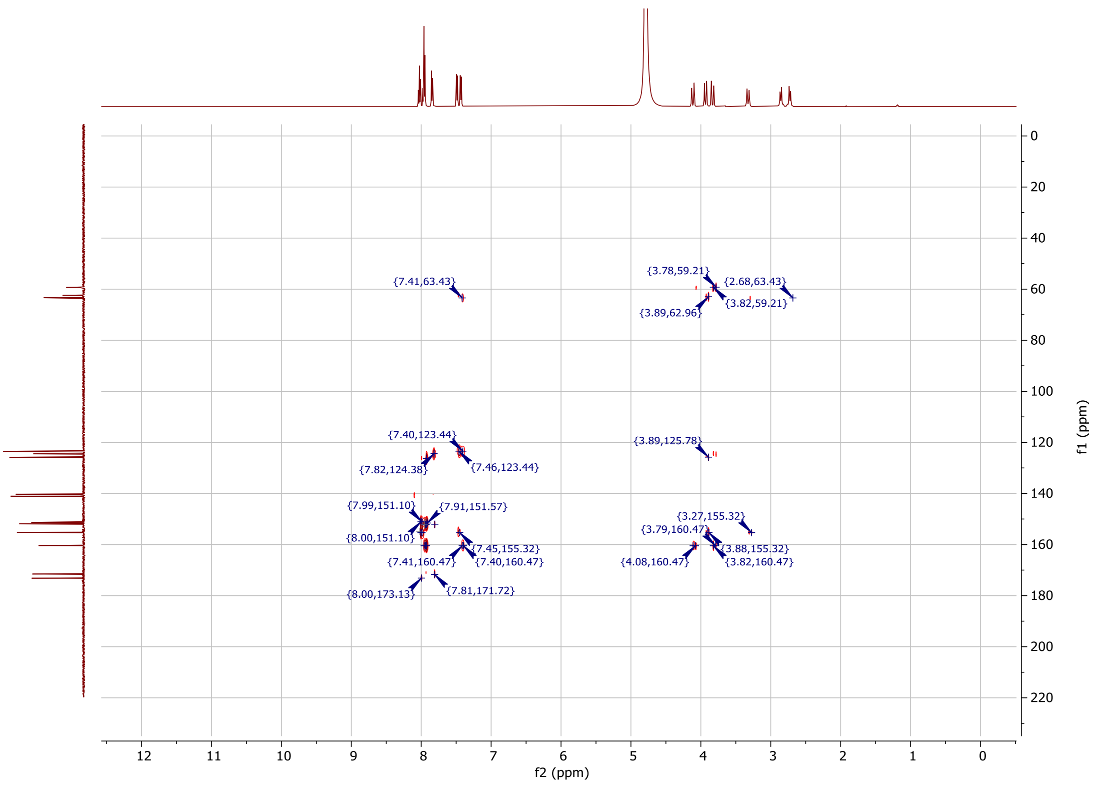

Figure S49. ^{1}H-^{13}C HMBC NMR spectrum (500 MHz, 298 K, D_2O pD=6) of [La(**tpaen**)]$^{-}$.

NMR spectra of [La(**tpaopd**)]⁻.

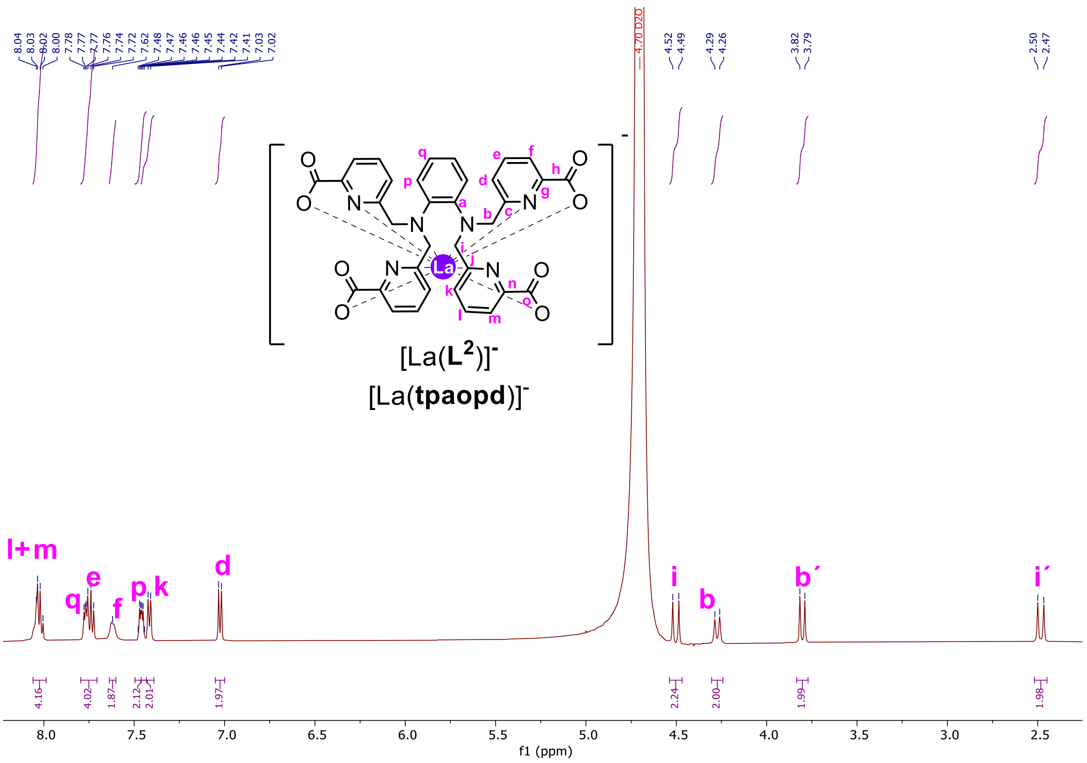

Figure S50. ^{1}H NMR spectrum (500 MHz, 298 K, D_2O pD=6) of [La(**tpaopd**)]⁻.

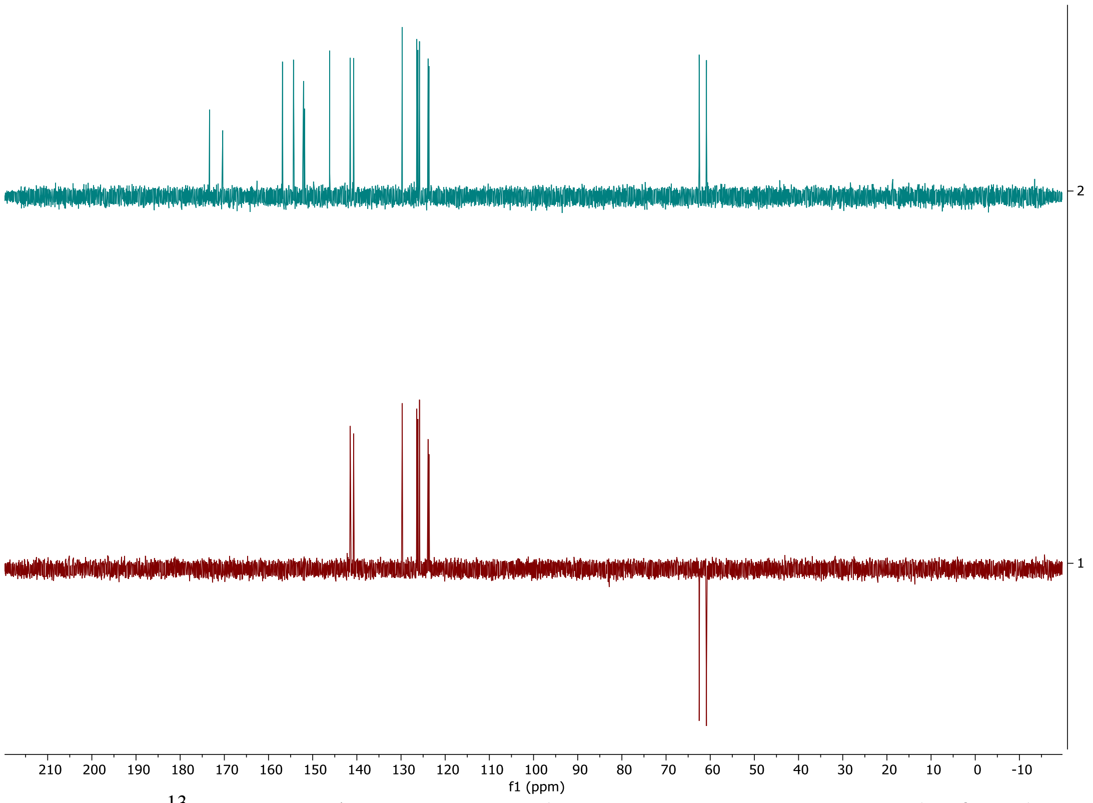

Figure S51. ^{13}C NMR and DEPT spectra (126 MHz, 298 K, D_2O pD=6) of [La(**tpaopd**)]⁻.

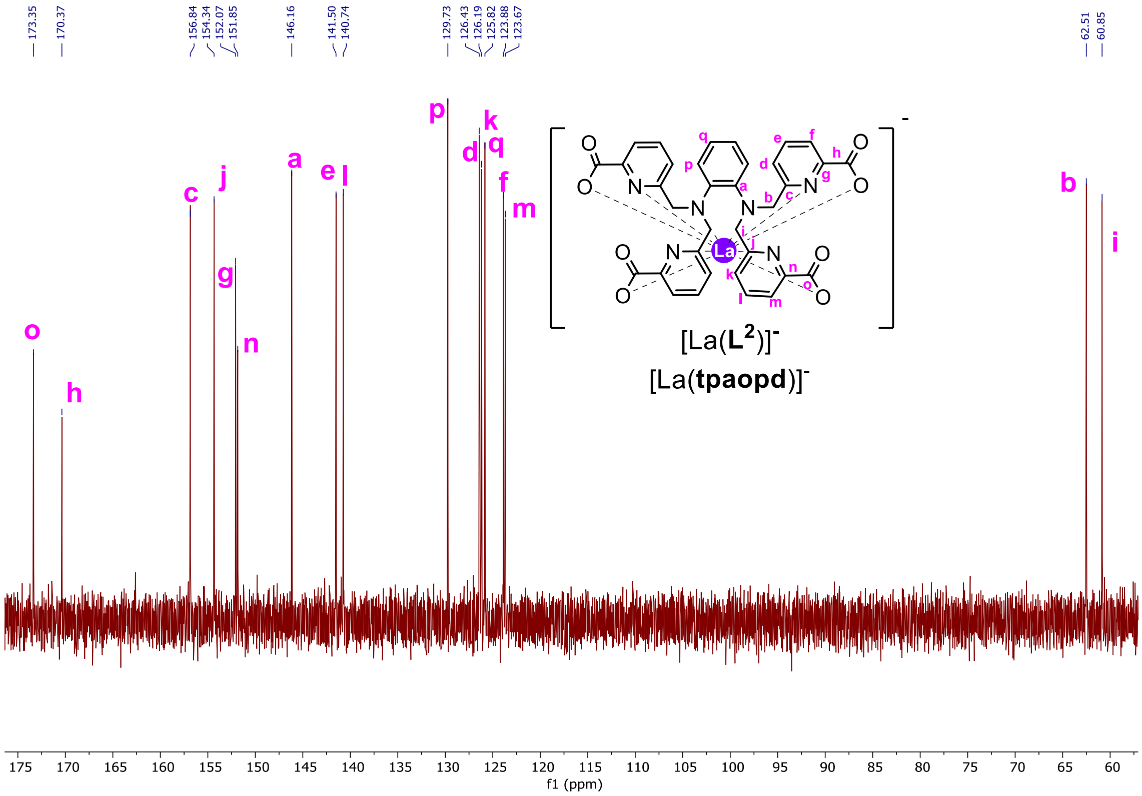

Figure S52. ^{13}C NMR spectrum (126 MHz, 298 K, D_2O pD=6) of [La(**tpaopd**)]$^-$.

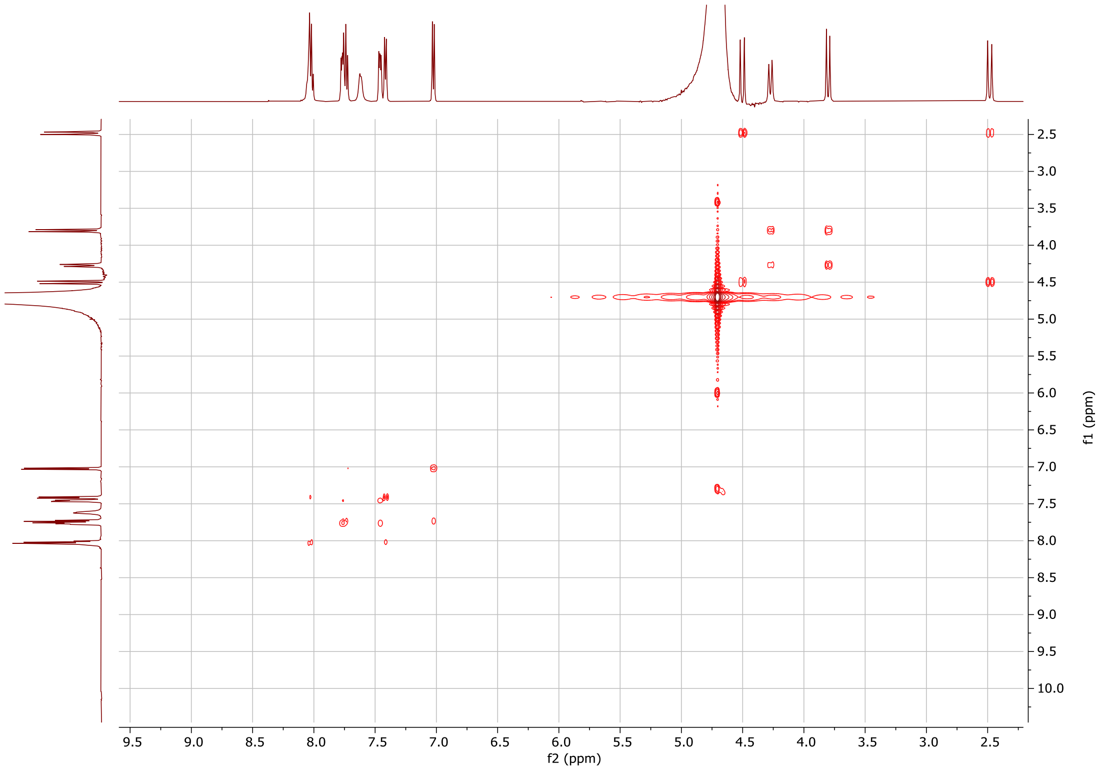

Figure S53. ^{1}H-^{1}H COSY NMR spectrum (500 MHz, 298 K, D_2O pD=6) of [La(**tpaopd**)]$^-$.

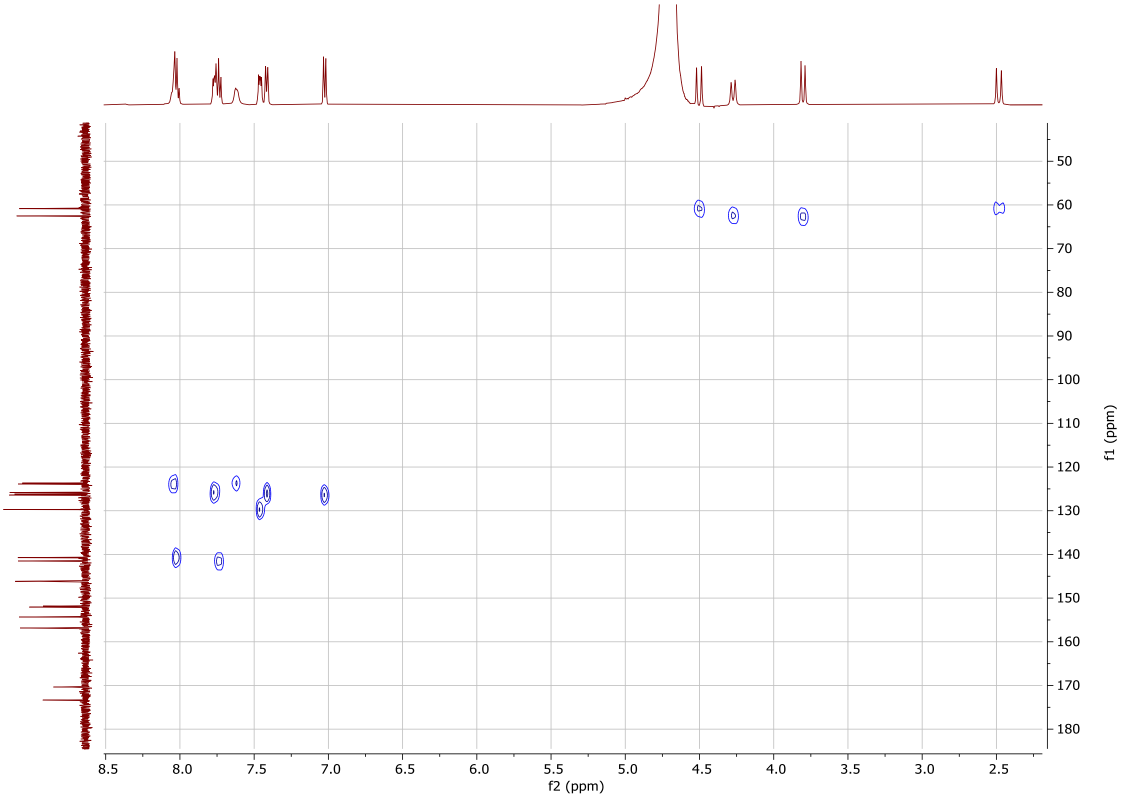

Figure S54. ^{1}H-^{13}C HSQC NMR spectrum (500 MHz, 298 K, D_2O pD=6) of [La(**tpaopd**)]$^{-}$.

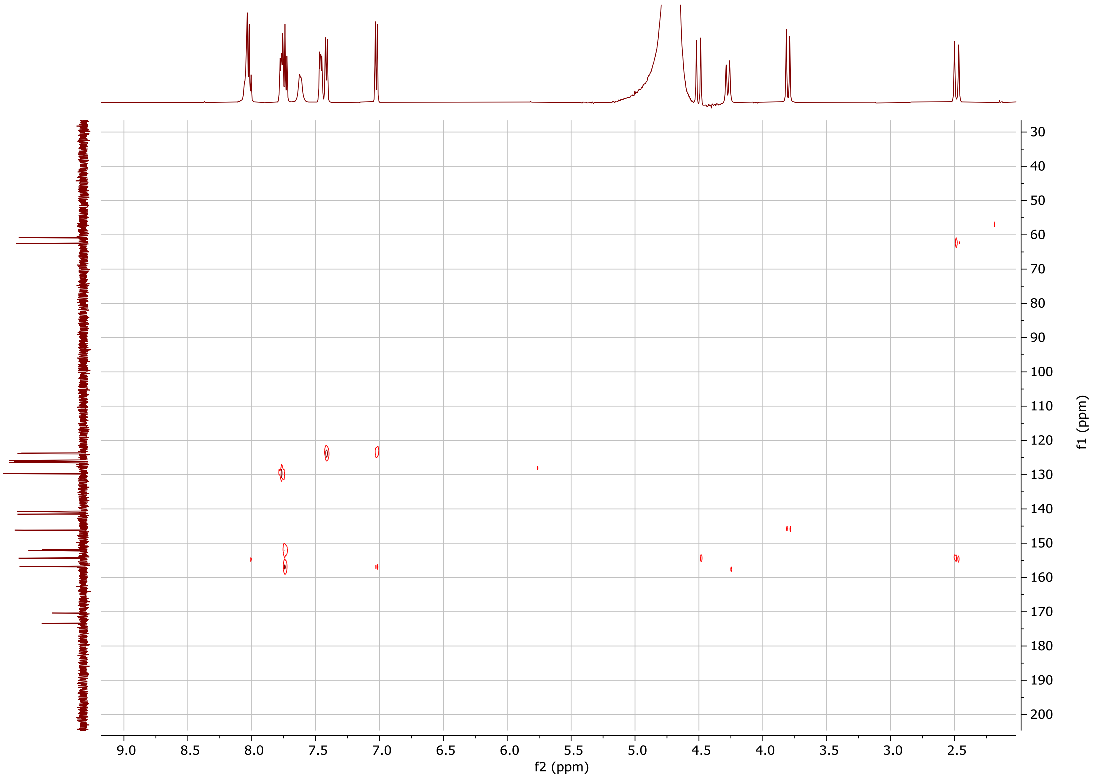

Figure S55. ^{1}H-^{13}C HMBC NMR spectrum (500 MHz, 298 K, D_2O pD=6) of [La(**tpaopd**)]$^{-}$.

NMR spectra of [La(**tpaond**)]$^-$.

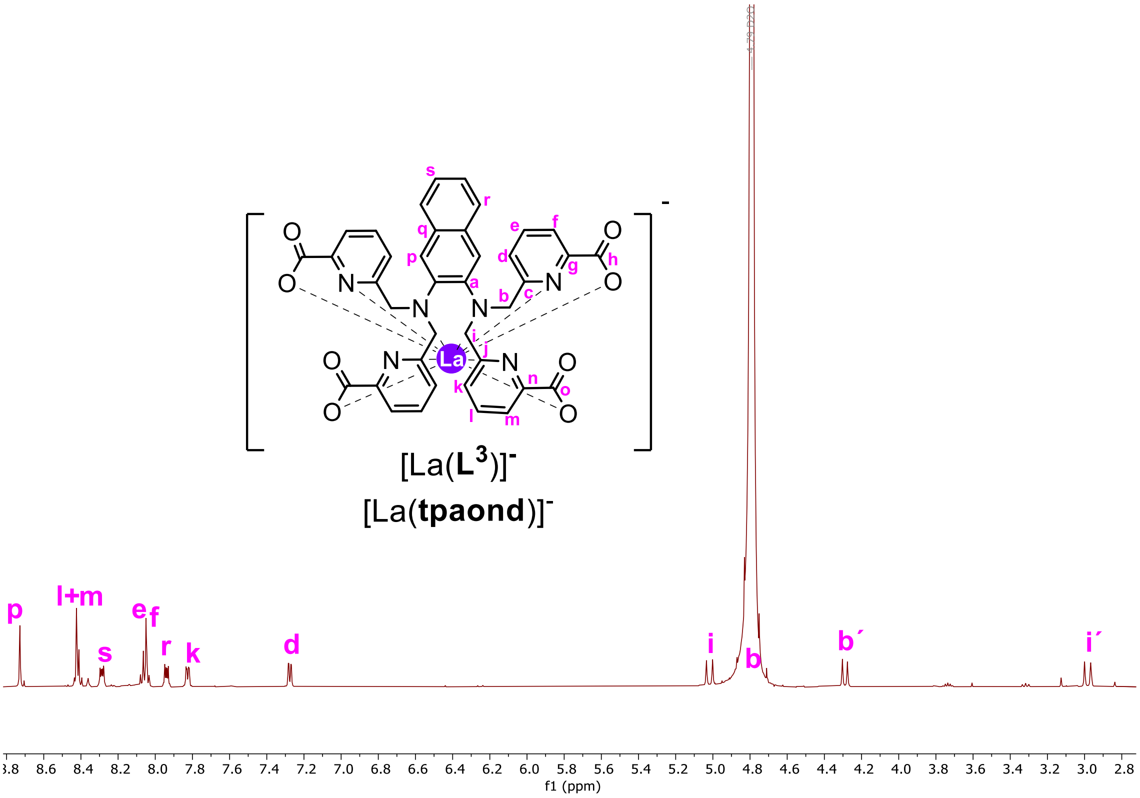

Figure S56. ^{1}H NMR spectrum (500 MHz, 298 K, D_2O:CD_3CN (7:3) pD=6) of [La(**tpaond**)]$^-$

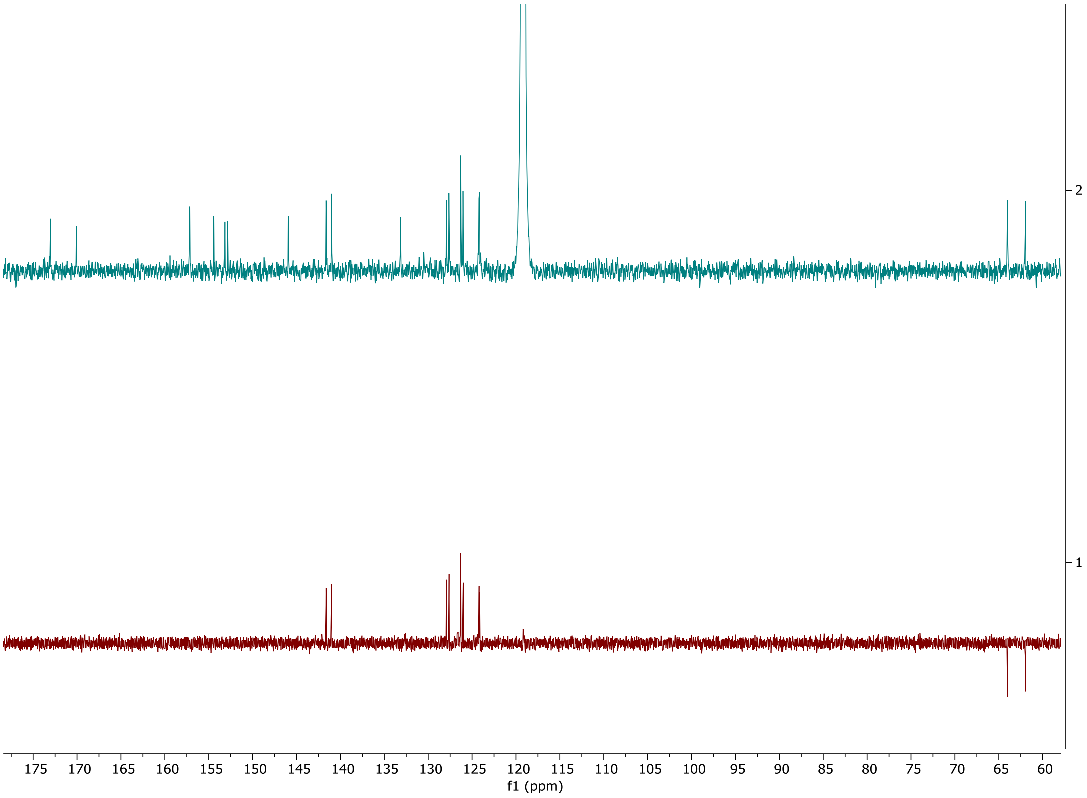

Figure S57. ^{13}C NMR and DEPT spectra (126 MHz, 298 K, D_2O:CD_3CN (7:3) pD=6) of [La(**tpaond**)]$^-$.

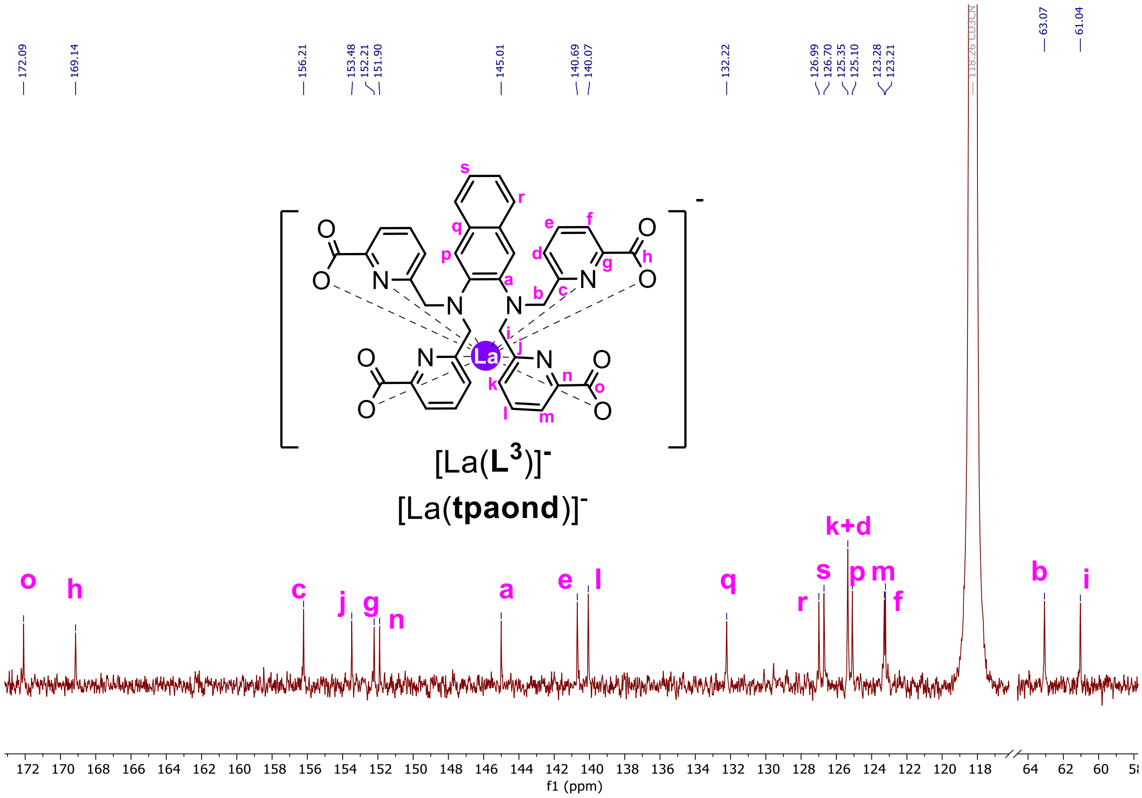

Figure S58. ^{13}C NMR spectra (126 MHz, 298 K, D_2O:CD_3CN (7:3) pD=6) of [La(**tpaond**)]$^-$.

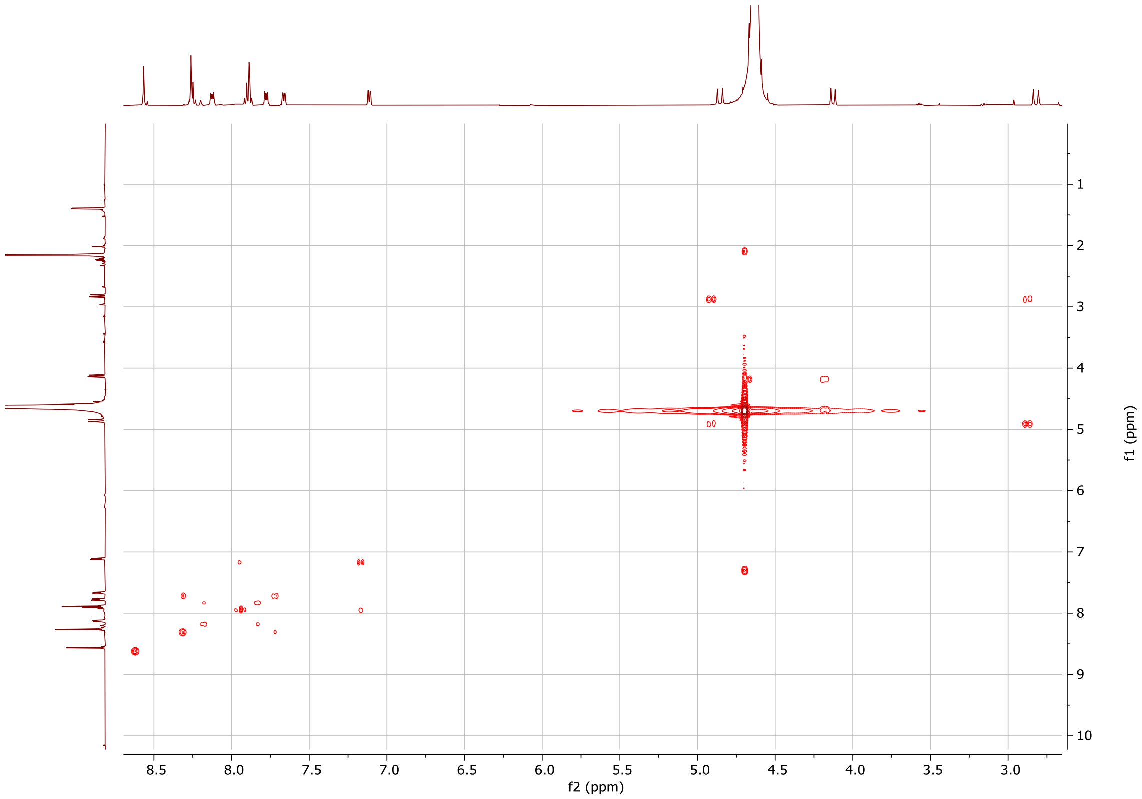

Figure S59. ^{1}H-^{1}H COSY NMR spectrum (500 MHz, 298 K, D_2O:CD_3CN (7:3) pD=6) of [La(**tpaond**)]$^-$.

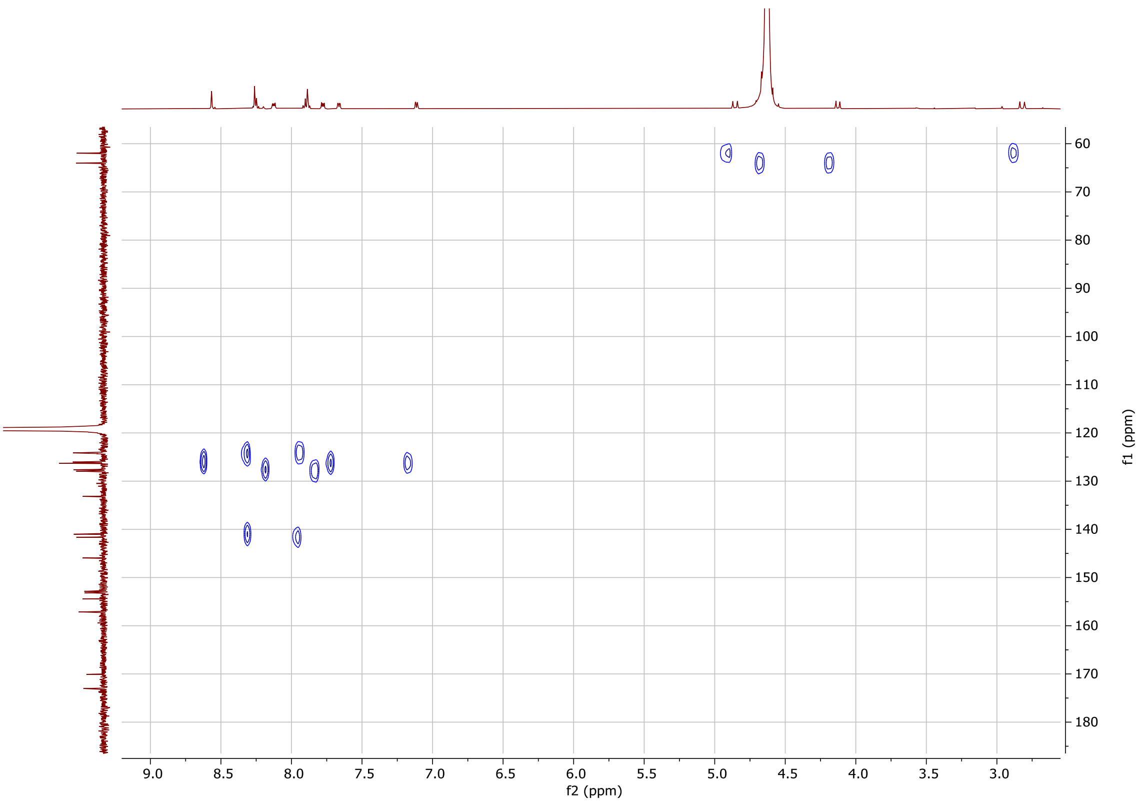

Figure S60. ^{1}H-^{13}C HSQC NMR spectrum (500 MHz, 298 K, D_2O:CD_3CN (7:3) pD=6) of [La(**tpaond**)]$^{-}$.

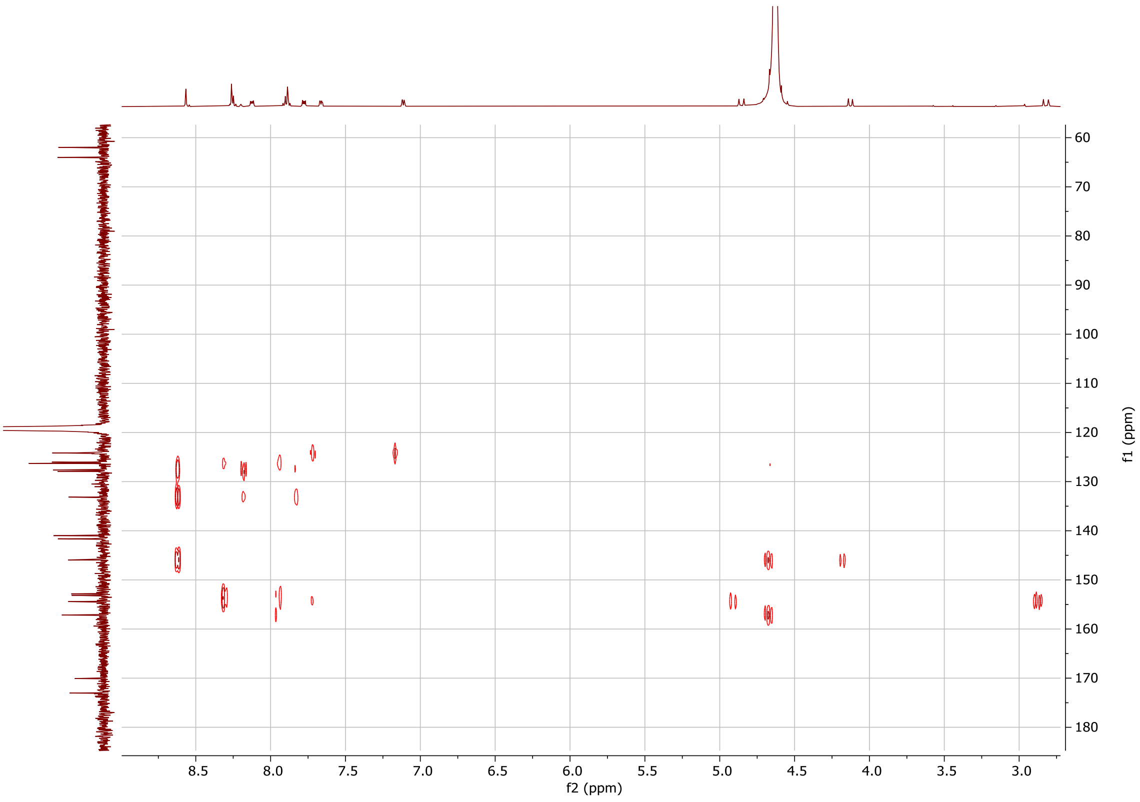

Figure S61. ^{1}H-^{13}C HMBC NMR spectrum (500 MHz, 298 K, D_2O:CD_3CN (7:3) pD=6) of [La(**tpaond**)]$^{-}$.

NMR spectra of the lanthanum(III) complex with H_4**tpamxd**.

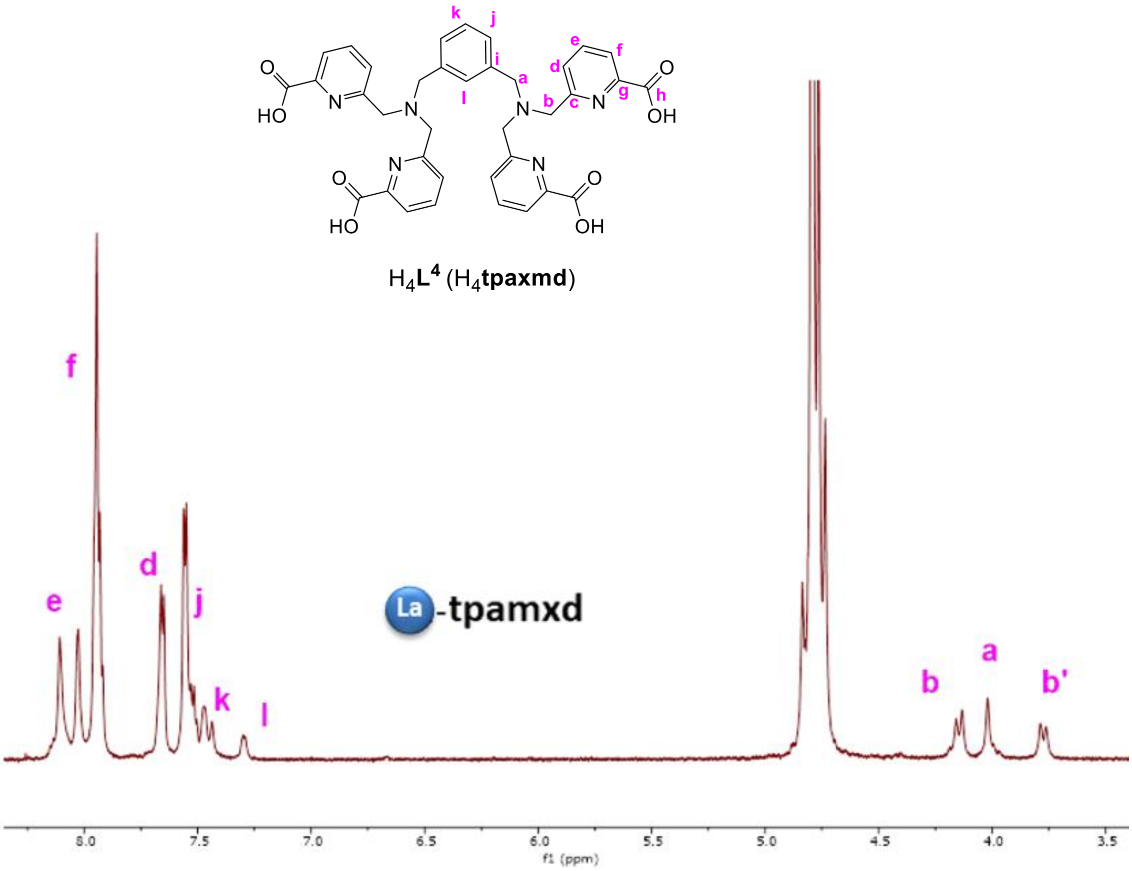

Figure S62. ^{1}H NMR spectrum (600 MHz, 298 K, D_2O pD=4.85) of the La^{3+} complex with chelator H_4**tpamxd**.

NMR spectra of the lanthanum(III) complex with H_3**tripaen**.

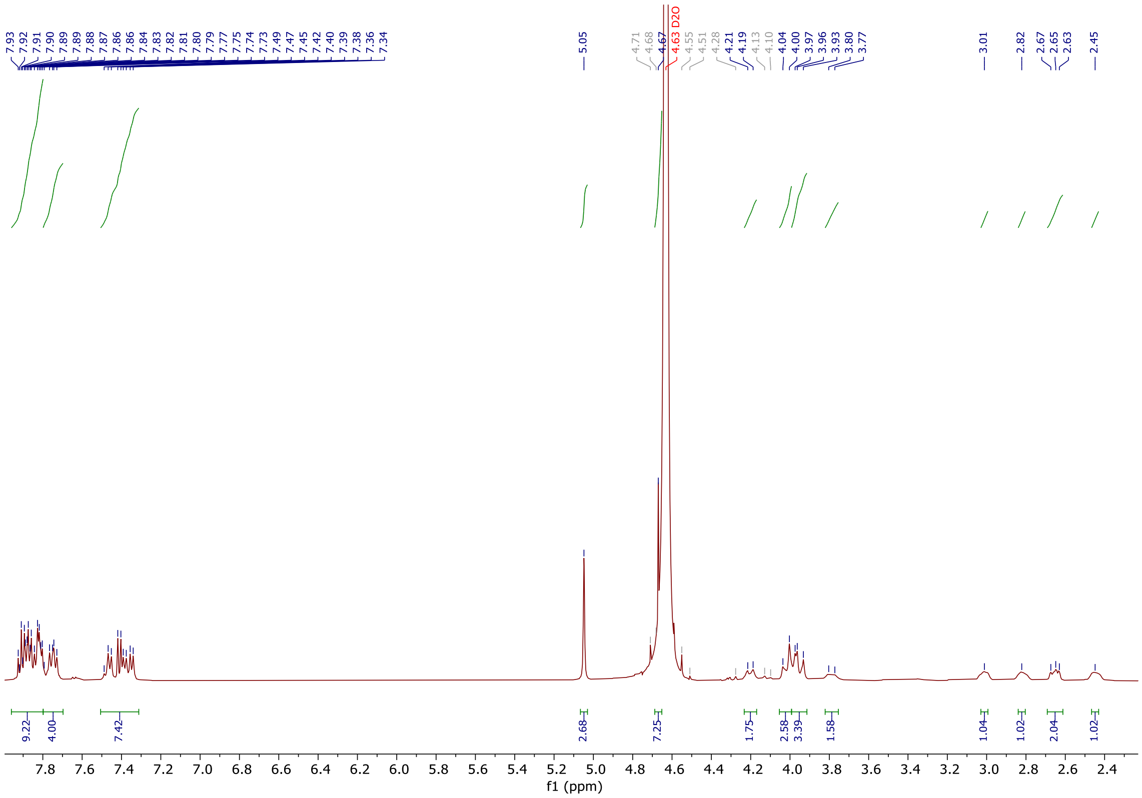

Figure S63. ^{1}H NMR spectrum (500 MHz, 298 K, D_2O pD=6) of the La^{3+} complex with chelator H_3**tripaen**.

NMR spectra of [La(**asyoctapa**)]$^-$.

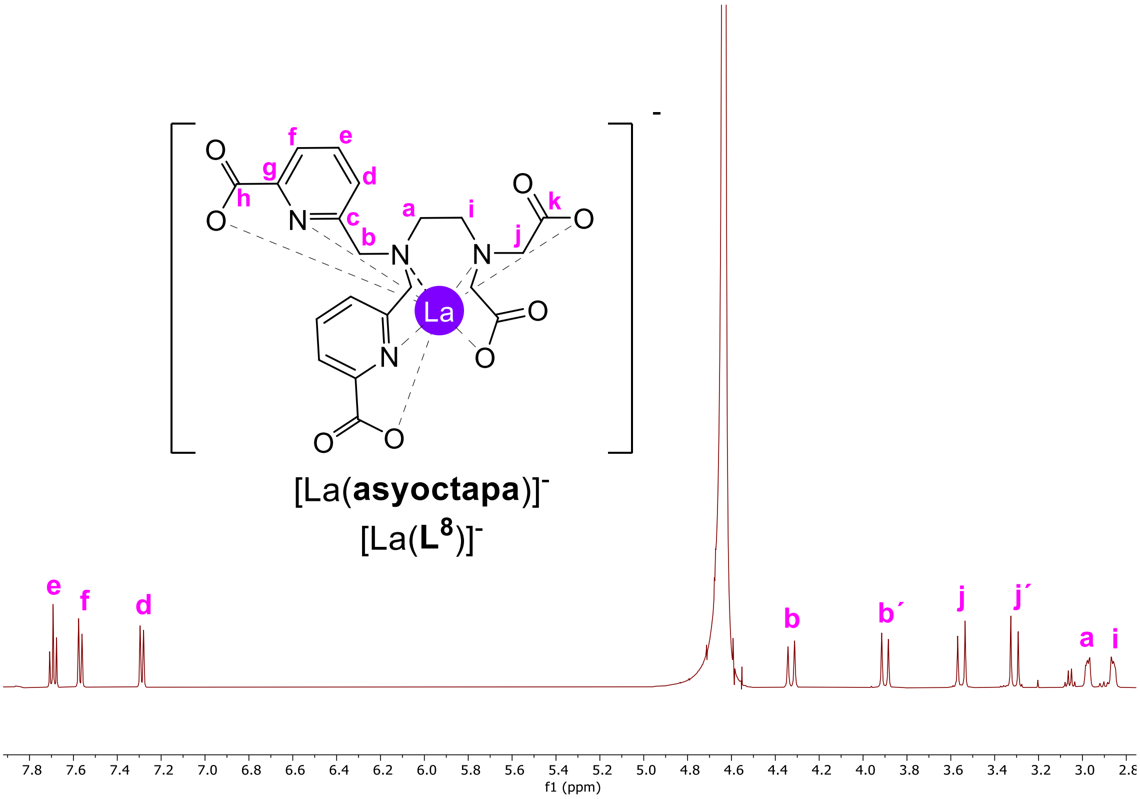

Figure S64. ^{1}H NMR spectrum (500 MHz, 298 K, D_2O pD=6) of [La(**asyoctapa**)]$^-$.

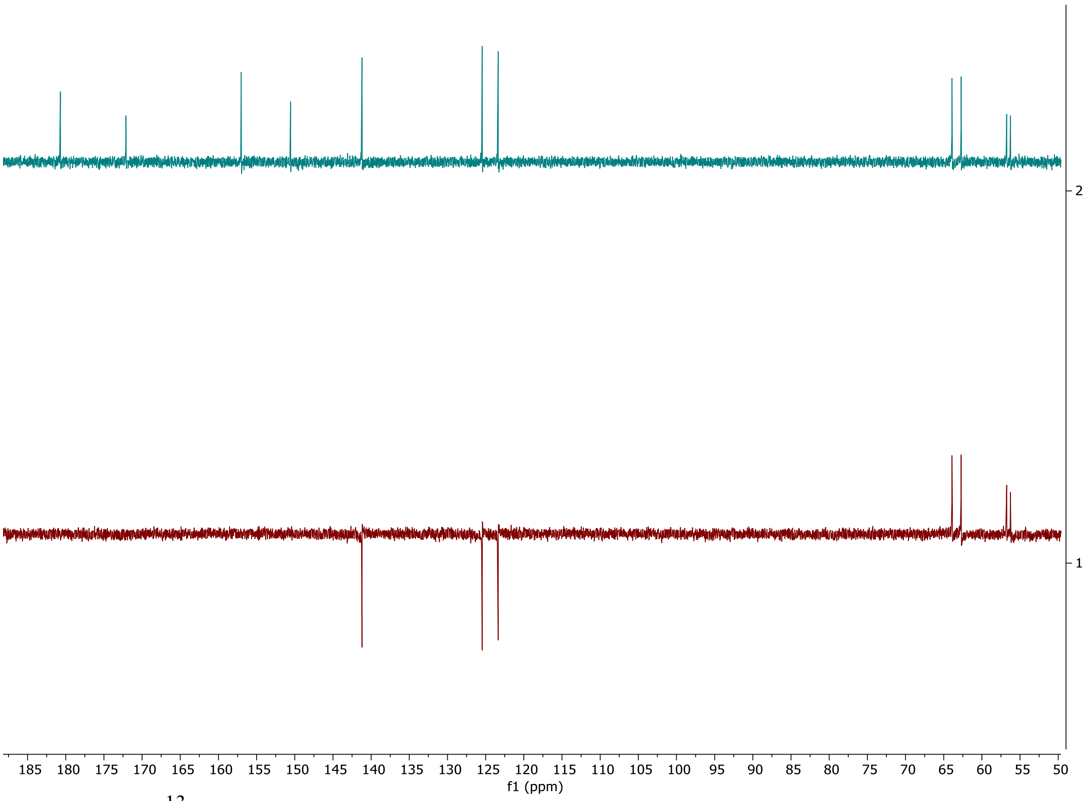

Figure S65. ^{13}C NMR and DEPT spectra (126 MHz, 298 K, D_2O pD=6) of [La(**asyoctapa**)]$^-$.

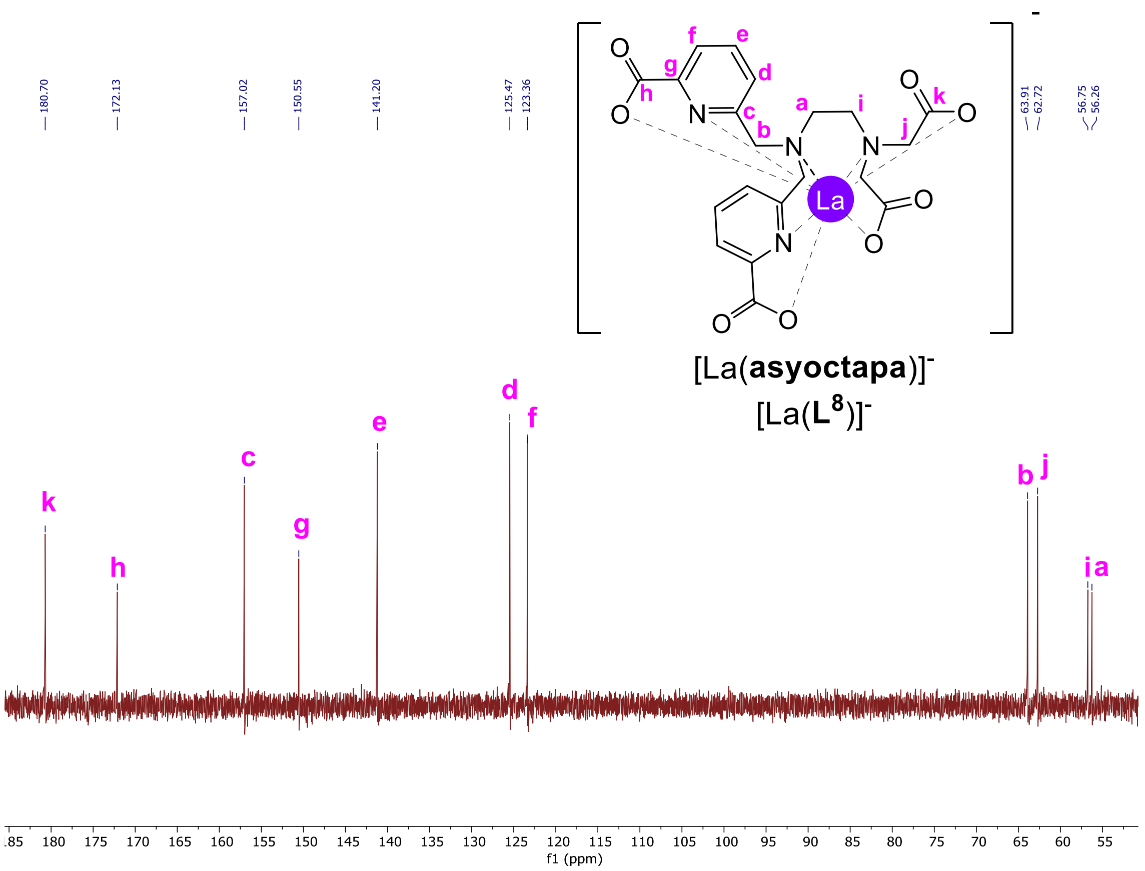

Figure S66. ^{13}C NMR spectrum (126 MHz, 298 K, D_2O pD=6) of [La(**asyoctapa**)]$^-$.

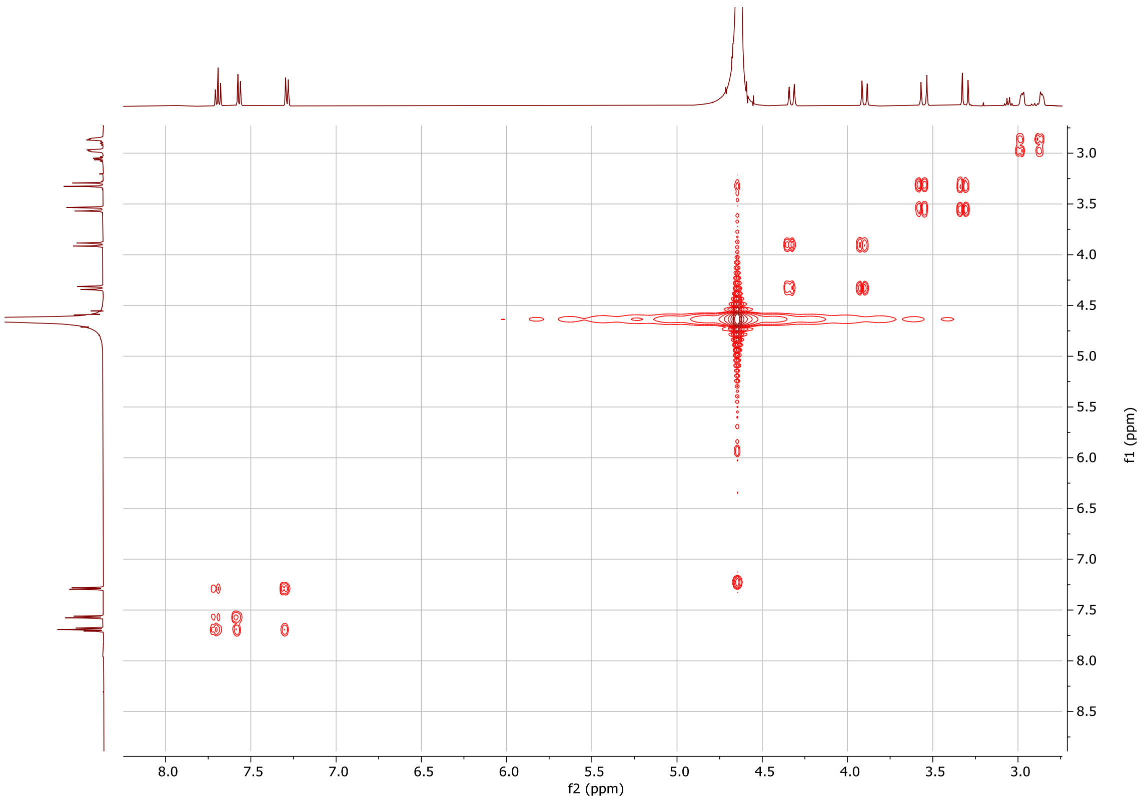

Figure S67. ^{1}H-^{1}H COSY NMR spectrum (500 MHz, 298 K, D_2O pD=6) of [La(**asyoctapa**)]$^-$.

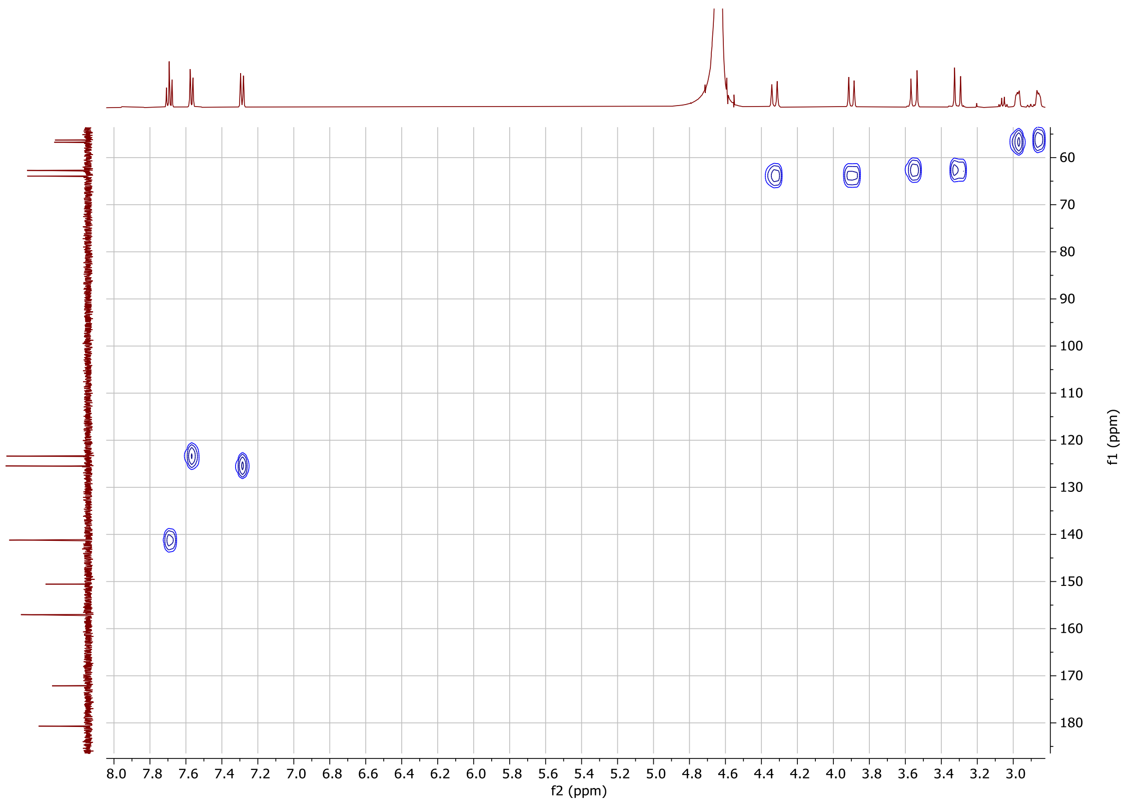

Figure S68. ^{1}H-^{13}C HSQC NMR spectrum (500 MHz, 298 K, D_2O pD=6) of [La(**asyoctapa**)]$^{-}$.

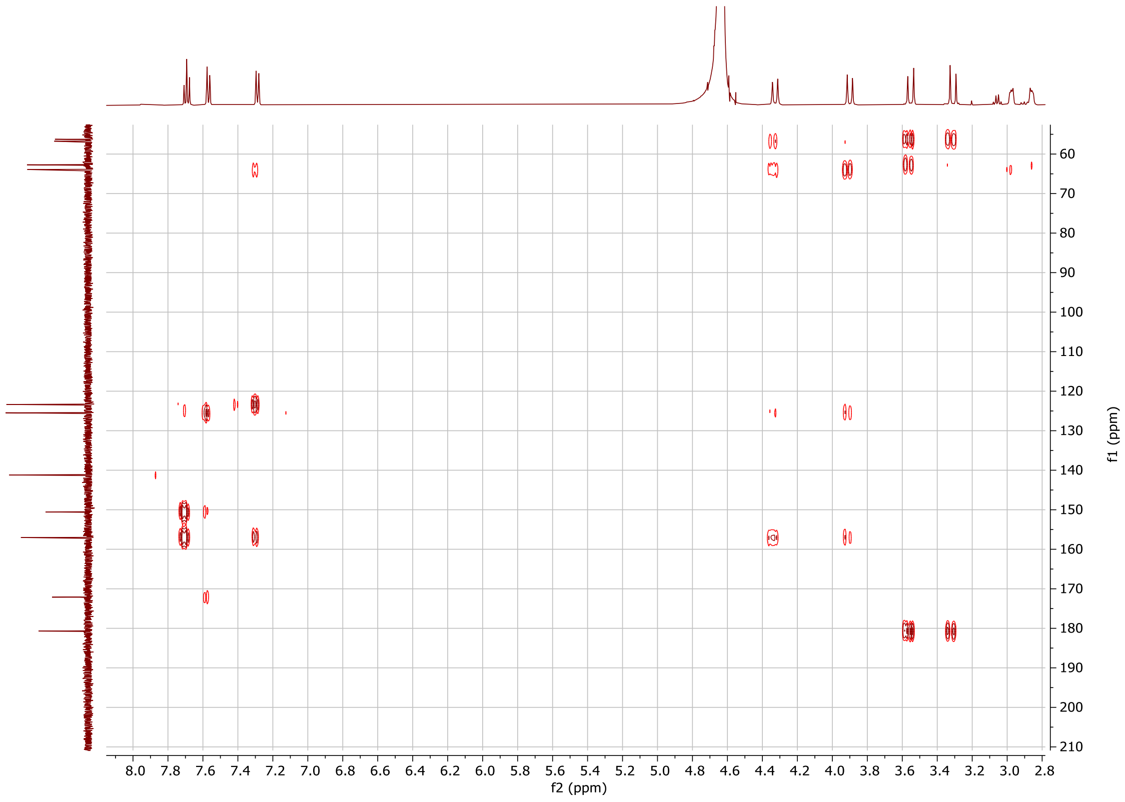

Figure S69. ^{1}H-^{13}C HMBC NMR spectrum (500 MHz, 298 K, D_2O pD=6) of [La(**asyoctapa**)]$^{-}$.

NMR Spectra of lutetium(III) complexes

NMR spectra of [Lu(**tpaen**)]$^-$.

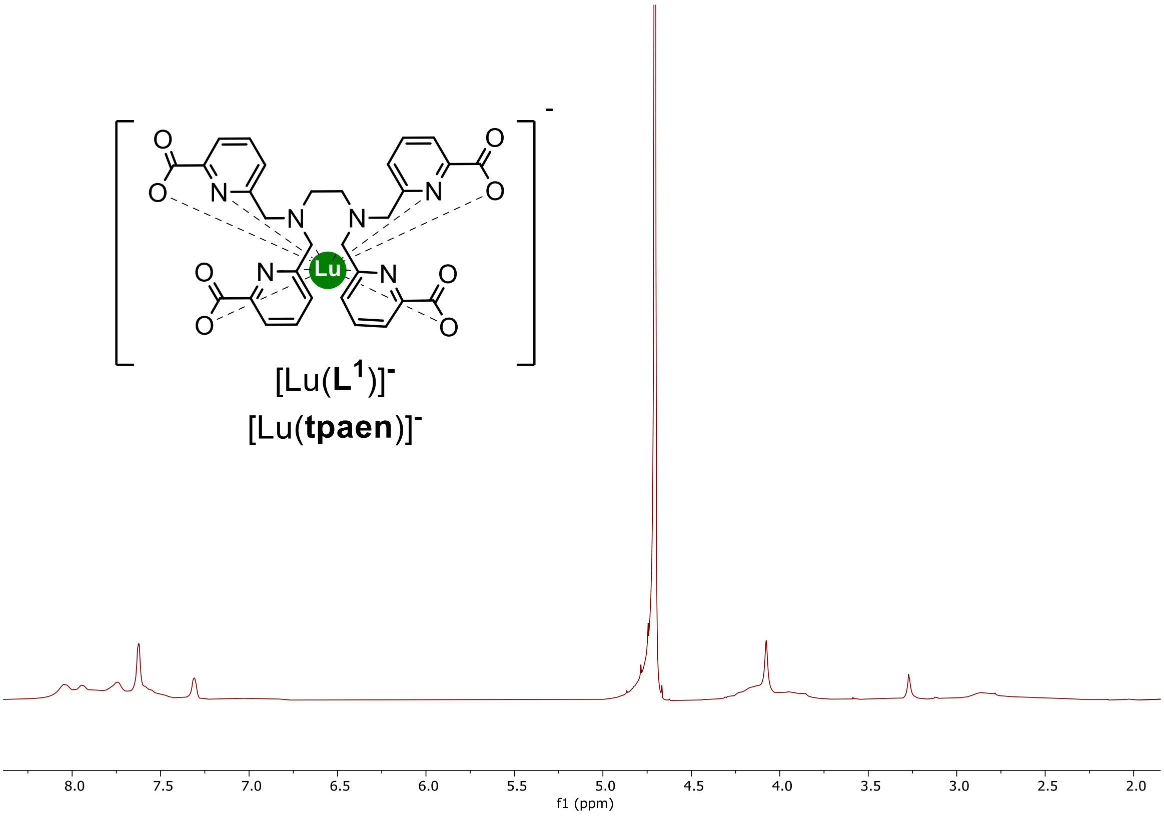

Figure S70. ^{1}H NMR spectrum (500 MHz, 298 K, D_2O pD=6) of ([Lu(**tpaen**)]$^-$.

NMR spectra of [Lu(**tpaopd**)]⁻.

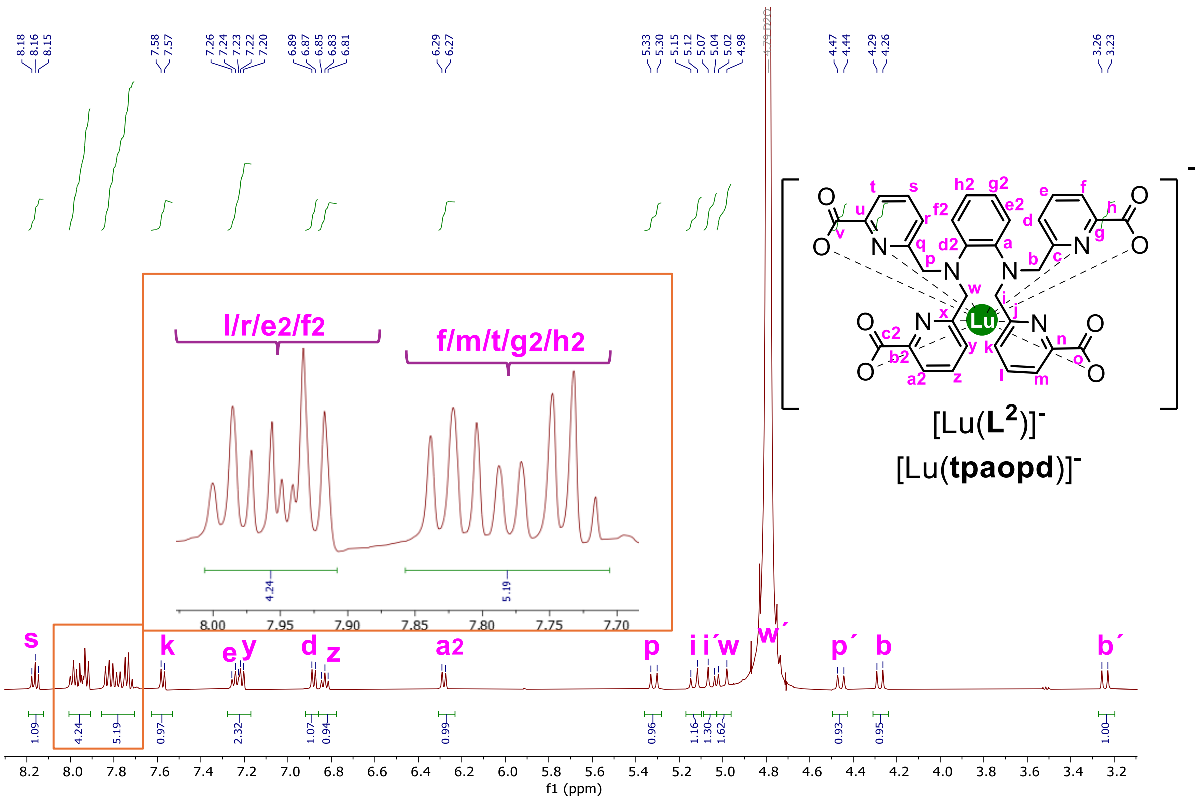

Figure S71. ^{1}H NMR spectrum (500 MHz, 298 K, D_2O pD=6) of [Lu(**tpaopd**)]$^{-}$.

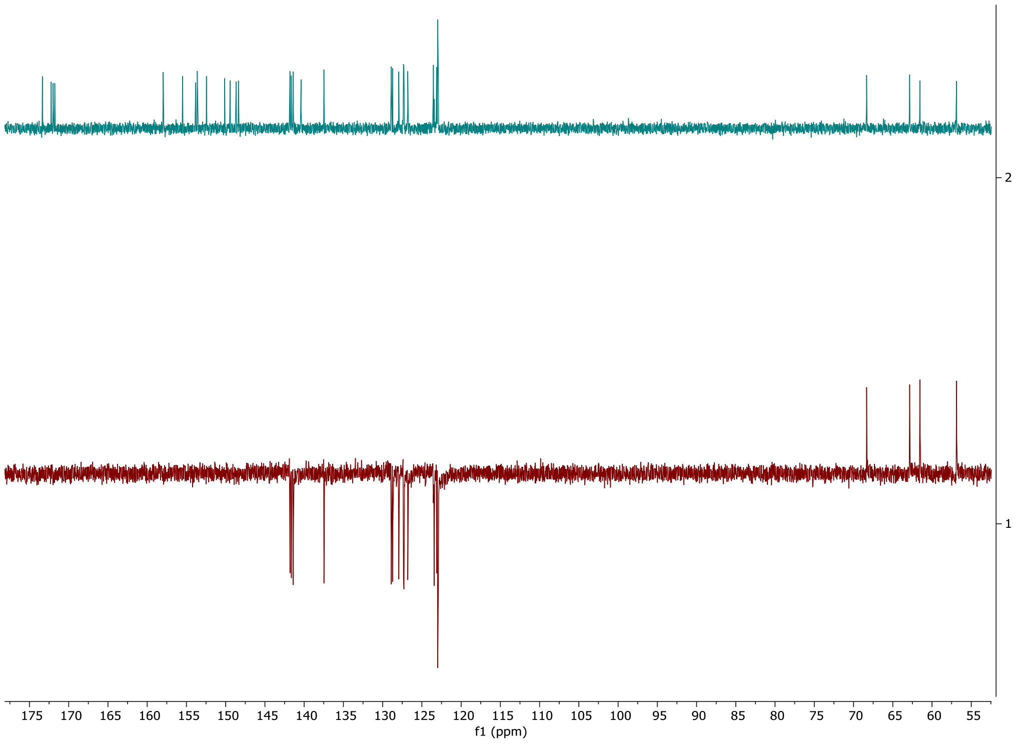

Figure S72. ^{13}C NMR and DEPT spectra (126 MHz, 298 K, D_2O pD=6) of [Lu(**tpaopd**)]$^{-}$.

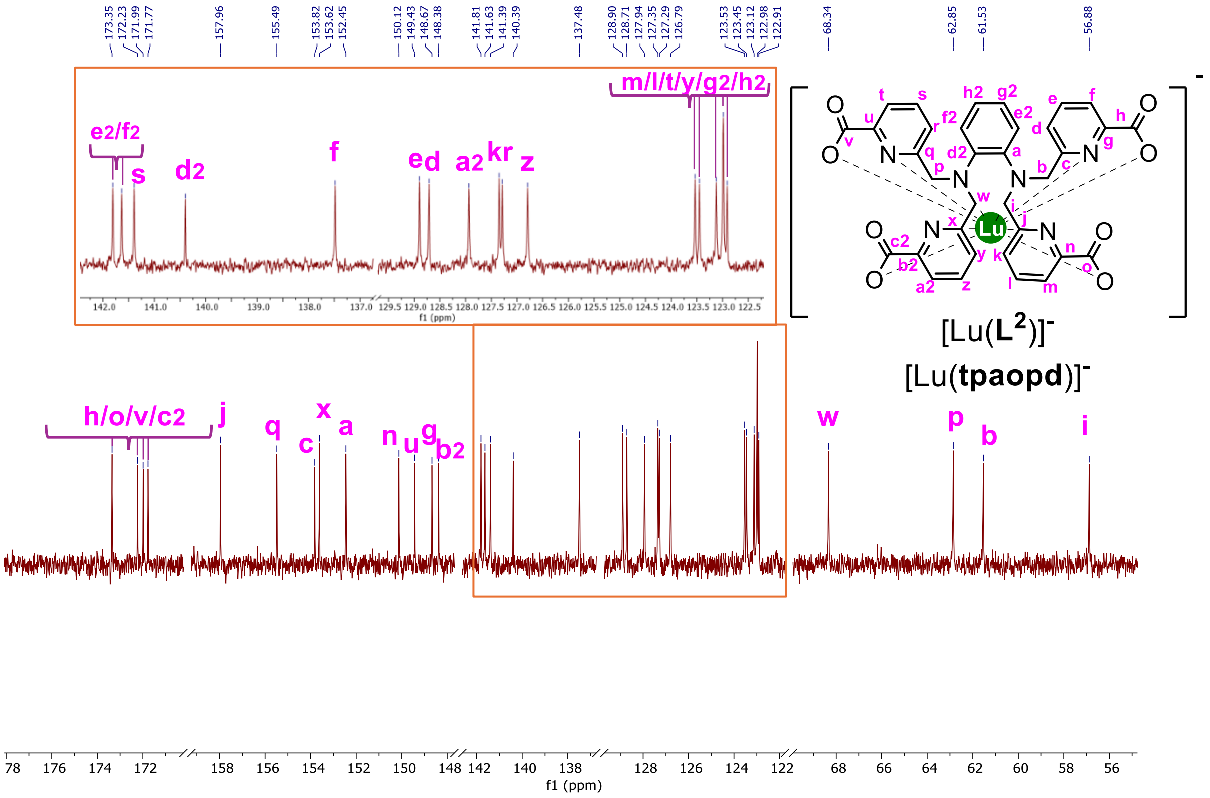

Figure S73. ^{13}C NMR spectra (126 MHz, 298 K, D_2O pD=6) of [Lu(**tpaopd**)]$^-$.

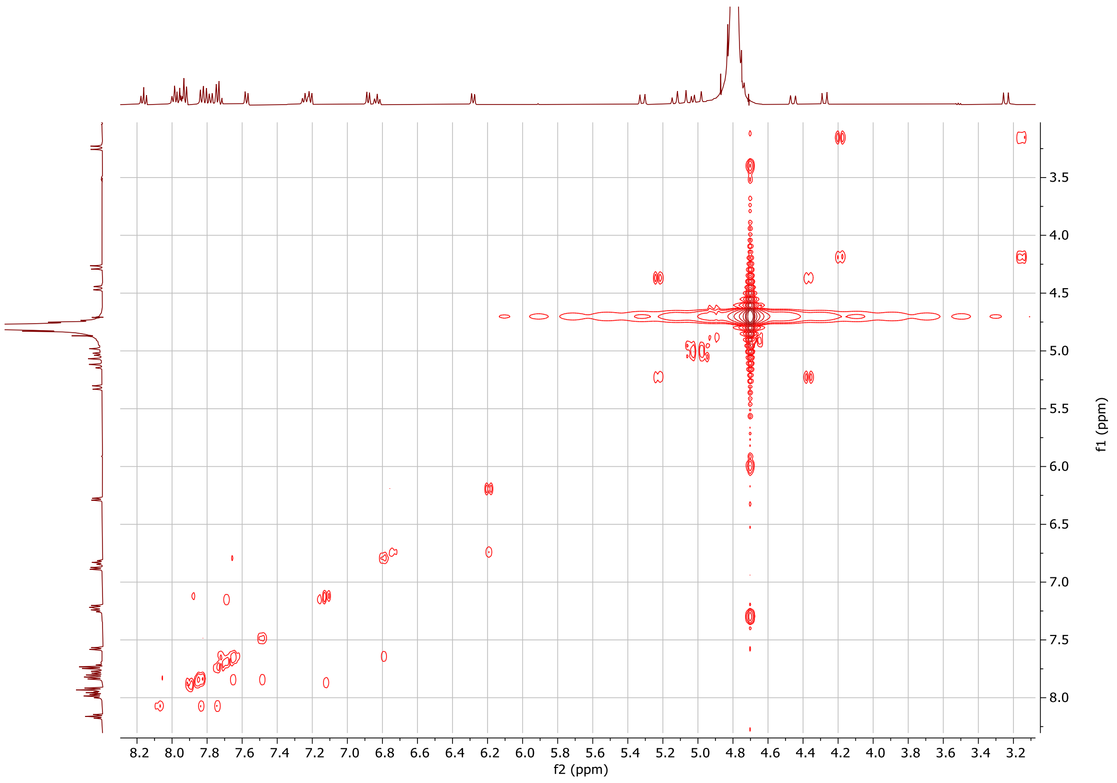

Figure S74. ^{1}H-^{1}H COSY NMR spectrum (500 MHz, 298 K, D_2O pD=6) of [Lu(**tpaopd**)]$^-$.

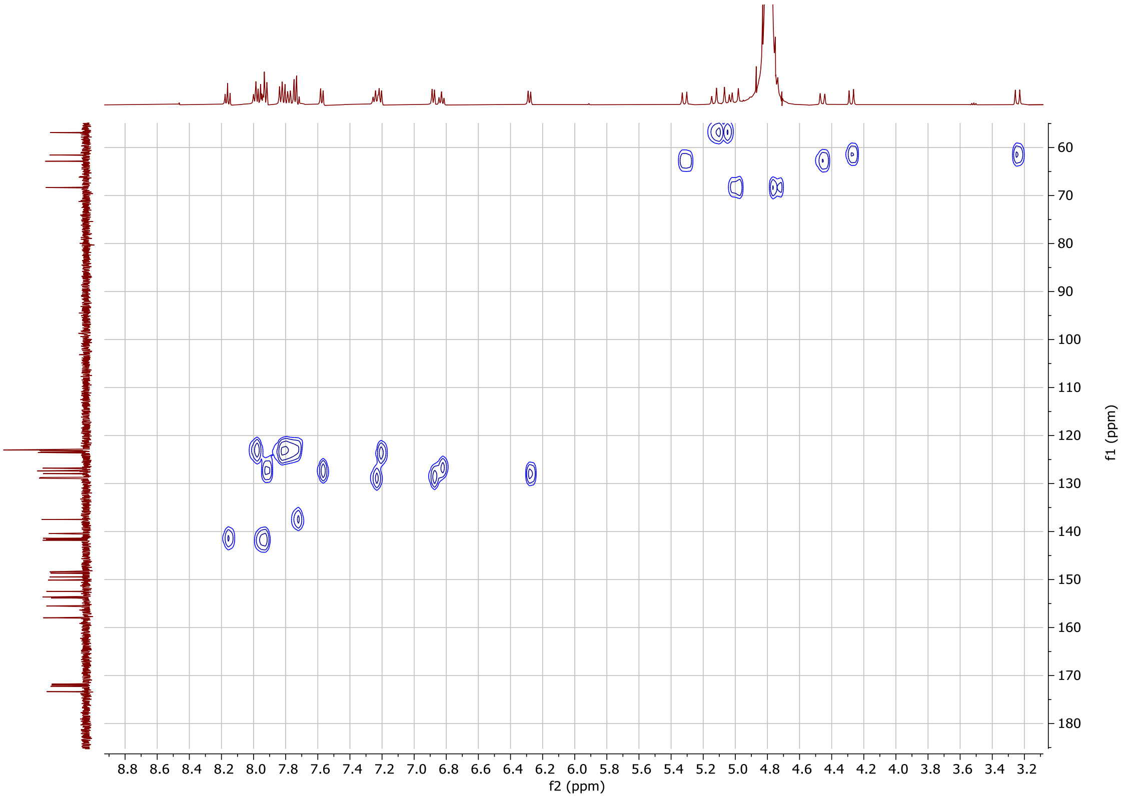

Figure S75. ^1H-^{13}C HSQC NMR spectrum (500 MHz, 298 K, D_2O pD=6) of [Lu(**tpaopd**)]$^-$.

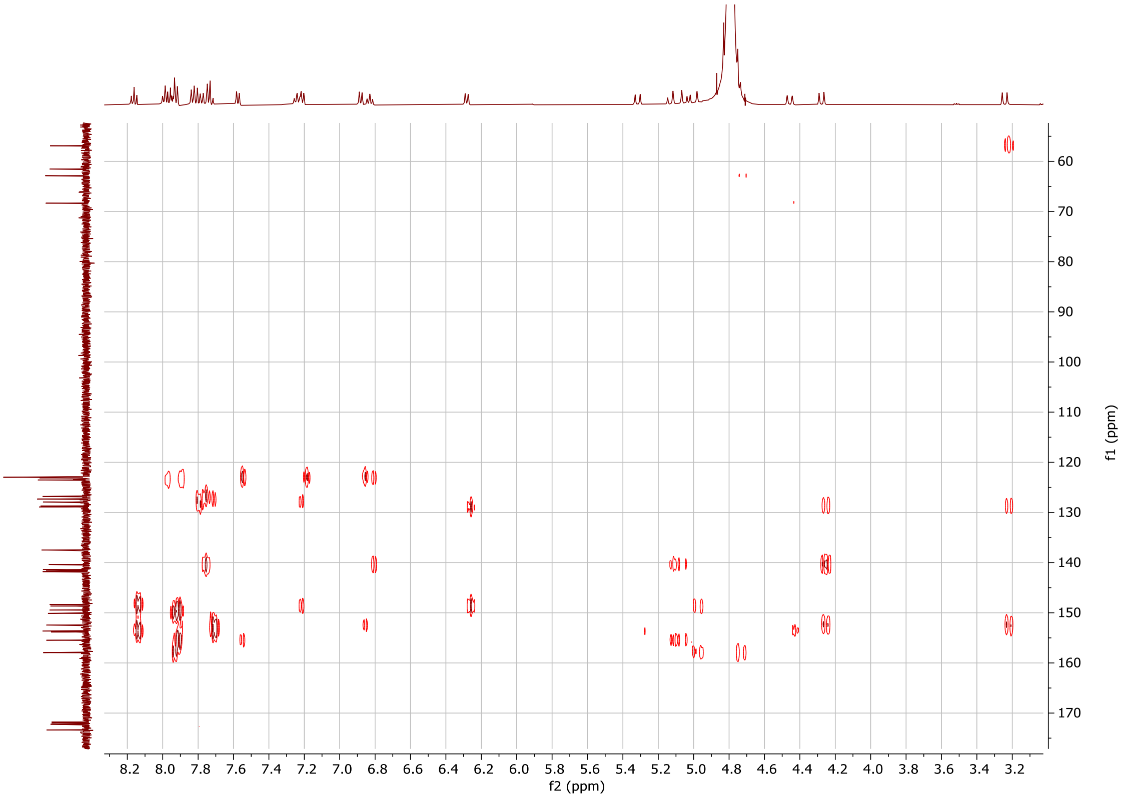

Figure S76. ^1H-^{13}C HMBC NMR spectrum (500 MHz, 298 K, D_2O pD=6) of [Lu(**tpaopd**)]$^-$.

NMR spectra of [Lu(**tpaond**)]$^-$.

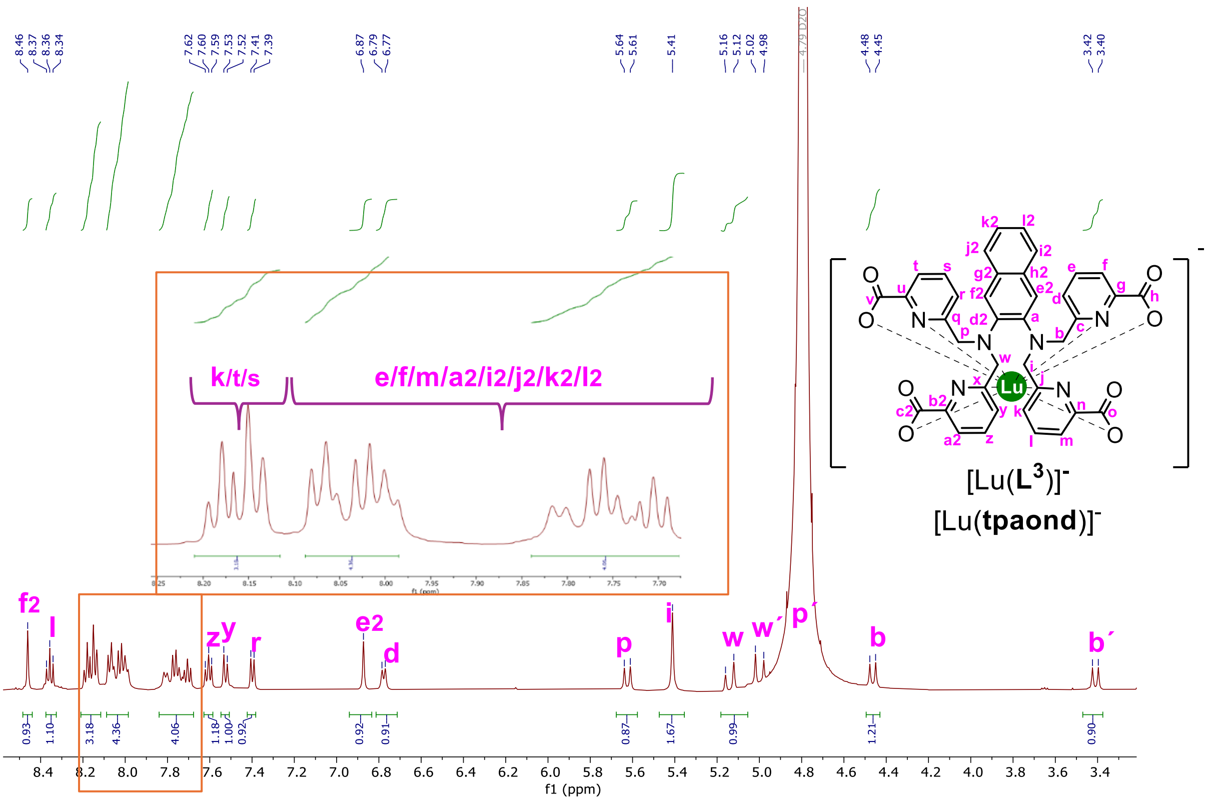

Figure S77. ^{1}H NMR spectrum (500 MHz, 298 K, D_2O:CD_3CN (7:3) pD=6) of [Lu(**tpaond**)]$^-$.

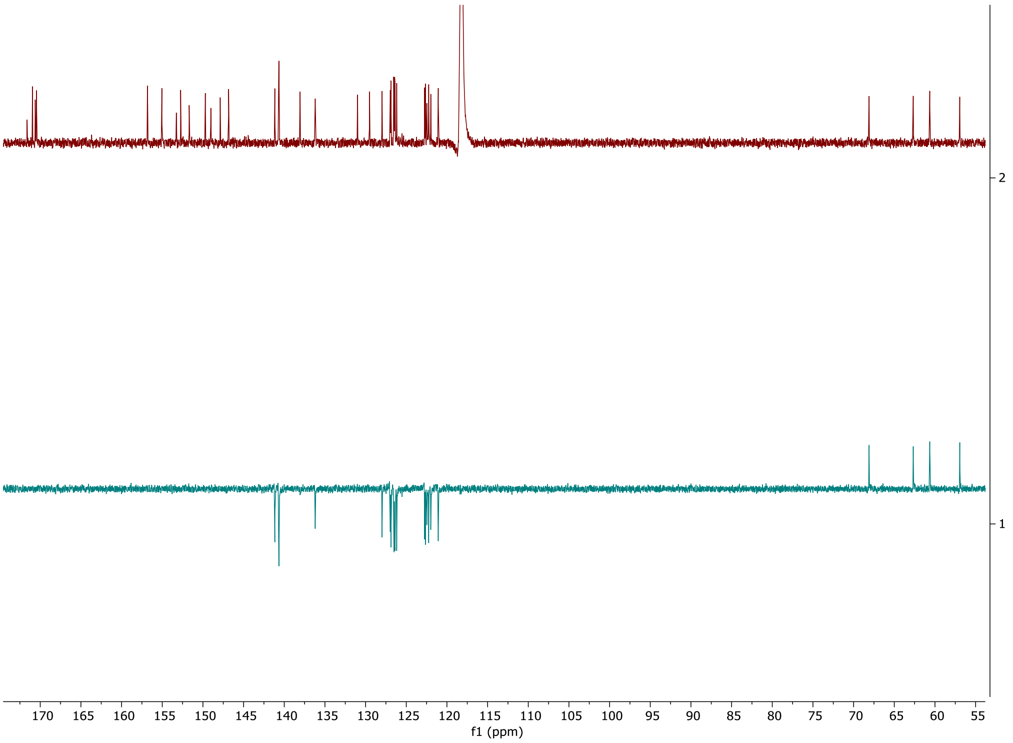

Figure S78. ^{13}C NMR and DEPT spectra (126 MHz, 298 K, D_2O:CD_3CN (7:3) pD=6) of [Lu(**tpaond**)]$^-$.

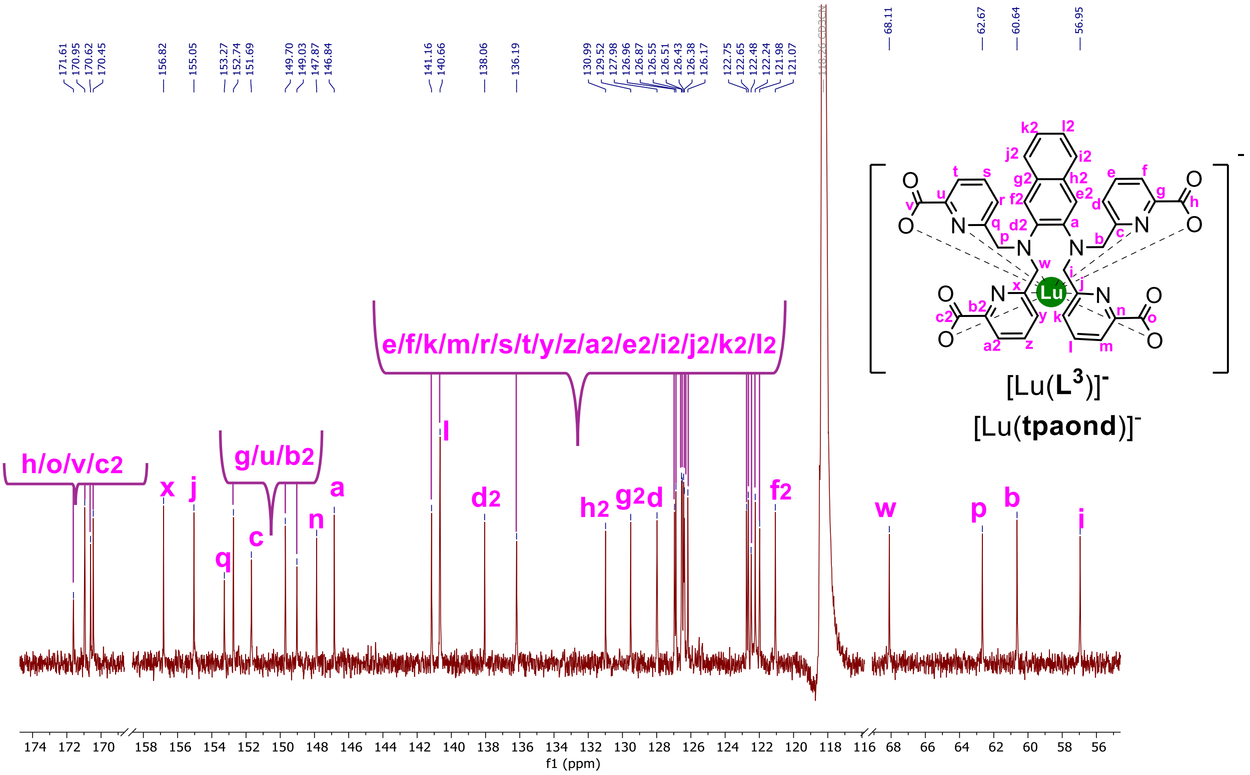

Figure S79. ^{13}C NMR spectrum (126 MHz, 298 K, D_2O:CD_3CN (7:3) pD=6) of [Lu(**tpaond**)]$^-$.

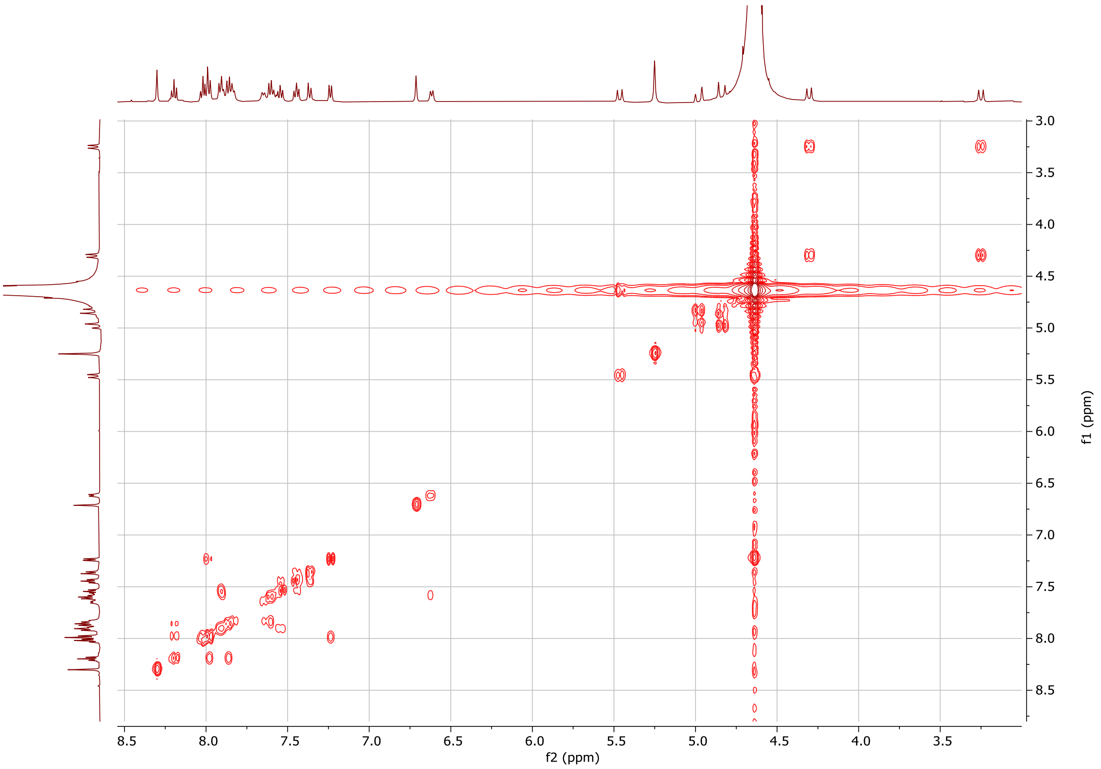

Figure S80. ^{1}H-^{1}H COSY NMR spectrum (500 MHz, 298 K, D_2O:CD_3CN (7:3) pD=6) of [Lu(**tpaond**)]$^-$.

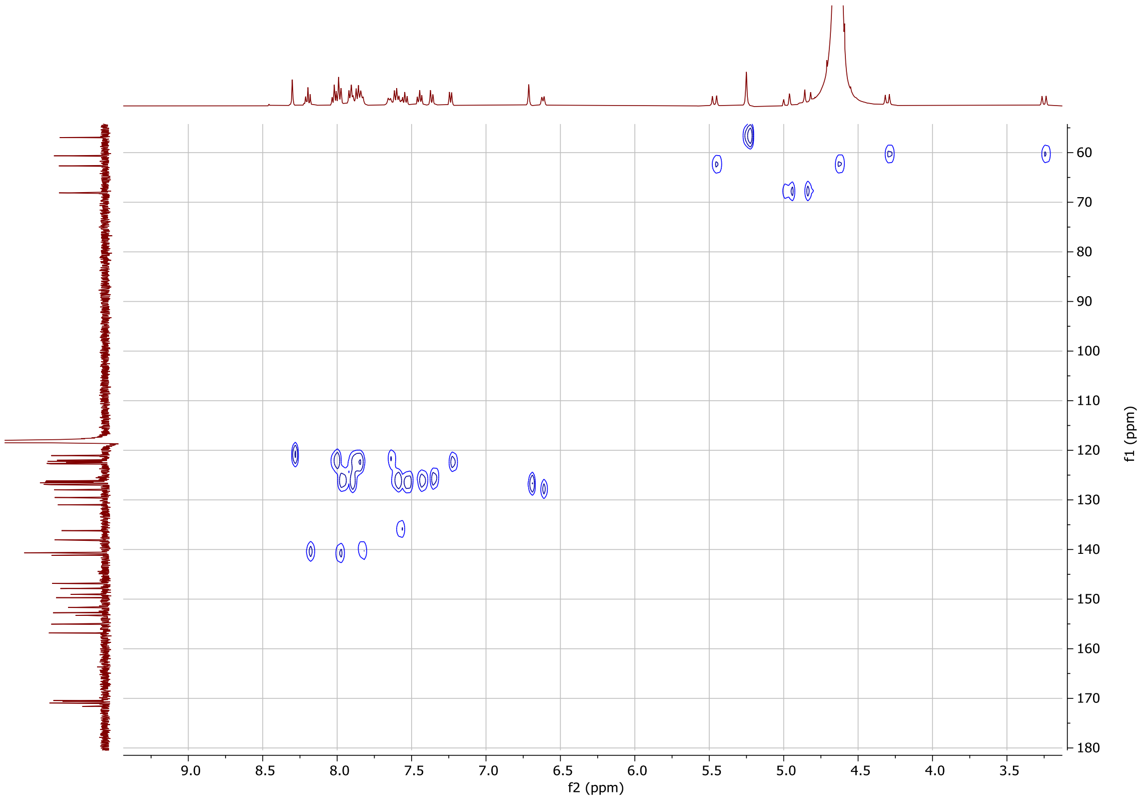

Figure S81. ^{1}H-^{13}C HSQC NMR spectrum (500 MHz, 298 K, D_2O:CD_3CN (7:3) pD=6) of [Lu(**tpaond**)]$^{-}$

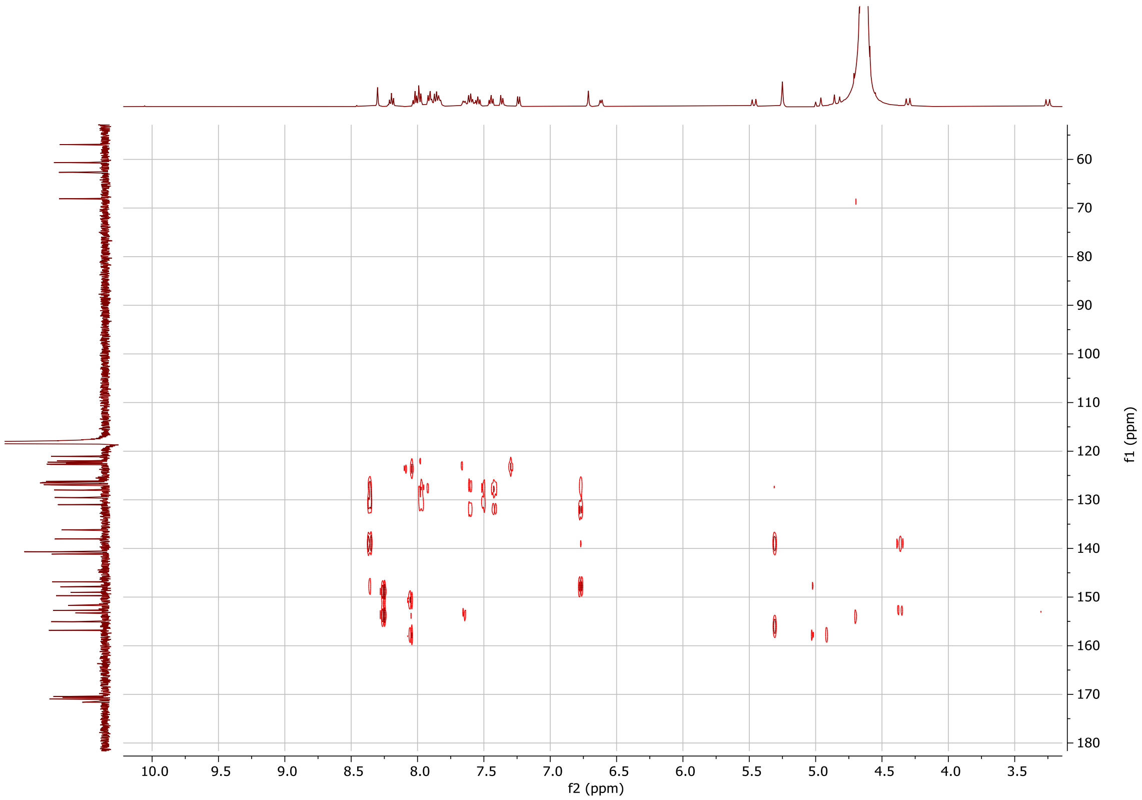

Figure S82. ^{1}H-^{13}C HMBC NMR spectrum (500 MHz, 298 K, D_2O:CD_3CN (7:3) pD=6) of [Lu(**tpaond**)]$^{-}$.

NMR spectra of the lutetium(III) complex with H_4**tpamxd**.

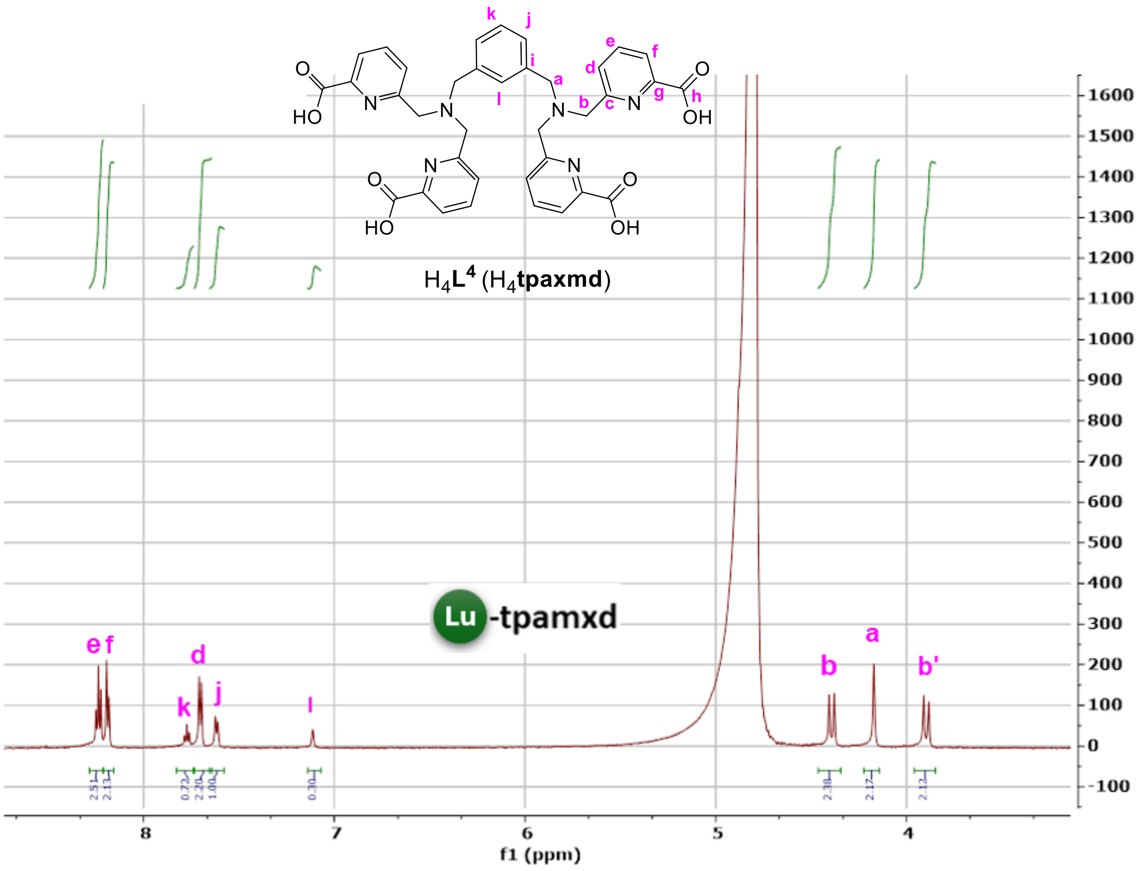

Figure S83. ^{1}H NMR spectrum (600 MHz, 298 K, D_2O pD=6.5) of the Lu^{3+} complex with chelator H_4**tpamxd**.

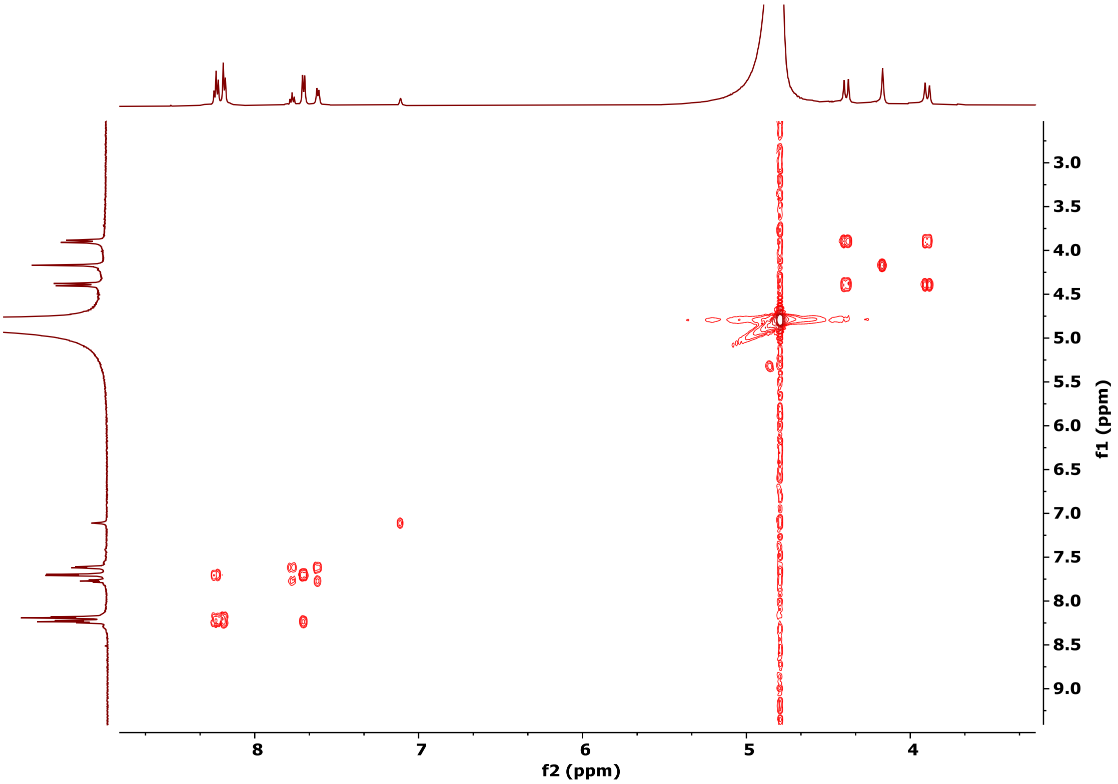

Figure S84. ^{1}H-^{1}H COSY NMR spectrum (600 MHz, 298 K, D_2O pD=6.5) of the Lu^{3+} complex with chelator H_4**tpamxd**.

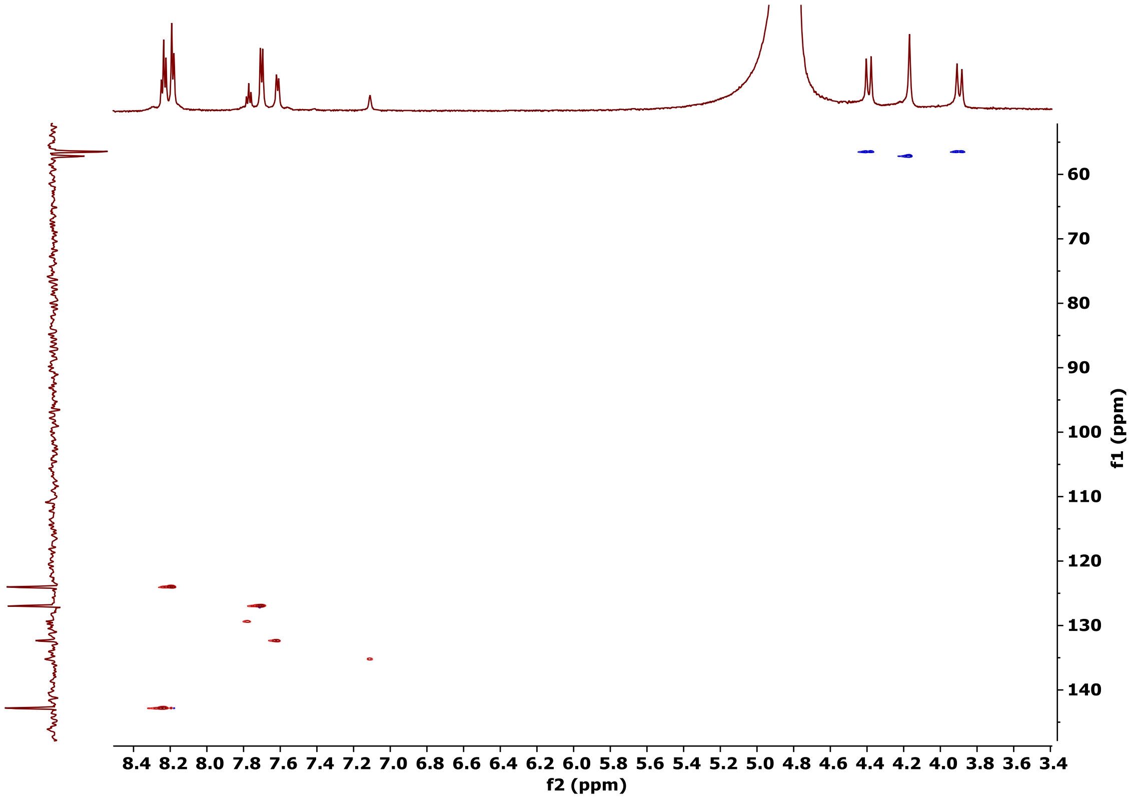

Figure S85. ^{1}H-DEPT HSQC NMR spectrum (600 MHz, 298 K, D_2O pD=6.5) of the Lu^{3+} complex with chelator H_4**tpamxd**.

NMR spectra of the lutetium(III) complex with H_4**tpapxd**.

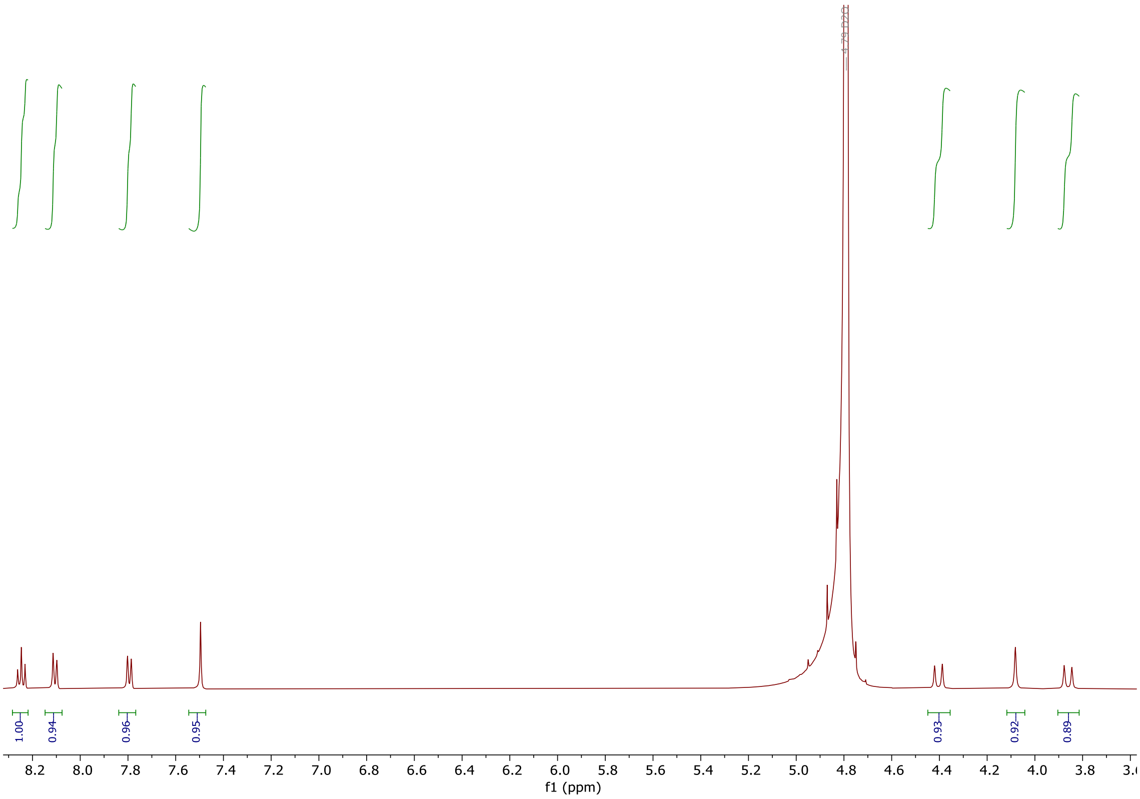

Figure S86. ^{1}H NMR spectrum (500 MHz, 298 K, D_2O pD=6) of the Lu^{3+} complex with chelator H_4**tpapxd**.

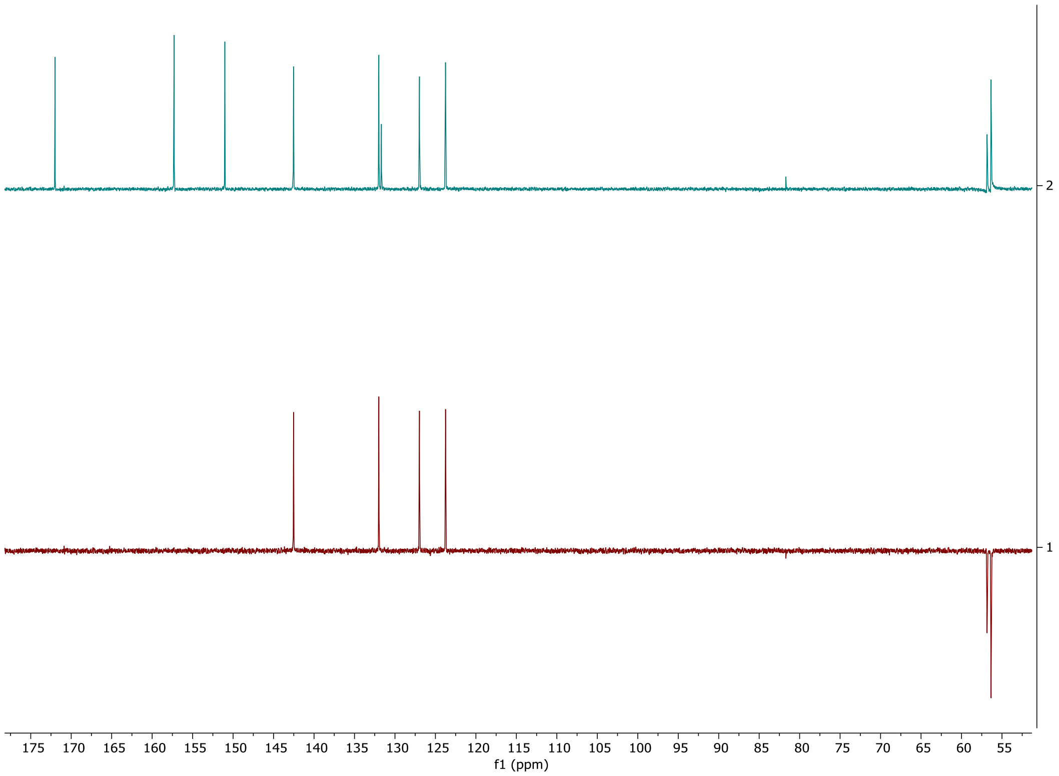

Figure S87. ^{13}C NMR and DEPT spectra of the complex of Lu^{3+} with chelator H_4**tpapxd** (126 MHz, 298 K, D_2O).

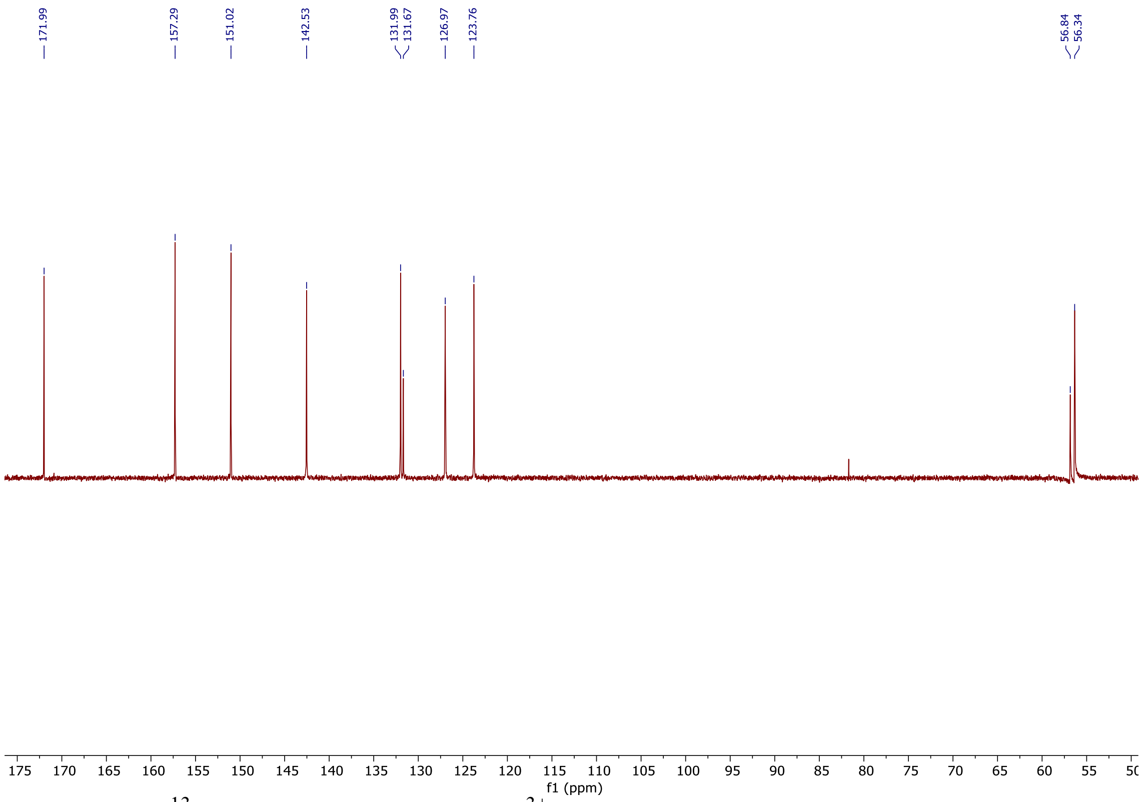

Figure S88. ^{13}C NMR spectra of the Lu^{3+} complex with chelator H_4**tpapxd** (126 MHz, 298 K, D_2O).

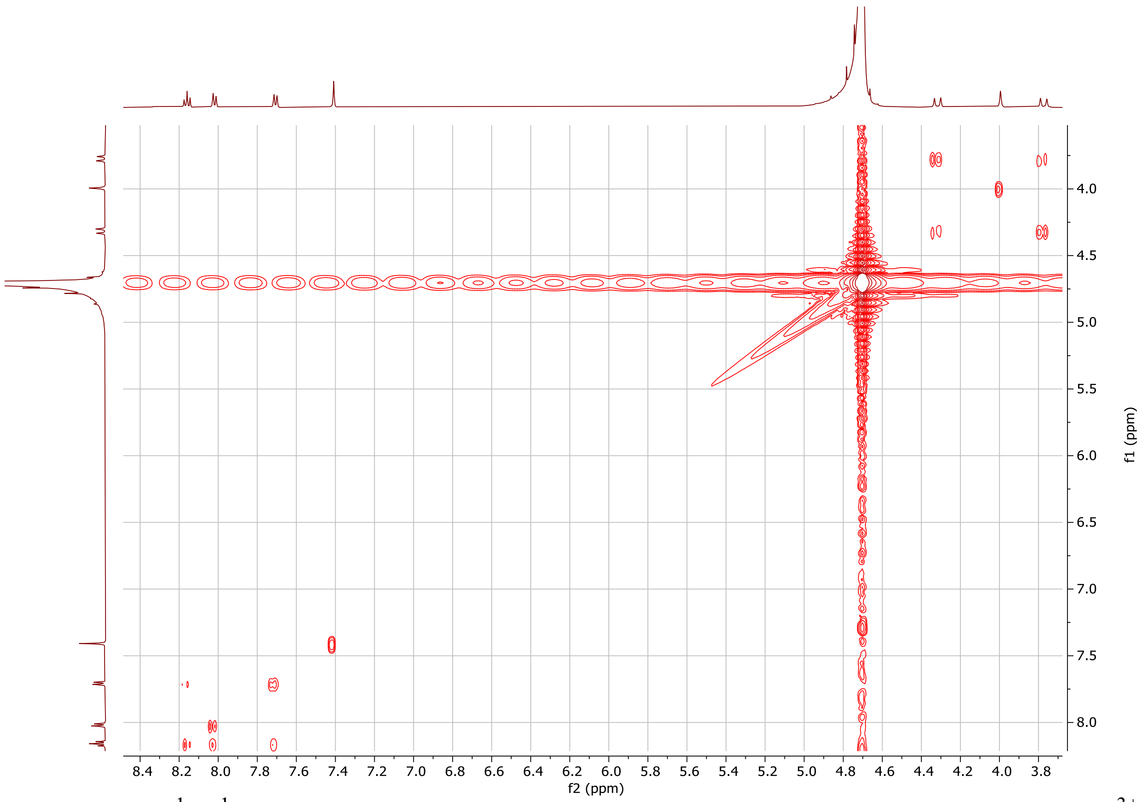

Figure S89. ^1H-^1H COSY NMR spectrum (500 MHz, 298 K, D_2O pD=6) of the Lu^{3+} complex with chelator H_4**tpapxd**.

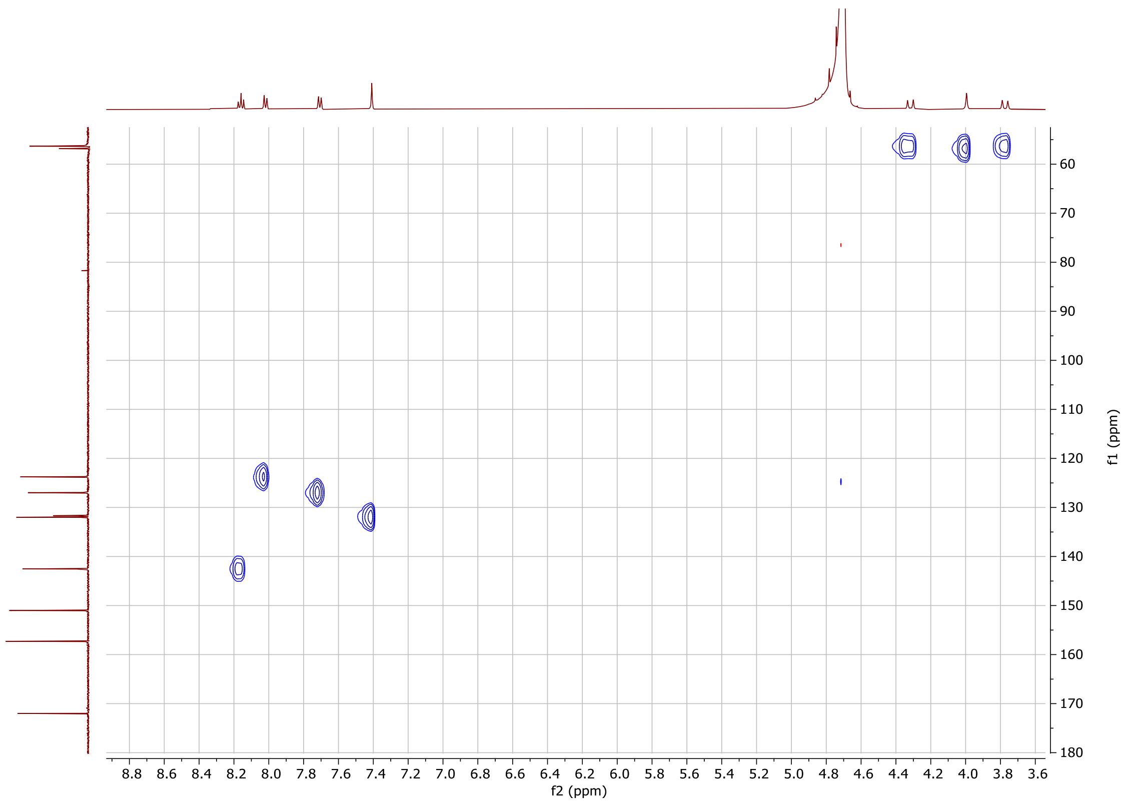

Figure S90. ^{1}H-^{13}C HSQC NMR spectrum (500 MHz, 298 K, D_2O pD=6) of the Lu^{3+} complex with chelator H_4**tpapxd**.

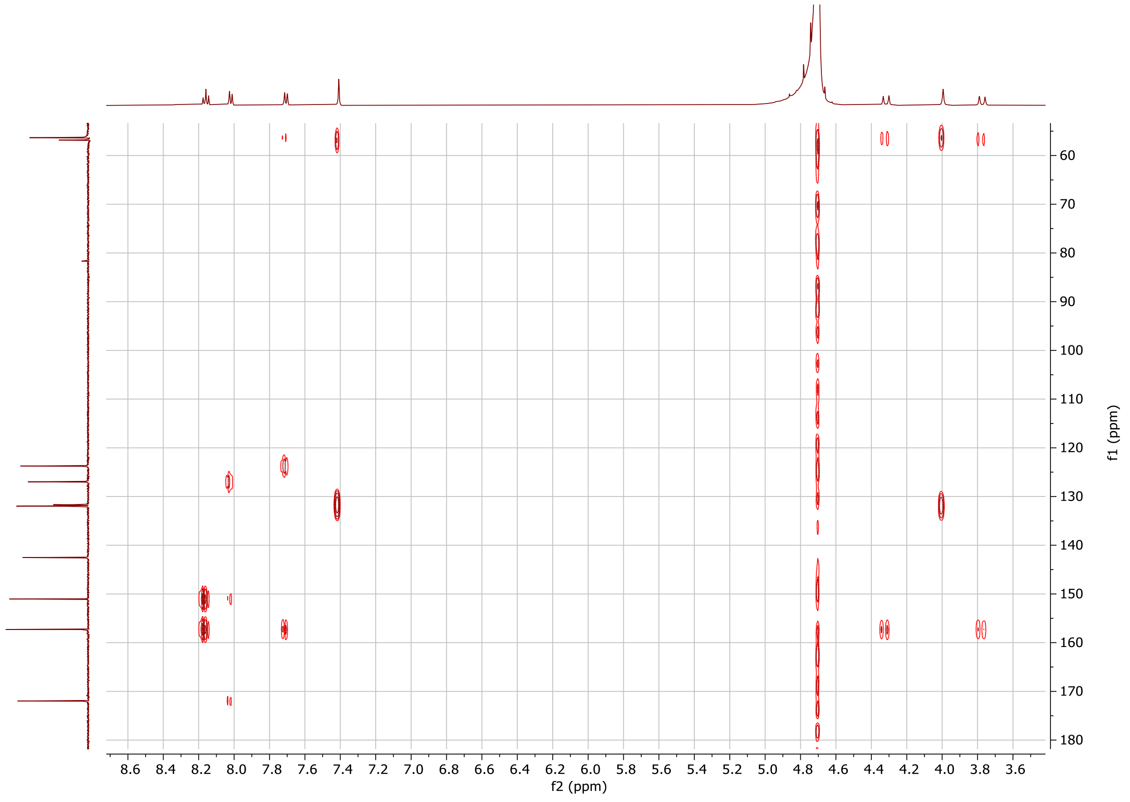

Figure S91. ^{1}H-^{13}C HMBC NMR spectrum (500 MHz, 298 K, D_2O pD=6) of the Lu^{3+} complex with chelator H_4**tpapxd**.

NMR spectra of the lutetium(III) complex with H_3**tripaen**.

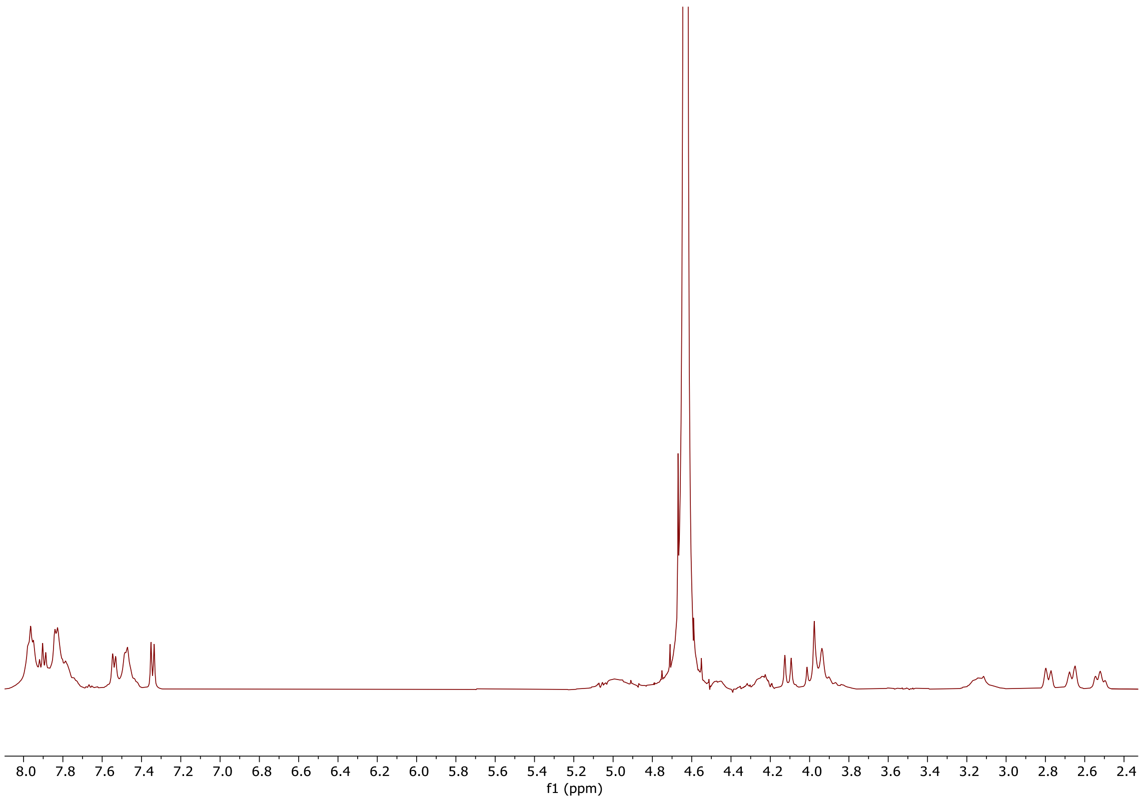

Figure S92. ^{1}H NMR spectrum (500 MHz, 298 K, D_2O pD=6) of the Lu^{3+} complex with chelator H_3**tripaen**.

NMR spectra of [Lu(**asyoctapa**)]$^-$.

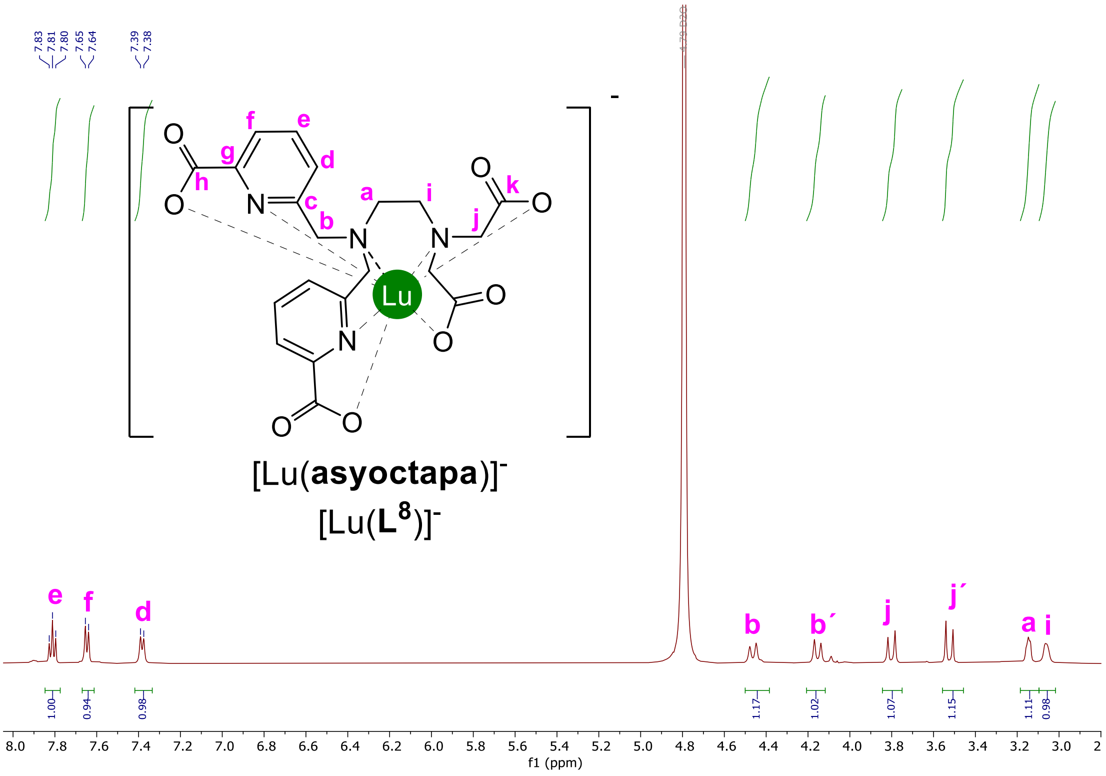

Figure S93. ^{1}H NMR spectrum (500 MHz, 298 K, D_2O pD=6) of [Lu(**asyoctapa**)]$^-$.

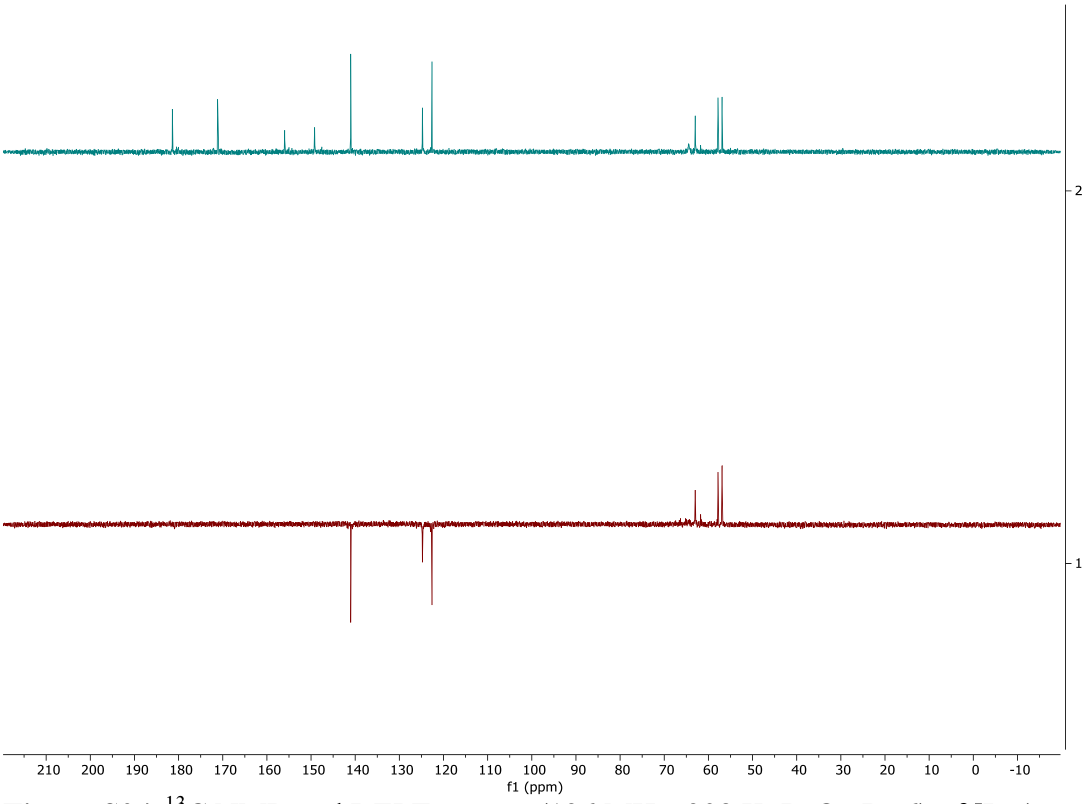

Figure S94. ^{13}C NMR and DEPT spectra (126 MHz, 298 K, D_2O pD=6) of [Lu(**asyoctapa**)]$^-$.

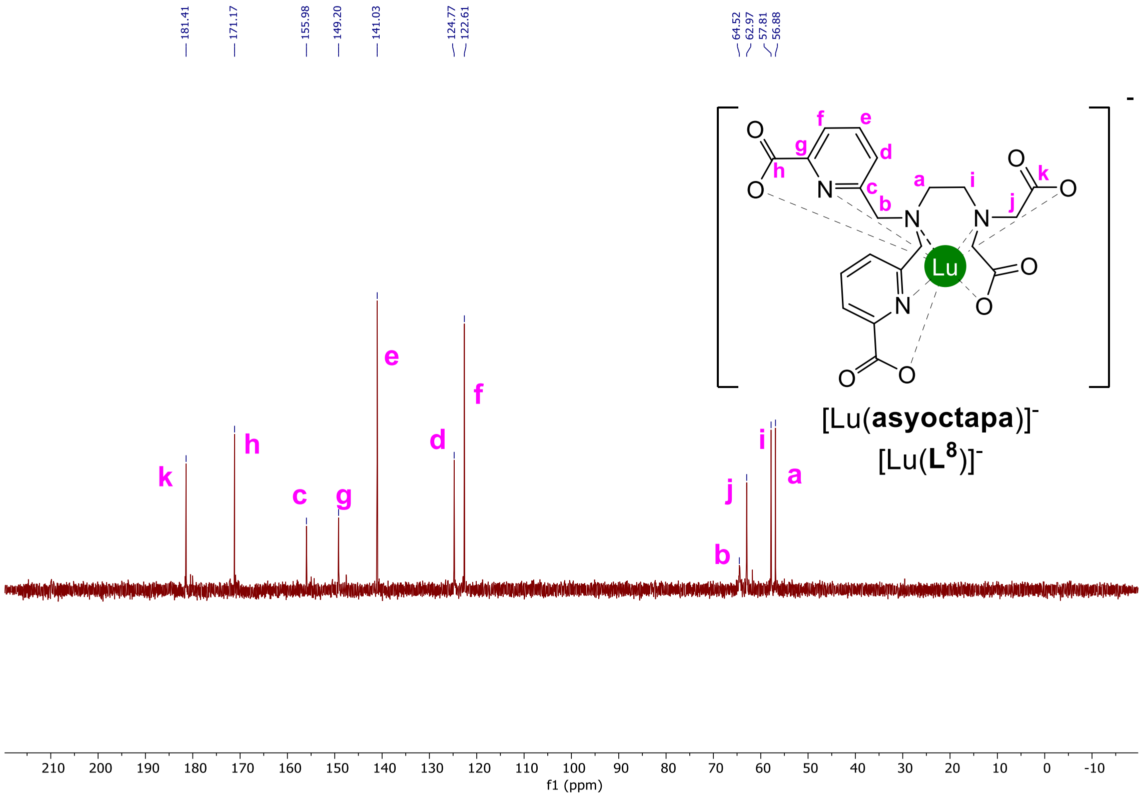

Figure S95. ^{13}C NMR spectrum (126 MHz, 298 K, D_2O pD=6) of [Lu(**asyoctapa**)]$^-$.

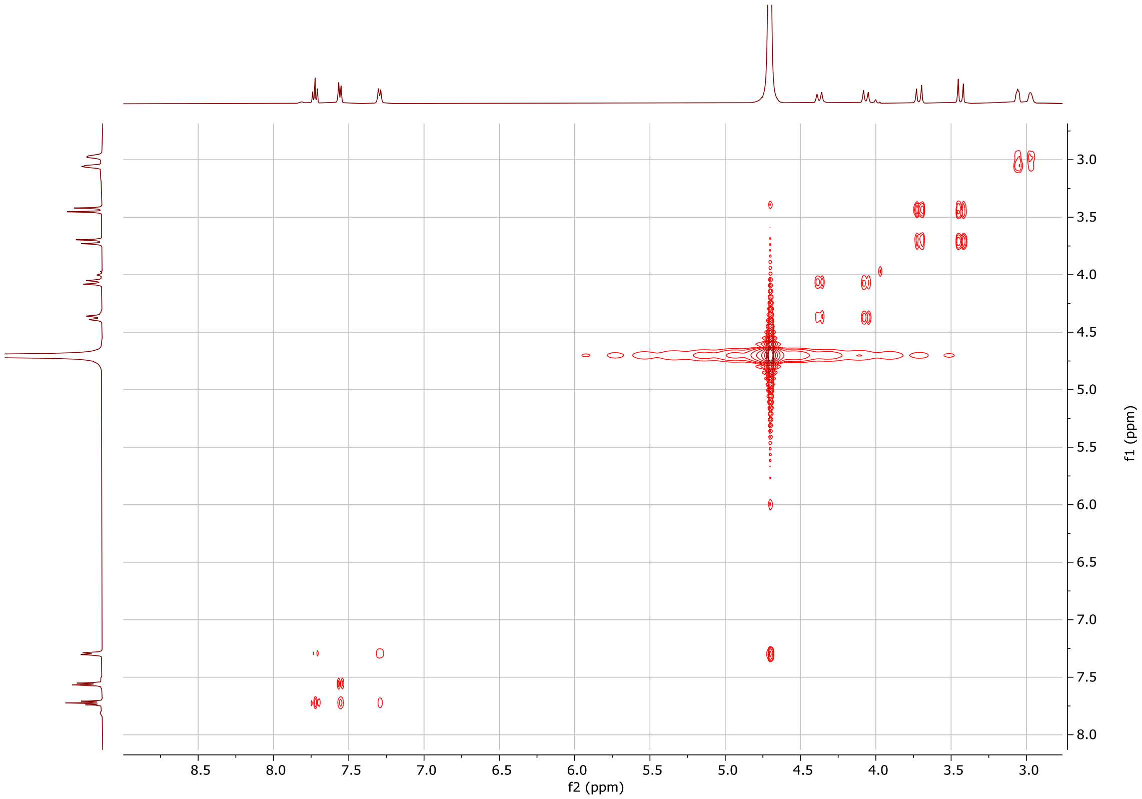

Figure S96. ^{1}H-^{1}H COSY NMR spectrum (500 MHz, 298 K, D_2O pD=6) of [Lu(**asyoctapa**)]$^-$.

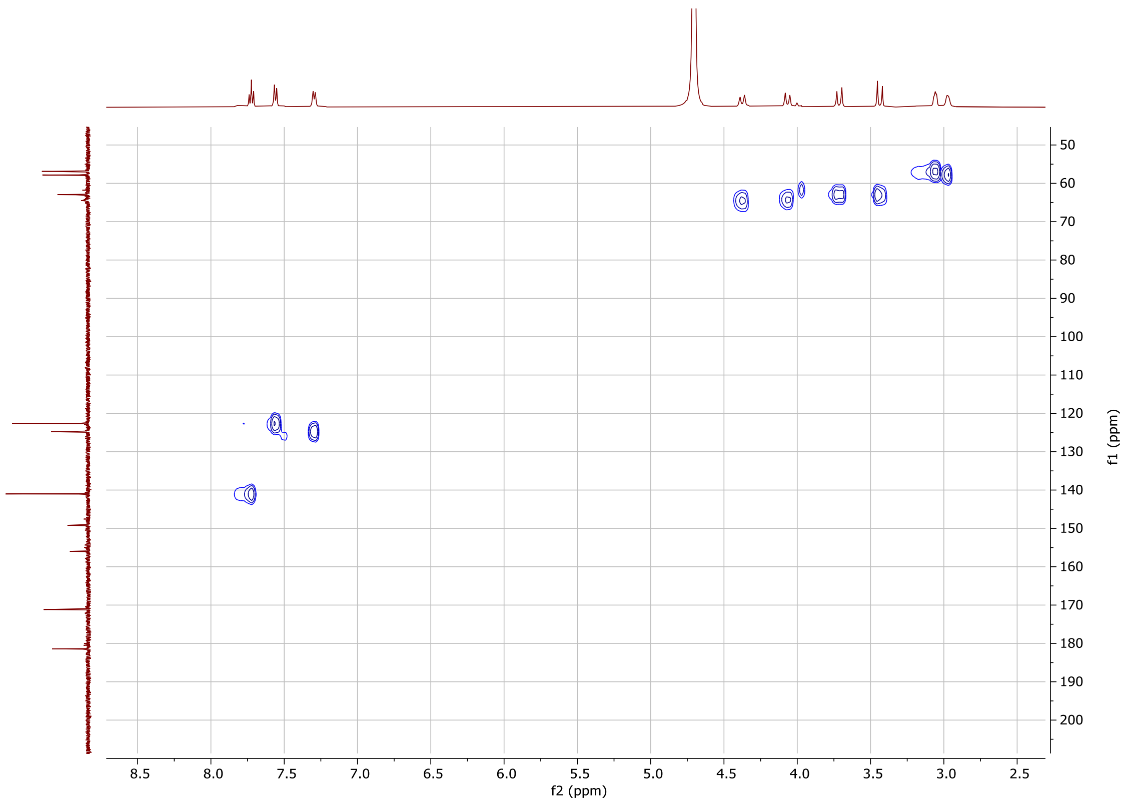

Figure S97. ^{1}H-^{13}C HSQC NMR spectrum (500 MHz, 298 K, D_2O pD=6) of [Lu(**asyoctapa**)]$^{-}$.

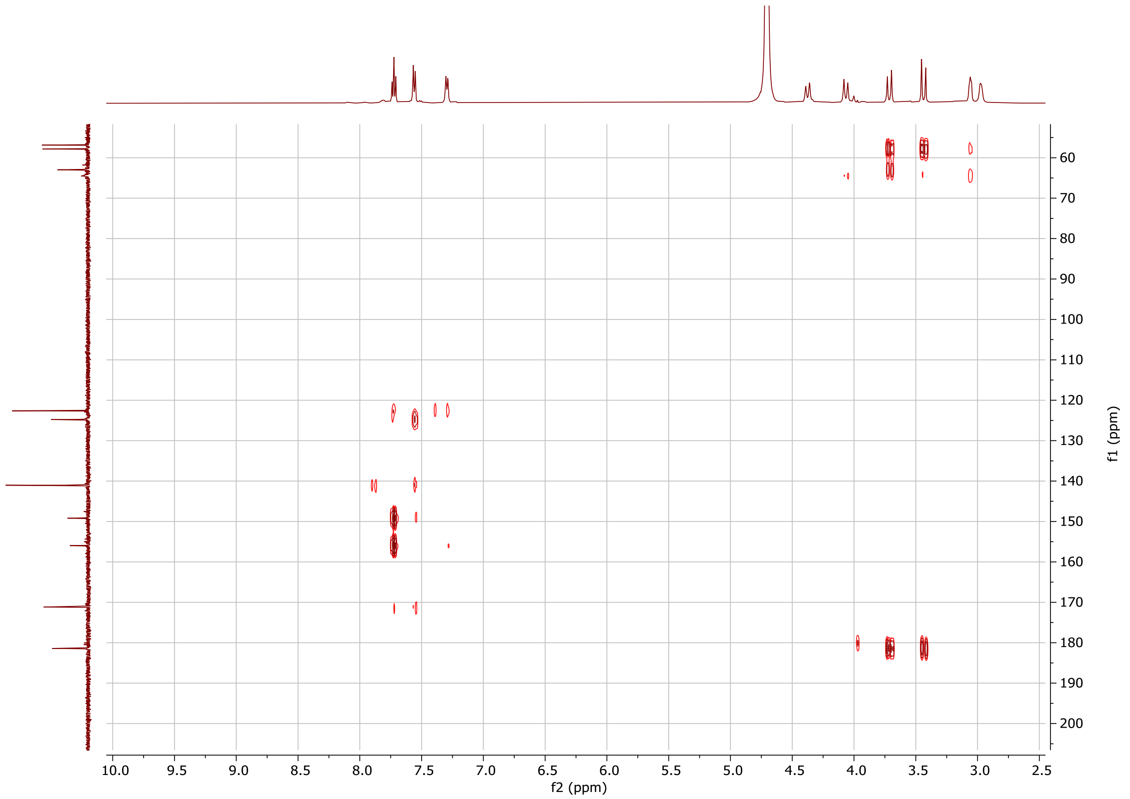

Figure S98. ^{1}H-^{13}C HMBC NMR spectrum (500 MHz, 298 K, D_2O pD=6) of [Lu(**asyoctapa**)]$^{-}$.

Tables with NMR chemical shift assignments

Table S4. Experimental ^{1}H NMR chemical shifts (ppm) (500 MHz, 298 K), of the complexes [La(**tpaen**)]$^{-}$ (D_2O pD = 6) [La(**tpaopd**)]$^{-}$ (D_2O pD = 6) and [La(**tpaond**)]$^{-}$ (D_2O:CD_3CN 7:3 pD = 6).

	**[La(tpaen)]**	**[La(tpaopd)]**	**[La(tpaond)]**
**Ha´**	2.73 (t, *J* = 10.2 Hz, **2H**)	-	-
**Ha**	2.86 (t, *J* = 10.2 Hz, **2H**)	-	-
**Hb´**	3.83 (d, *J* = 17.3 Hz, **2H**)	3.80 (d, *J* = 13.5 Hz, **2H**)	4.29 (d, *J* = 13.6 Hz, **2H**)
**Hb**	4.11 (d, *J* = 17.3 Hz, **2H**)	4.27 (d, *J* = 13.5 Hz, **2H**)	4.85 (d, *J* = 13.6 Hz, **2H**)
**Hd**	7.43 (d, *J* = 7.8 Hz, **2H**)	7.02 (d, *J* = 7.6 Hz, **2H**)	7.28 (d, *J* = 7.0 Hz, **2H**)
**He**	7.96 (t, **2H**)	7.73 (t, *J* = 7.7 Hz, **2H**)	8.06 (t, **2H**)
**Hf**	7.84 (d, *J* = 7.7 Hz, **2H**)	7.62 (d, **2H**)	8.05 (d, **2H**)
**Hi´**	3.32 (d, *J* = 15.6 Hz, **2H**)	2.48 (d, *J* = 16.7 Hz, **2H**)	2.98 (d, *J* = 16.2 Hz, **2H**)
**Hi**	3.93 (d, *J* = 15.6 Hz, **2H**)	4.50 (d, *J* = 16.7 Hz, **2H**)	5.02 (d, *J* = 16.2 Hz, **2H**)
**Hk**	7.49 (d, *J* = 7.7 Hz, **2H**)	7.42 (d, *J* = 7.4 Hz, **2H**)	7.83 (d, *J* = 6.4 Hz, **2H**)
**Hl**	8.03 (t, *J* = 7.7 Hz, **2H**)	8.02 (t, **2H**)	8.42 (t, **2H**)
**Hm**	7.96 (d, **2H**)	8.04 (d, **2H**)	8.41 (d, **2H**)
**Hp**	-	7.46 (dd, *J* = 6.3, 3.5 Hz, **2H**)	8.73 (s, **2H**)
**Hq**	-	7.76 (dd, **2H**)	-
**Hr**	-	-	7.94 (dd, *J* = 6.4, 3.2 Hz, **2H**)
**Hs**	-	-	8.29 (dd, *J* = 6.3, 3.4 Hz, **2H**)

Table S5. Experimental ^{13}C NMR chemical shifts (ppm) (126 MHz, 298 K), of the complexes [La(**tpaen**)]$^-$ (D_2O pD = 6) [La(**tpaopd**)]$^-$ (D_2O pD = 6) and [La(**tpaond**)]$^-$ (D_2O:CD_3CN 7:3 pD = 6).

	**[La(tpaen)]$^-$**	**[La(tpaopd)]$^-$**	**[La(tpaond)]$^-$**
**Ca**	59.33	146.16	145.01
**Cb**	63.38	62.51	63.07
**Cc**	160.39	156.84	156.21
**Cd**	124.50	126.43	125.35
**Ce**	141.05	141.50	140.69
**Cf**	123.51 or 123.48*	123.67 or 123.88*	123.21
**Cg**	151.92	152.07	152.21
**Ch**	171.55	170.37	169.14
**Ci**	62.42	60.85	61.04
**Cj**	155.19	154.34	153.48
**Ck**	125.81	126.19	125.35
**Cl**	140.27	140.74	140.07
**Cm**	123.51 or 123.48*	123.67 or 123.88*	123.28
**Cn**	151.30	151.85	151.90
**Co**	173.16	173.35	172.09
**Cp**	-	129.73	125.10
**Cq**	-	125.82	132.22
**Cr**	-	-	126.70 or 126.99*
**Cs**	-	-	126.70 or 126.99*

*They cannot be assigned unequivocally.

Table S6. Experimental ^{1}H NMR chemical shifts (ppm) (500 MHz, 298 K), of the complexes [Lu(**tpaopd**)]$^{-}$ (D_2O pD = 6) and [Lu(**tpaond**)]$^{-}$ (D_2O:CD_3CN 7:3 pD = 6).

	**[Lu(tpaopd)]**	**[Lu(tpaond)]**
**Hb´**	3.24 (d, *J* = 13.7 Hz, **1H**)	3.41 (d, *J* = 13.7 Hz, **1H**)
**Hb**	4.28 (d, J = 13.7 Hz, **1H**)	4.46 (d, *J* = 13.7 Hz, **1H**)
**Hd**	6.88 (d, *J* = 7.8 Hz, **1H**)	6.78 (d, *J* = 7.9 Hz, **1H**)
**He**	7.24 (t, *J* = 7.8 Hz, **1H**)	*
**Hf**	*	*
**Hi´**	5.05 (d, *J* = 15.0 Hz, **1H**)	5.41 (s, **2H**)
**Hi**	5.13 (d, *J* = 15.0 Hz, **1H**)	
**Hk**	7.57 (d, *J* = 7.9 Hz, **1H**)	*
**Hl**	*	8.36 (t, *J* = 7.7 Hz, **1H**)
**Hm**	*	*
**Hp´**	4.46 (d, J = 14.4 Hz, **1H**)	4.78 (d, *J* = 14.2 Hz, **1H**)
**Hp**	5.32 (d, *J* = 14.4 Hz, **1H**)	5.62 (d, *J* = 14.2 Hz, **1H**)
**Hr**	*	7.40 (d, *J* = 7.6 Hz, **1H**)
**Hs**	8.16 (t, *J* = 7.7 Hz, **1H**)	*
**Ht**	*	*
**Hw´**	4.78(d, *J* = 19.4 Hz, **1H**)	5.00 (d, *J* = 19.7 Hz, **1H**)
**Hw**	5.00 (d, *J* = 19.4 Hz, **1H**)	5.14 (d, *J* = 19.7 Hz, **1H**)
**Hy**	7.21 (d, *J* = 7.6 Hz, **1H**)	7.53 (d, *J* = 8.2 Hz, **1H**)
**Hz**	6.83 (t, *J* = 7.8 Hz, **1H**)	7.61 (t, *J* = 7.6 Hz, **1H**)
**Ha2**	6.28 (d, *J* = 8.2 Hz, **1H**)	*
**He2**	*	6.87 (s, **1H**)
**Hf2**	*	8.46 (s, **1H**)
**Hg2**	*	-
**Hh2**	*	-
**Hi2**	-	*
**Hj2**	-	*
**Hk2**	-	*
**Hl2**	-	*

*They cannot be assigned unequivocally.

Table S7. Experimental ^{13}C NMR chemical shifts (ppm) (126 MHz, 298 K), of the complexes [Lu(**tpaopd**)]$^-$ (D_2O pD = 6) and [Lu(**tpaond**)]$^-$ (D_2O:CD_3CN 7:3 pD = 6).

	**[La(tpaopd)]$^-$**	**[La(tpaond)]$^-$**
**Ca**	152.45	146.84
**Cb**	61.53	60.64
**Cc**	153.82	151.69
**Cd**	128.71	127.98
**Ce**	128.90	*
**Cf**	137.48	*
**Cg**	148.67	*
**Ch**	*	*
**Ci**	56.88	56.95
**Cj**	157.96	155.05
**Ck**	127.35	*
**Cl**	*	140.66
**Cm**	*	*
**Cn**	150.12	147.87
**Co**	*	*
**Cp**	62.85	62.67
**Cq**	155.49	153.27
**Cr**	127.29	*
**Cs**	141.39	*
**Ct**	129.73	*
**Cu**	149.43	*
**Cv**	*	*
**Cw**	68.34	68.11
**Cx**	153.62	156.82
**Cy**	*	*
**Cz**	126.79	*
**Ca2**	127.94	*
**Cb2**	148.38	*
**Cc2**	*	*

*They cannot be assigned unequivocally.

Table S7 (continuation). Experimental ^{13}C NMR chemical shifts (ppm) (126 MHz, 298 K), of the complexes [Lu(**tpaopd**)]$^-$ (D_2O pD = 6) and [Lu(**tpaond**)]$^-$ ($D_2O:CD_3CN$ 7:3 pD = 6).

	**[Lu(tpaopd)]$^-$**	**[Lu(tpaond)]$^-$**
**Cd2**	140.39	138.06
**Ce2**	141.68 or 141.81*	130.99
**Cf2**	141.68 or 141.81*	121.07
**Cg2**	*	129.52
**Ch2**	*	130.99
**Ci2**	-	*
**Cj2**	-	*
**Ck2**	-	*
**Cl2**	-	*

*They cannot be assigned unequivocally.

Table S8. Experimental ^{1}H NMR chemical shifts (ppm) (500 MHz, 298 K, D_2O pD=6), of the complexes [La(**asyoctapa**)]$^-$ and [Lu(**asyoctapa**)]$^-$.

	**[La(asyoctapa)]$^-$**	**[Lu(asyoctapa)]$^-$**
**Ha**	3.13 (t, *J* = 4.9 Hz, **2H**).	3.15 (t, *J* = 3.2 Hz, **2H**).
**Hb´**	4.06 (d, *J* = 15.1 Hz, **2H**)	4.15 (d, *J* = 15.6 Hz, **2H**)
**Hb**	4.48 (d, *J* = 15.0 Hz, **2H**)	4.46 (d, *J* = 15.1 Hz, **2H**)
**Hd**	7.44 (d, *J* = 7.8 Hz, **2H**)	7.38 (d, *J* = 7.8 Hz, **2H**)
**He**	7.85 (t, *J* = 7.8 Hz, **2H**)	7.81 (t, *J* = 7.8 Hz, **2H**)
**Hf**	7.73 (d, *J* = 7.8 Hz, **2H**)	7.65 (d, *J* = 7.8 Hz, **2H**)
**Hi**	3.02 (t, *J* = 4.9 Hz, **2H**)	3.06 (t, *J* = 3.2 Hz, **2H**)
**Hj´**	3.47 (d, *J* = 16.8 Hz, **2H**)	3.52 (d, *J* = 16.9 Hz, **2H**)
**Hj**	3.71 (d, *J* = 16.9 Hz, **2H**)	3.80 (d, *J* = 16.9 Hz, **2H**)

Table S9. Experimental ^{13}C NMR chemical shifts (ppm) (126 Mz, 298 K, D_2O pD=6), of the complexes [La(**asyoctapa**)]$^-$ and [Lu(**asyoctapa**)]$^-$.

	**[La(asyoctapa)]$^-$**	**[Lu(asyoctapa)]$^-$**
**Ca**	56.26	56.88
**Cb**	63.91	64.52
**Cc**	157.02	155.98
**Cd**	125.47	124.77
**Ce**	141.20	141.03
**Cf**	123.36	122.61
**Cg**	150.55	149.20
**Ch**	172.13	171.17
**Ci**	56.75	57.81
**Cj**	62.72	62.97
**Ck**	180.70	181.41

Mass Spectrometry of compounds (2), (3), (5) and chelators H_4L^1 to H_4L^8

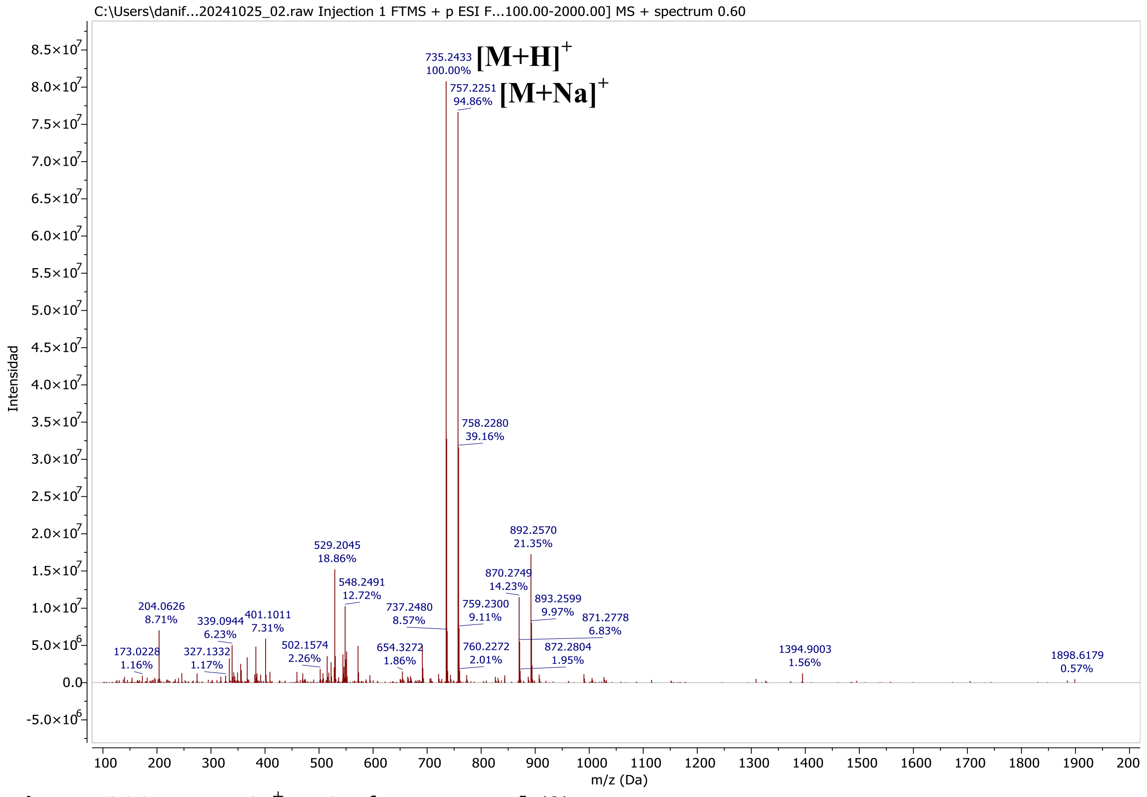

Figure S99. HR-ESI$^+$-MS of **compound** (**2**).

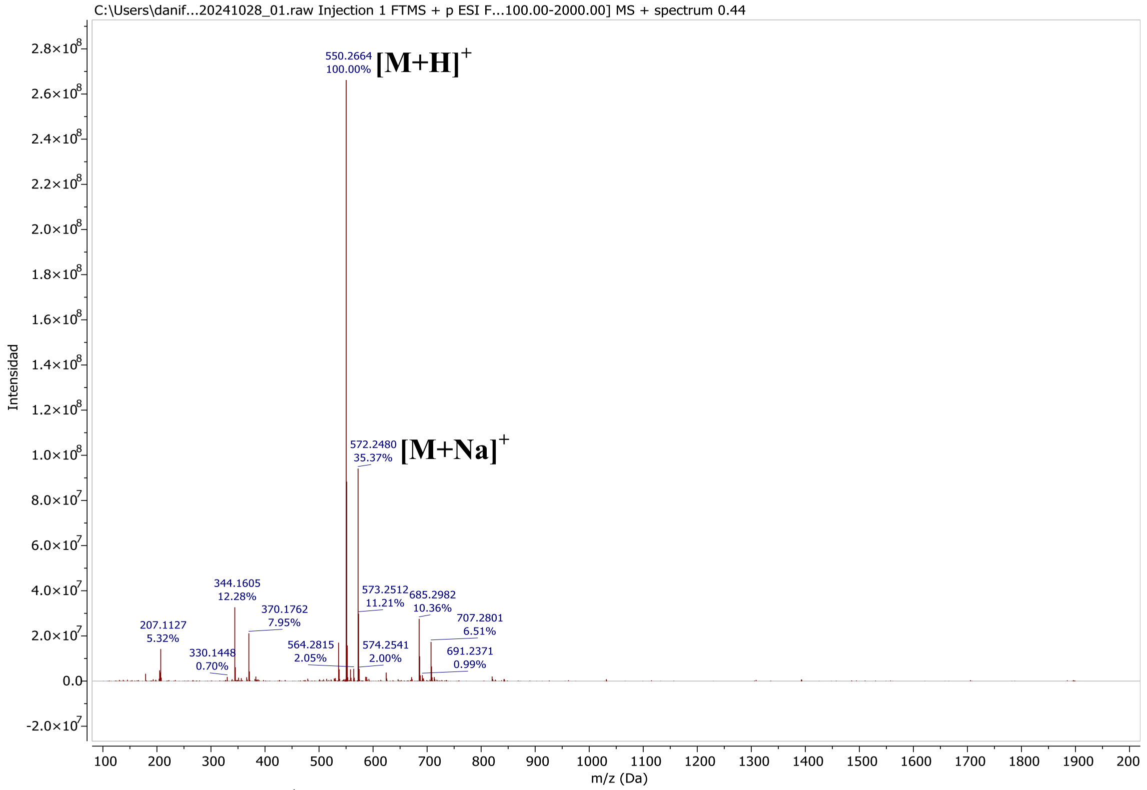

Figure S100. HR-ESI$^+$-MS of **compound** (**3**).

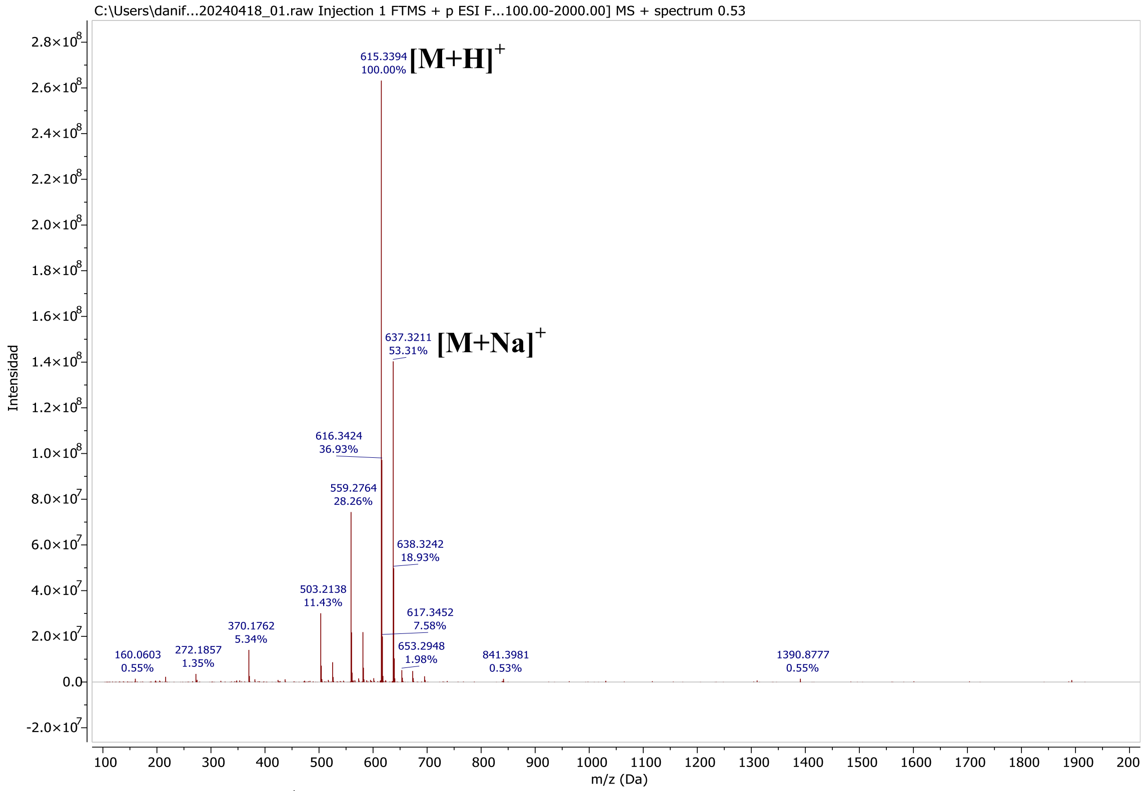

Figure S101. HR-ESI$^+$-MS of **compound** (**5**).

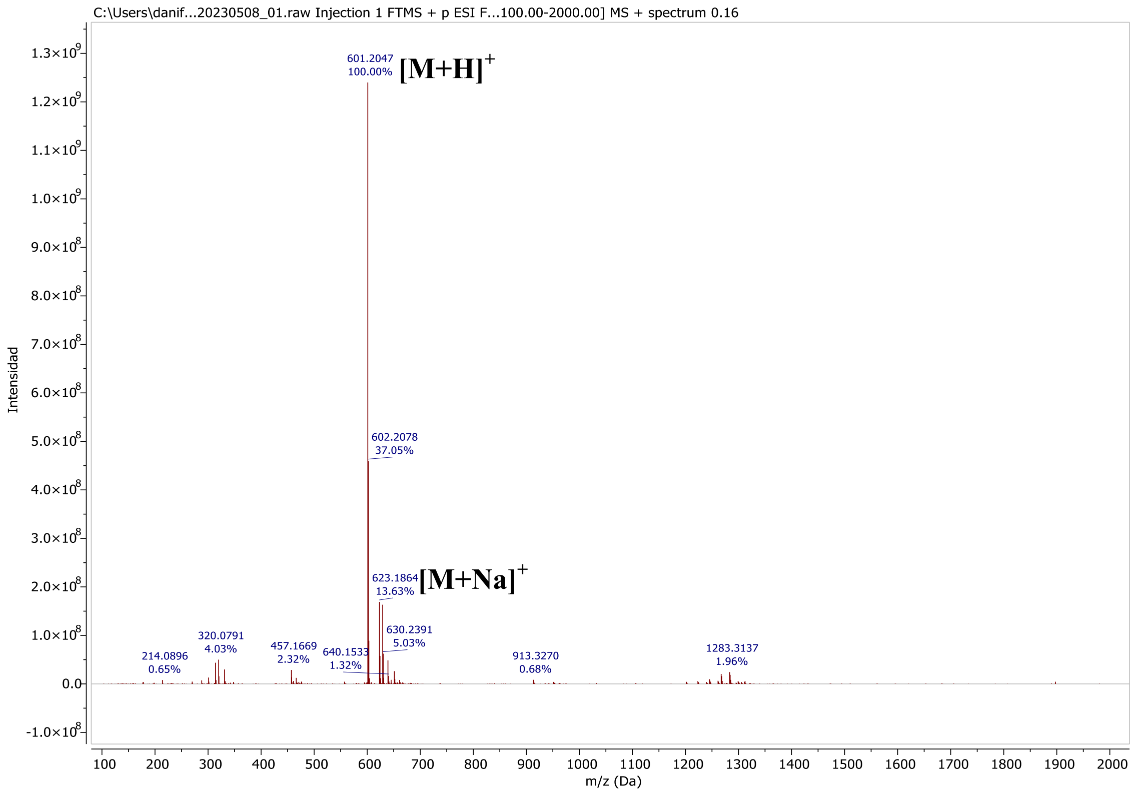

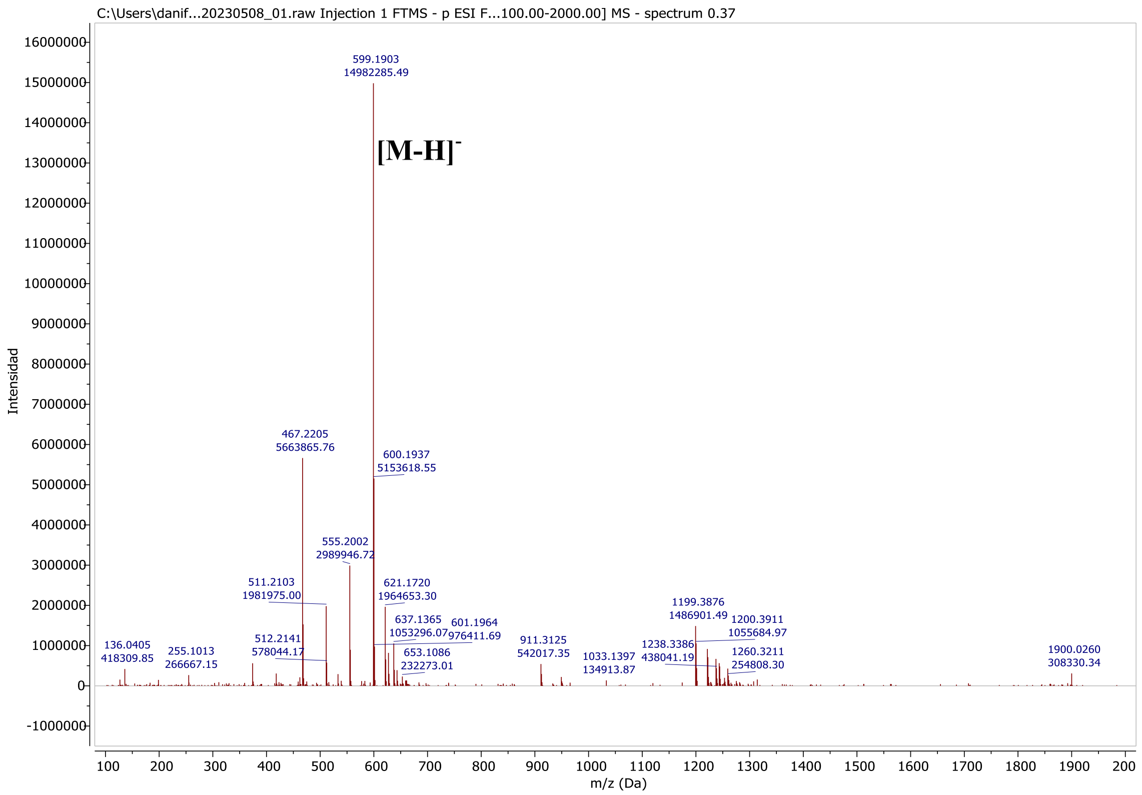

Figure S102. HR-MS spectra of H_4**tpaen** (H_4**L**$^{\mathbf{1}}$): ESI^+ (top), ESI^- (bottom).

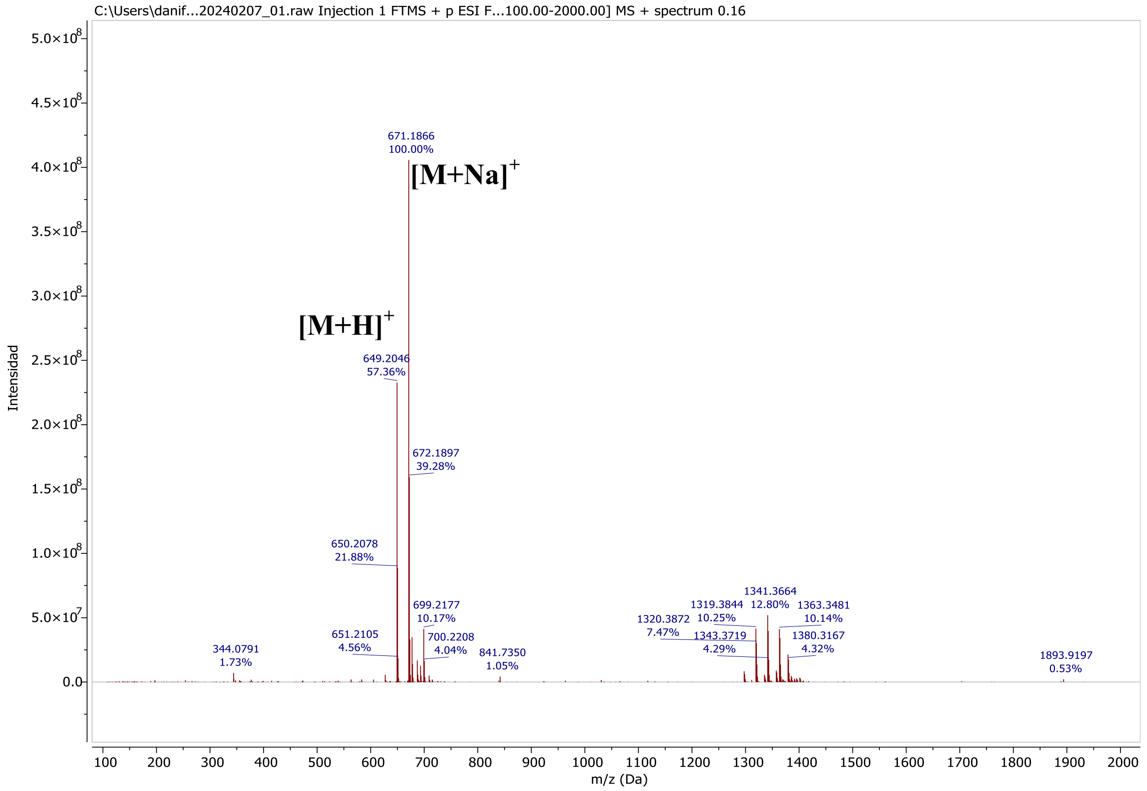

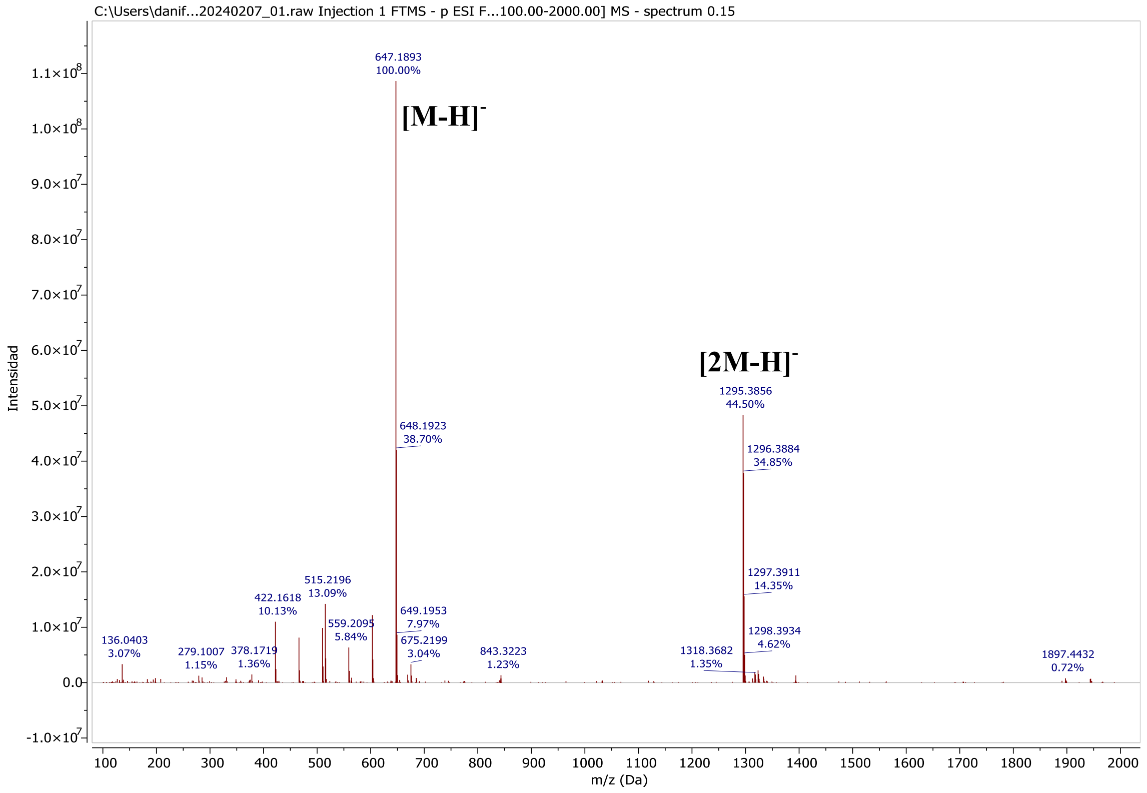

Figure S103. HR-MS spectra of H_4**tpaopd** (H_4**L^2**): ESI^+ (top), ESI^- (bottom).

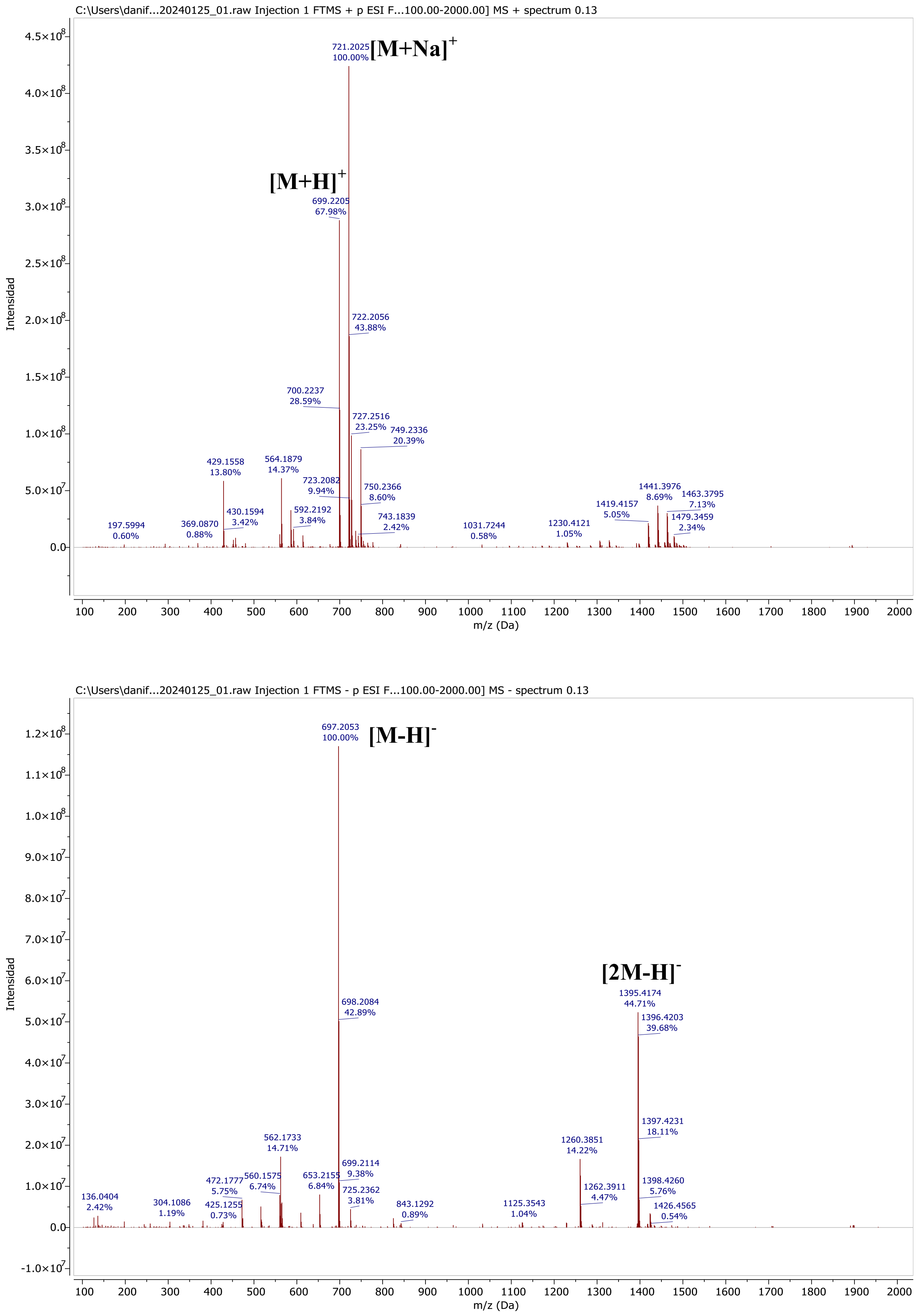

Figure S104. HR-MS spectra of H_4**tpaond** (H_4**L**^{3}): ESI$^+$ (top), ESI$^-$ (bottom).

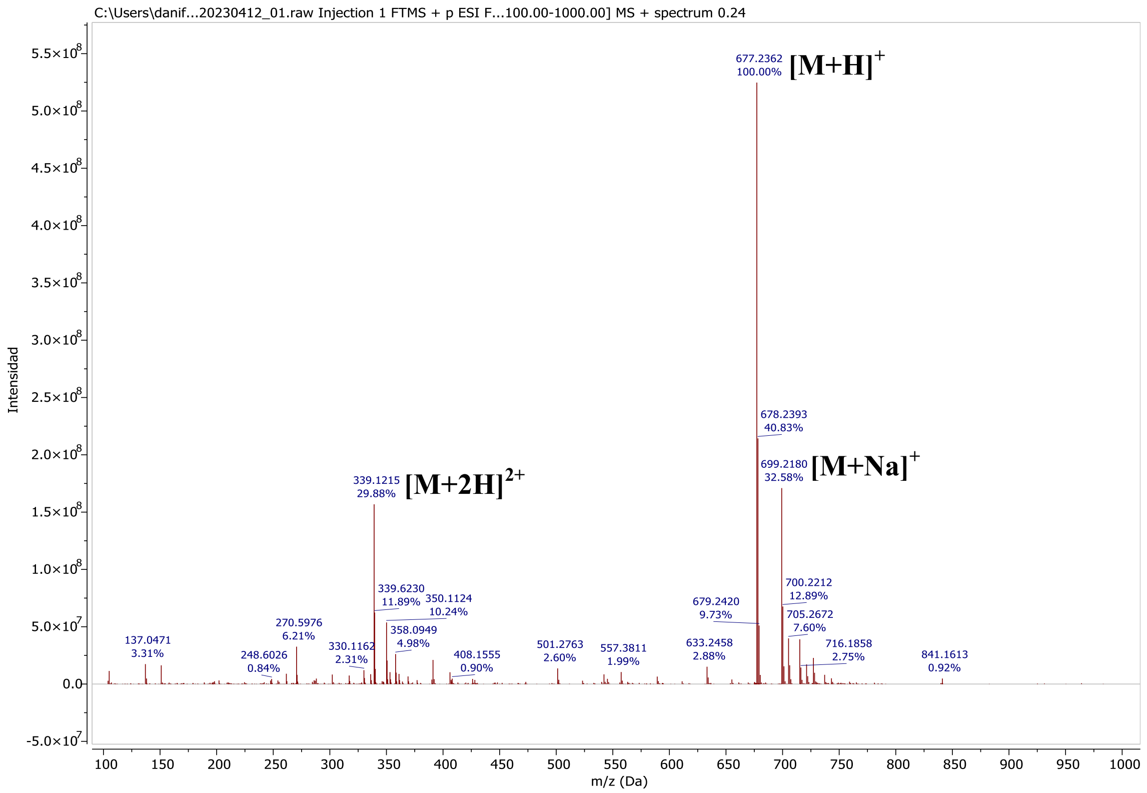

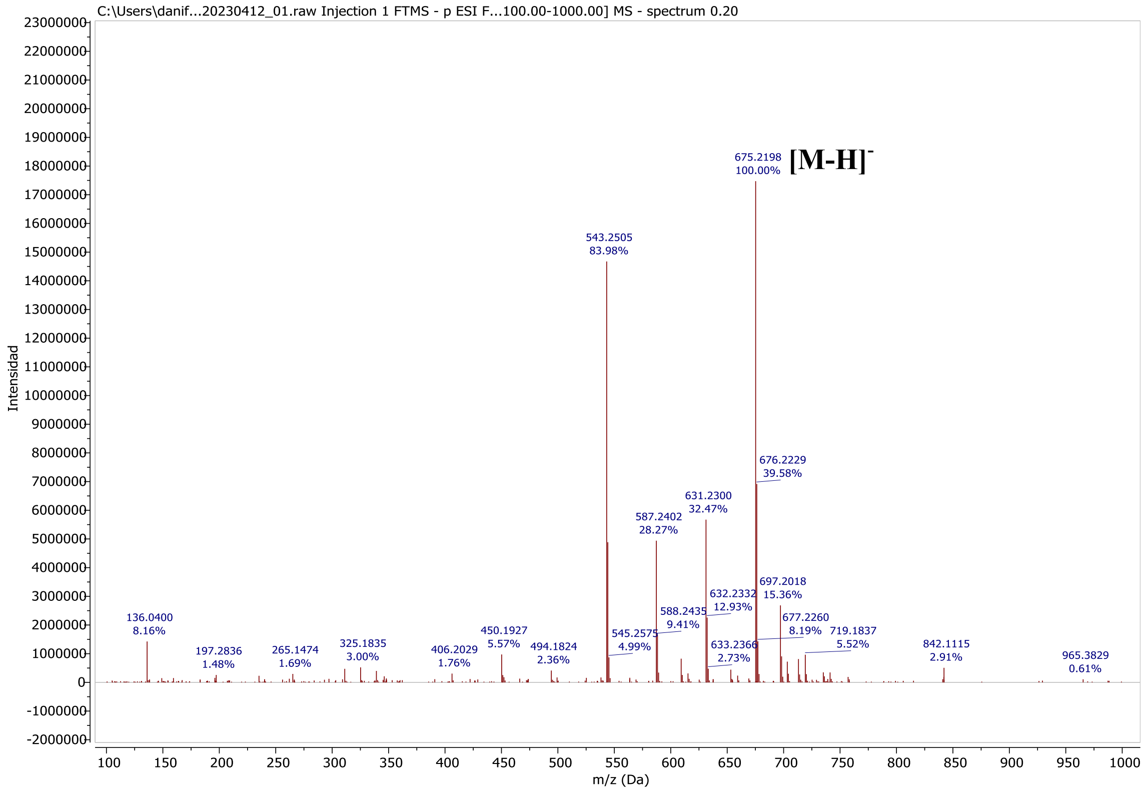

Figure S105. HR-MS spectra of H_4**tpmxd** (H_4**L**^{4}): ESI$^+$ (top), ESI$^-$ (bottom).

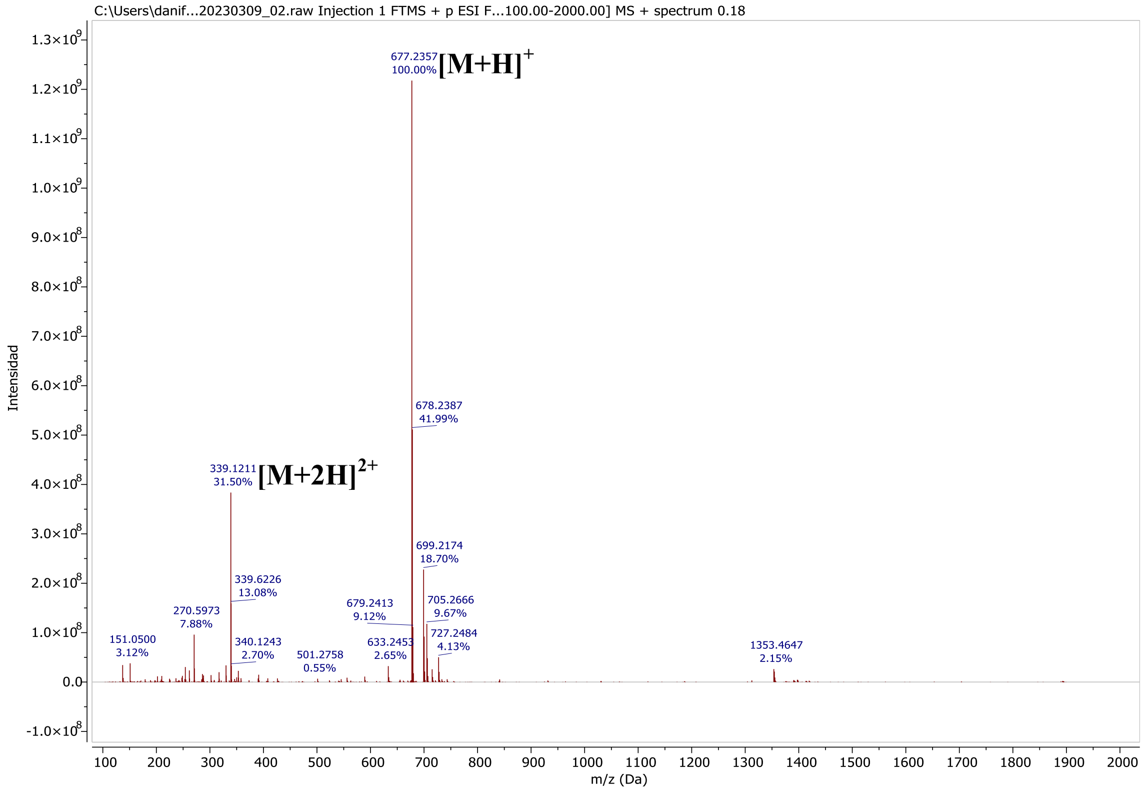

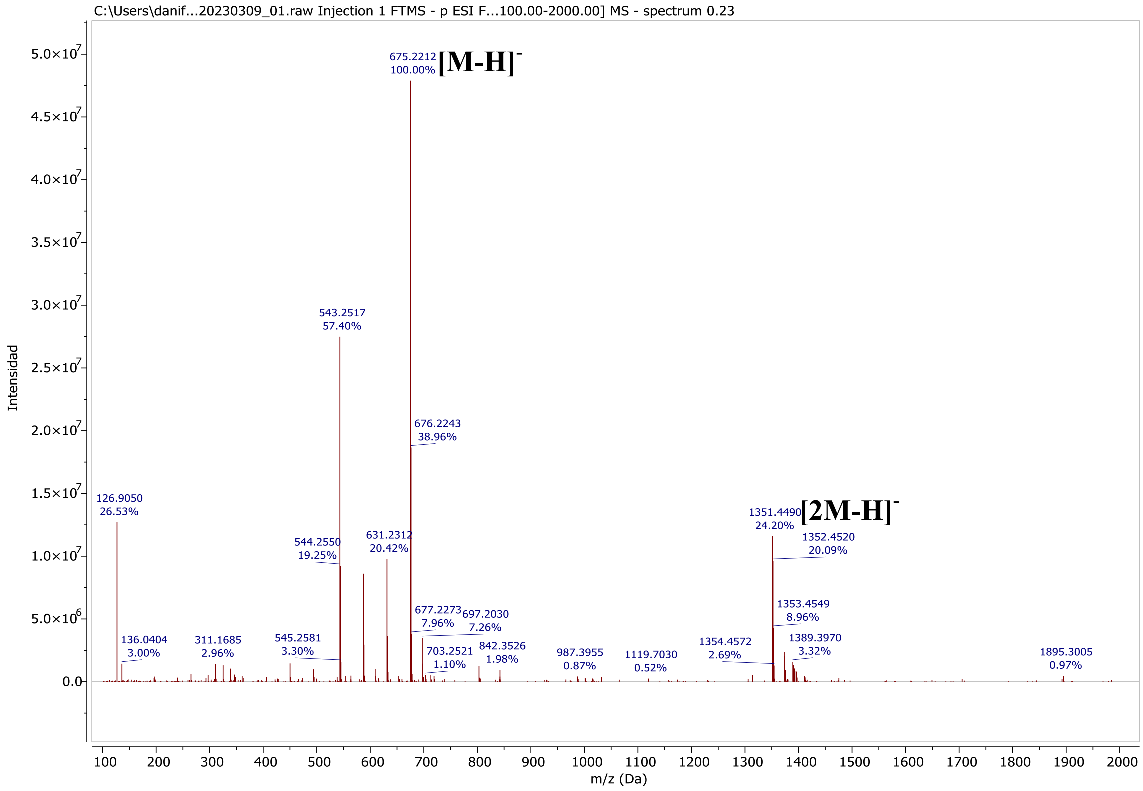

Figure S106. HR-MS spectra of H_4**tppxd** (H_4**L**^{5}): ESI^+ (top), ESI^- (bottom).

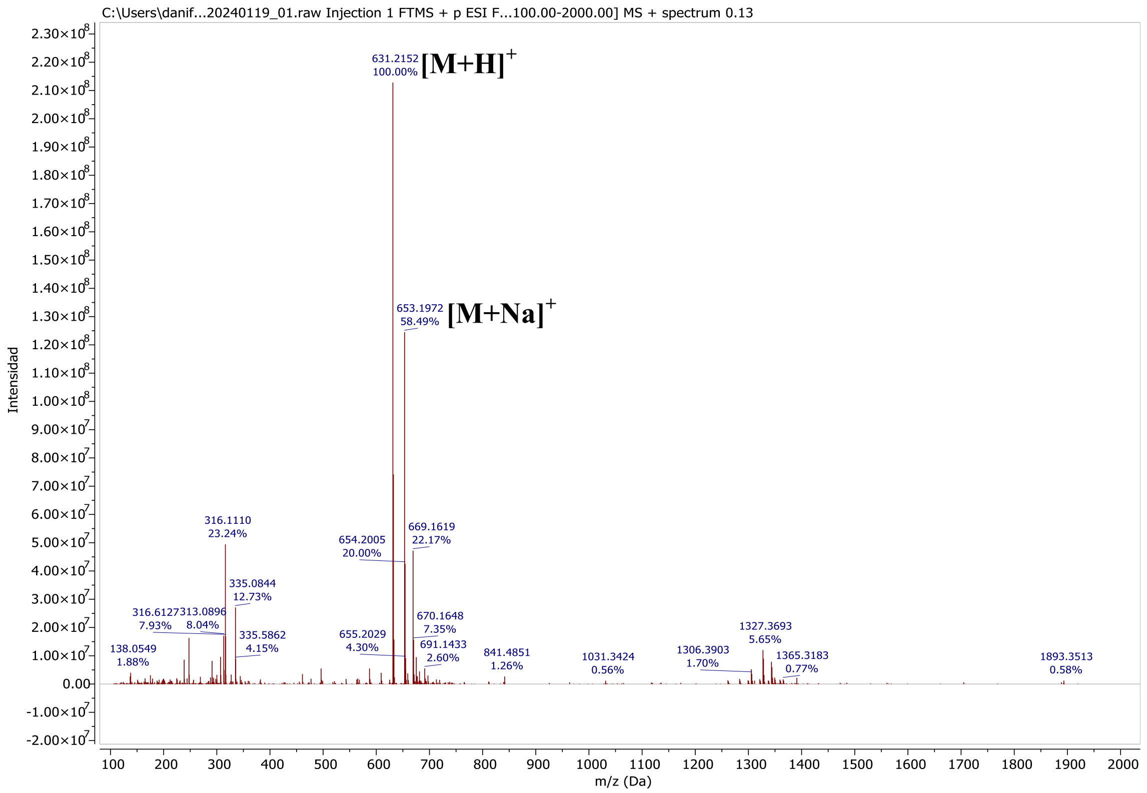

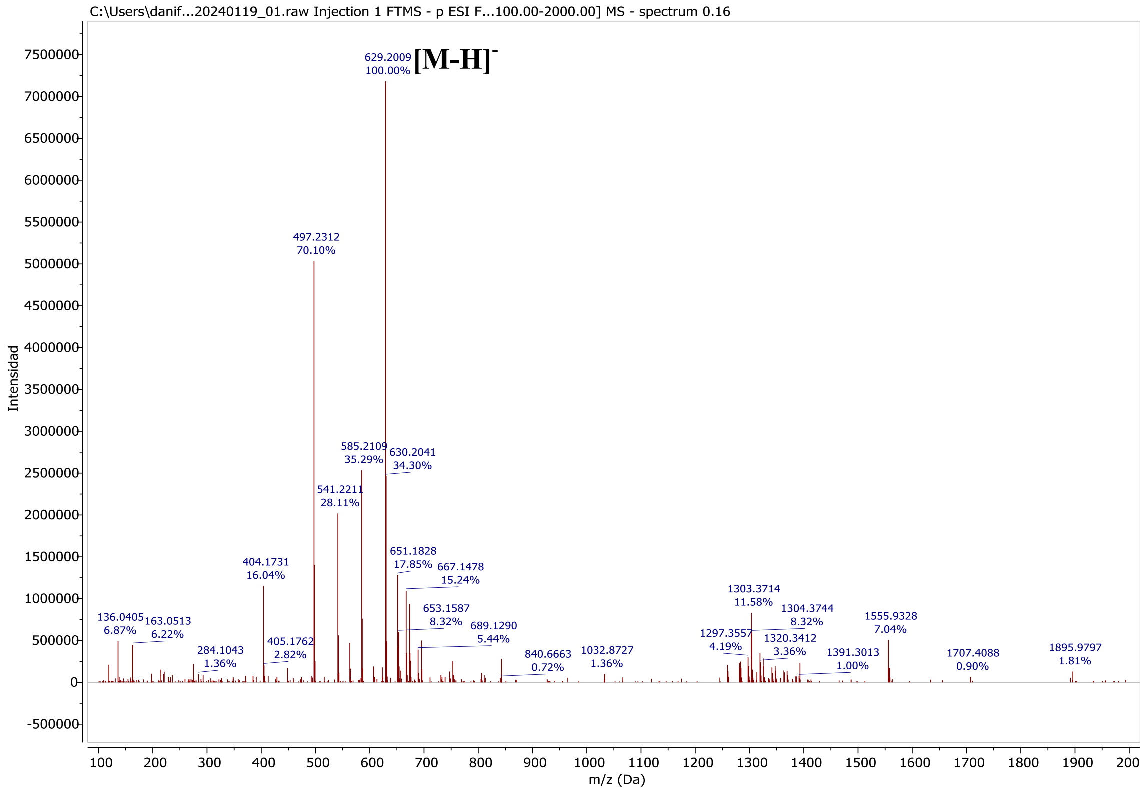

Figure S107. HR-MS spectra of H_4**tpadapo** (H_4**L**^{6}): ESI^+ (top), ESI^- (bottom).

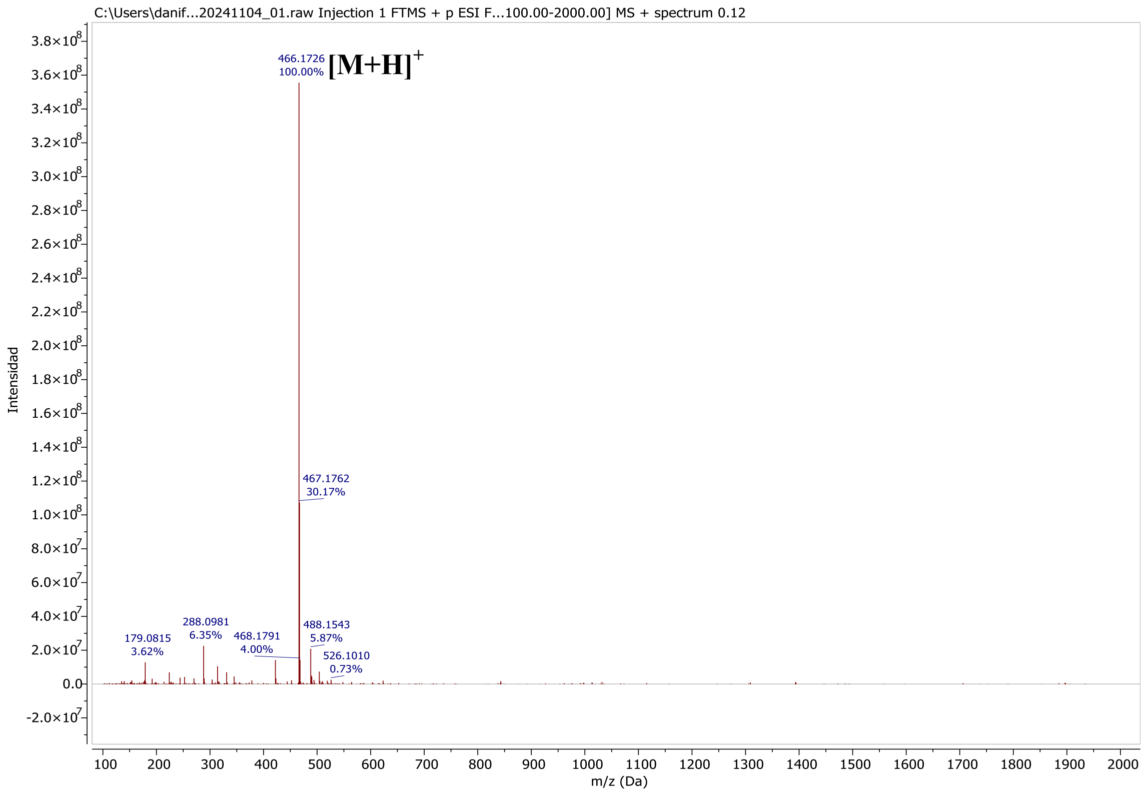

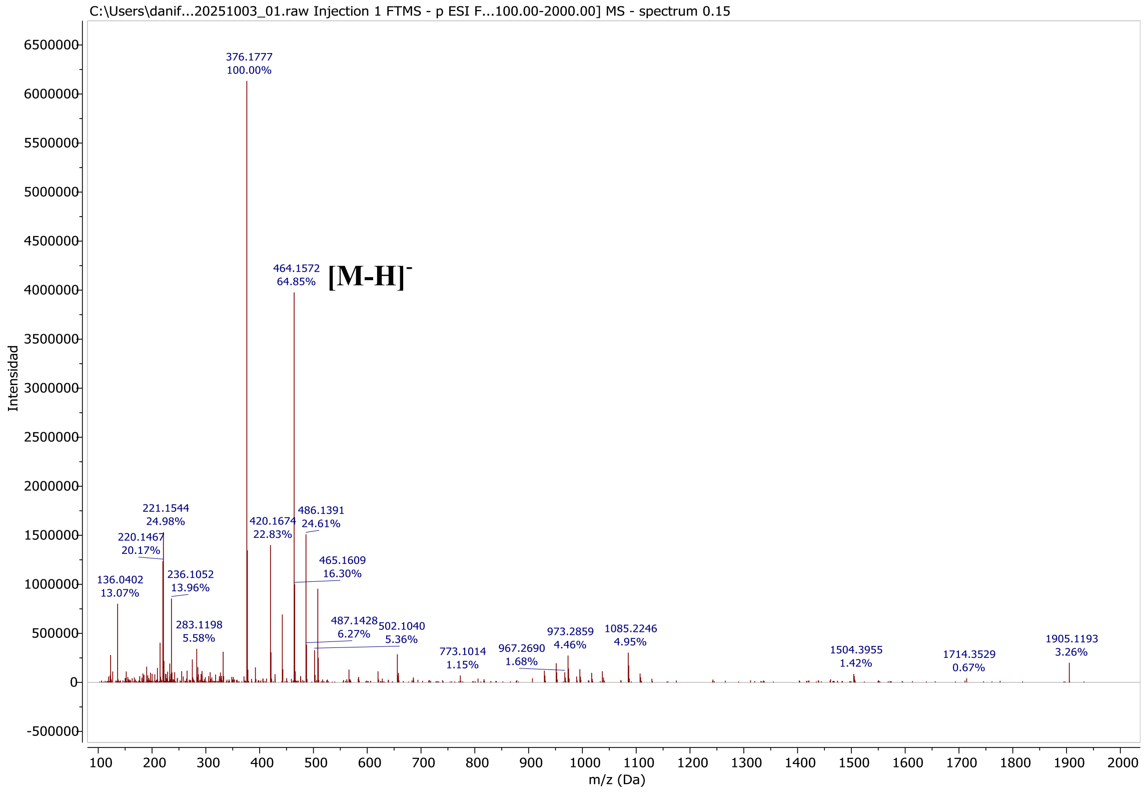

Figure S108. HR-MS spectra of H_3**tripaen** (H_3**L**^{7}): ESI$^+$ (top), ESI$^-$ (bottom).

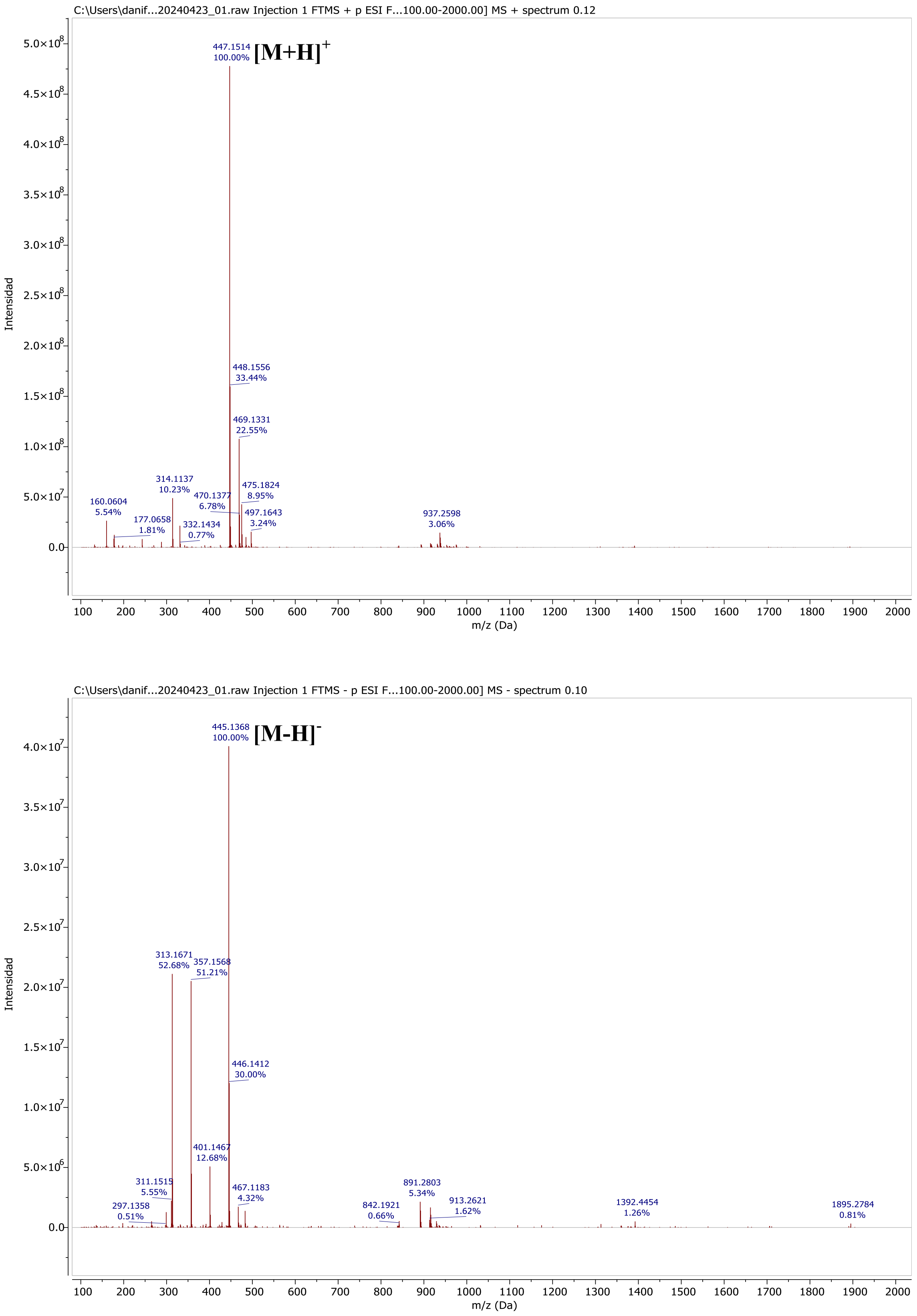

Figure S109. HR-MS spectra of H_4**asyoctapa** (H_4**L**^{1}): ESI^+ (top), ESI^- (bottom).

Mass Spectrometry of cold lanthanum(III) complexes

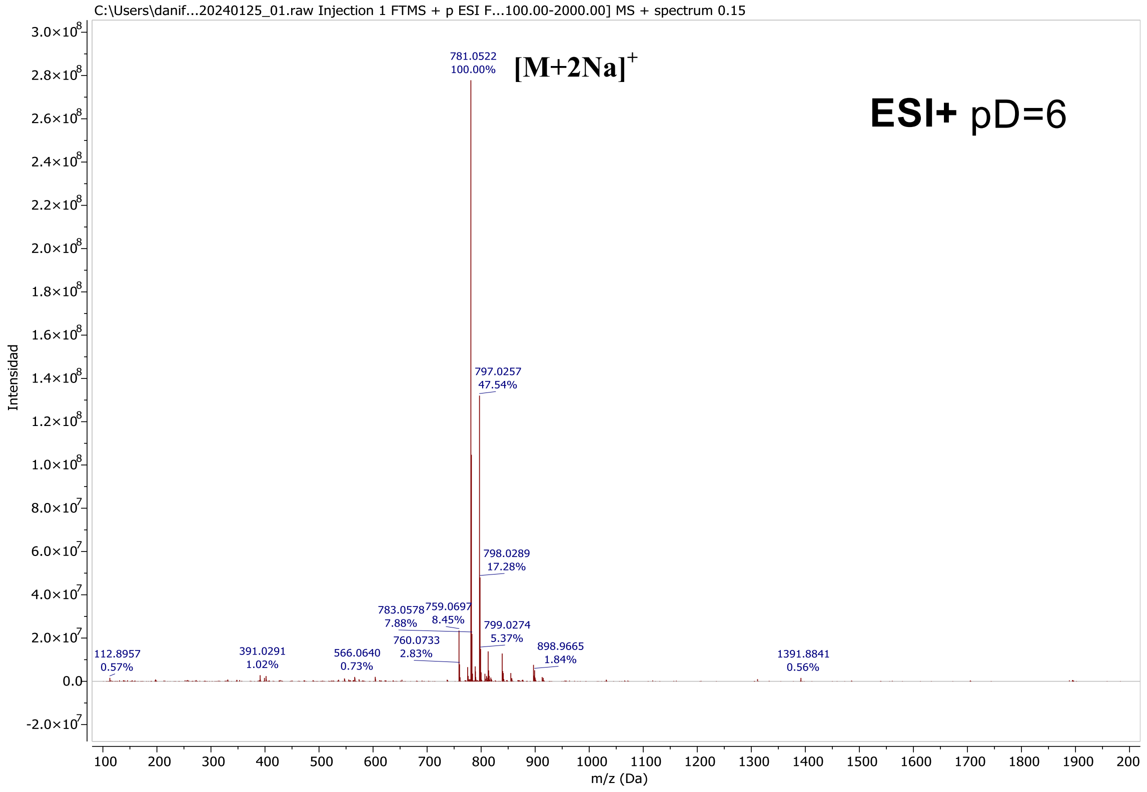

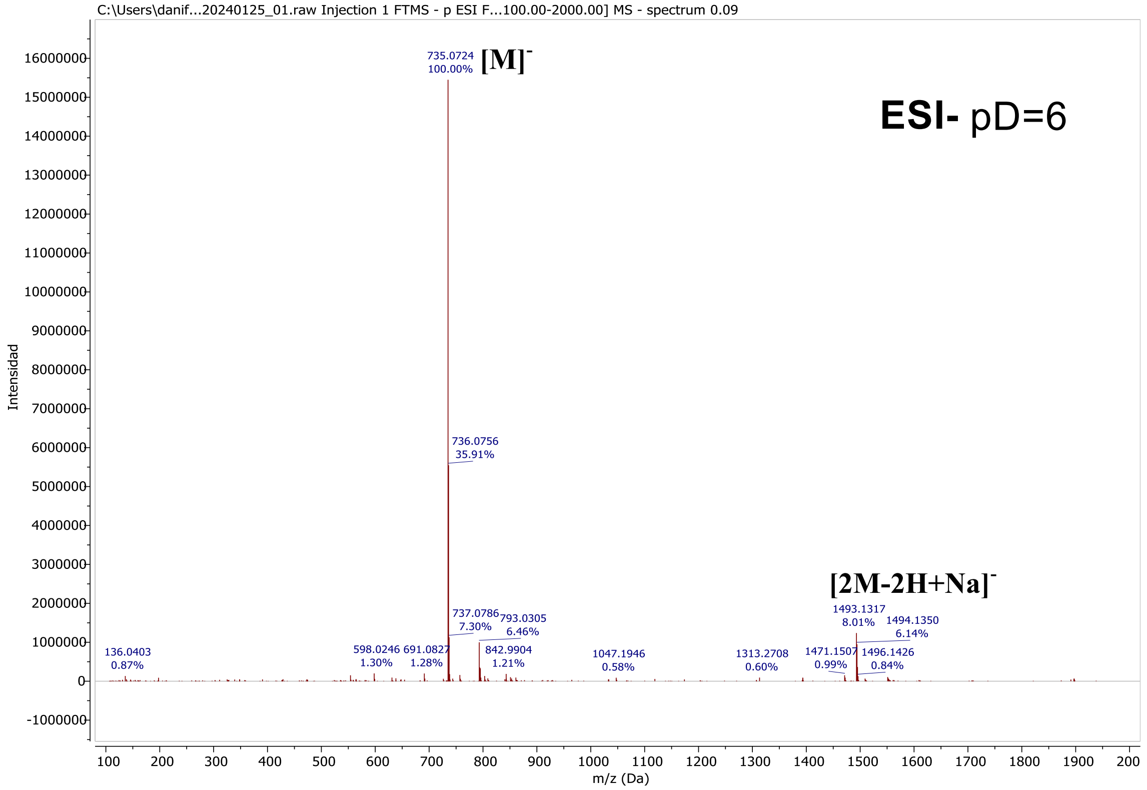

Figure S110. HR-MS spectra of [La(**tpaen**)]$^-$.

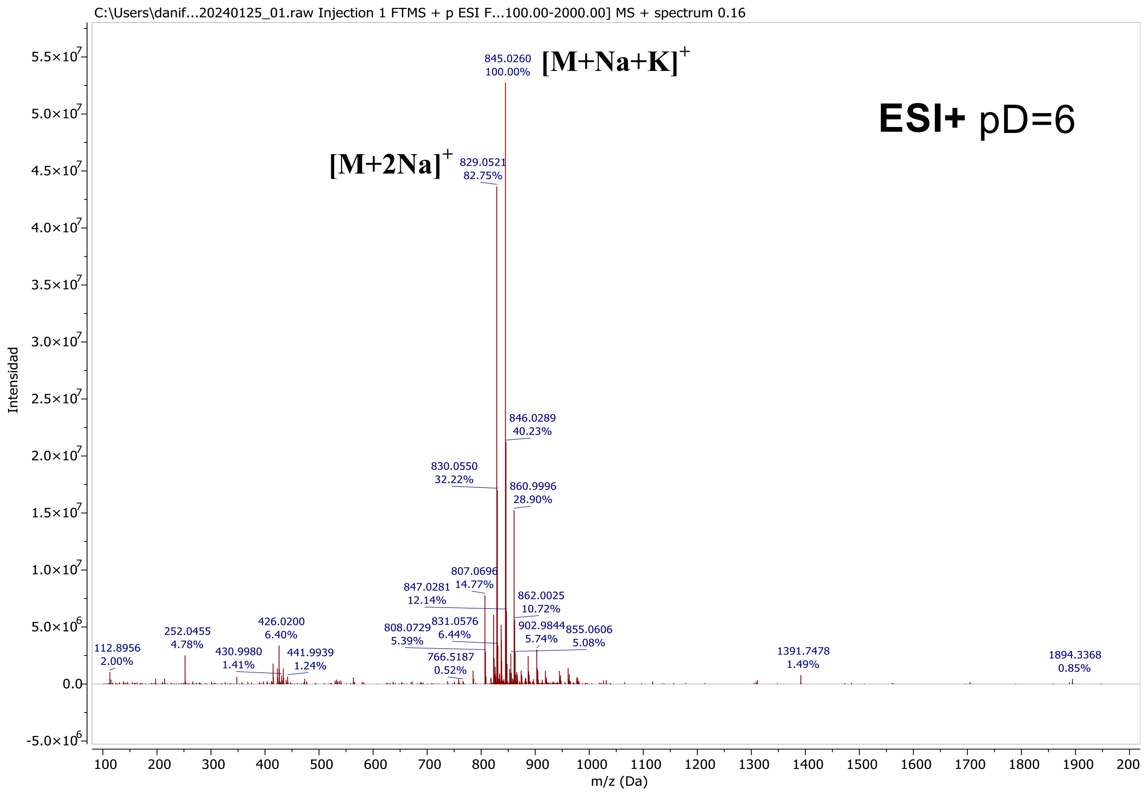

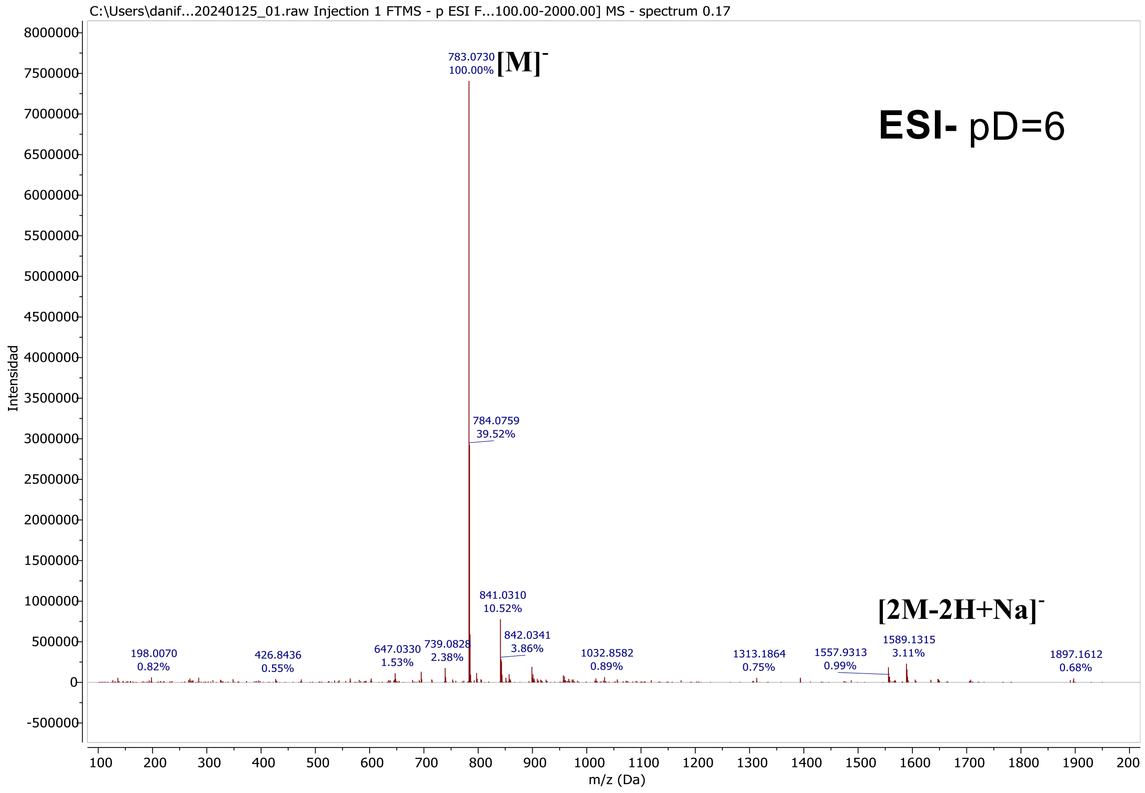

Figure S111. HR-MS spectra of [La(**tpaopd**)]$^-$.

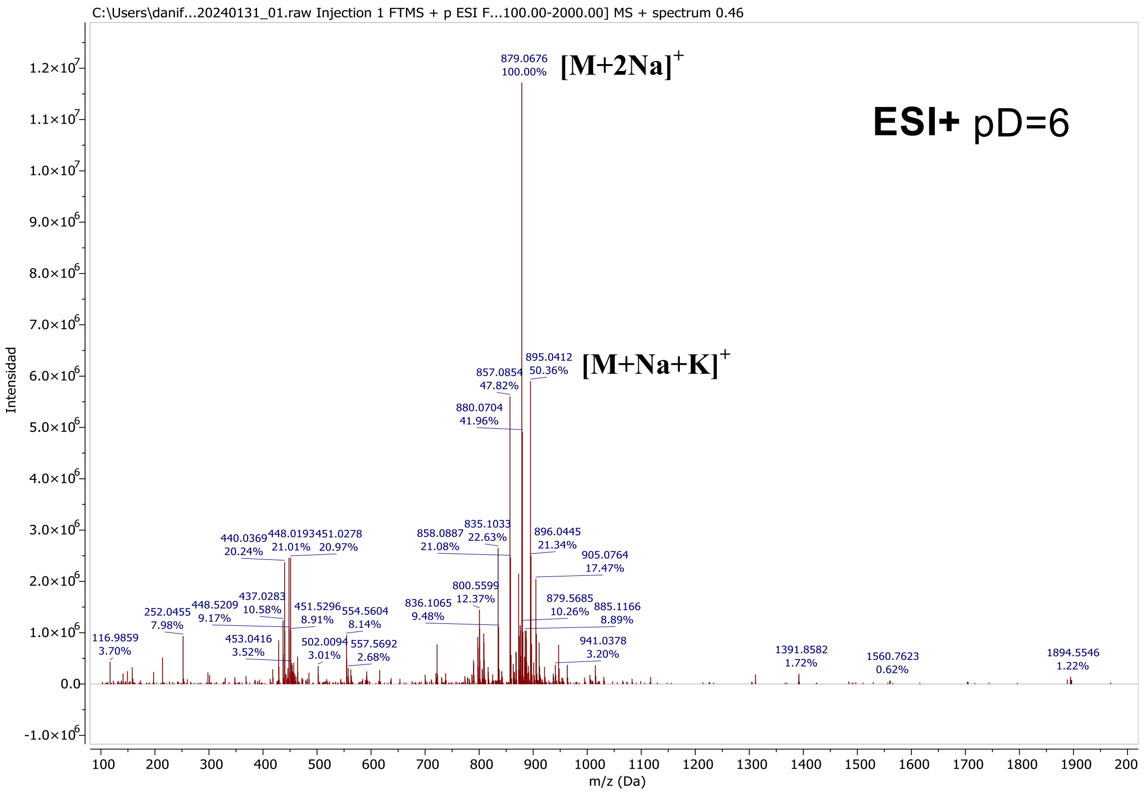

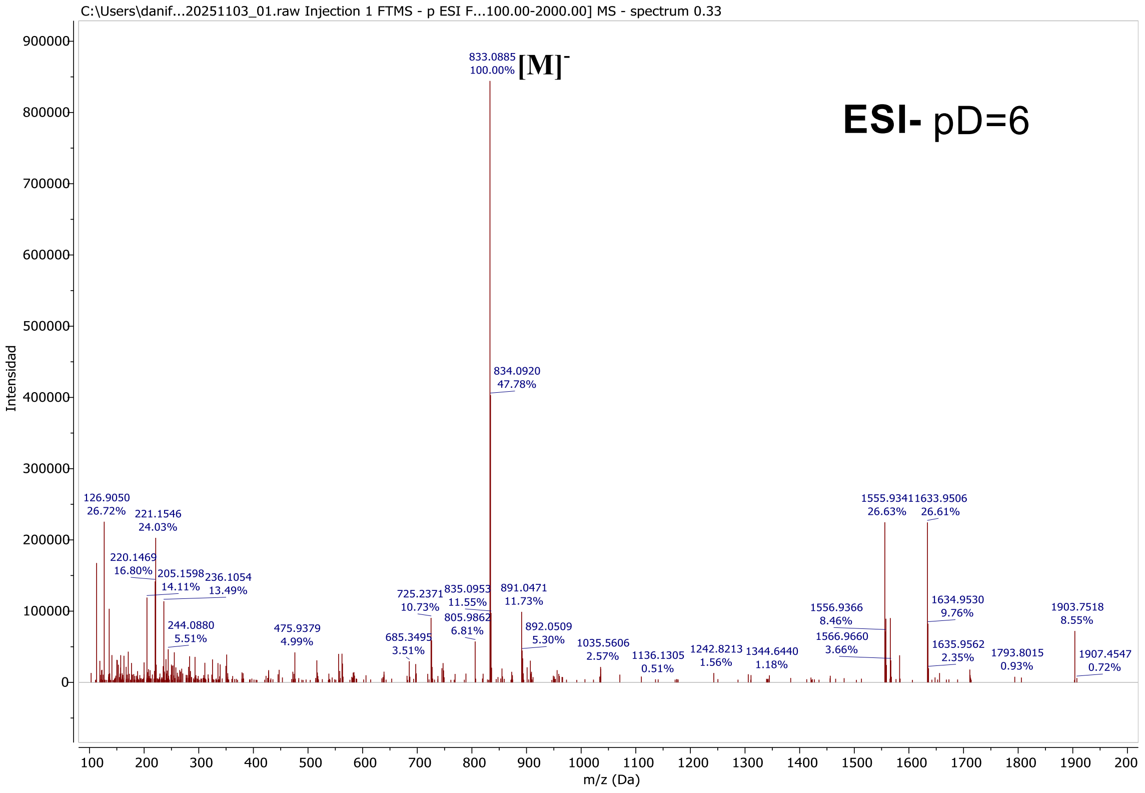

Figure S112. HR-MS spectra of [La(**tpaond**)]$^{-}$.

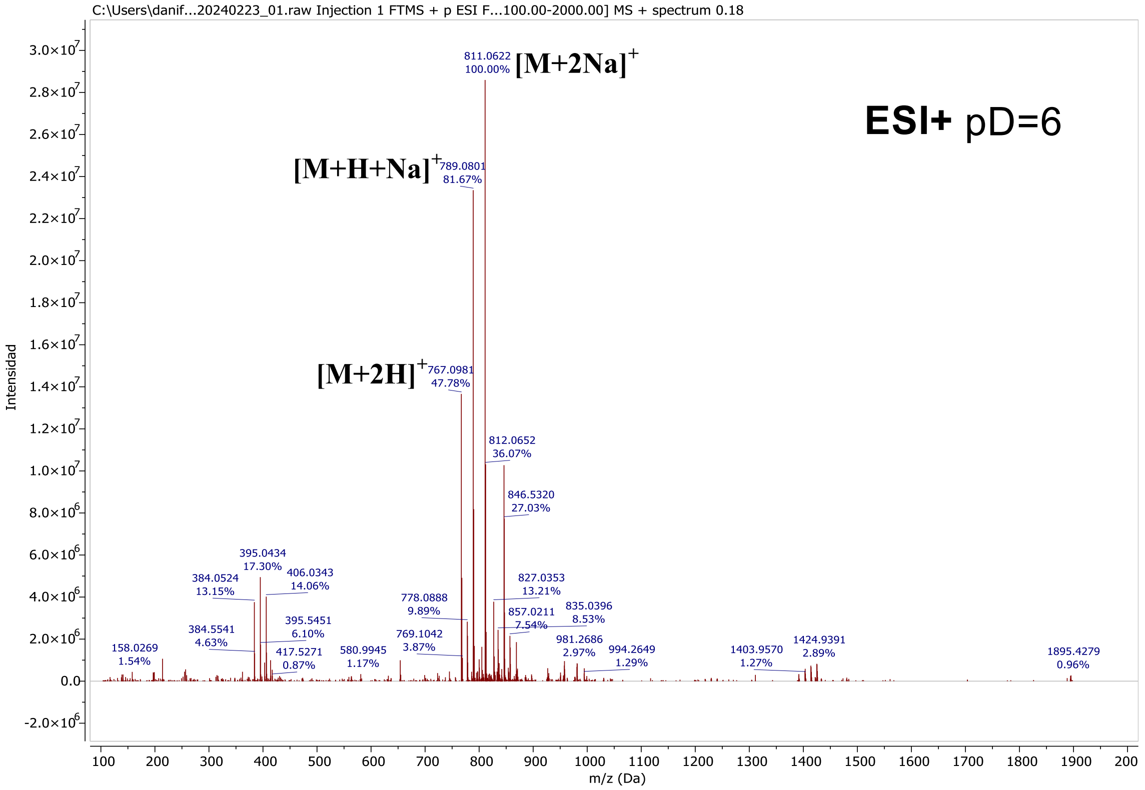

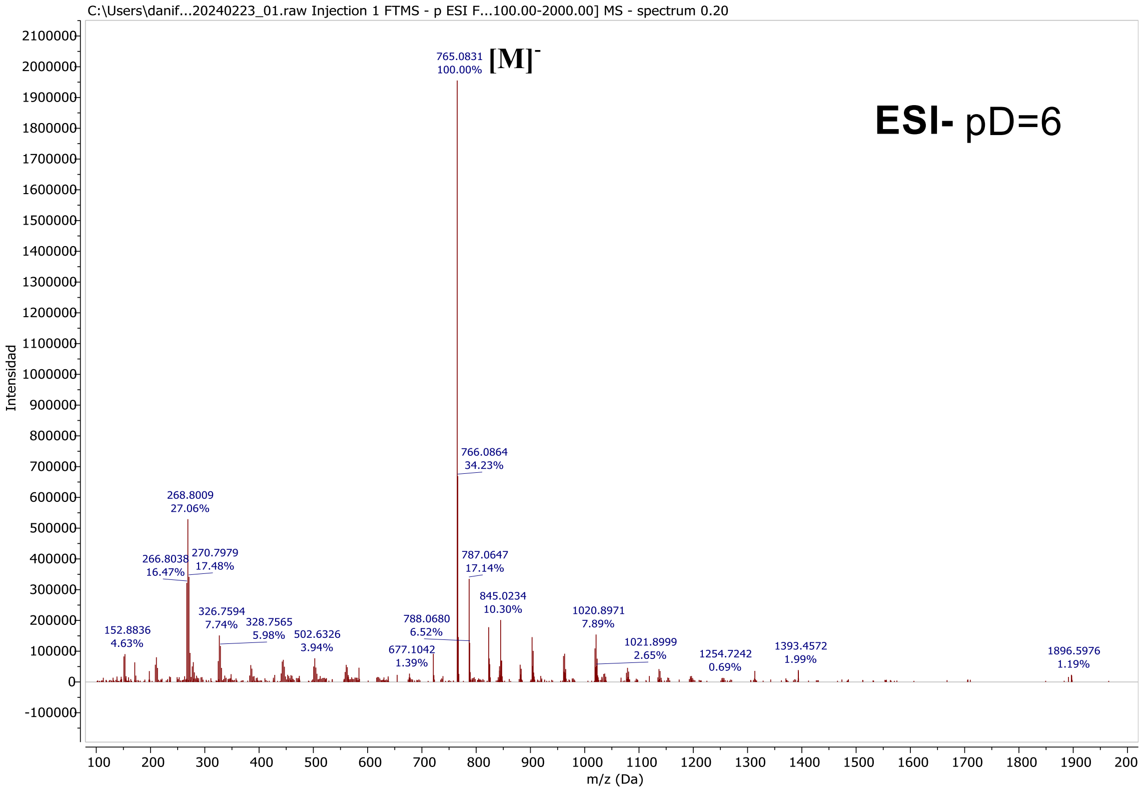

Figure S113. HR-MS spectra of [La(**tpadapo**)]$^-$.

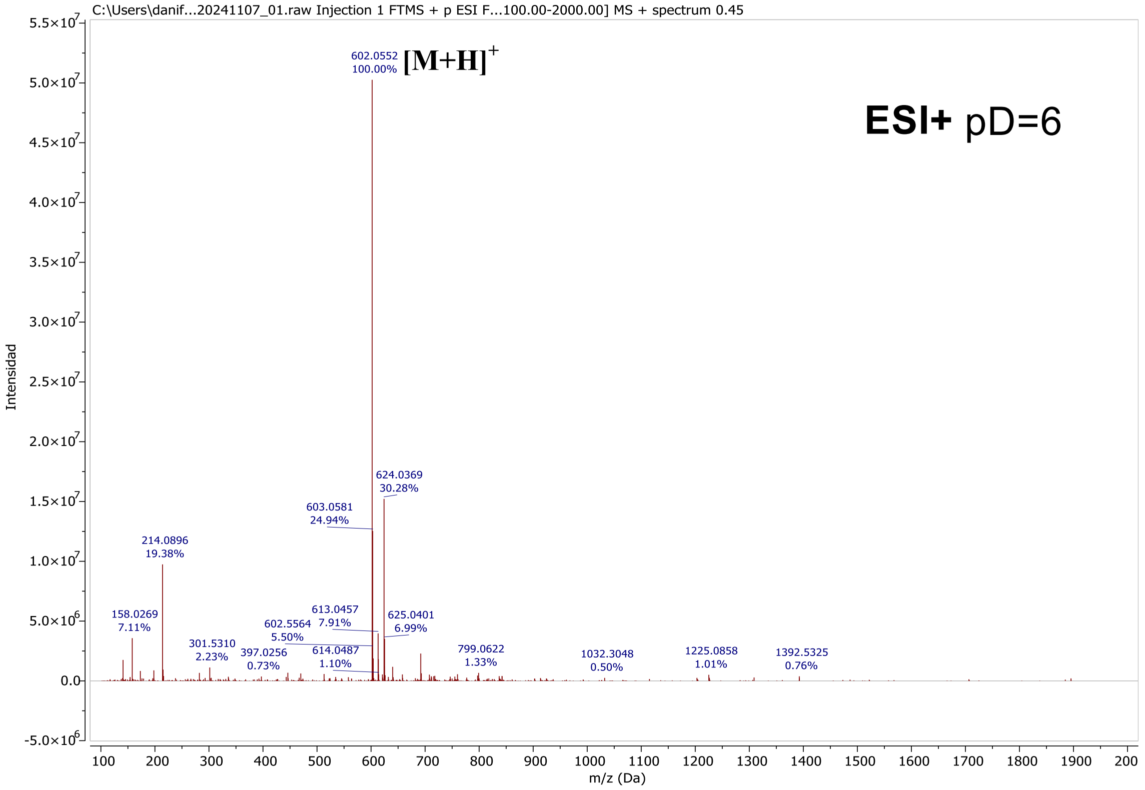

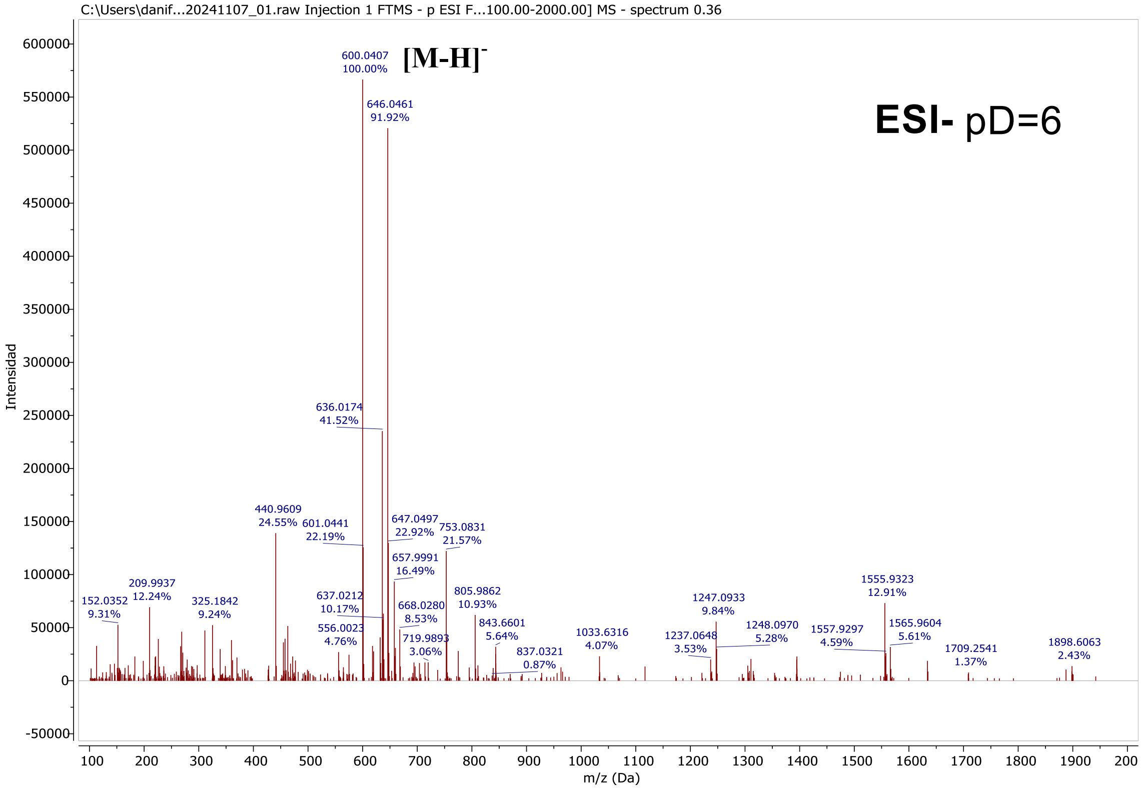

Figure S114. HR-MS spectra of [La(**tripaen**)].

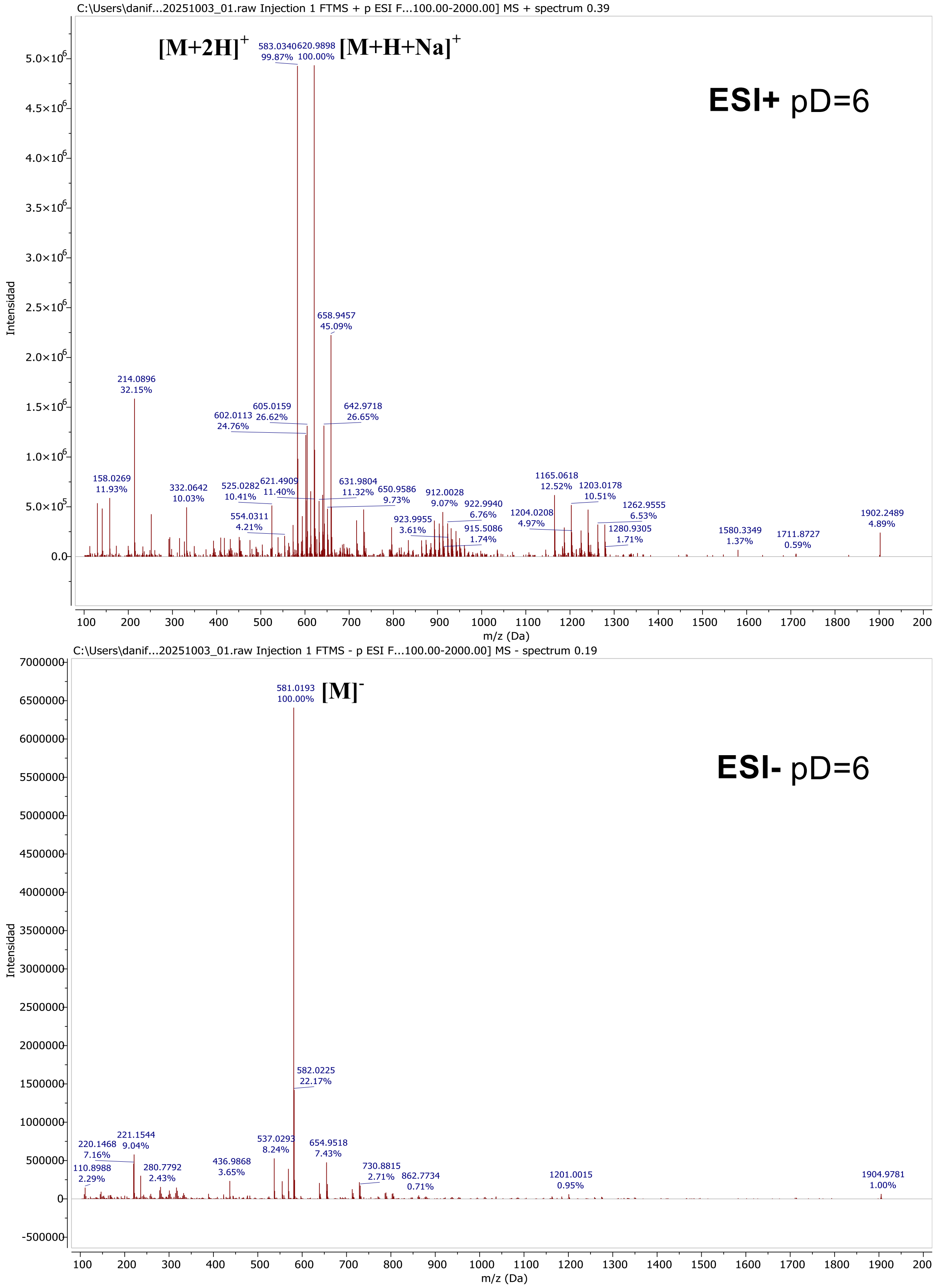

Figure S115. HR-MS spectra of [La(**asyoctapa**)]$^{-}$.

Mass Spectrometry of cold terbium(III) complexes

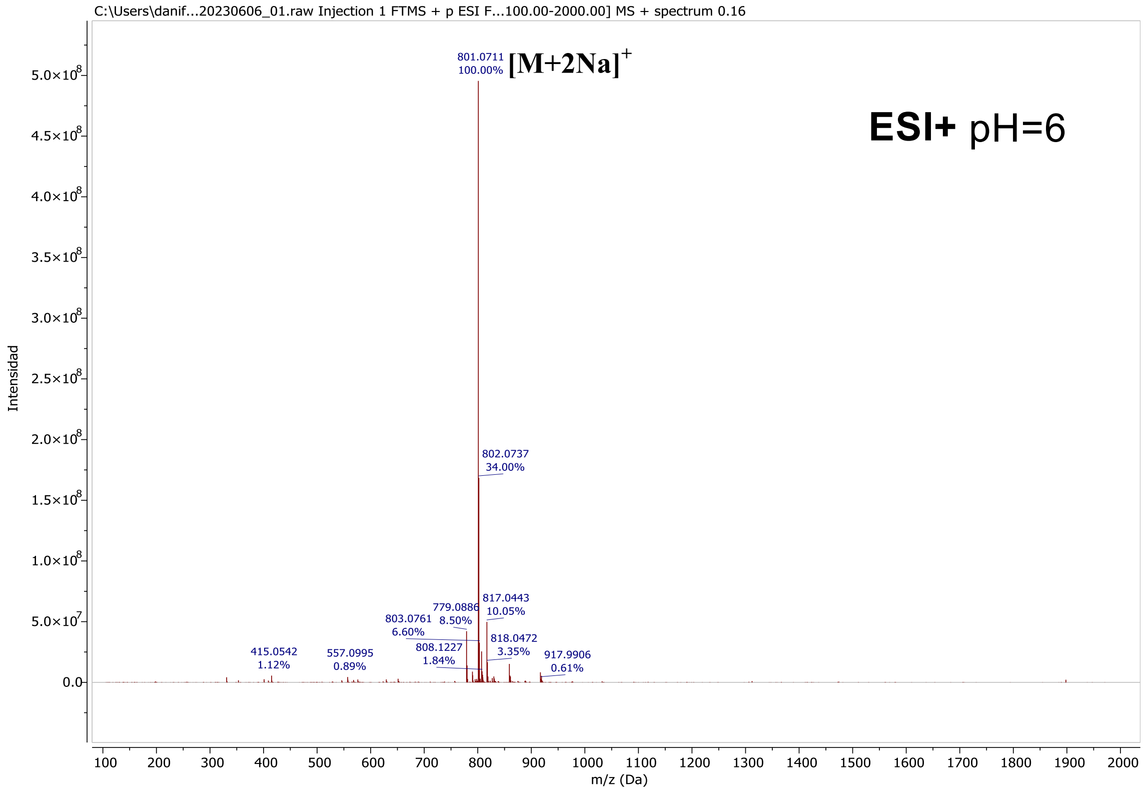

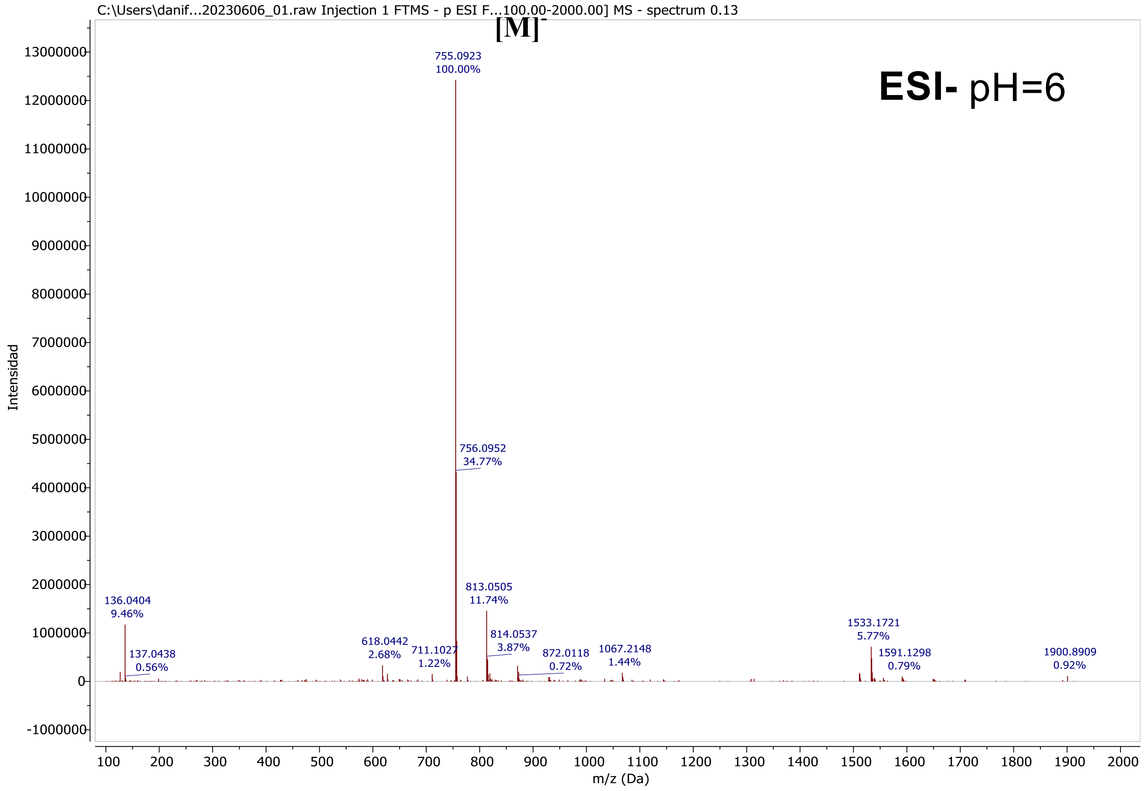

Figure S116. HR-MS$^-$ spectra of [Tb(**tpaen**)]$^-$.

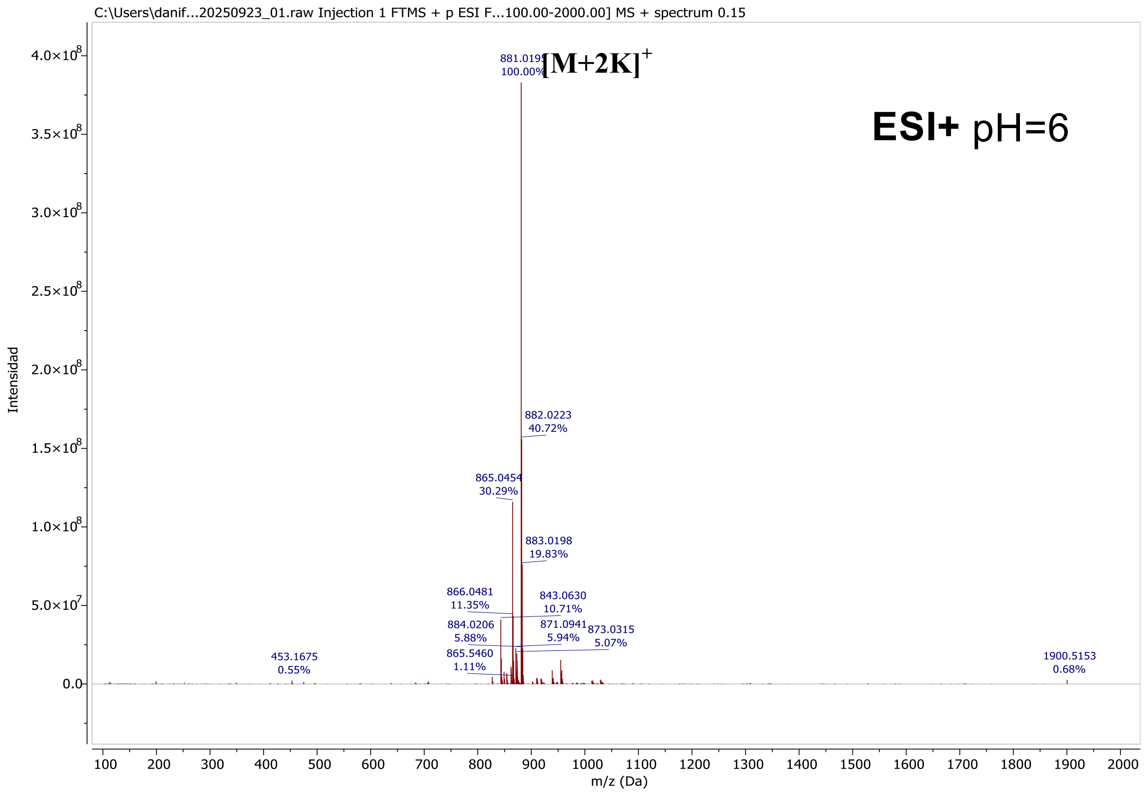

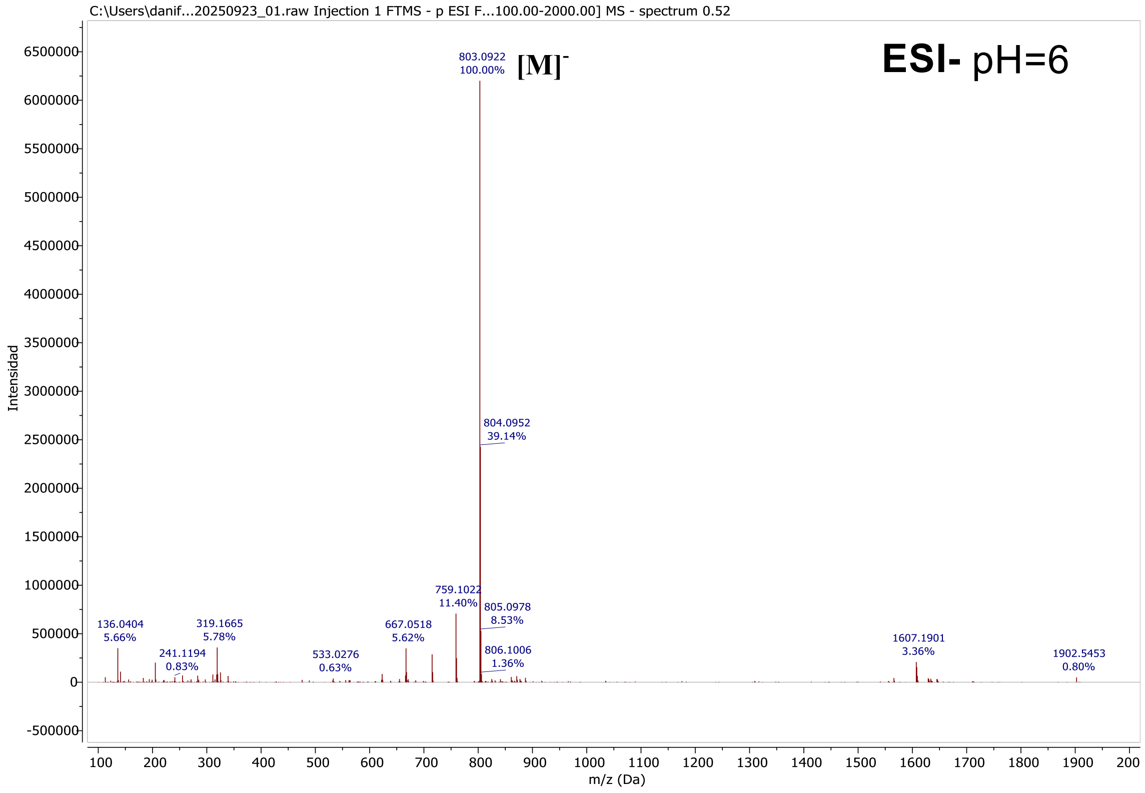

Figure S117. HR-MS spectra of [Tb(**tpaopd**)]$^{-}$.

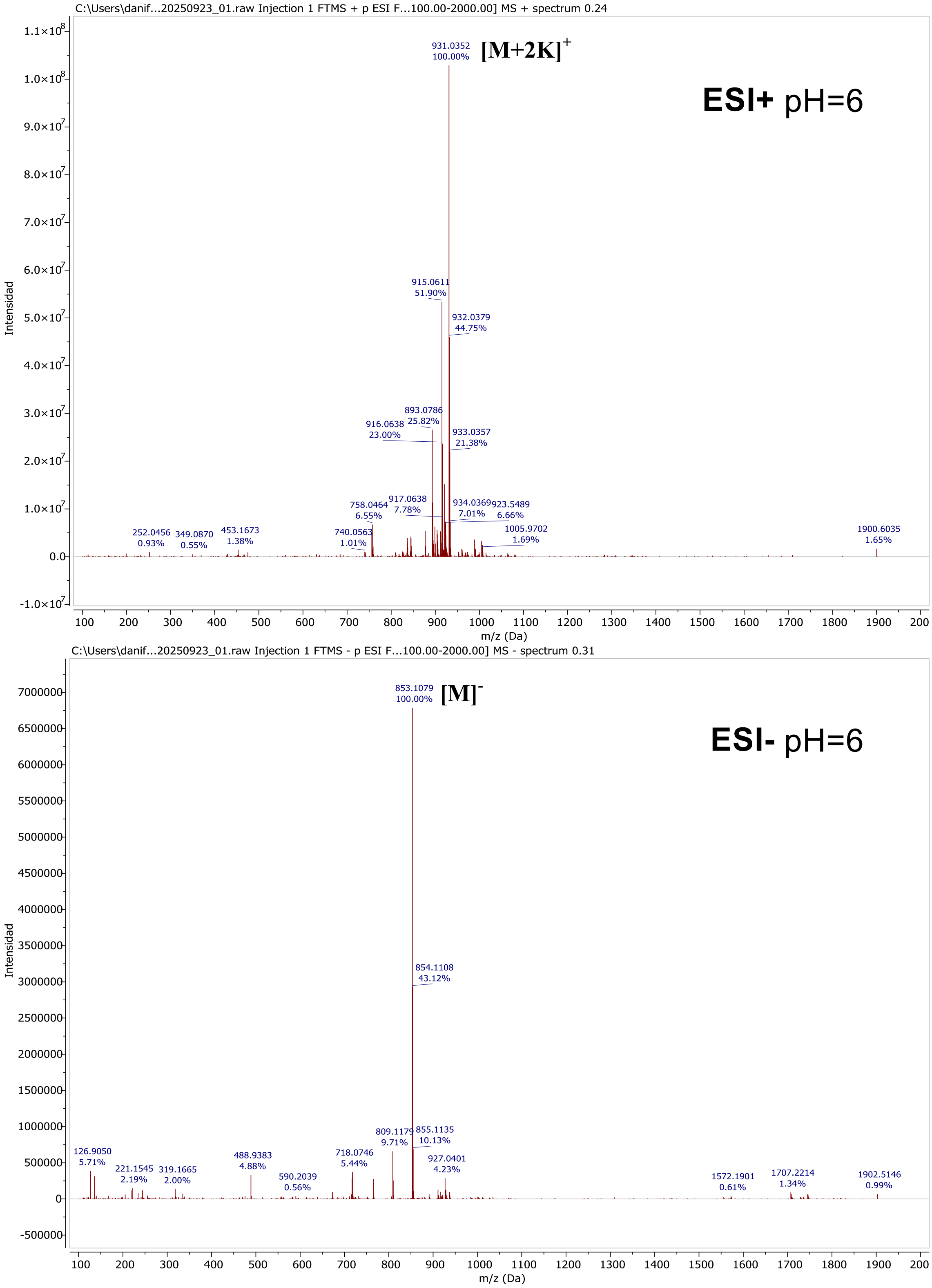

Figure S118. HR-MS spectra of [Tb(**tpaond**)]$^-$.

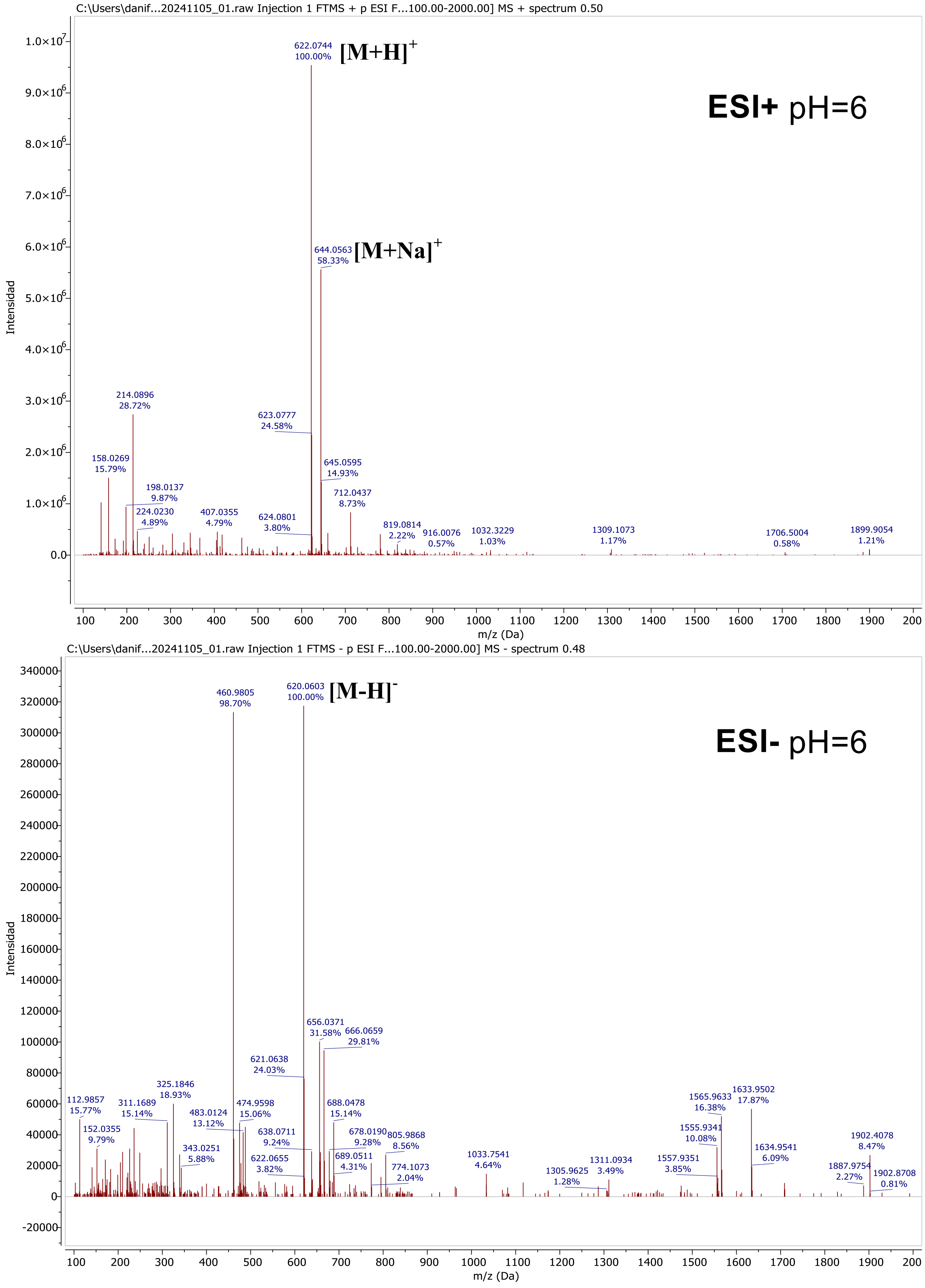

Figure S119. HR-MS spectra of [Tb(**tripaen**)].

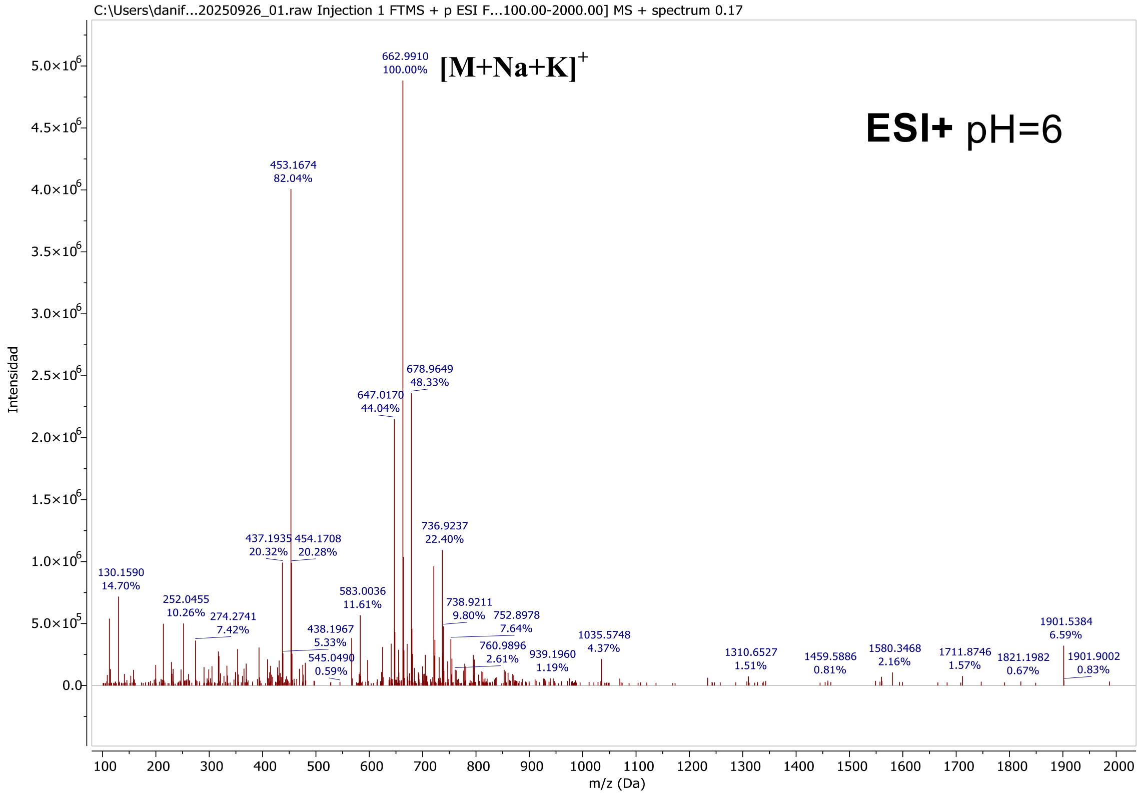

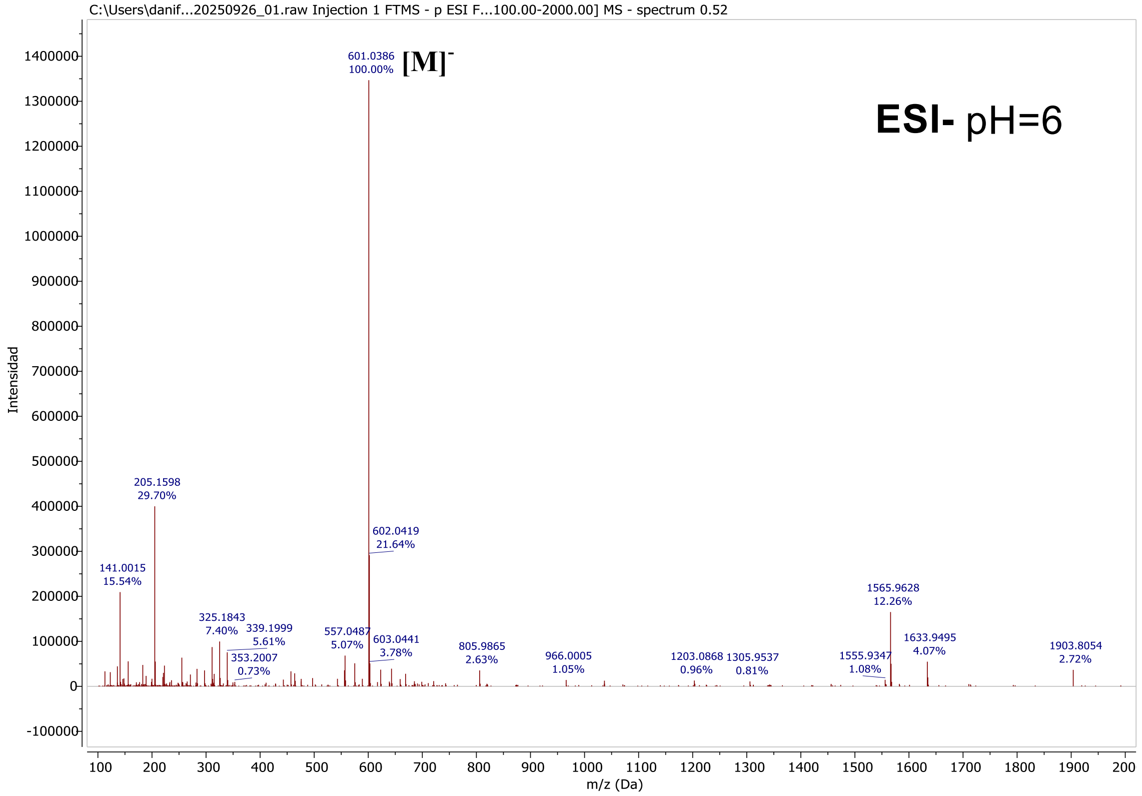

Figure S120. HR-MS$^-$ spectra of [Tb(**asyoctapa**)]$^-$.

Mass Spectrometry of cold lutetium (III) complexes

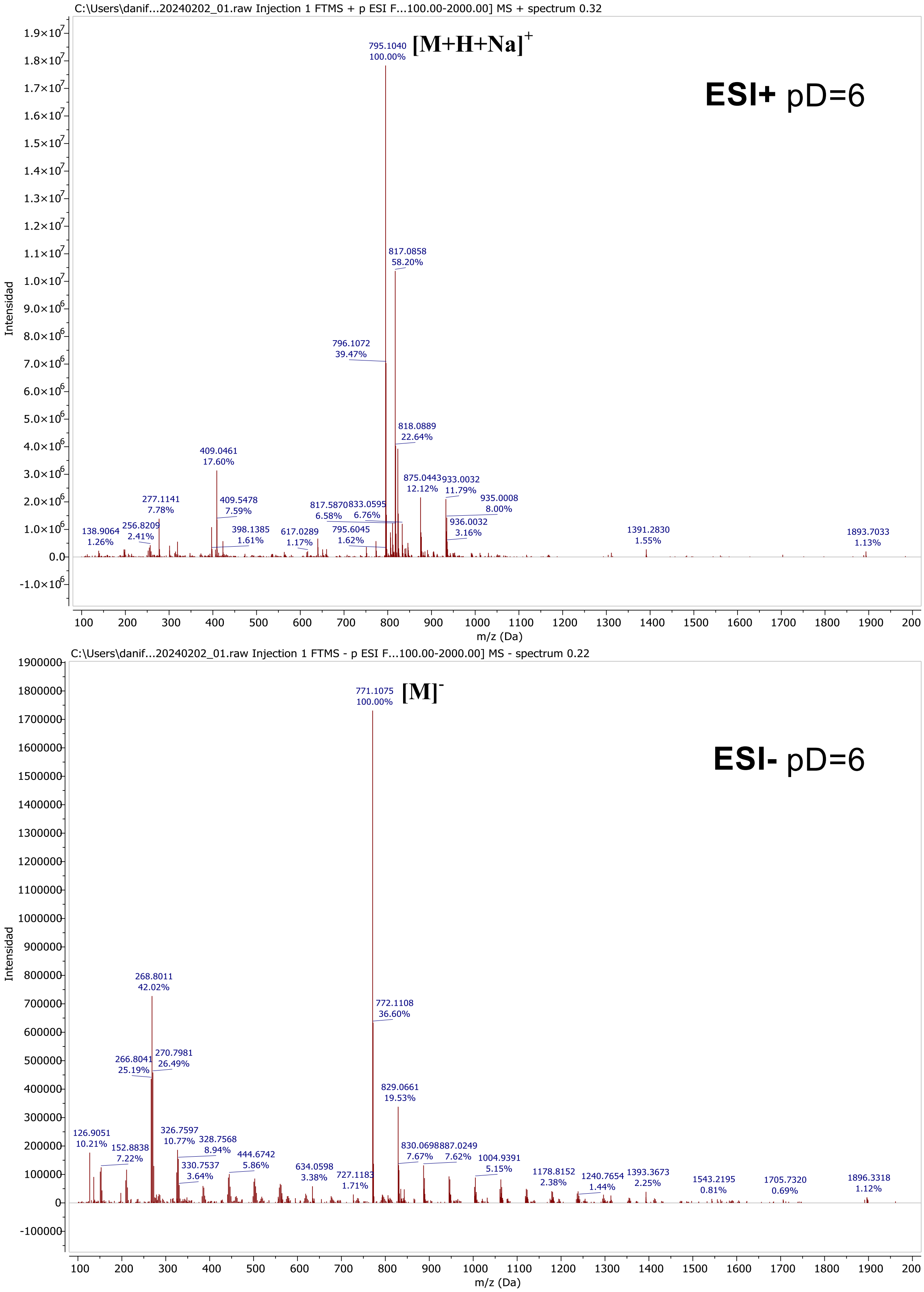

Figure S121. HR-MS spectra of [Lu(**tpaen**)]$^-$.

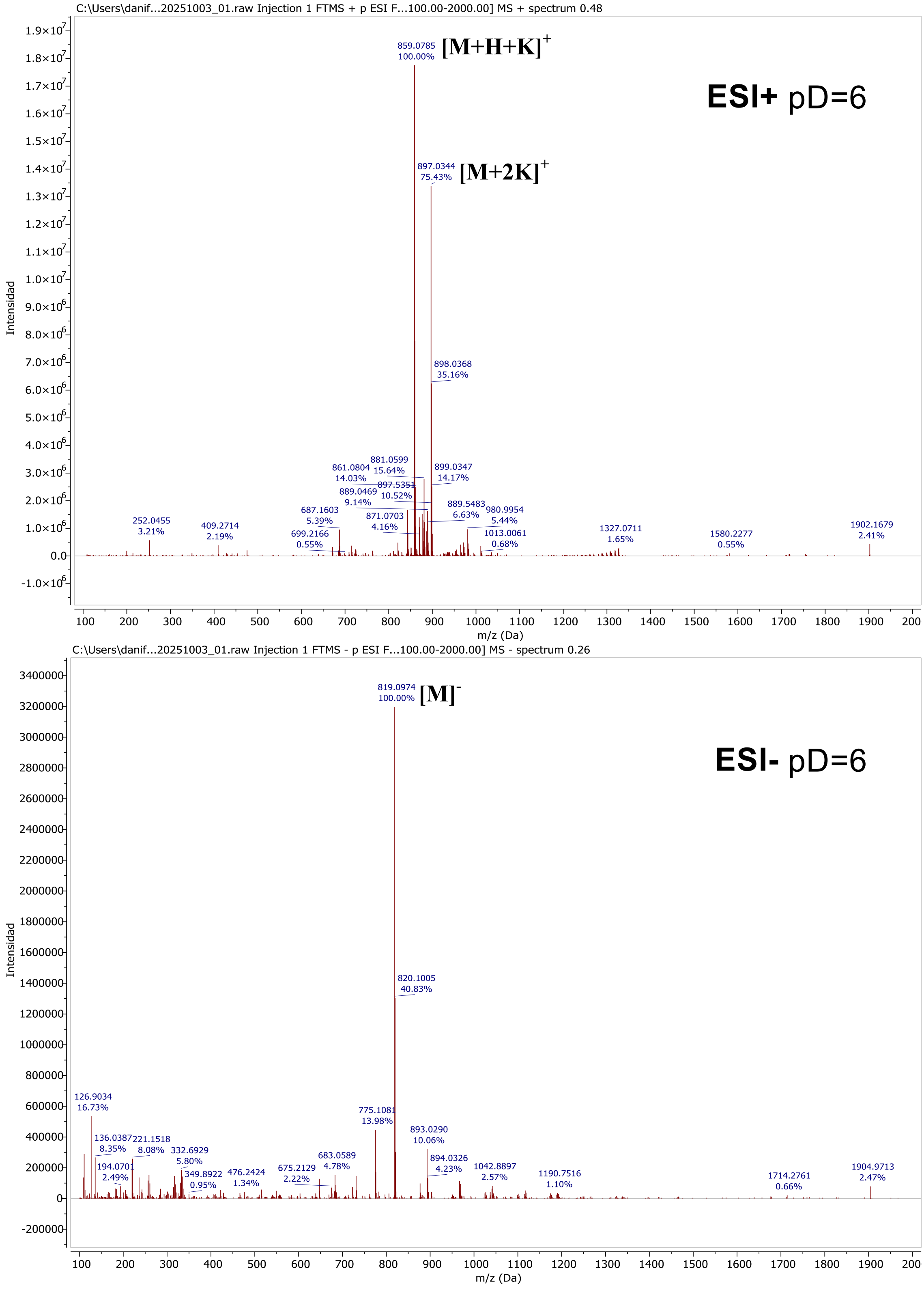

Figure S122. HR-MS spectra of [Lu(**tpaopd**)]$^-$.

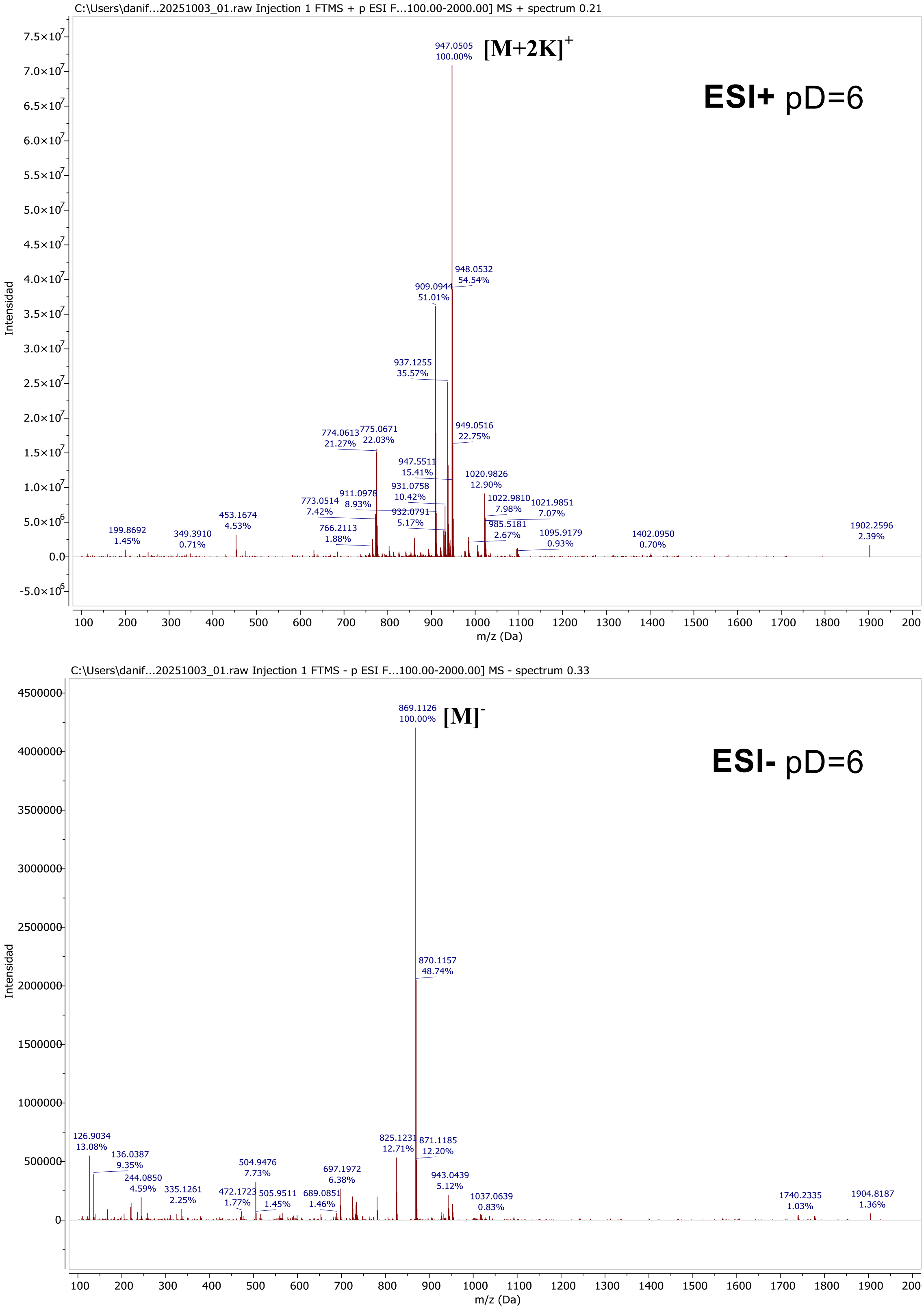

Figure S123. HR-MS spectra of [Lu(**tpaond**)]$^-$.

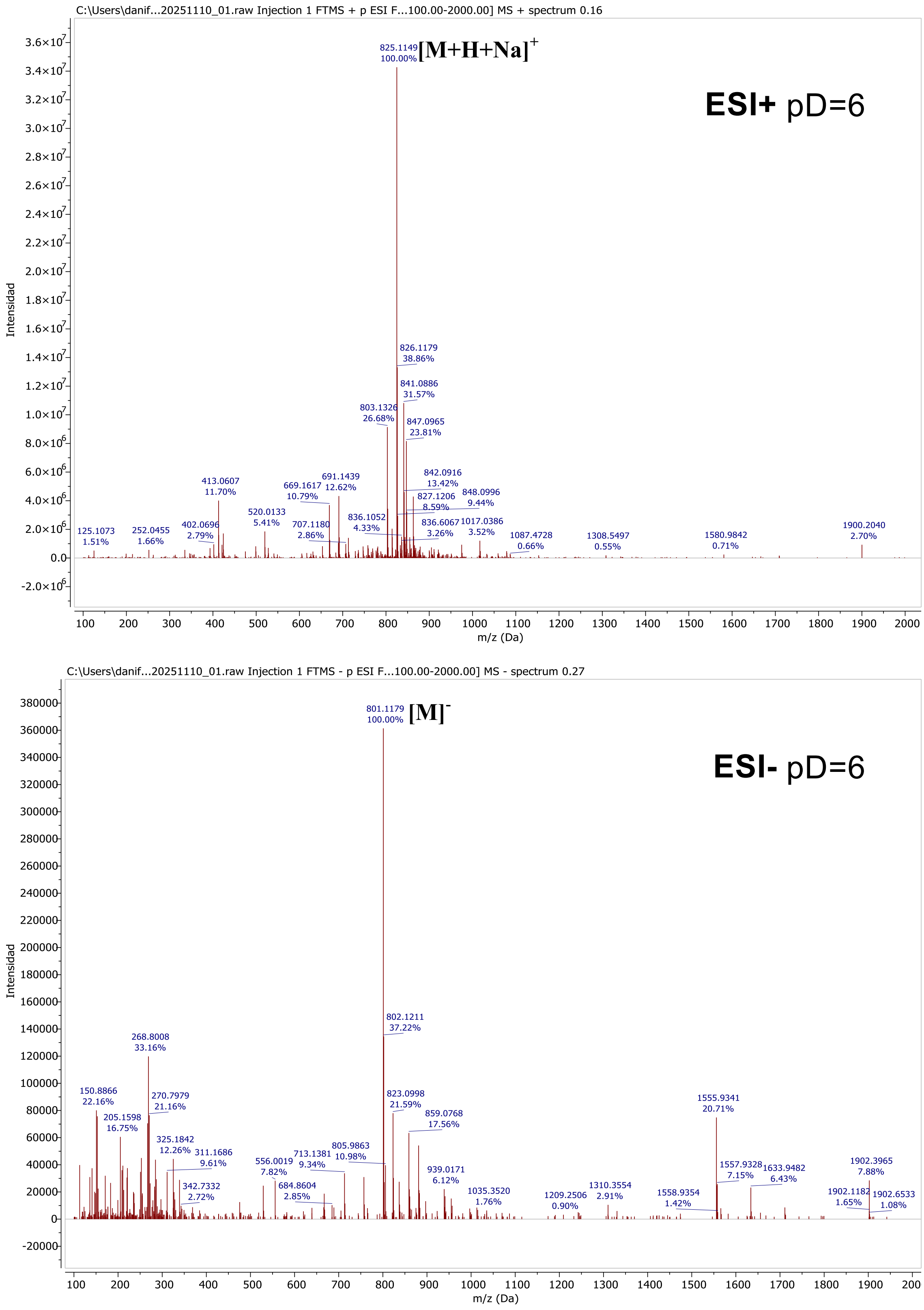

Figure S124. HR-MS spectra of [Lu(**tpadapo**)]$^-$.

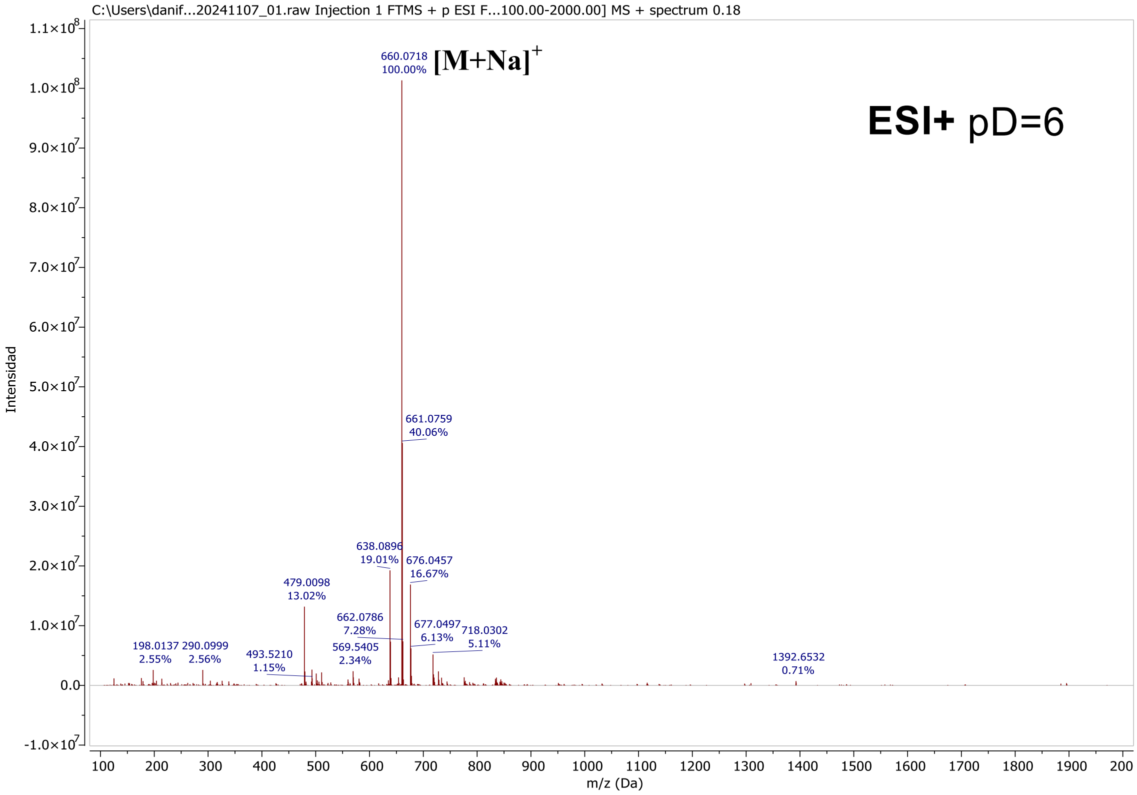

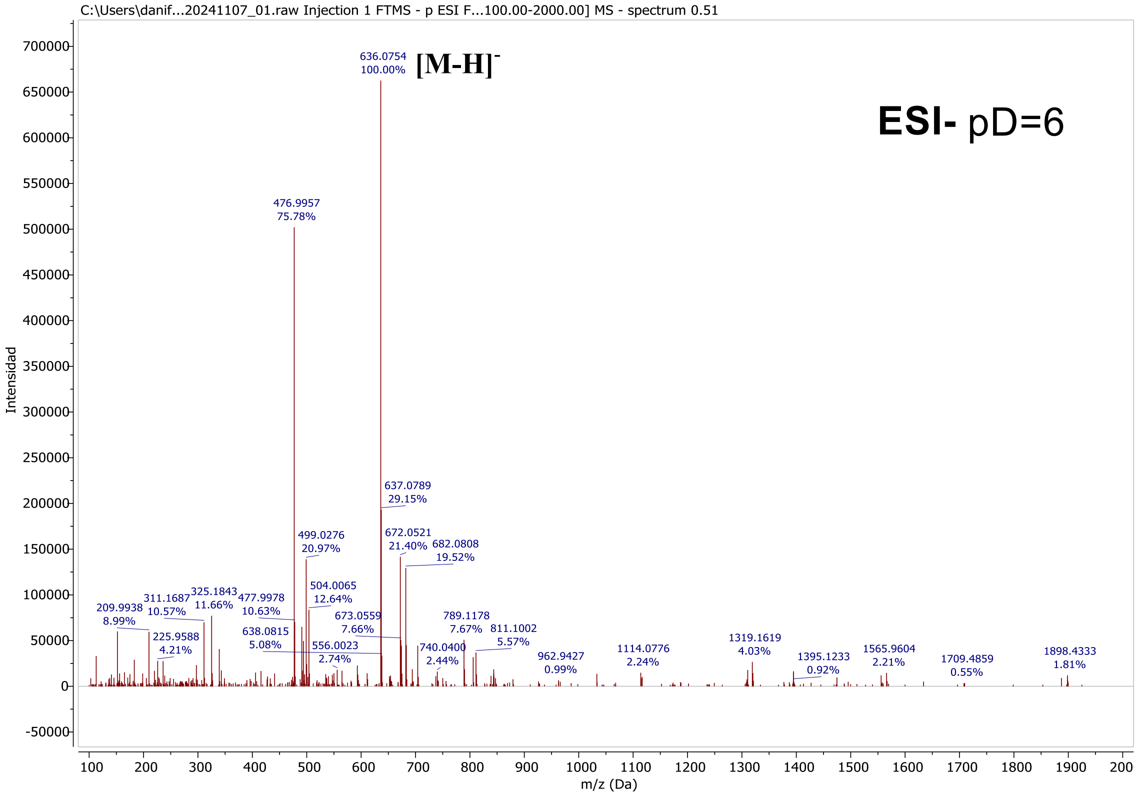

Figure S125. HR-MS spectra of [Lu(**tripaen**)].

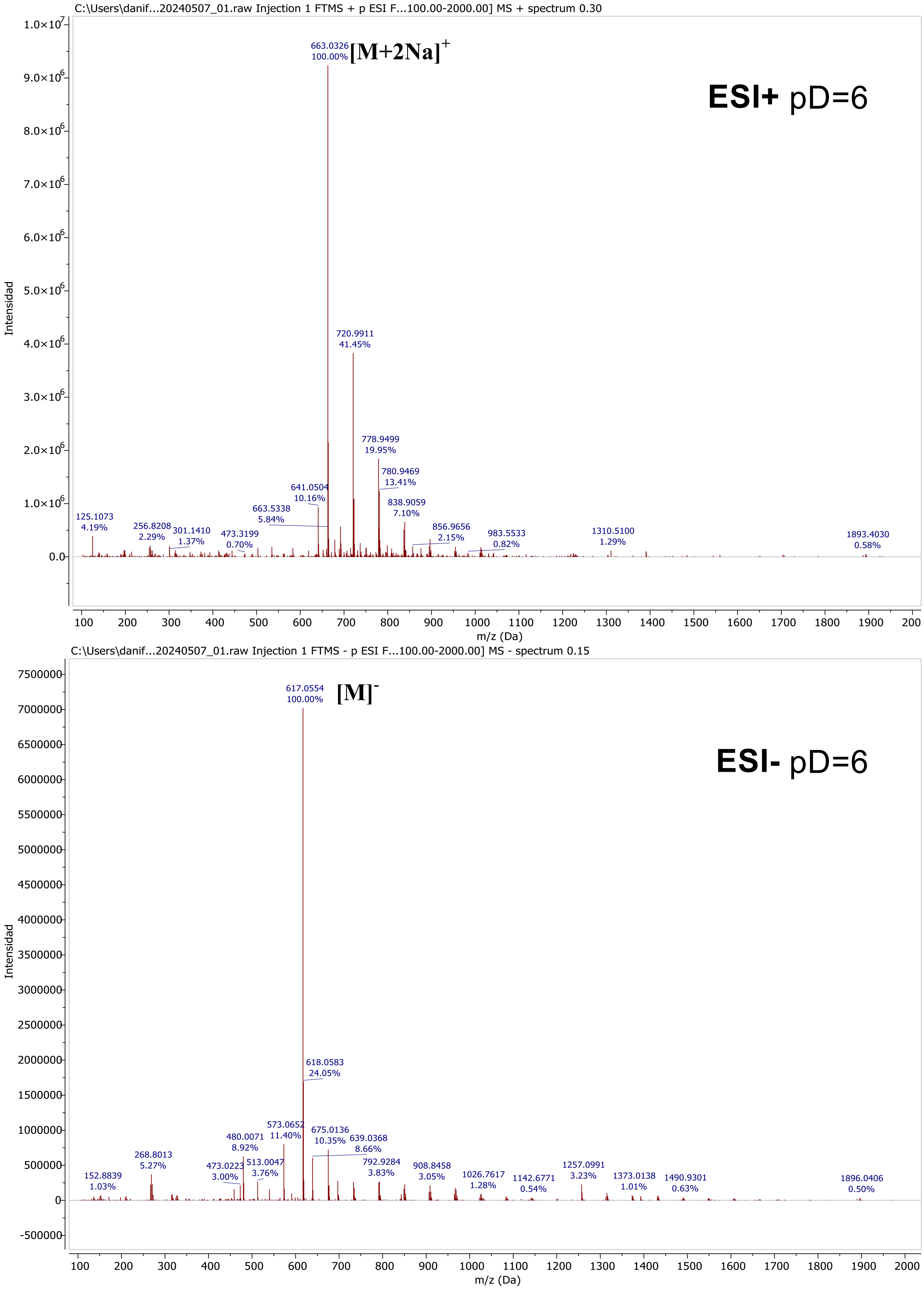

Figure S126. HR-MS spectra of [Lu(**asyoctapa**)]$^-$.

Solution Thermodynamics

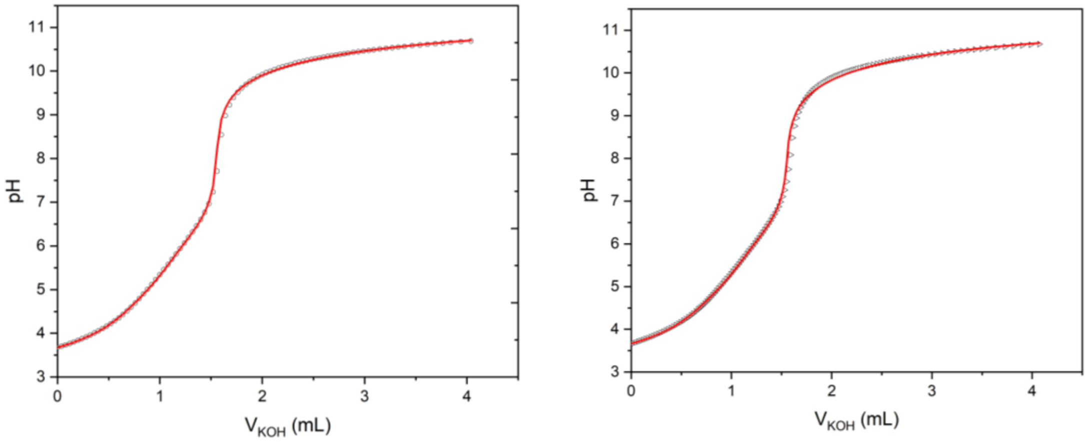

H$_4$tpaopd

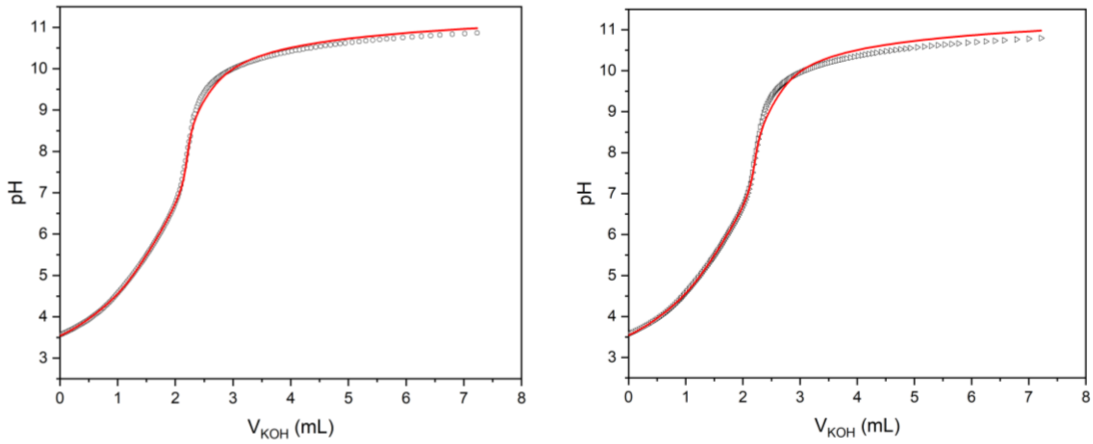

H$_4$tpaond

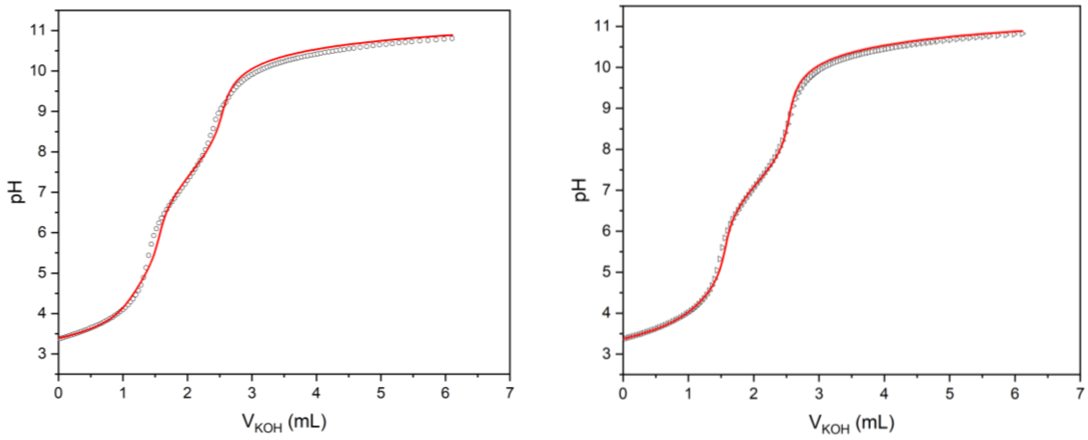

H$_4$tpamxd

Figure S127. Titrations for chelators (two replicates). Dots and triangles represent experimental measurements, and the red line represents the fitting performed by *HyperQuad2013*.

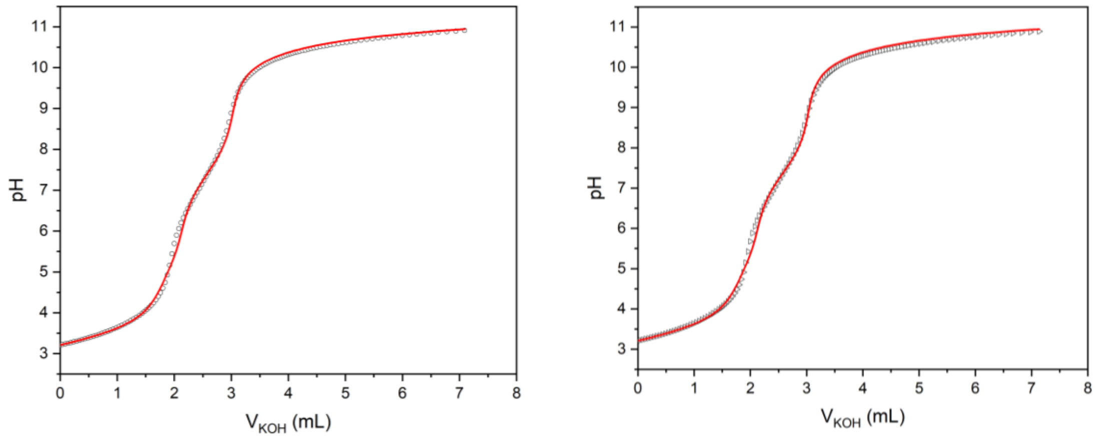

H_4tpapxd

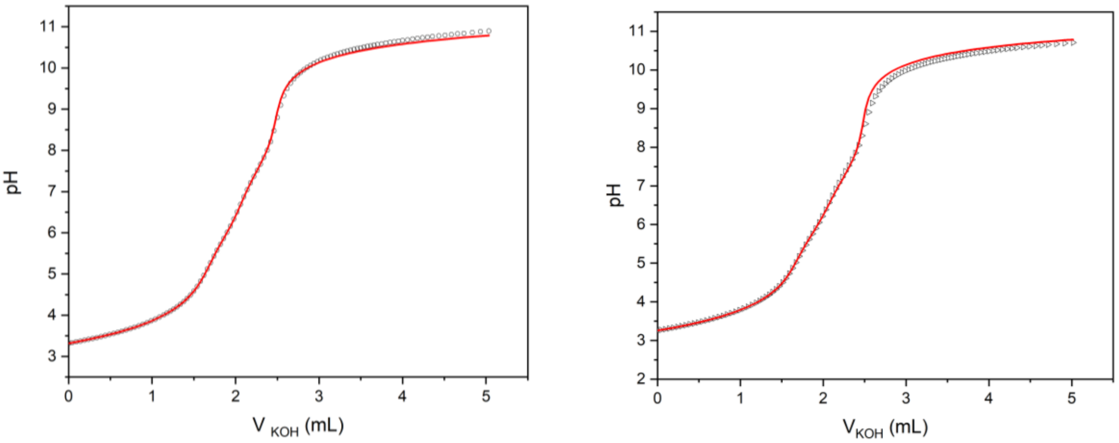

H_4tpadapo

Figure S127 (continuation). Titrations for chelators (two replicates). Dots and triangles represent experimental measurements, and the red line represents the fitting performed by *HyperQuad2013*.
